# Holistic Assessment of Actinide-Borohydride Covalency Using NMR and X‑ray Absorption Spectroscopies

**DOI:** 10.1021/jacs.6c02674

**Published:** 2026-09-22

**Authors:** Hannah M. Hansen, Nathan R. Loutsch, Thien H. Nguyen, Aaron Christianson, Jacob J. Schuely, Mai Yer Yang, Peter A. Zacher, Lucia Zuin, Trevor W. Hayton, Bess Vlaisavljevich, Scott R. Daly

**Affiliations:** † Department of Chemistry, 4083 The University of Iowa, E331 Chemistry Building, Iowa City, Iowa 52242, United States; ‡ Department of Chemistry and Biochemistry, 8786 University of California Santa Barbara, Santa Barbara, California 93106, United States; § 117197 Canadian Light Source, University of Saskatchewan, 44 Innovation Place, Saskatoon, Saskatchewan S7N 2V3, Canada

## Abstract

The influence of metal-ligand covalency on actinide electronic properties and reactivity continues to be a topic of intense interest given its implications for nuclear and radiochemical applications. Extensive work has focused on developing spectroscopic methods that can be used to quantify metal-ligand covalency within actinide complexes for benchmarking and comparison with theoretical efforts. Ligand K-edge X-ray absorption spectroscopy (XAS) and NMR spectroscopy are two physical methods that have dominated these efforts, but surprisingly, they have not been used together on the same systems to see how the methods compare. Herein, we report the first comparative assessment of actinide-ligand covalency using B K-edge XAS and multinuclear NMR spectroscopy. The B K-edge XAS spectra of An­(κ^3^-BH_4_)­(NR_2_)_3_ (R = SiMe_3_) with An = Th (**1**) and U (**2**) reveal a dominant feature assigned to B 1s → An–H–B π* MOs due to covalent three-center, two-electron bonding with the actinide 6d orbitals, as revealed by comparison with time-dependent density functional theory (TDDFT) calculations. By contrast, the data indicate that 5f-orbital contributions to the bonds are significantly smaller. These findings were corroborated by ^1^H and ^11^B NMR studies of diamagnetic **1** and DFT calculations used to quantify the impact of orbital mixing via spin-orbit coupling on the chemical shifts. These results represent the first instance of using B K-edge XAS and ^11^B NMR spectroscopy to assess covalency in actinide complexes. Moreover, they demonstrate how ligand K-edge XAS, which provides rich orbital-specific information relevant to chemical bonding, and NMR spectroscopy, which is routinely available in most chemical research settings, can be used as complementary techniques to provide a holistic assessment of covalency in actinide-ligand bonds.

## Graphical abstract

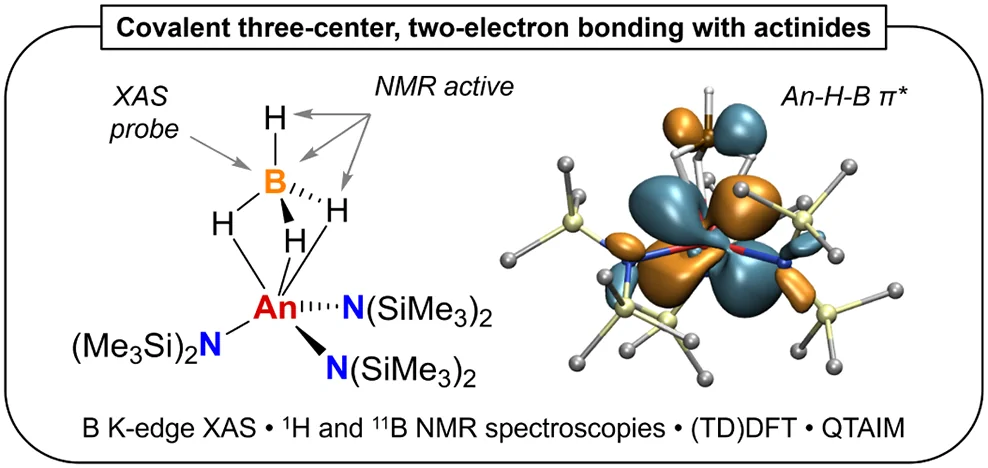

## Introduction

Quantifying variations in metal-ligand (M-L) covalency remains an important objective in actinide science given its critical influence on the reactivity and properties of f-elements.^1–8^ For example, successful separation of trivalent lanthanides and actinides is often attributed to their differing M-L covalency with extractant molecules, as first hypothesized over 70 years ago.^9^ These early qualitative observations subsequently spurred the development of experimental methods that could quantify the extent of covalency in actinide-ligand bonds.

One physical method that has proven exceptionally successful for measuring M-L covalency in actinide complexes (as well as those with lanthanides)^10–15^ is ligand K-edge X-ray absorption spectroscopy (XAS).^16–22^ This technique uses synchrotron generated X-rays to probe dipole-allowed transitions between core 1s orbitals localized on the ligand and vacant molecular orbitals (MOs) derived from M-L bonds.^23^ The presence and intensity of the associated transitions are governed by the amount of ligand *n*p character mixing in the corresponding MOs, thereby providing an experimental measure of orbital mixing.^5^ Though this technique can afford orbital-level resolution of electronic structure and bonding, XAS is not readily accessible to most chemists due to limited access to facilities and technical barriers associated with sample preparation and data collection, especially on air-sensitive systems (Figure 1).

**Figure 1:**
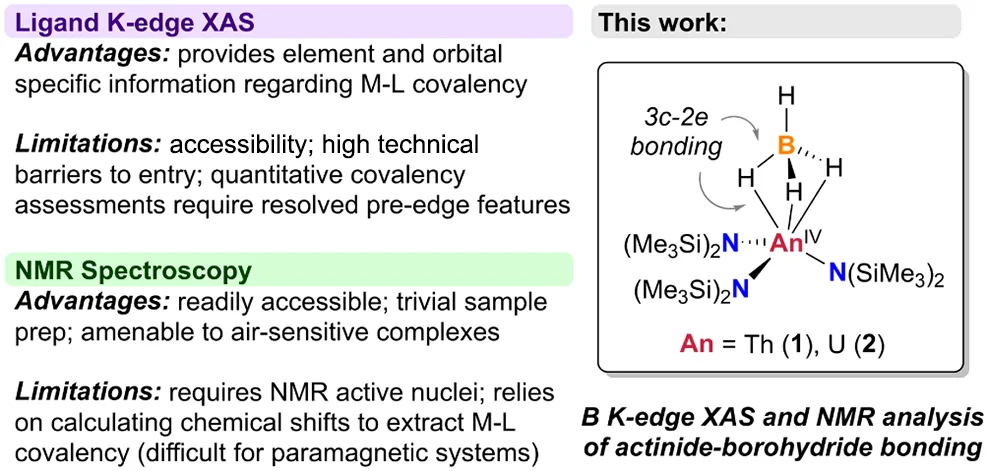
*Left*—Comparative characteristics of ligand K-edge XAS and NMR spectroscopy for evaluating metal-ligand covalency. *Right*—This work: B K-edge XAS and NMR studies of covalent three-center, two electron (3c–2e) actinide-borohydride bonding in **1** and **2**.

An alternative method to ligand K-edge XAS, that has also been used to quantify M-L covalency in actinide-ligand bonds, is NMR spectroscopy.^24–27^ NMR spectroscopy is widely available for researchers, in contrast with synchrotron-based methods like XAS. Though it is much more accessible, it relies more heavily on comparison to theory to abstract orbital-specific covalency information, which typically limits this approach to diamagnetic complexes due to the challenge of adequately modeling NMR shifts for paramagnetic complexes (though this is starting to be addressed).^28^ This NMR-based approach relies on measuring the chemical shifts (δ) and nuclear shielding constants (σ) of actinide-bound nuclei, the latter of which is comprised of three components, as shown in eq 1,

1 $${\sigma =\sigma }_{\mathrm{dia}}{+\sigma }_{\mathrm{para}}{+\sigma }_{\mathrm{SO}}$$

where σ_dia_, σ_para_, and σ_SO_ refer to the diamagnetic, paramagnetic, and spin-orbit contributions, respectively. The magnitude of σ_SO_ is the crucial component for assessing M-L covalency, since this component correlates with the amount of ligand s-character and M-L orbital overlap present in the bonds.^24^ The σ_SO_ value is obtained indirectly by using DFT to quantify the difference in σ values with scalar relativistic (SR) and spin-orbit (SO) treatment.^29–32^ The chemical shift attained with SO treatment (δ_SO_) is directly comparable to experiment. This approach has been demonstrated for f-block coordination complexes with ligands containing NMR-active nuclei such as ^13^C,^33–39^ ^15^N,^40–42^ ^29^Si,^43^ ^31^P,^44^ ^77^Se,^45^ and ^125^Te.^45^ It has also been used to analyze the ^1^H NMR shifts of Pb hydride and Tl borohydride complexes,^46,47^ as well as hydride complexes with Zr, Hf, Th, and U.^48^

Both ligand K-edge XAS and NMR spectroscopy (as well as EPR spectroscopy)^49,50^ have been used to investigate M-L covalency in actinide complexes containing ligands with many of the p-block elements, but a notable exception is boron. In this context, there have been longstanding questions about covalency in actinide borohydride complexes and its impact on molecular properties.^51^ Homoleptic actinide borohydrides, like U­(BH_4_)_4_, are remarkably volatile and were investigated for isotopic separations during the Manhattan Project.^52–59^ Trivalent actinide borohydride complexes are also known to exhibit structural and reactivity differences consistent with increased covalency when compared to isomorphous complexes with lanthanides.^60–63^

Here we describe a combined B K-edge XAS and NMR study of An­(κ^3^-BH_4_)­(NR_2_)_3_,^64^ where An = Th (**1**) and U (**2**) and R = SiMe_3_, that reveals corroborating evidence of covalent actinide-borohydride bonding (Figure 1). These complexes were selected for B K-edge XAS studies because they have relatively high (axial) symmetry at the metal and boron. The (BH_4_)^1–^ ligand also offers numerous advantages for these studies. It is electronically simple, lacking substituents and competing π systems. Accordingly, the NMR shifts are dominated by metal interactions, which should make spin-orbit shifts easier to interpret as a covalency metric. Perhaps most importantly, the same B 2p orbitals that result in B K-edge XAS features are also involved in the spin-orbit deshielding observed in the NMR spectra, which allows the two techniques to be directly compared.

Though boron is not directly bound to the metals, some of us recently showed how the delocalized nature of M–H–B bonding (*i.e.,* three-centered, two-electron bonds)^65^ allows boron to serve as a reporter of covalent mixing in Zr- and Hf-borohydride bonds using B K-edge XAS.^66^ We demonstrate here that the same approach can be used to interrogate actinide-borohydride bonds. Borohydrides also have two common NMR-active nuclei, ^1^H and ^11^B, which permitted us to evaluate how the covalency assessments from B K-edge XAS compared to those obtained from NMR spectroscopy experiments with diamagnetic **1**. To our knowledge, this is the first instance where both methods have been used to assess covalency in the same complex.

## Results and Discussion

Complexes **1** (An = Th) and **2** (An = U) were prepared as described previously by treating AnCl­(NR_2_)_3_ (R = SiMe_3_) with LiBH_4_ in Et_2_O.^64^ The complexes were crystallized from pentane, and their structures confirmed by single-crystal XRD. The structure of **1** had been described previously,^64^ but was recollected here, while the structure of **2** had yet to be reported (Figure 2a). Both complexes feature C_3_ point group symmetry with An-B distances of 2.64(1) and 2.55(1) Å, respectively, consistent with a κ^3^-BH_4_ binding mode and expected differences in Th^4+^ and U^4+^ radii.^67^ The ^1^H NMR spectra collected in C_6_D_6_ matched those reported previously.^64^ A 1:1:1:1 quartet assigned to BH_4_ was observed at δ_H_ = 4.25 (**1**; ^1^ *J*_HB_ = 83 Hz) and δ_H_ = −99.95 ppm (**2**; ^1^ *J*_HB_ = 78 Hz) (Figure 2b). These assignments were confirmed by synthesizing the BD_4_ analogues, **1-*d*_4_** and **2-*d*_4_** (see ESI for details). The ^1^H NMR data indicates that the terminal and bridging hydrides are equivalent on the NMR time scale due to rapid exchange, as reported for other tetrahydroborate complexes.^51^ The ^11^B NMR spectra revealed the complementary BH_4_ resonances at δ_B_ = −8.0 ppm (**1**) and δ_B_ = −10.0 ppm (**2**). Cooling a CD_2_Cl_2_ sample of either **1** or **2** to −40 °C results in no decoalescence of the hydride resonances, which is not surprising since the barrier for exchange is known to be extremely small.^68^

**Figure 2:**
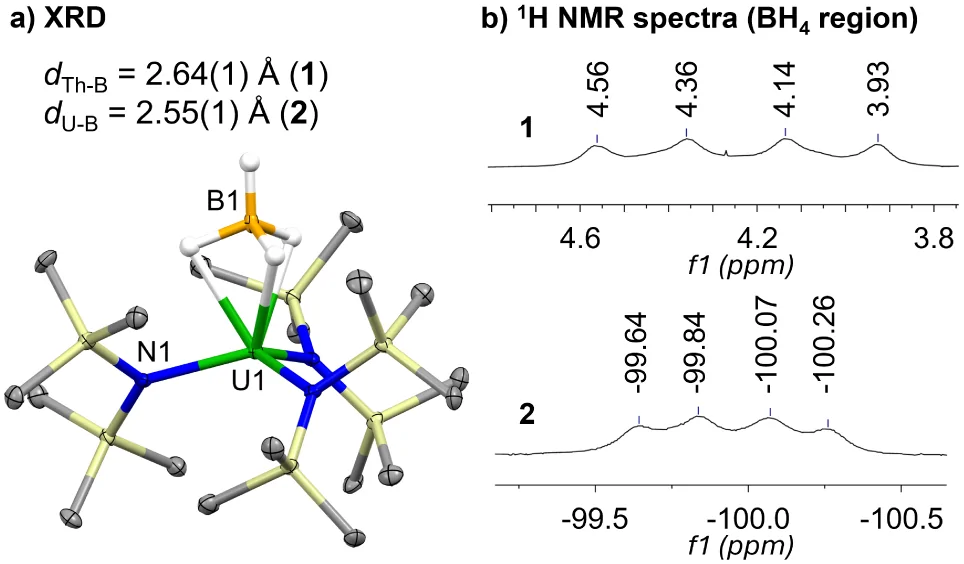
a) Molecular structure of **2** (An = U) and An-B distances from XRD. Ellipsoids drawn at 50% probability. Hydrogen atoms attached to carbon are omitted. b) ^1^H NMR resonances for the (BH_4_)^1–^ ligand in **1** and **2** in C_6_D_6_.

B K-edge XAS data were collected at room temperature on solid samples of **1** and **2** at the Canadian Light Source (CLS) using the Variable Line Spacing Plate Grating Monochromator (VLS-PGM) beamline.^69^ Samples were finely ground in a mortar and pestle and pressed into In foil. Total fluorescence yield (FLY) and total electron yield (TEY) data were collected simultaneously. The pressure in the experimental chamber was in the range of 10^–8^ to 10^–9^ Torr (UHV) during the measurements. We note that TEY measurements are surface sensitive, primarily due to the low energy and escape depth of electrons,^70,71^ whereas FLY measurements are bulk sensitive as they probe deeper into the material due to photons’ decreased interaction with matter.^71^

It is important to start by noting that these are remarkably challenging measurements by XAS standards given the very high air- and water-sensitivity of **1** and **2**, in combination with the relatively low B K-edge energy (ca. 188 eV). Any materials that are normally used to protect samples from air during sample loading (e.g., polypropylene, Kapton) block the beam due to the very short attenuation length of light at this energy. Despite the challenges, we have developed robust procedures for delivering and loading samples in the chamber with minimum hydrolysis (see Experimental Section in the Supporting Information for full details).

Another challenge is photodecomposition. We have previously shown that molecular borohydride and dicarbollide complexes can readily undergo photodecomposition during B K-edge XAS data collection.^66,72^ To help distinguish between spectral features for **1** (An = Th) and **2** (An = U) from those associated with photodecomposition, B K-edge XAS data were collected using two different scanning techniques: step scans and continuous scans (Table 1). To minimize light on the sample and attenuate potential photodecomposition to the best of our ability, step scans with 0.5 s dwell times were collected using a piezoelectric (PZ) shutter that closed the beam between energy moves. We also moved to a new sample position after every step scan, unless noted otherwise. The second scanning technique, continuous scans, used longer 1.0 s intervals and no PZ shutter. These scans were used to intentionally induce and monitor for photodecomposition, as described in the aforementioned B K-edge XAS studies of Ni­(C_2_B_9_H_11_)_2_ and M­(κ^3^-RBH_3_)_4_ complexes with M = Zr, Hf and R = aryl and benzyl.^66,72^

**Table 1:** Characteristics of Differing B K-Edge Scans Used in This Study (PZ = Piezoelectric)

**Scan type**	Step scan (SS)	Continuous scan (CS)
**PZ shutter**	yes	no
**Dwell time**	0.5 s	1.0 s

The step scan data (with PZ shutter) collected for **1** and **2** revealed four main features in the spectra of both complexes, as evident in the second derivative traces (Figure 3; Table 2). Each spectrum possessed a low-resolution pre-edge shoulder at 189.9 eV (**1**) and 189.8 eV (**2**) (labeled A in Figure 3), and a more prominent second feature labeled B at 190.7 eV (**1**) and 190.8 eV (**2**). For comparison, these features were not observed in the B K-edge XAS spectrum of NaBH_4_ when collected using step scans; only a broad rising edge feature was observed at 191.4 eV (Figure 3; gray trace), consistent with values reported in the literature.^73^ The step scan spectra also revealed two additional features at higher energy above the edge (C and D) with slight shifts in energy depending on the metal. These features appeared at 192.5 and 193.8 eV when An = Th (**1**), and 192.2 and 194.8 eV when An = U (**2**).

**Figure 3:**
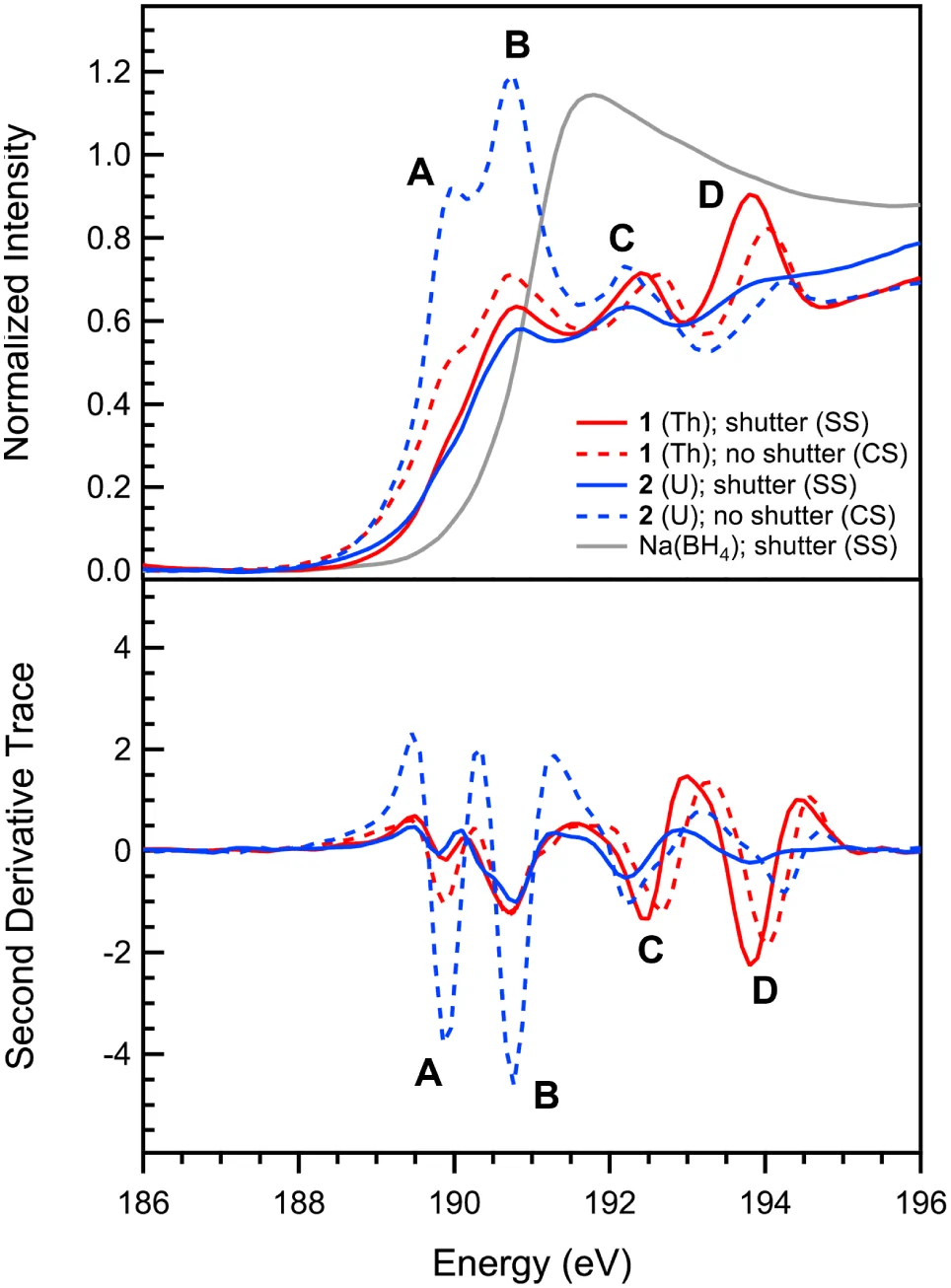
*Top*—B K-edge XAS comparison of An­(κ^3^-BH_4_)­[N­(SiMe_3_)_2_]_3_ with An = Th (**1**; red) and U (**2**; blue) with major features labeled A–D to facilitate discussion. Solid lines indicate step scans (SS) collected at 0.5 s dwell times with a PZ shutter between energy moves. Dashed lines indicate continuous scans (CS) collected at 1.0 s dwell times without a PZ shutter. The B K-edge XAS spectrum of NaBH_4_ is shown in gray for comparison. *Bottom*—Second derivative traces for scans of **1** and **2**.

**Table 2:** Step Scan (SS) and Continuous Scan (CS) Peak Position Energies in eV for 1 (M = Th) and 2 (M = U) from Second Derivative Traces Shown in Figure 3

Peak	**1** (SS)	**1** (CS)	**2** (SS)	**2** (CS)
A	189.9	189.9	189.8	189.9
B	190.7	190.7	190.8	190.8
C	192.5	192.7	192.2	192.2
D	193.8	194.0	194.8	194.3

Interestingly, the same features identified in the step scan data were observed at nearly identical energies when spectra were collected using continuous scans (no PZ shutter). However, the continuous scan and step scan spectra showed dramatic differences in intensity, especially for features A and B (Figure 3; Table 2). More remarkably, the samples did not initially show significant scan-to-scan changes (as would be expected for photodecomposition) when repeat continuous scans were collected on the same spot without moving sample positions (Figures S14–S15). These continuous scan spectra were also reproducible on numerous independently prepared samples collected on separate experimental runs. Only slight differences in peak intensity and resolution were observed (Figure S21).

To ensure that the differences in the spectra collected with and without the PZ shutter were not due to sample-to-sample differences, we also collected repeat continuous scan data on the same samples that yielded the step scan data shown in Figure 3. This was performed by moving to a fresh spot on the sample plate after the step scan data collection was complete. The continuous scan data collected on these new sample positions once again showed the characteristic increase in intensity for features A and B (Figure 4), and they culminated in spectra like those shown in Figure 3 (see Figures S21 and S22 for overlays). Another notable observation is that we were able to capture scan-to-scan spectral changes in these latter experiments by further decreasing the energy range and time required for each continuous scan (Figure 4). This scan-to-scan transformation was also captured in a repeat scan collected on the same spot of **1** using the PZ shutter and step scans (Figure S16). Collectively, these data show that the differences in the step scan and continuous scan spectra shown in Figure 3 can be attributed to photodecomposition associated with different levels of exposure to the beam.

**Figure 4:**
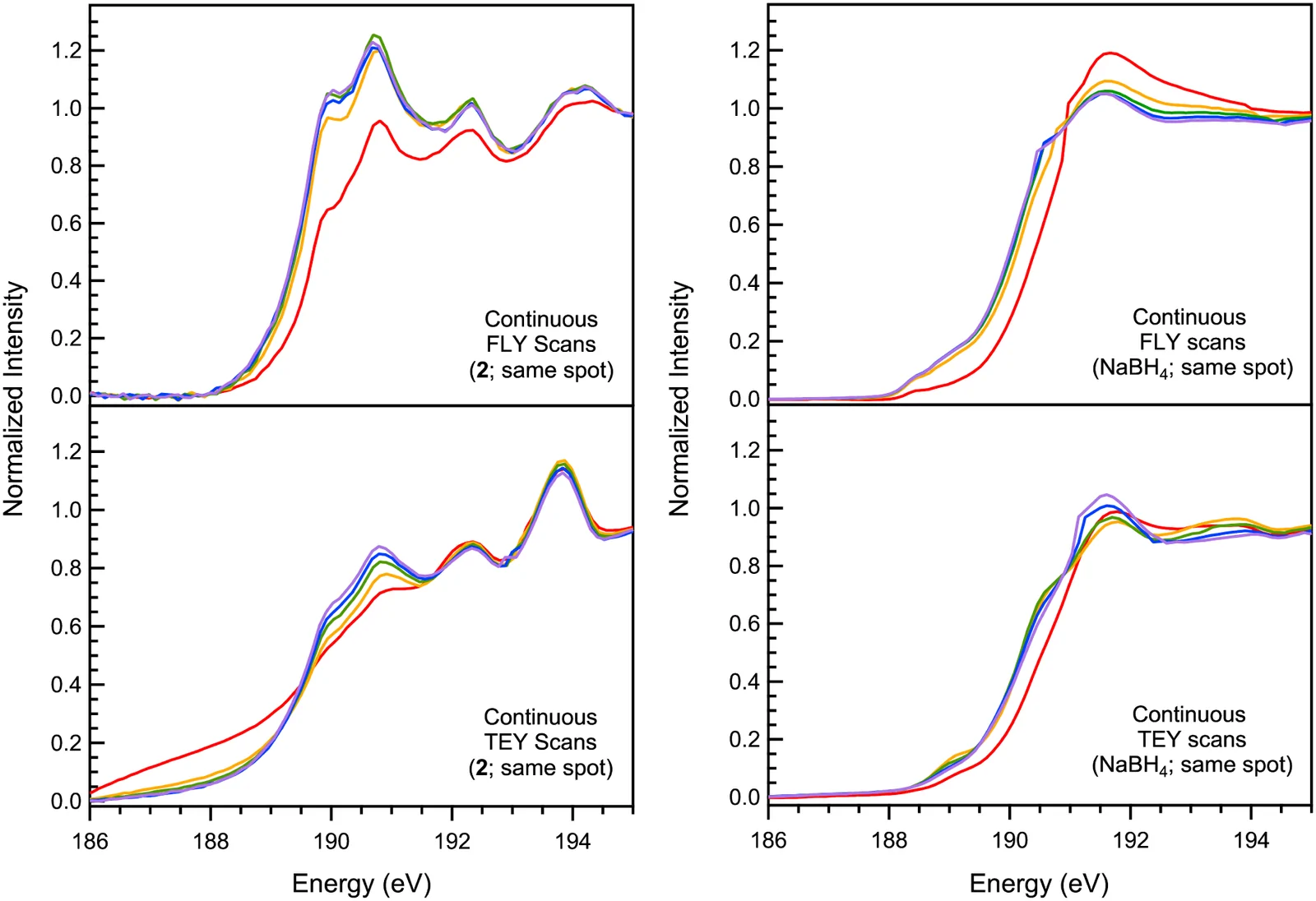
*Left*—Normalized continuous FLY (top) and TEY (bottom) spectra collected repeatedly on the same sample position of **2** with 1.0 s dwell times. The red trace is the first scan, and the purple trace is the final scan. The sample of **2** used to collect these data was the same as that used to collect the step scan FLY data presented in Figure 3 with 0.5 s dwell times. The beam position was moved to a fresh spot between collection of the step scan data shown in Figure 3 and the continuous scan data shown here. *Right*—Normalized continuous FLY (top) and TEY (bottom) spectra collected repeatedly on the same sample position of NaBH_4_ with 1.0 s dwell times. The red trace is the first scan, and the purple trace is the final scan.

To round out our analysis, TEY spectra were collected concurrently with the FLY data to evaluate spectral differences between the sample surface and the bulk (Figures 4, S19 and S20). Generally, the TEY data reproduced the spectral features in the FLY data for **1** and **2**, though the spectra show more hydrolysis on the sample surface, as expected given the high air sensitivity of the samples. For example, the TEY spectra reveal a more intense feature close to 194 eV, which is consistent with transitions associated with B–O bonds.^74^ It is likely that subtle sample-to-sample energy and intensity differences for features C and D in the bulk FLY data are affected by small variations in hydrolysis on the surface. However, we note that features C and D are predicted by TDDFT calculations for **1** and **2** (see below), so they do not appear to be ascribed to surface hydrolysis alone.

Repeat continuous scans collected on the same sample position again revealed a stepwise increase in intensity, as illustrated with the TEY scans of **2** shown in Figure 4. The TEY signal corresponds to collected Auger electrons ejected from the sample (as measured using drain current), so the increasing intensity may suggest an increase in sample conductivity as a function of beam exposure. Consistently, we observed that sample positions exposed to the beam revealed spots that appeared to expose more of the underlying In foil used for conduction (Figure S31).

With these observations, we can elaborate on the origin of the scan-to-scan intensity differences in the B K-edge XAS spectra for **1** and **2**. One hypothesis is that the increasing spectral intensity, especially for the first two features below 191 eV, are assignable to the ingrowth of photodecomposition products as the sample is continuously exposed to the beam. The most compelling evidence in support of this hypothesis is similar data collected for NaBH_4_ subjected to repeat continuous FLY and TEY scans on the same sample spot (Figure 4). These NaBH_4_ data revealed the ingrowth of new broad features below 191 eV. It is clear to see how the ingrowth of similar features in repeat scans on the same positions for **1** and **2** would account for the growing intensity of features A and B, as shown in Figure 4. One unusual observation is that we do not see features associated with the real sample disappear, as observed by us previously, and as one would expect as sample is lost to photodecomposition processes. The features for **1** and **2** only appear to increase in intensity. This observation could suggest that a different process occurs during repeated scanning of **1** and **2**.

Another hypothesis is that samples of **1** and **2** sublime, or photodecompose to form boron-free products that then sublime, when exposed to the beam under UHV conditions. Evidence in support of this hypothesis is the relatively uniform increase in intensity in the FLY data. This behavior is consistent with decreasing self-absorption (also called over-absorption)^75,76^ as the sample concentration in the path of the beam decreases with loss of material. This hypothesis is also consistent with increasing TEY signal (and sample conductivity), and the visible spots that appear in the sample when scanning repeatedly on the position. Moreover, any B–O containing products that form due to hydrolysis would likely be less volatile (these are often oligomeric with B–O–B linkages), and boron oxides are more robust with respect to photodecomposition in the beam. This may account for why peaks occurring in the region where hydrolysis features are expected (features C and D; Figure 3 and Table 2), do not appear to change significantly in intensity during repeated scanning on the same position (Figure 4).

Most importantly, this hypothesis is consistent with the known chemistry of U^IV^ tetrahydroborates, which are known to undergo both photo and thermal reduction to U^III^ complexes with net loss of H_2_ and B_2_H_6_.^77,78^ Similar reactivity occurs with **2**.^79^ In particular, this complex thermally decomposes at 70 °C in C_6_D_6_ to form the U^IV^ metallocycle U^IV^[N­(SiMe_2_CH_2_)­R]­(NR_2_)_2_ (**3**; R = SiMe_3_), a process which is presumed to proceed through the hydride intermediate UH­(NR_2_)_3_ (Scheme 1a). The Th analogue Th­[N­(SiMe_2_CH_2_)­R]­(NR_2_)_2_ (**4**; R = SiMe_3_) has similarly been isolated and shown to undergo reactions with H_2_ to form ThH­(NR_2_)_3_, which is unstable with respect to **4** (Scheme 1b). Both **3** and **4** have appreciable volatility, as is typically observed for f-element complexes with [N­(SiMe_3_)_2_]^1–^ ligands. Andersen and coworkers reported that **3** and **4** sublime at 100–110 °C under vacuum (pressure not reported),^80^ suggesting that samples could sublime when heated by the beam under UHV conditions (which is much higher vacuum than is typically used in synthetic preparations and lab-scale sublimations). Any non-boron containing species like **3** and **4** generated during analysis of **1** and **2** would not be observable by B K-edge XAS, so the spectra obtained would be attributed almost exclusively to these BH_4_-containing samples. This could explain why the energies of the first two features do not change appreciably over repeat scanning despite the changes in intensity.

**Scheme 1:**
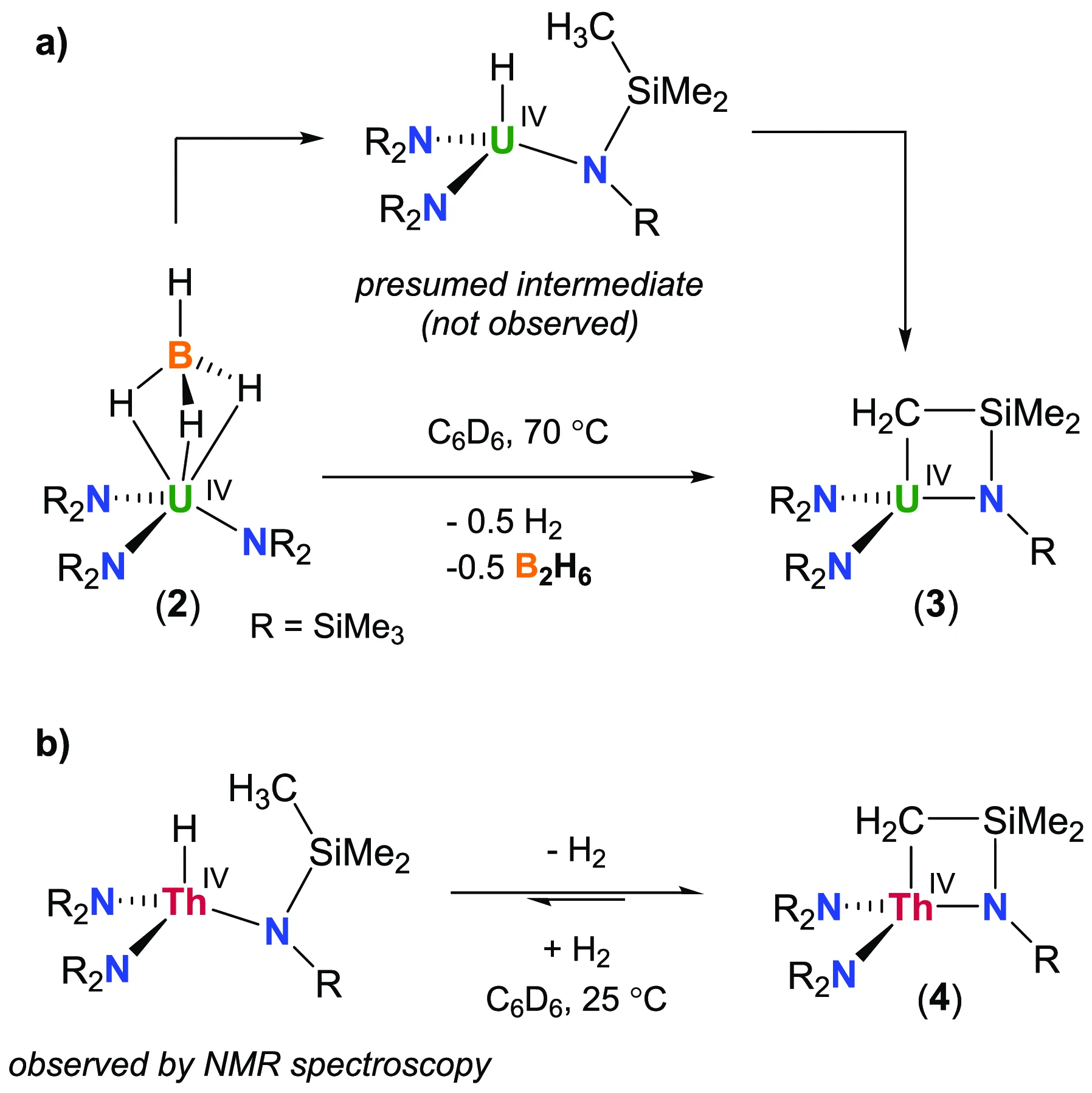
a) Solution Thermolysis of 2 to Form the Metallocycle 3;^79^ b) Equilibrium Reactivity of 4 with H_2_ Demonstrating the Instability of the Hydride ThH­(NR_2_)_3_ (R = SiMe_3_)^79^

To summarize, the B K-edge XAS data strongly suggest that **1** and **2** undergo beam-induced photodecomposition, but the associated spectral changes deviate from those typically observed for photodecomposition in other B K-edge studies. Two hypotheses were presented to address the underlying origin of these differences, including one that accounts for the potential identity of the photodecomposition products. Unfortunately, it is difficult to rule out either hypothesis completely with the data at hand, and it is possible that both contribute at some level to the spectra collected after repeated beam exposure. However, it seems the first hypothesis is the most likely. The increasing intensity of features A and B as **1** and **2** are subjected to repeated scanning mirror the appearance of new pre-edge features when NaBH_4_ was analyzed under identical conditions. The identity of the boron-containing material that is formed is not known, but generation of similar photodecomposition products in scans of **1** and **2** would account for the intensity increase in features A and B after extended beam exposure.

Despite the appearance of beam-induced photodecomposition at longer scanning times, it appears we have sufficiently minimized photodecomposition with the step scans and PZ shutter so that reliable comparisons can be made between the two samples. The step scan data reveal spectra for **1** and **2** that are similar in profile and peak positions, which would not be expected if there were substantial 5f contributions to the metal-borohydride bonding. It is well known that 5f orbitals drop in energy as the actinide series is traversed from Th to U, whereas the 6d orbitals energies do not. This has been illustrated in previous ligand K-edge XAS studies of isomorphous complexes containing different actinides in the +4 oxidation state.^21,81^ The similarities in the B K-edge XAS spectra for **1** and **2** therefore suggest that any covalent metal-borohydride bonding is dominated by An 6d contributions. This hypothesis is indeed corroborated by supporting calculations described in the following section.

### DFT and TDDFT Calculations

To evaluate the underlying electronic structure of **1** and **2** and assign the B K-edge XAS features, density functional theory (DFT) and time-dependent density functional theory (TDDFT) computations were performed. The ground state geometries of **1** and **2** were first optimized using DFT, and 14 different functionals were tested (see SI for Computational Details). In general, the calculated DFT geometries are in good agreement with the experimental structures with most exhibiting only minor functional dependence (Figure S32).

Before comparing the TDDFT results, it is instructive to first discuss the orbitals available for B K-edge XAS transitions in the ground states of **1** and **2** (Figure 5). The calculations reveal that the lowest unoccupied molecular orbitals (MOs) are relatively nonbonding 5f with B 2p contributions of 1.4% or less (Tables S12–S13). Among the 5f orbitals for **1** is the one assigned with the 1a Mulliken symbol shown in Figure 5. Though group theory predicts the possibility of “through-space” An-B σ and σ* MOs that could arise from mixing between the actinide 5f_z3_ and the B 2p_*z*_, the actual calculations reveal that the 1a MO does not have significant B 2p character (Figure 5; right). However, we note its presence because it is sometimes invoked in discussions of metal-borohydride bonding.^82^ The small amount of orbital mixing in the remaining 5f-derived MOs for **1** and **2** appear to occur through the An–H–B linkages (Tables S12–S13). Among those for **1** is MO 5a (Figure 5). Though it contains negligible B 2p character (0.2%), it is worth noting because it exhibits a small amount of M-H mixing with Th (47.8% 5f; 11.2% 6d; 2.7% H 1s). A similar MO is observed for **2** with U (4.4% H 1s; assigned as 3a in Figure 5), though this MO contains significantly less 5f character and more 6d mixing (3.0% 5f; 29.1% 6d) due to the decrease in U 5f orbital energies compared to those with Th.

**Figure 5:**
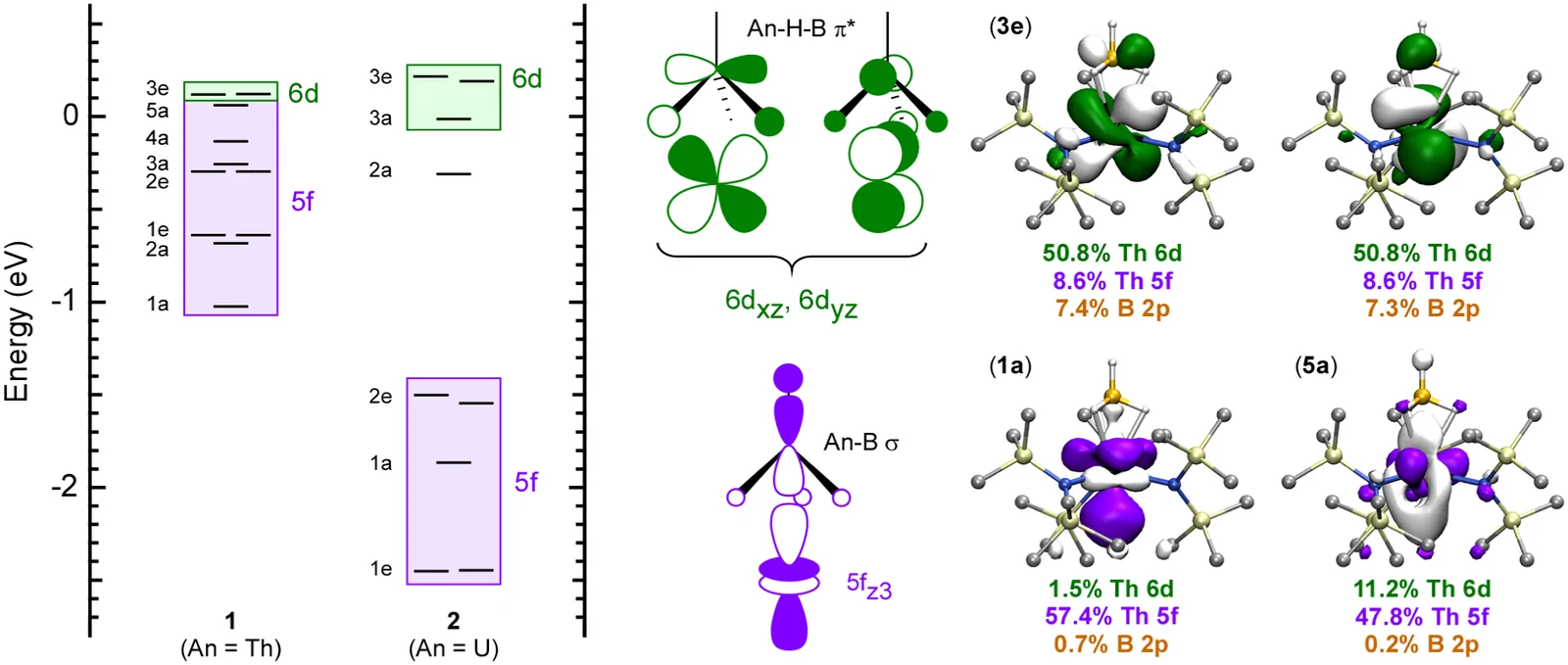
*Left*—energies of select unoccupied An 5f- (purple) and 6d-derived MOs (green) calculated for An­(κ^3^-BH_4_)­[N­(SiMe_3_)_2_]_3_ with An = Th (**1**) and U (**2**) using the PBE functional. Mulliken symbols of the orbitals in C_3_ point group symmetry are provided. MO 2a for **2** is not color coded because it contains less than 3% U 5f and 6d character (Table S13). *Right*—qualitative depiction of An–H–B π* MOs (6d*_xz_* and 6d*_yz_*) and An–H–B σ MO (5f_z3_) predicted by group theory and comparison to actual MOs and percent B 2p and An 5f and 6d character calculated for **1** with DFT. Composition of 3e MOs for **2** for comparison (U 6d/U 5f/B 2p): 56.4%/0.9%/6.5% and 56.0%/1.0%/6.6%. Additional information on MO compositions and shapes is provided in Tables S12–S13 in the SI.

The most important MOs with respect to the B K-edge XAS studies are two degenerate 6d-containing MOs of e symmetry (3e) that appear at higher energy relative to the MOs of 5f parentage. These can be described either as An ← H–B σ* or An–H–B π* based on the relative arrangement of the M 6d and B 2p orbitals (Figure 5; right). We use the latter An–H–B π* description based on previous precedent^83,84^ and to better distinguish the relative arrangement of the B 2p orbitals. These 6d-derived 3e MOs have significantly more B 2p mixing compared to the MOs of 5f parentage: B_2p_ = 7.4%/7.3% for **1** (14.7% total) and B_2p_ = 6.5%/6.6% to **2** (13.1% total). The 3e MOs for **1** exhibit some 5*f*/6d hybridization (Th_6*d*/5f_ = 50.8%/8.6% each; Figure 5), but this decreases in **2** as the 5f orbitals drop in energy (U_6*d*/5f_ = 56.4%/0.9% and 56.0%/1.0%). As is typically observed when traversing the actinide series, the 5f orbitals drop significantly in energy from Th to U, whereas the 6d orbitals are less perturbed. We note that the calculations indicate that the An–H–B π* MOs in **1** shift to higher energy by 0.05–0.07 eV for **2** using the PBE functional (the same trend is also observed with B3LYP-D3). The difference in energy is consistent with the 0.1 eV shift of feature B in the experimental spectra (190.7 eV in **1** to 190.8 eV in **2**).

TDDFT calculations were performed to simulate the B K-edge XAS spectra for **1** and **2**. The simulated spectra were calculated using the TPSSh-D4 and B3LYP-D4 functionals.^85,86^ Three different optimized ground state geometries were also tested (PBE, B3LYP, and B3LYP-D3)^87–89^ to evaluate functional and structure dependence on the simulated spectra. All the TDDFT predicted spectra show the same trends and provide generally good agreement with experiment (Figure 6 and Figures S33–S35; SI).

**Figure 6:**
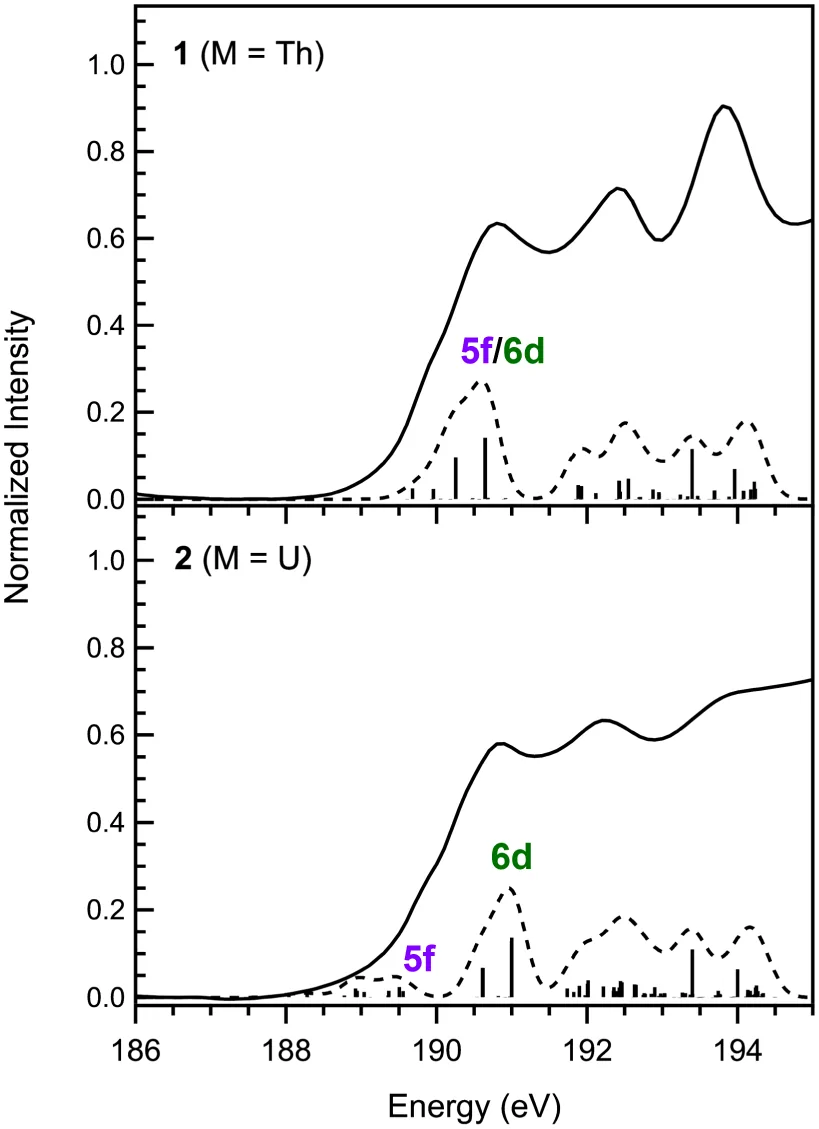
Comparison of experimental and TDDFT spectra of **1** (top) and **2** (bottom) computed at the B3LYP-D4 level of theory on the PBE optimized geometry. The experimental spectra are the step scan FLY data shown in Figure 3. Computed spectra (dotted line) are shifted by 9.3 eV and the broadening employs a full width at half maximum (FWHM) of 0.45 eV. The computed spectra and individual transitions (black bars) are magnified to bring them on scale with the experimental data.

Based on orbital decomposition analysis of the underlying transitions from TDDFT (Figure S36 and Tables S10–S11; SI), the first two features in the B K-edge XAS spectra of **1** and **2** (labeled A and B in Figure 3 and Table 2) are assigned as primarily B 1s → An–H–B π* transitions that are modulated slightly by differences in 6d and 5f energies and hybridization. Though small transitions involving the 5f MOs are observed at lower energy in the calculated spectra, these are not readily apparent in the experimental data, especially for **2**. This is consistent with their relatively non-bonding nature and the minimal B 2p character in the associated MOs. Two more intense transitions appear at higher energy that are nearly identical for **1** and **2**. These transitions involve the 3*e* MOs of 6d parentage shown in Figure 5. The relative similarities in peak positioning are consistent with the expected and calculated similarities in the An–H–B π* MOs for **1** and **2**. The energy of the B features at 190.7/190.8 eV are similar to those previously assigned as B 1s → Hf–H–B π* MOs (5d parentage) for Hf­(RBH_3_)_4_.^66^ Note that the same assignment arises from analyzing the natural transition orbitals (NTOs) (Table S14–S15). Rounding out the analysis, the higher energy features labeled C and D in Figure 3 are primarily transitions into diffuse, non-bonding virtual orbitals. As noted above, these latter features, especially feature D, are in the same region as where hydrolysis features appear.

The TDDFT calculations suggest that the B K-edge XAS spectra of **1** and **2** are dominated by B 1s → An–H–B π* transitions involving 3e MOs of primarily 6d parentage, though we note the 5f hybridization in **1** (Figure 5) also contributes to the Th–H–B π* bonding. This is relevant when considering covalency-induced measurement of NMR shifts.

### Calculated NMR Shifts

The presence of NMR active nuclei in (BH_4_)^1–^ provided a rare opportunity to compare covalency assessments made using B K-edge XAS to those obtained using ^1^H and ^11^B NMR spectroscopy with diamagnetic **1**. To account for deshielding in the hydride and boron resonance of **1**, NMR shifts and chemical bonding were calculated with DFT using the scalar relativistic (SR) and spin-orbit (SO) all electron ZORA Hamiltonian.^29–32^ Based on literature precedent, the PBE0 functional was employed (PBE results were similar; Table S14). The ^1^H and ^11^B chemical shifts were computed both with (δ_SO_) and without (δ_SR_) the use of spin orbit (SO) effects, using SiMe_4_ (^1^H) and BF_3_·Et_2_O (^11^B) as references (Table 3).

**Table 3:** Experimental (δ_exp_) and Computed (PBE0) NMR Chemical Shifts (ppm) of 1 Using Both Scalar Relativistic Computations (δ_SR_) and Computations That Include Corrections for Spin-Orbit Coupling (δ_SO_)­^a^

	δ_exp_	δ_SO_	δ_SR_	Δ_SO_
^1^H_terminal_	-	5.68	1.92	3.76
^1^H_bridging_	-	3.55	1.70	1.86
^1^H_avg_	4.25	4.09	1.75	2.33
^11^B	–8.0	–13.68	–21.68	7.72

a Δ_SO_ = δ_SO_ – δ_SR_.

Calculations performed on the DFT optimized structure of **1** yield distinct δ_SO_ and δ_SR_ values for the terminal (^1^H_terminal_) and bridging (^1^H_bridging_) hydride positions. Averaging these values yields a calculated hydride shift (^1^H_avg_) of δ_H_ = 4.09 ppm, which is in excellent agreement with the experimental shift in C_6_D_6_ at δ_H_ = 4.25 ppm. The calculated average *J*_BH_ (90 Hz) also closely matches the experimental value (83 Hz). The deshielding induced by SO effects (Δ_SO_) contributes 2.33 ppm to the downfield shift. While small, this value points to a modest Th–H covalency, in line with natural localized molecular orbital (NLMO) analysis (see below). Curiously, Δ_SO_ for ^1^H_terminal_ (δ 3.76 ppm) is larger than those observed for ^1^H_bridging_ (δ 1.86 ppm), despite its substantially longer distance from the Th center, and negligible orbital overlap (see below). We hypothesize that the aforementioned “through-space” An-B σ* interaction is responsible for this effect; however, understanding its exact origin will require further work.

The calculated ^11^B shift (δ_B_ = −13.68 ppm) is in acceptable agreement with experiment (δ_B_ = −8.0 ppm), considering the large errors often found for calculated ^11^B chemical shifts.^90^ Moreover, the ^11^B shift showed a Δ_SO_ of 7.72 ppm, suggesting some degree of orbital mixing between Th and B, consistent with the results of the B K-edge XAS study. The ^1^H and ^11^B chemical shifts of the Zr­(PhBH_3_)_4_ and Hf­(PhBH_3_)_4_ complexes previously reported by Hansen et al.^66^ were additionally computed (Table S17). The Δ_SO_ values were noted to increase from Zr to Hf in ^1^H (0.26, 0.83 ppm) and ^11^B (1.25, 3.03 ppm) calculations, as expected given the increase in SO effects on going down the group. For further comparison, a recently reported thallium κ^2^-borohydride complex exhibited a negligible Δ_SO_ of 0.2 ppm for its ^11^B chemical shift, due to the highly ionic Tl-borohydride interaction.^47^

While the B K-edge XAS measurements emphasize orbital mixing in the antibonding orbitals, the orbitals of interest in the comparison of DFT with NMR data focuses on the occupied orbitals (in this case the NLMOs). Moreover, the NMR shielding tensors can be decomposed in terms of the NLMOs.^91,92^ Specifically, the NLMOs calculated for **1** highlight the orbital interactions between the borohydride and Th responsible for the spin-orbit shifts (Figures S37–S38; SI).^93^ The three bridging hydrides (H_bridging_) each show a σ-type interaction along the B–H_bridging_ bond, which weakly donates via the H_bridging_ into empty orbitals on Th to yield the three-center, two-electron Th–H_bridging_-B bonds. On average, these three NLMOs have atomic contributions with 6.7% from thorium, 42.0% from boron, and 50.7% from hydrogen (Tables S23–S24; SI). Of the thorium contribution, 55% is from 6d orbitals, while 39% is from 5f orbitals. While the thorium contribution to the three NLMOs is somewhat smaller than that observed for a typical Th–C bond,^24^ in line with the small Δ_SO_ value, the 6*d*/5f ratio is comparable.^36^ As expected, the NLMO corresponding to the terminal hydride (H_terminal_) shows a very small Th weight of only 0.5% (Tables S23–S24; SI).

### QTAIM Calculations

As a final assessment of metal–ligand (M-L) bonding in **1** and **2**, quantum theory of atoms in molecules (QTAIM)^94–96^ was used to obtain both the average electron density (ρ) at bond critical points and the delocalization indexes (δ_DI_) between metal and hydrogen atoms (Table 4). Covalency in M-L bonds can be influenced by changes in either orbital overlap or frontier orbital energy matching.^8,21^ QTAIM has been used to identify these contributions. The ρ values have been used by us and others as a reflection of overlap-driven covalency, whereas the δ_DI_ values can be used as a metric for energy-degeneracy-driven covalency.^62,63,97,98^ Moreover, this analysis has been used in prior studies by some of us to assess the covalent contributions to the M-L bonding in actinide­(III) and lanthanide­(III) borohydrides.^62,63^

**Table 4:** QTAIM Parameters and Nalewajski–Mrozek Type 3 Bond Orders

	Distance (Å)	Bond Order	ρ^a^	δ_DI_
Th–B	2.625	0.466	0.046	0.151
Th–H_bridging_	2.423	0.146	–	0.198
U–B	2.541	0.748	0.056	0.185
U–H_bridging_	2.324	0.242	–	0.242

a No An-H BCPs were identified by QTAIM analysis.

The QTAIM calculations indicate a larger accumulation of electron density in the An-B critical point in **2** (ρ = 0.056) compared to **1** (ρ = 0.046), supporting the assignment of increased orbital overlap in **2** (Tables S4–S5). The delocalization indexes for the An-H_bridging_ and An-B (Tables 4 and S9) likewise indicate more favorable orbital energy matching in **2**. These results are consistent with prior work showing a similar trend across early actinide borohydride complexes.^63^ Finally, energy decomposition analysis (EDA) examined the interaction between the anionic BH_4_ ligand and the remainder of the molecule. The results, like the XAS and NMR spectra, show a subtle increase in the orbital interactions in **2**, despite having the same interaction energy (Tables S6–S7). Subsequently, the orbital contribution to the bonding can be further decomposed via extended transition state-natural orbital for chemical valence (ETS-NOCV) analyses. These results show three larger orbital interactions consisting of the “through-space” interaction and two sets of orbital contributions via the bridging hydrogen groups (Table S8).

## Concluding Remarks

In summary, we have described the first investigation of actinide-borohydride bonding using ligand K-edge XAS and NMR spectroscopy. The B K-edge XAS spectra of An­(κ^3^-BH_4_)­(NR_2_)_3_ with An = Th (**1**) and U (**2**) and R = SiMe_3_ suggest appreciable An–H–B π covalency involving the actinide 6d-orbitals with substantially smaller contributions from the 5f-orbitals. The NMR results for **1** corroborate these results by revealing chemical shifts attributed to small but measurable M-L covalency involving the 6d and, to a lesser extent, 5f orbitals. These results also represent the first instance of using ^11^B NMR spectroscopy and B K-edge XAS to measure covalency in the actinides. Because B K-edge XAS has not been used previously for evaluating metal-ligand covalency in f-element borohydride complexes, the NMR studies provide independent corroboration that demonstrates the viability of using boron as an XAS reporter of three-center, two-electron An–H–B bonding.

The experimental results are tightly connected by two computationally driven analyses: NLMOs that provide NMR shielding constants in **1** and NTOs and TDDFT calculations that corroborate the B K-edge XAS results for **1** and **2**. The relatively smaller downfield chemical shift in **1** is a direct result of a small δ_SO_ and ultimately the small participation of Th 5f orbitals. This is verified by XAS measurements and the subsequent DFT and TDDFT results that indicate predominate 6d character in the bonding. The results demonstrate how these complementary spectroscopies can be combined with connecting theoretical methods to establish a holistic assessment of 5f and 6d orbital mixing in actinide-ligand bonds. These findings open the door to similar comparative covalency studies on the many complexes containing actinide-ligand bonds (e.g., An-C, An-F, An-Si, An-P) that are accessible using both ligand K-edge XAS and NMR spectroscopy.

## Supplementary Material



## Data Availability

Input and output files and xyz structures available: https://doi.org/10.6084/m9.figshare.31242301.

## Notes

Experimental and computational details; NMR spectra and X-ray crystallographic data for **1** and **2**; full B K-edge spectra and second-derivative traces (PDF)
