# Supplementary material for: Holistic Assessment of Actinide-Borohydride Covalency Using NMR and X‑ray Absorption Spectroscopies
Source: J Am Chem Soc. 2026 Sep 22;148(39):41786–97. doi: 10.1021/jacs.6c02674 (PMC13651512; doi:10.1021/jacs.6c02674)
Supplement: Supplementary file 1 [file ja6c02674_si_001.pdf]

SUPPORTING INFORMATION

Holistic Assessment of Actinide-Borohydride Covalency using NMR and X-ray Absorption Spectroscopies

Hannah M. Hansen,[1‡] Nathan R. Loutsch,[1‡] Thien H. Nguyen,[2] Aaron Christianson,[1] Jacob J. Schuely,[1] Mai Yer Yang,[1] Peter A. Zacher III,[1] Lucia Zuin,[3] Trevor W. Hayton,[2*] Bess Vlaisavljevich,[1*] Scott R. Daly[1*]

[1]*Department of Chemistry, The University of Iowa, E331 Chemistry Building, Iowa City, Iowa 52242, United States*

[2]*Department of Chemistry and Biochemistry, University of California Santa Barbara, Santa Barbara, California 93106, United States*

[3]*Canadian Light Source, University of Saskatchewan, 44 Innovation Place, Saskatoon, SK, S7N 2V3, Canada*

[‡]These authors contributed equally.

Corresponding author emails: scott-daly@uiowa.edu, bess-vlaisavljevich@uiowa.edu, hayton@chem.ucsb.edu

Table of Contents

Experimental 2

NMR Spectra 6

B K-edge XAS 16

Computational Details 29

  Functional Testing 30

  Geometry and Bonding 32

  Time-Dependent Density Functional Theory K-Edge 36

  Nuclear Magnetic Resonance 91

  XYZ Coordinates 96

Supporting Information References 101

Experimental

General. All reactions and subsequent manipulations were performed under anaerobic and anhydrous conditions under an atmosphere of nitrogen. Et_2O was dried using a Vacuum Atmospheres DRI-SOLV Solvent Purification system and stored over 3 Å sieves for 24 h prior to use. Pentane was dried using a Pure Process Technology, LLC Glass Contour Solvent system and stored over 3 Å sieves for 24 h prior to use. Tetrahydrofuran (THF) was distilled over Na/benzophenone and stored over activated 3 Å molecular sieves for 24 h prior to use. Benzene-d_6 was dried over 3 Å molecular sieves for 24 h prior to use. UCl_4, [$ThCl_4$(DME)$_2$], and [AnCl(NR_2)$_3$] (An = Th, U; R = $SiMe_3$) were synthesized according to the previously reported procedures.[1-4] All other reagents were purchased from commercial suppliers and used as received.

NMR spectra were recorded on a Bruker Advance III HD 400 MHz spectrometer. ^{1}H NMR spectra were referenced to external tetramethylsilane (TMS) using the residual protio solvent peaks as internal standards. ^{11}B NMR spectra were referenced indirectly with the ^{1}H resonance of TMS at 0.0 ppm, according to IUPAC standard, using the residual solvent peak as an internal standard.

Synthesis of [Th(NR_2)$_3$(BH_4)] (1). [Th(NR_2)$_3$(BH_4)] (where R = $SiMe_3$) was synthesized following the previously reported procedure.[1] A $LiBH_4$ (1.5 mg, 0.068 mmol) solution in THF (2 mL) was added to [ThCl(NR_2)$_3$] (50.7 mg, 0.068 mmol) in THF (4 mL) at -25 °C. The mixture was stirred for 20 h at room temperature. The volatiles were then removed *in vacuo* to yield a white solid. The white solid was triturated with pentane (1 mL), dissolved in pentane (4 mL), and filtered through a Celite column supported on glass wool (0.5 × 2 cm). The filtrate was stored at -25 °C for 12 h, which resulted in the deposition of white needles. The needles were isolated by decanting the supernatant and dried *in vacuo* to afford **1**. ^{1}H and ^{11}B NMR spectra of the needles were recorded, which were consistent with the previously reported spectra.[1] Yield: 10.0 mg, 20% yield. ^{1}H NMR (400 MHz, 25 °C, C_6D_6): δ 4.25 (q, 4H, BH, J_{BH} = 83.4 Hz), 0.38 (s, 54H, $SiMe_3$). ^{11}B NMR (128.4 MHz, 25 °C, C_6D_6): δ -8.00 (p, J_{BH} = 84.9 Hz).

Synthesis of [U(NR_2)$_3$(BH_4)] (2). [U(NR_2)$_3$(BH_4)] (where R = $SiMe_3$) was synthesized following the previously reported procedure.[1] A $LiBH_4$ (3.0 mg, 0.138 mmol) suspension in diethyl ether (2 mL) was added to [UCl(NR_2)$_3$] (54.9 mg, 0.073 mmol) in diethyl ether (4 mL) at -25 °C. The mixture was stirred for 20 h at room temperature, which resulted in a color change from pale purple to deep yellow reaction mixture. The volatiles were removed *in vacuo* to yield a brown solid. The brown solid was triturated with pentane (1 mL), dissolved in pentane (4 mL), and filtered through a Celite column supported on glass wool (0.5 × 2 cm). The filtrate was stored at -25 °C for 12 h, which resulted in the deposition of brown needles. The needles were isolated by decanting the supernatant and dried *in vacuo* to afford **2**. Yield:

17.2 mg, 32% yield. ^{1}H and ^{11}B NMR spectra of the needles were recorded, which were consistent with the previously reported spectra.[1] ^{1}H NMR (400 MHz, 25 °C, C_6D_6): δ -2.05 (s, 54H, $SiMe_3$), -99.95 (q, 4H, BH, J_{BH} = 78.5 Hz). ^{11}B NMR (128.4 MHz, 25 °C, C_6D_6): δ -10.00 (br s).

Synthesis of [Th(NR$_2$)$_3$(BD$_4$)] (1-*d*$_4$). A $LiBD_4$[5] (2.7 mg, 0.105 mmol) solution in Et_2O (2 mL) was added to [ThCl(NR$_2$)$_3$] (60.2 mg, 0.080 mmol) in Et_2O (4 mL) at -25 °C. The mixture was stirred for 24 h at room temperature. The volatiles were then removed *in vacuo* to yield a white solid. The white solid was triturated with pentane (1 mL), dissolved in pentane (3 mL), and filtered through a Celite column supported on glass wool (0.5 × 2 cm). The filtrate was stored at -25 °C overnight, resulting in the deposition of white needles. The needles were isolated by decanting the supernatant and dried *in vacuo* to afford **1-*d*$_4$**. Yield: 17.0 mg, 28.9% yield. $^{1}H\{^{11}B\}$ NMR (500 MHz, 25 °C, C_6D_6): δ 0.38 (s, 54H, $SiMe_3$). $^{2}H\{^{11}B\}$ NMR (77 MHz, 25 °C, C_6D_6): δ 4.25 (s, BD_4). $^{2}H\{^{11}B\}$ NMR (77 MHz, 25 °C, CH_2Cl_2): δ 3.66 (s, BD_4). $^{2}H\{^{11}B\}$ NMR (77 MHz, -40 °C, CH_2Cl_2): δ 3.56 (s, BD_4). $^{11}B\{^{1}H\}$ NMR (160.5 MHz, 25 °C, C_6D_6): δ -8.42 (s).

Synthesis of [U(NR$_2$)$_3$(BD$_4$)] (2-*d*$_4$). A $LiBD_4$ (6.7 mg, 0.260 mmol) solution in THF (1 mL) was added to [UCl(NR$_2$)$_3$] (145.5 mg, 0.193 mmol) in THF (3 mL) at -30 °C. The mixture was stirred for 24 h at room temperature, resulting in a color change from a pale purple to yellow. The volatiles were then removed *in vacuo* to yield a yellow-brown solid. The solid was triturated with pentane (1 mL), extracted into pentane (3 mL), and filtered through a Celite column supported on glass wool (0.5 × 2 cm). The filtrate was stored at -30 °C overnight, resulting in the deposition of brown needles. The needles were isolated by decanting the supernatant and dried *in vacuo* to afford **2-*d*$_4$**. Yield: 45.8 mg, 32.2% yield. $^{1}H\{^{11}B\}$ NMR (500 MHz, 25 °C, C_6D_6): δ -2.06 (s, 54H, $SiMe_3$). $^{2}H\{^{11}B\}$ NMR (77 MHz, 25 °C, CH_2Cl_2): δ -104.31 (br s). $^{2}H\{^{11}B\}$ NMR (77 MHz, 0 °C, CH_2Cl_2): δ -116.57 (br s). $^{2}H\{^{11}B\}$ NMR (77 MHz, -20 °C, CH_2Cl_2): δ -128.00 (br s). $^{11}B\{^{1}H\}$ NMR (128 MHz, 25 °C, C_6D_6): δ -7.15 (s).

X-ray Crystallography. Data for **1** and **2** were collected on a Bruker KAPPA APEX II diffractometer equipped with an APEX II CCD detector using a TRIUMPH monochromator with a Mo Kα X-ray source (α = 0.71073 Å). The crystals were mounted on a cryoloop under NVH oil. Complexes **1** and **2** were collected at 110(2) K using an Oxford nitrogen gas cryostream. Data were collected using ω scans with 0.5° frame widths. Frame exposures of 5 and 10 s were used for **1** and **2**, respectively. Data collection and cell parameter determinations were conducted using the SMART program.[6] Integration of the data frames and final cell parameter refinements were performed using SAINT software.[7] Absorption corrections of the data were carried out using the multi-scan method SADABS.[8] Subsequent calculations were carried out using SHELXTL.[9] Structure determination was done using direct or Patterson methods and difference Fourier techniques. All hydrogen

atom positions were idealized, and rode on the atom of attachment. Structure solution, refinement, graphics, and creation of publication materials were performed using SHELXTL.[9]

For both complexes **1** and **2**, the terminal B-H distances were fixed to 1.19 Å and the bridging B-H distances were fixed to 1.33 Å using the DFIX command.

Table S1. X-ray Crystallographic Data for **1** (M = Th) and **2** (M = U).

	**1**	**2**
empirical formula	$C_{18}H_{58}B_1N_3Si_6Th_1$	$C_{18}H_{58}B_1N_3Si_6U_1$
crystal habit, color	needle, colorless	needle, brown
crystal size (mm)	0.25 × 0.1 × 0.1	0.25 × 0.1 × 0.1
space group	R3c	R3c
volume (Å^3)	4992.9(14)	4925.6(9)
a (Å)	18.382(2)	18.2991(15)
b (Å)	18.382(2)	18.2991(15)
c (Å)	17.062(2)	16.9852(17)
α (deg)	90	90
β (deg)	90	90
γ (deg)	120	120
Z	6	6
formula weight (g/mol)	728.06	734.05
density (calculated) (Mg/m^3)	1.453	1.485
absorption coefficient (mm^{-1})	4.707	5.173
F_{000}	2196	2208
total no. reflections	11993	11690
unique reflections	3044	2712
Final R Indices [I > 2σ(I)]	R1 = 0.0198 wR2 = 0.0441	R1 = 0.0266 wR2 = 0.0410
largest diff. peak and hole (e$^-$ A^{-3})	0.368 and -0.913	0.699 and -0.543
GOF	1.041	1.203

NMR Spectra

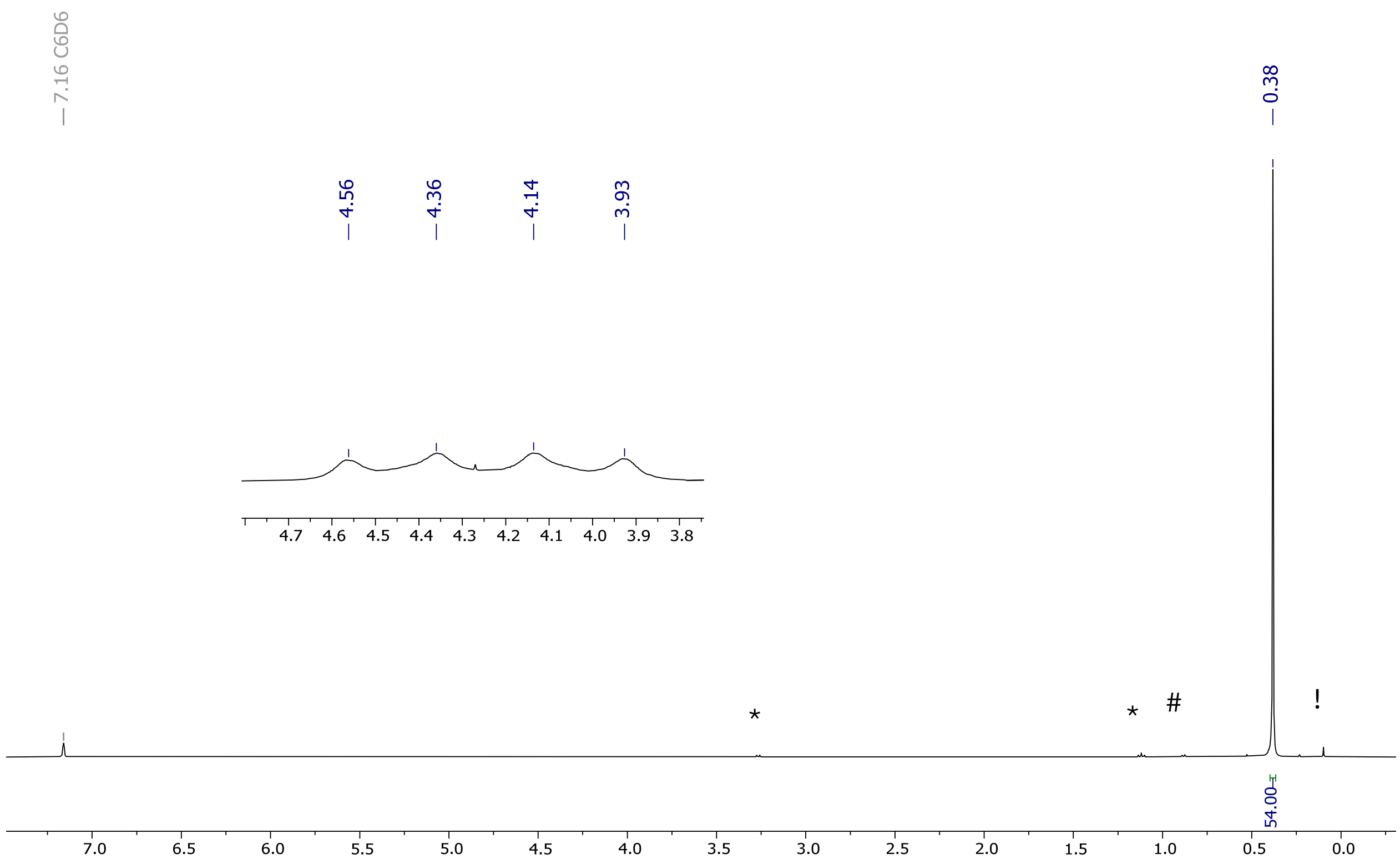

Figure S1. ^{1}H NMR spectrum of **1** (M = Th), recorded in benzene-d_6. (*) indicates resonances assignable to diethyl ether. (#) indicates resonances assignable to pentane. (!) indicates resonances assignable to HMDS. Inset: expansion of the BH_4 resonance.

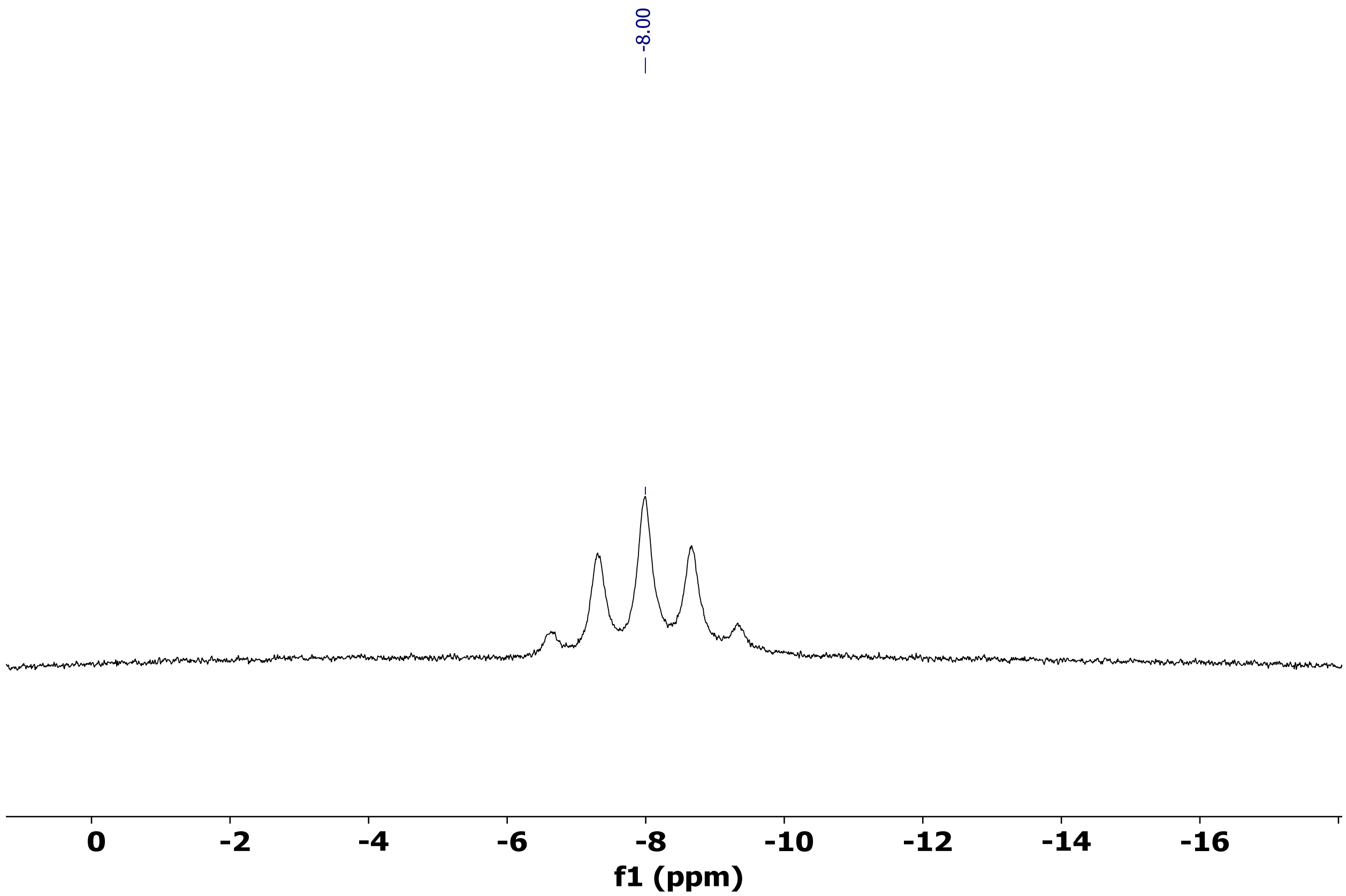

Figure S2. ^{11}B NMR spectrum of **1** (M = Th), recorded in benzene-d_6.

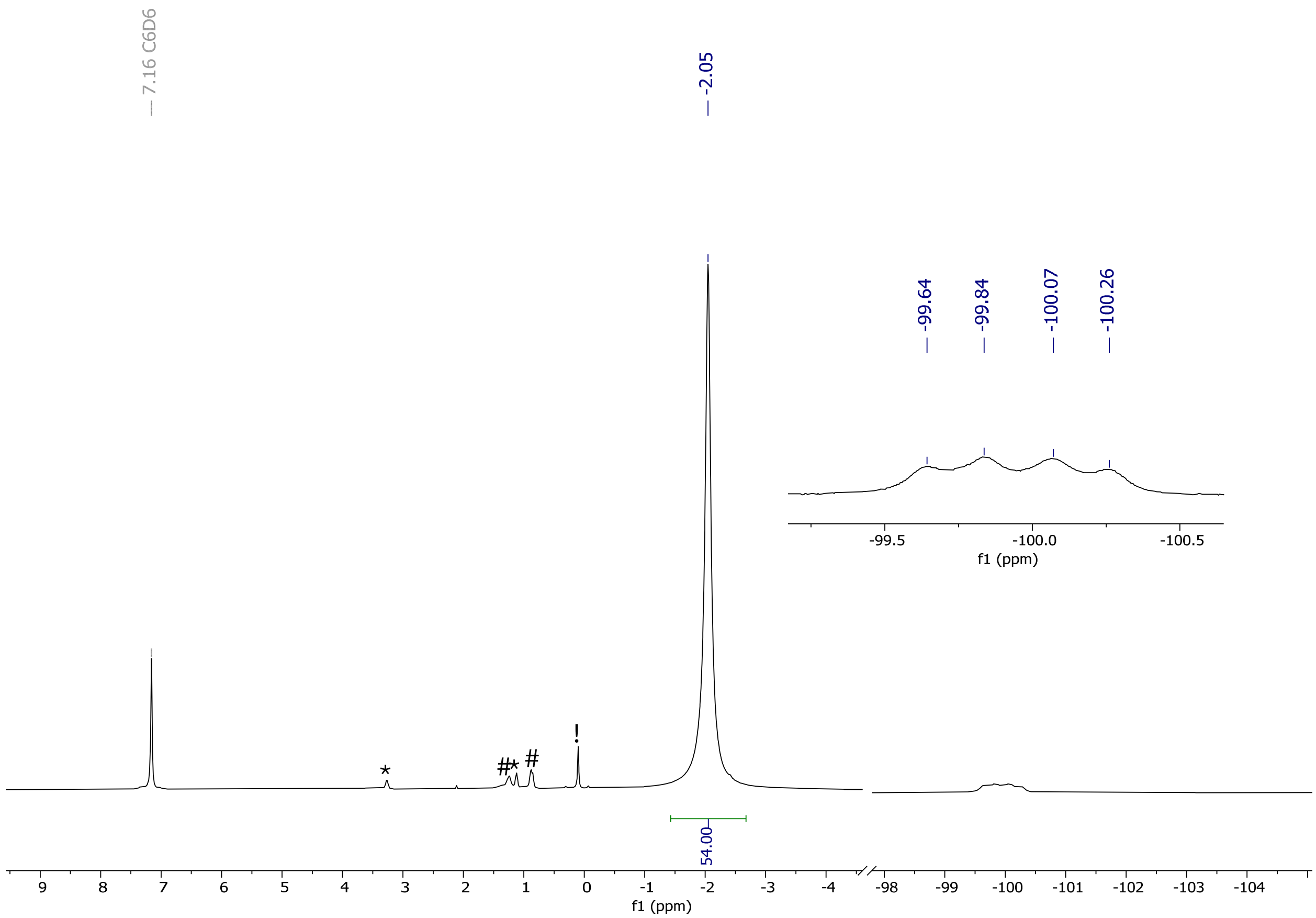

Figure S3. ^{1}H NMR spectrum of **2** (M = U), recorded in benzene-d_6. (*) indicates resonances assignable to diethyl ether. (#) indicates resonances assignable to pentane. (!) indicates resonances assignable to HMDS. Inset: expansion of the BH_4 resonance.

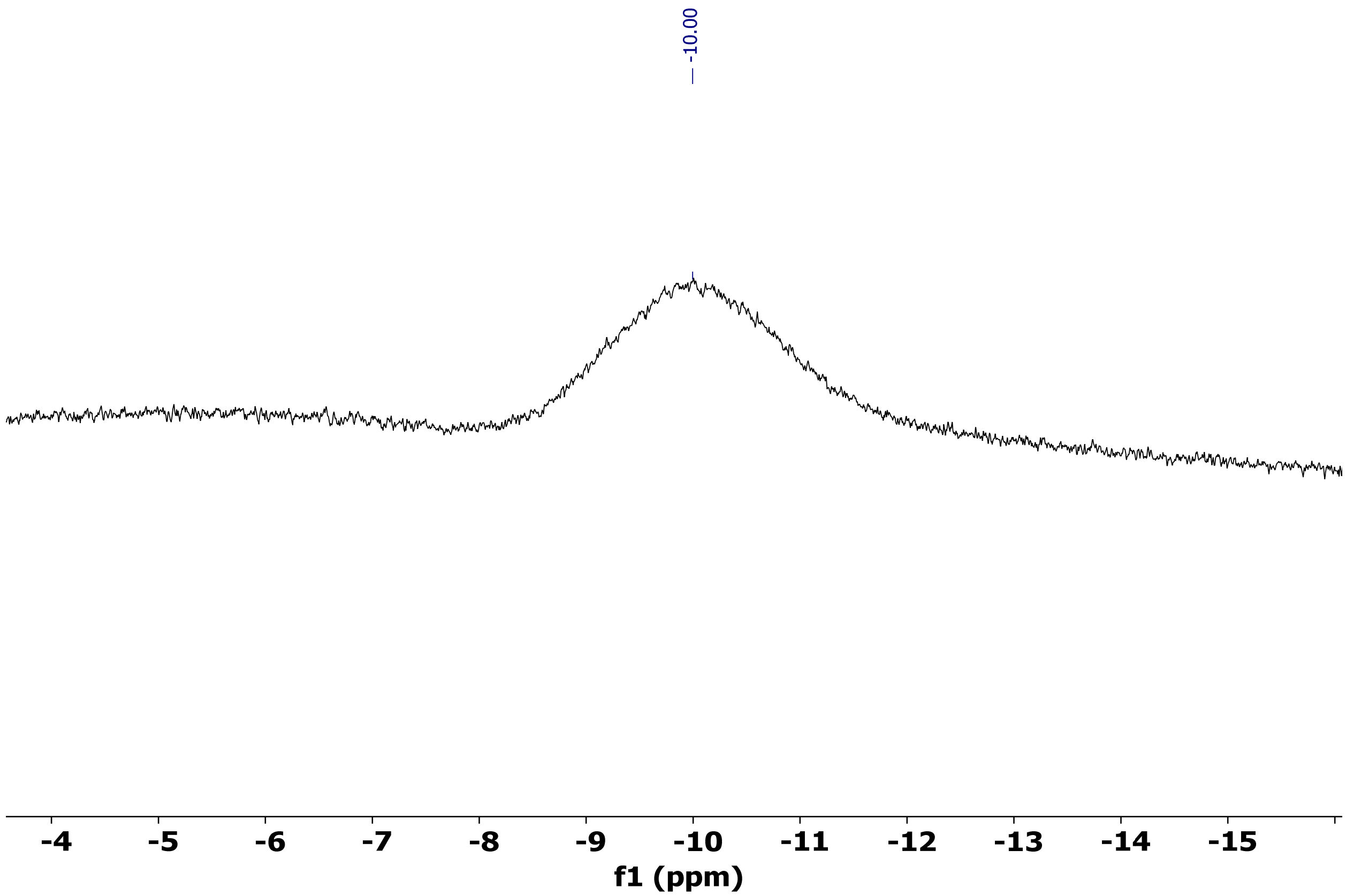

Figure S4. ^{11}B NMR spectrum of **2** (M = U), recorded in benzene-d_6.

*

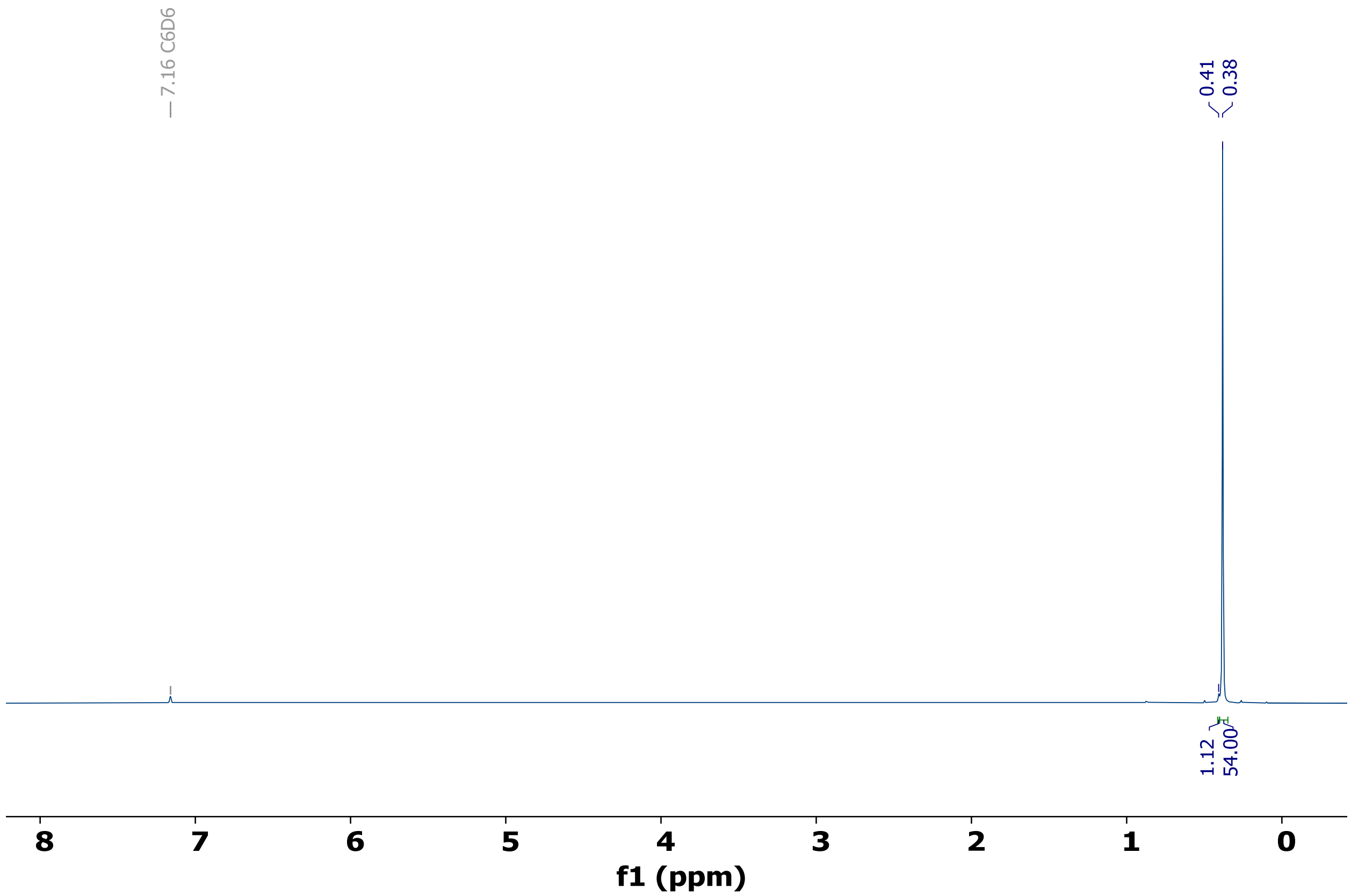

Figure S5. $^{1}H\{^{11}B\}$ NMR spectrum of **1-*d*$_4$** (M = Th), recorded in benzene-*d*$_6$. (*) indicates resonances assignable to $[ThCl(NR_2)_3]$.

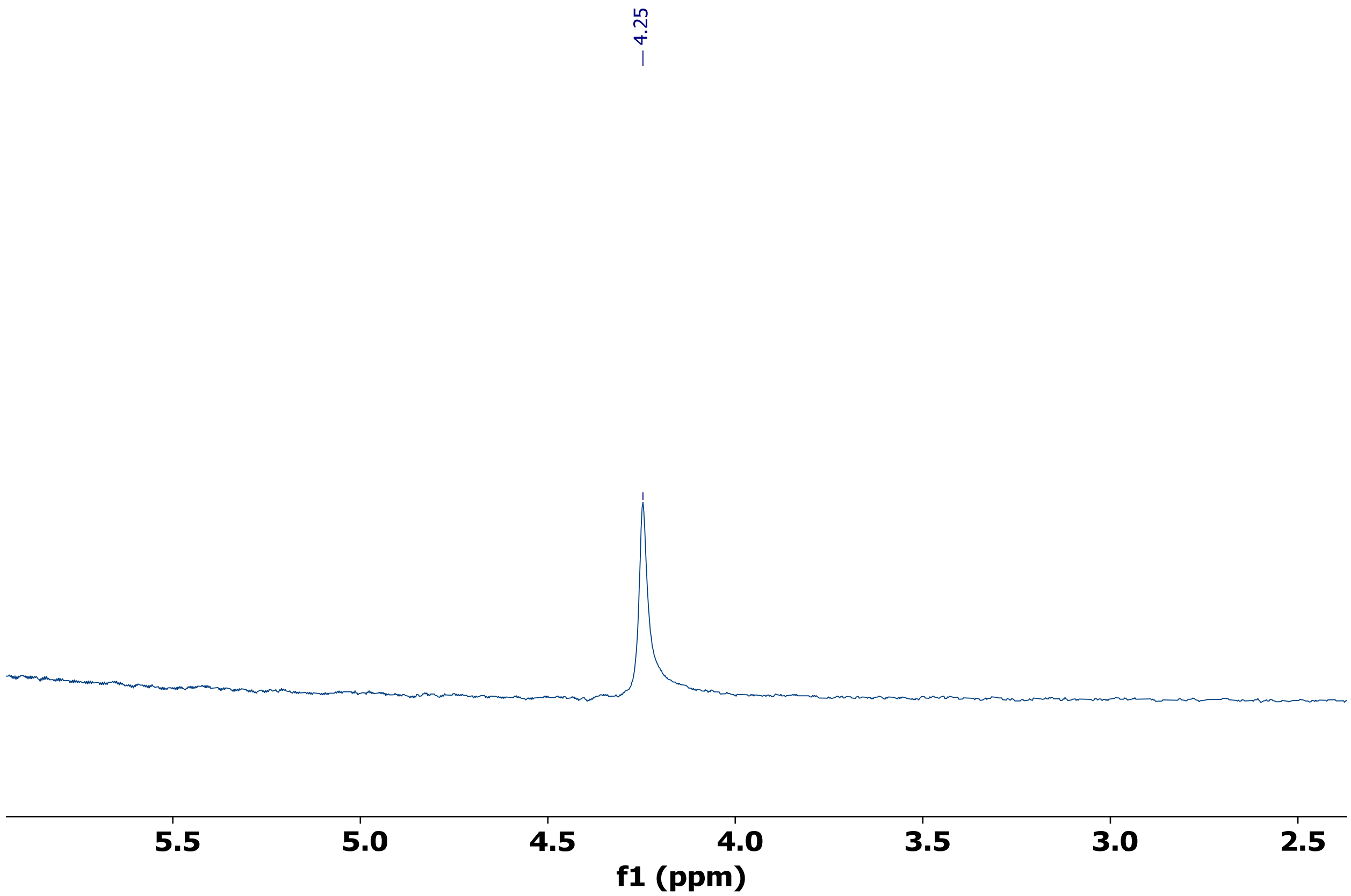

Figure S6. $^{2}H\{^{11}B\}$ NMR spectrum of **1-*d*$_4$** (M = Th), recorded in benzene-*d*$_6$.

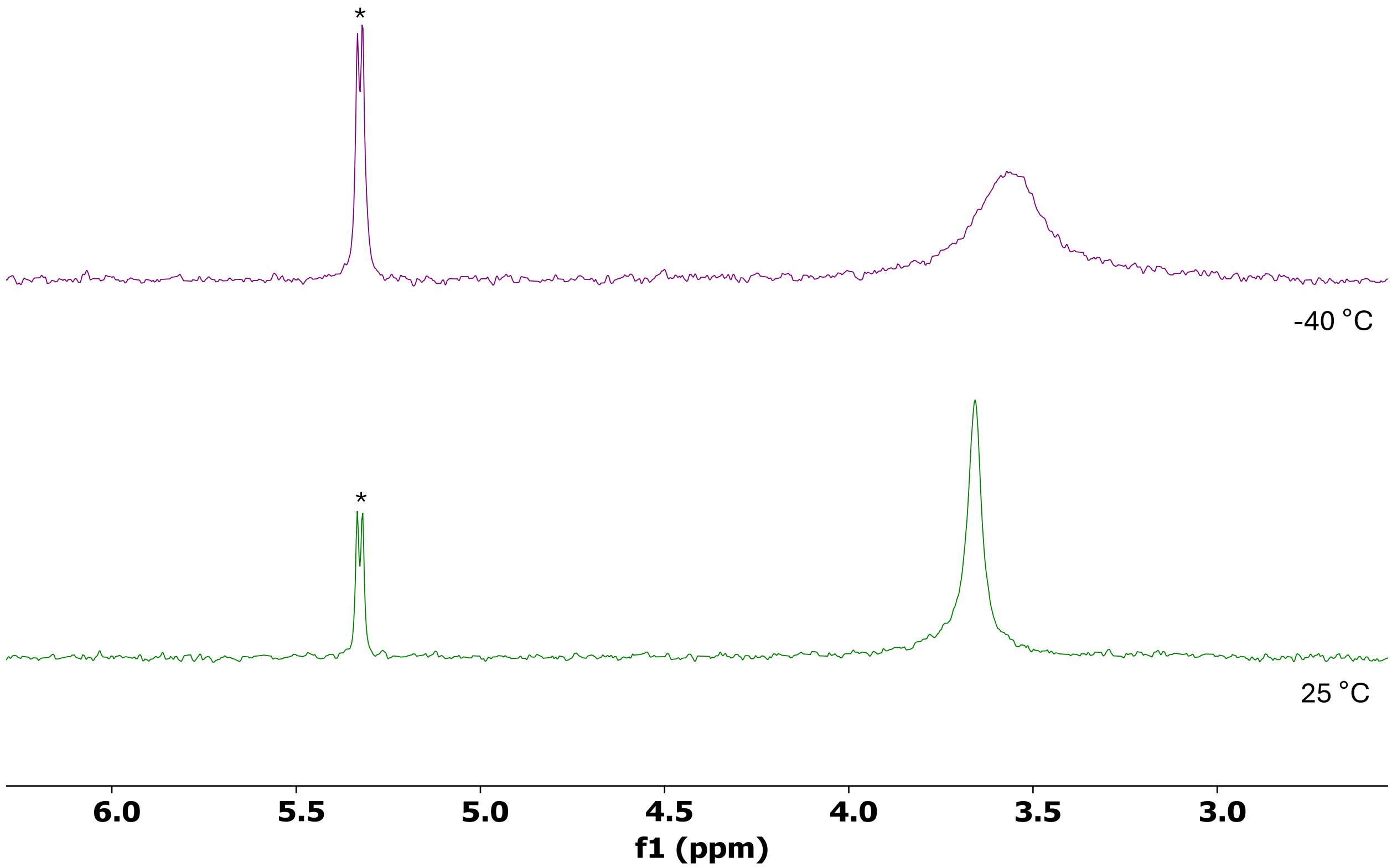

Figure S7. Variable temperature $^{2}H\{^{11}B\}$ NMR spectra of **1-*d*$_4$** (M = Th), recorded in DCM. (*) indicates resonances assignable to $CHDCl_2$.

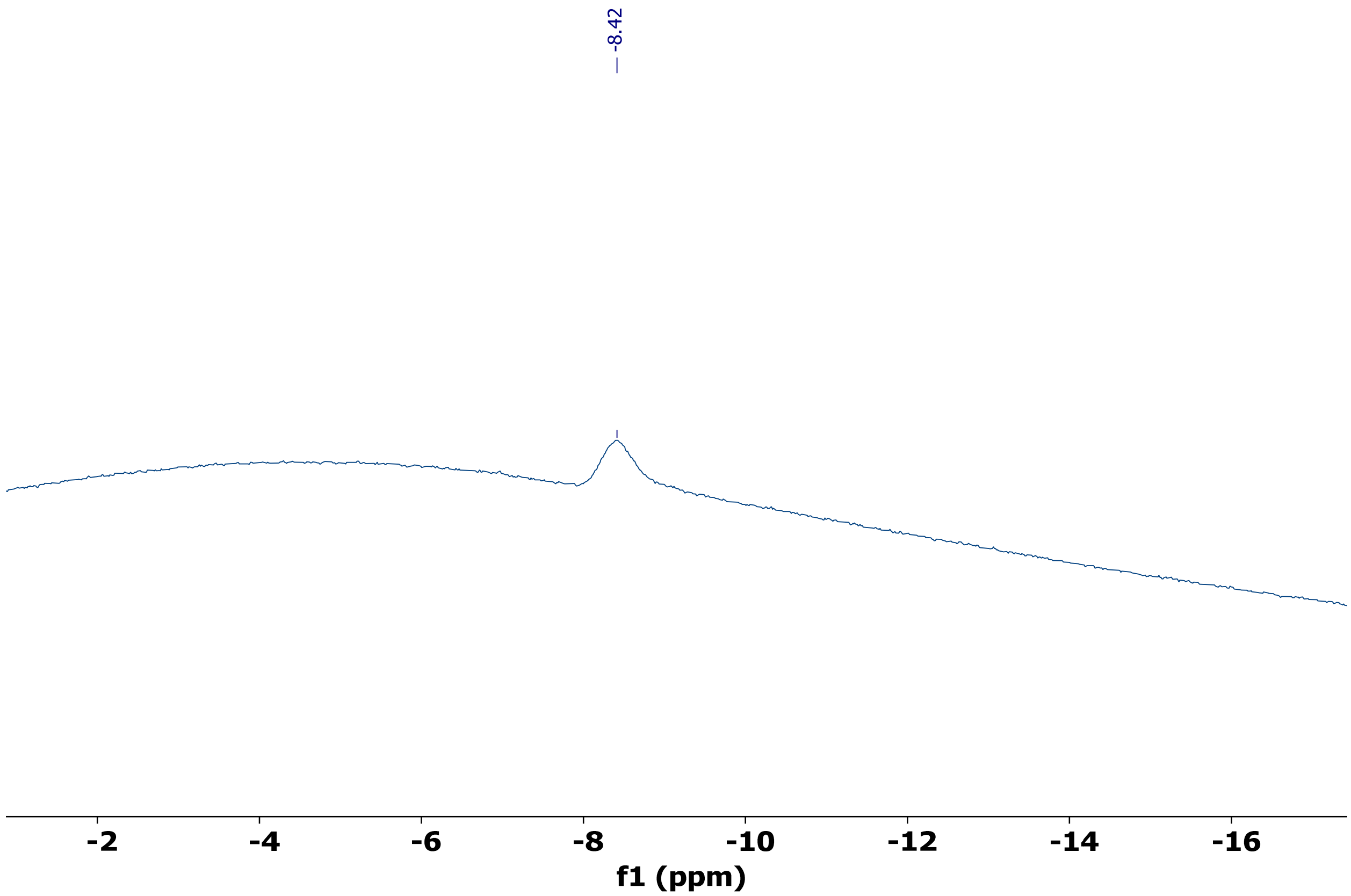

Figure S8. $^{11}B\{^{1}H\}$ NMR spectrum of **1-*d*₄** (M = Th), recorded in benzene-d_6.

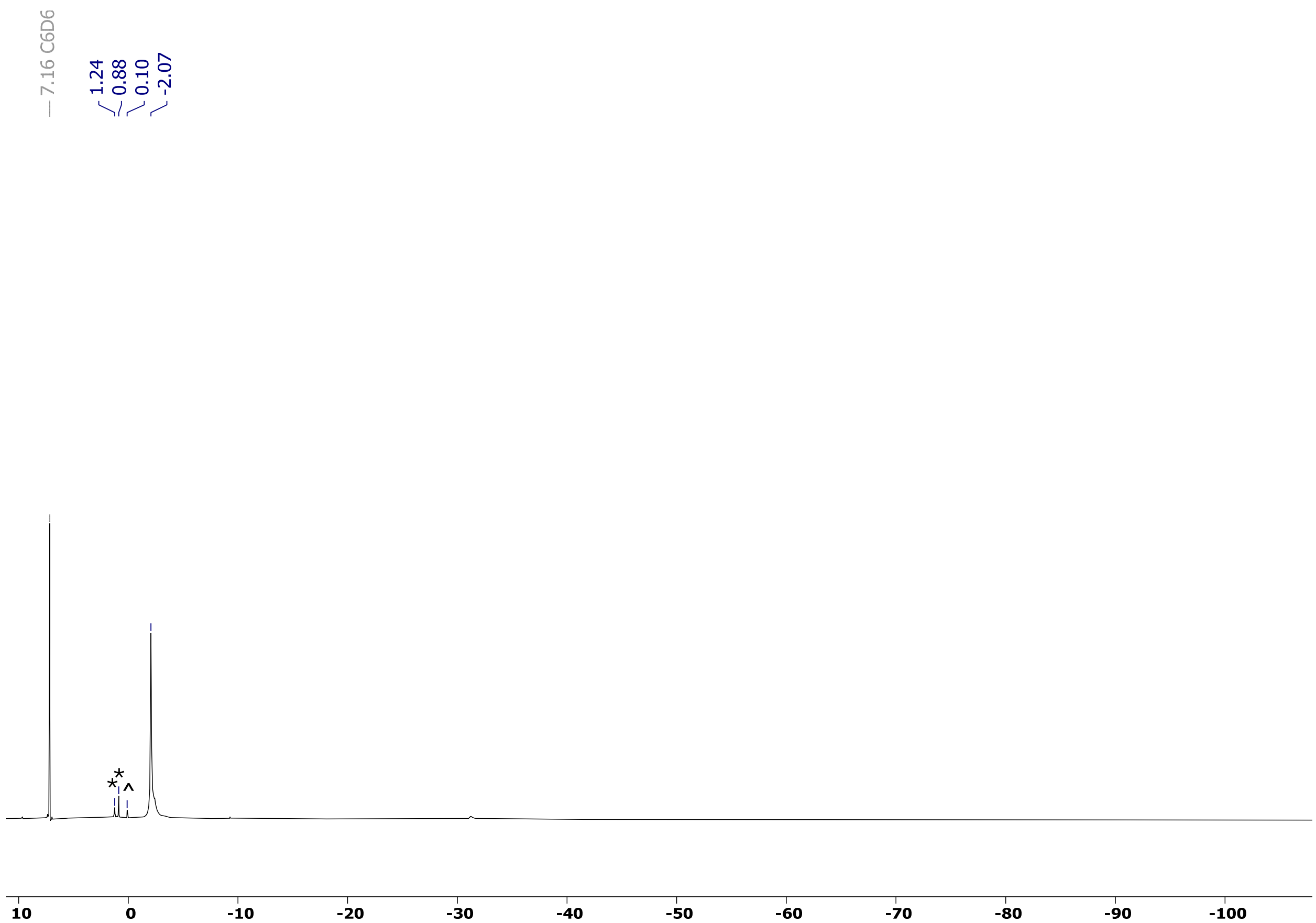

Figure S9. $^{1}H\{^{11}B\}$ NMR spectrum of **2-*d*$_4$** (M = U), recorded in benzene-d_6. (*) corresponds to resonances assignable to pentane. (^) corresponds to resonances assignable to free HMDS.

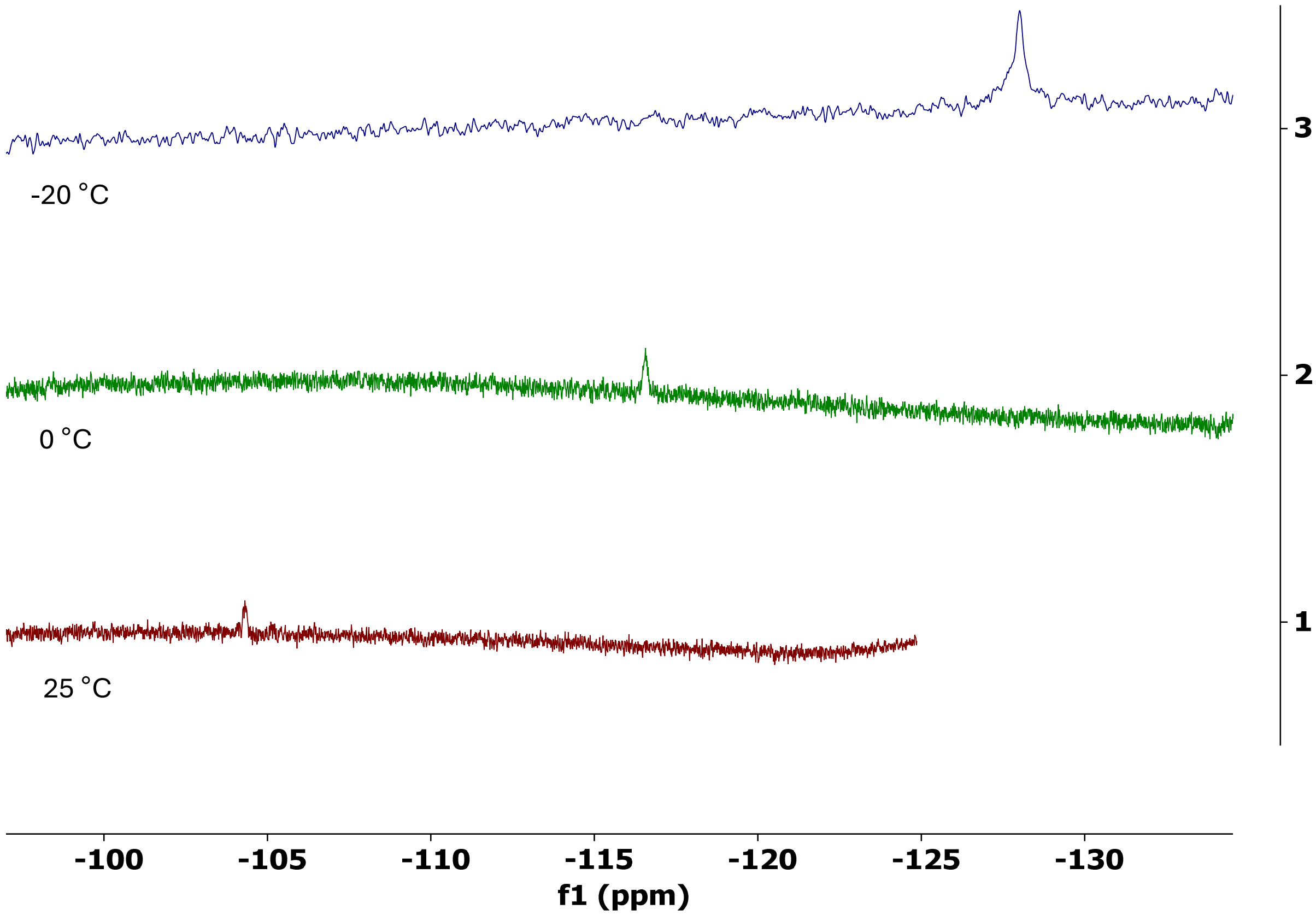

Figure S10. Variable temperature $^{2}H\{^{11}B\}$ NMR spectra of **2-*d*$_4$** (M = U), recorded in CH_2Cl_2.

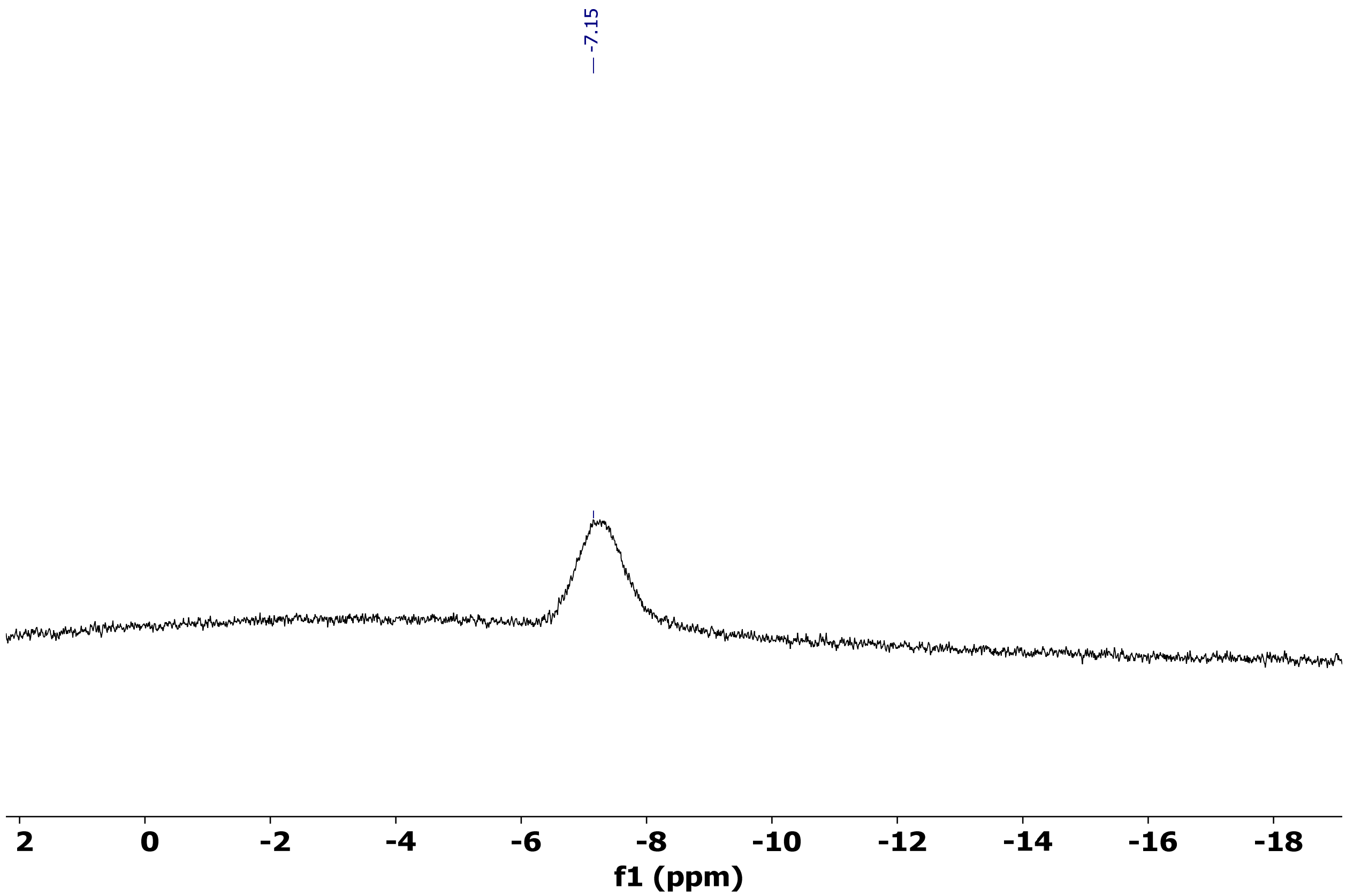

Figure S11. $^{11}B\{^{1}H\}$ NMR spectrum of **2-*d*₄** (M = U) recorded in benzene-d_6.

XAS Studies

B K-edge XAS data were collected at the Canadian Light Source (CLS) in Saskatoon, Saskatchewan, Canada on the Variable Line Spacing Plane Grating Monochromator (VLS-PGM) beamline. All measurements were performed in the Linear Horizontal Polarization mode using the high-energy grating (98–250 eV). The data were collected at room temperature in total fluorescence yield (FLY) mode using a microchannel plate (MCP) detector. Total electron yield (TEY) data was collected concurrently. The samples were positioned 45° with respect to the incoming beam and 10 cm away from the detector located perpendicular to the beam (90°). Beamline slit sizes used for collection were 100 x 100 μm. The sample chamber was maintained between 10^{-8} and 10^{-9} Torr for all measurements. Data were normalized to the I_0 current measured using a nickel mesh (90% transmission) placed in front of the sample. The energy of the beamline was calibrated using the inflection point of the B K-edge XAS spectrum of elemental boron (188.0 eV; Figure S28). The energy calibration was routinely assessed throughout the experimental runs by scanning elemental boron and/or boron nitride, which revealed no evidence of beam energy drift (Figure S29).

B K-edge XAS data were collected on at least three unique sample points to minimize beam-induced photodecomposition except when explicitly testing scan-to-scan photodecomposition by repeating scans at the same sample position. Data were collected using two different methods: (1) using a continuous scan (*i.e.* no piezoelectric shutter) at step sizes of ca. 0.1 eV and a dwell time of 1.0 s; (2) using a step scan with 0.5 s dwell times and a piezoelectric shutter between steps to further minimize beam-induced photodecomposition. The step sizes were 0.25 eV (185-187 eV), 0.1 eV (187-196 eV), 0.25 eV (196-210 eV), and 1.0 eV (210-240 eV). Continuous scan data collected in 2024 at CLS were collected in the region of 185-210 eV. These scans took approximately 7 min to collect. On a second run in 2026, we decreased the energy range of the continuous scans to 185-196 eV. This smaller energy range further reduced the time of scans to < 4 min. The data were worked up and normalized via two different methods, depending on the experimental runs (2024 or 2026). This choice was made based upon pre-edge and post-edge fitting challenges for the 2026 data and lack of an extended post-edge for 2024 data where scans were only collected up to 210 eV (compared to 240 eV in 2026). The B K-edge XAS spectra collected in 2024 at CLS were baselined and normalized using Python[10-14] scripts we developed. These scripts baseline subtracted each spectrum between 184 and 187 eV and normalized the spectrum by taking the intensity of the last point (210 eV) and dividing all data points by the corresponding value to set that point to equal 1. Scans for each unique sample were then averaged to obtain the final spectra shown in this work. Alternatively, the data collected in 2026 were worked up and normalized using Athena[15] by fitting each spectrum collected on the same unique sample run in the pre-edge and post-edge for baseline and normalization fitting with E0 = 200 eV. Each scan was fit in the pre-edge and post-edge the same way and merged (averaged) as the normalized μ(E). The merged spectrum was then exported as the μ(E) and imported into Igor Pro,[16] where a binomial smoothing of 2 was applied.

Samples were prepared in an Ar-filled glovebox (Innovative Technologies) with less than 1.0 ppm of O_2 and H_2O at CLS. Single crystals of each complex were ground by hand in a mortar and pestle for approximately 60 s. The pestle was used to gently press a thin layer of the powder into In foil (Thermo Fisher) adhered to a copper sample plate with double sided Cu tape (SPI Supplies). The Cu tape and plate assembly were heated together in a muffle furnace at 150 °C for *at least* 90 min and then allowed to cool in the glovebox antechamber under vacuum. The In foil was left stored under vacuum in the glovebox antechamber for ca. 24 h before use and applied to the Cu tape in the glovebox. Samples were transported to the beamline under Ar in sealed glass jars that were treated the same way as the sample plates (heated to 150 °C for at least 90 min). Once at the beamline, the sealed sample jars were placed in the antechamber of an N_2-purged glovebox attached to the beamline sample loading chamber. The antechamber was purged for ca. 2 h before transferring them to the N_2-purged glovebox attached to the beamline sample loading

chamber. The main chamber was continuously purged with N_2 over the course of the entire data collection run using blowoff from 180 L LN2 dewars. The N_2-purged glovebox atmosphere was tested at periodic intervals by exposing electrified filaments of NOMA 7 w or 5 w incandescent night light bulbs. The light bulbs consistently remained lit for >30 min with the longest surviving 86 min. The sample loading exposed the samples to the glovebox atmosphere for no more than 30 s. Upon loading, the beamline sample-loading chamber was immediately evacuated to ca. 10^{-6} torr upon loading for transfer to the main data collection chamber maintained at 10^{-8} – 10^{-9} torr.

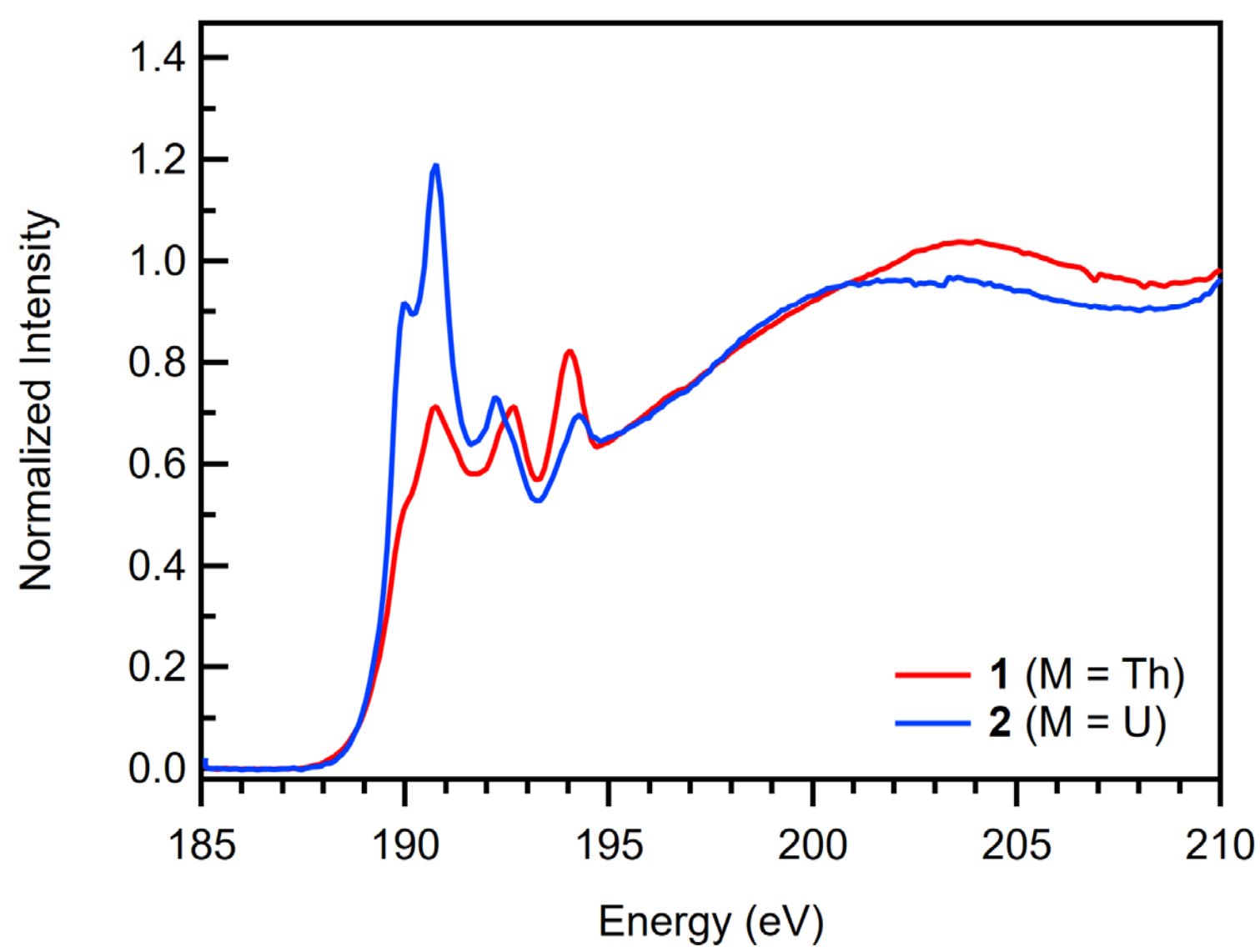

Figure S12. Full B K-edge XAS spectra (FLY) of **1** (M = Th) and **2** (M = U) collected using continuous scans and 1.0 s dwell times in 2024 at CLS (185 – 210 eV range).

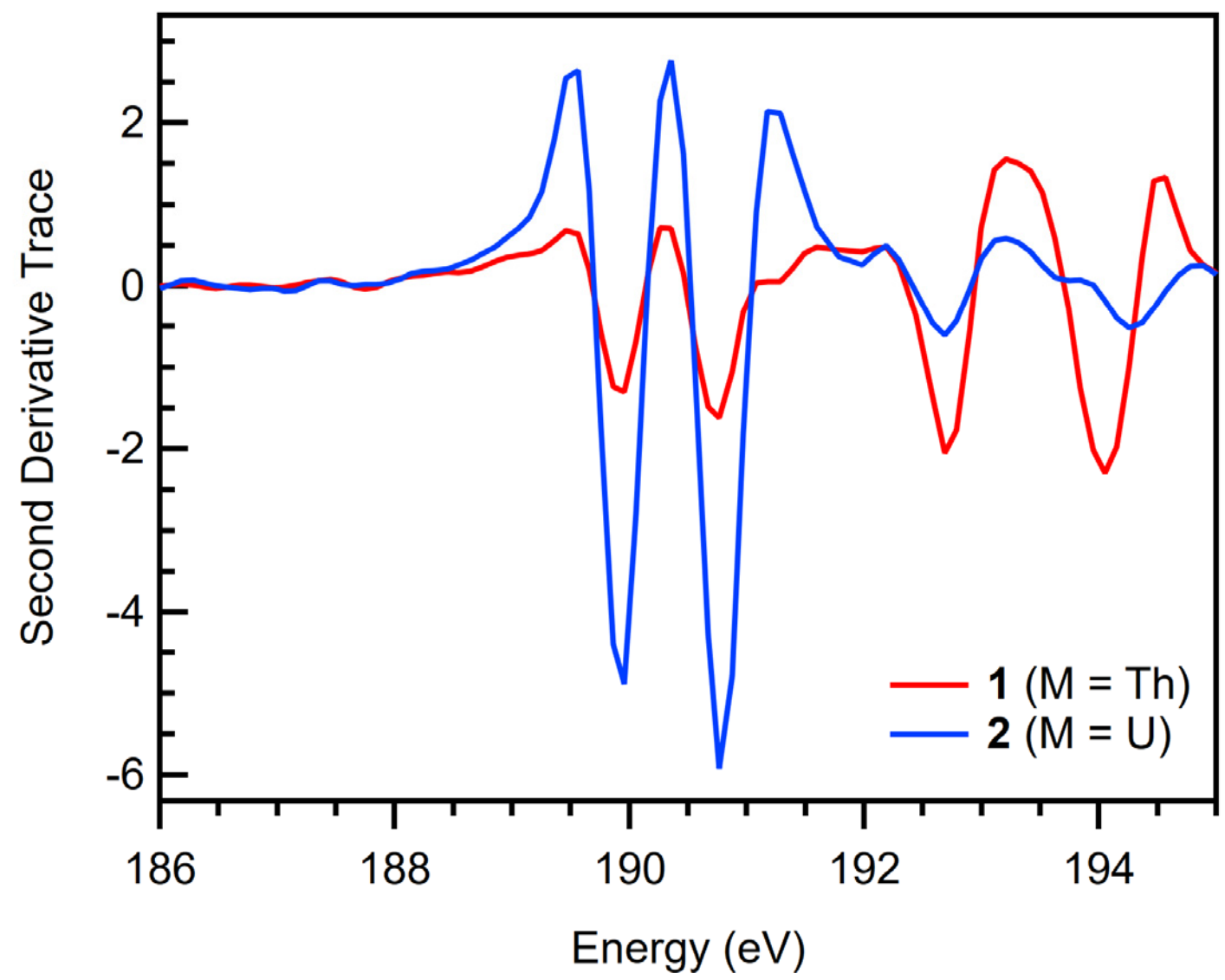

Figure S13. Second derivative traces of B K-edge XAS spectra (FLY) for **1** (M = Th) and **2** (M = U) shown in Figure S12 and collected using continuous scans with 1.0 s dwell times in 2024 at CLS (185 – 210 eV range).

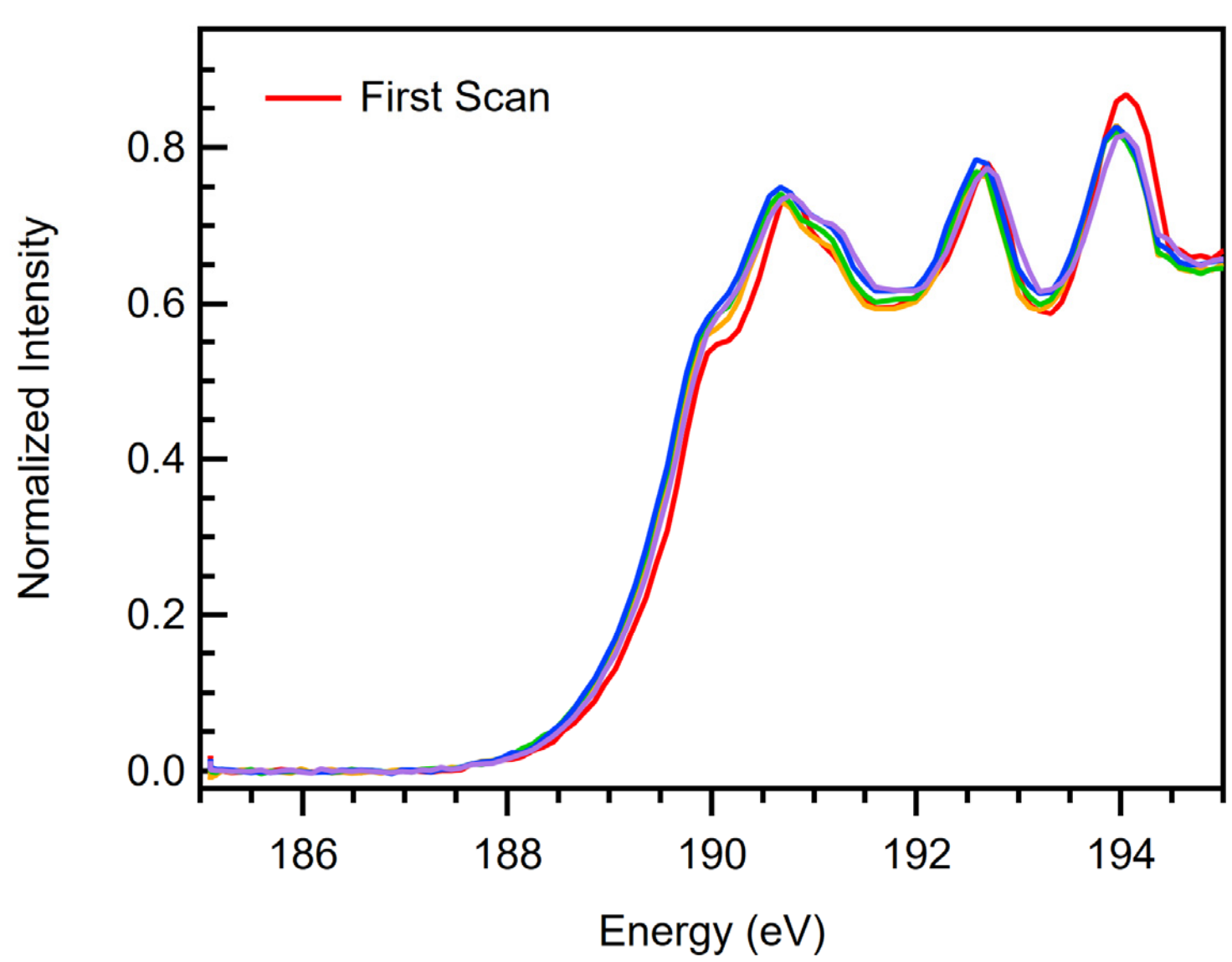

Figure S14. Repeat B K-edge continuous FLY scans of **1** (M = Th) collected on the same spot using 1.0 s dwell times in 2024 at CLS (185 – 210 eV range).

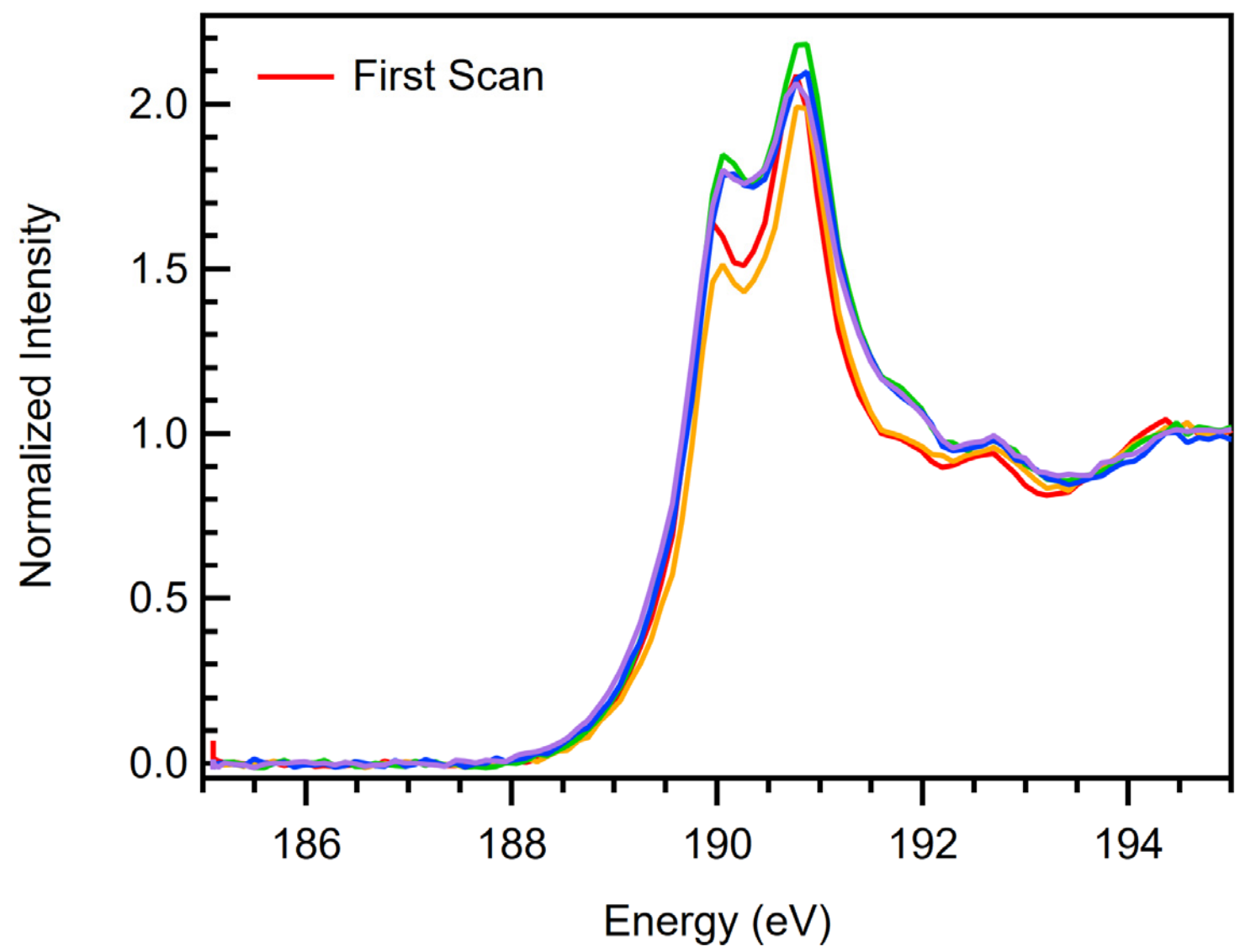

Figure S15. Repeat B K-edge continuous FLY scans of **2** (M = U) collected on the same spot using 1.0 s dwell times in 2024 at CLS (185 – 210 eV range).

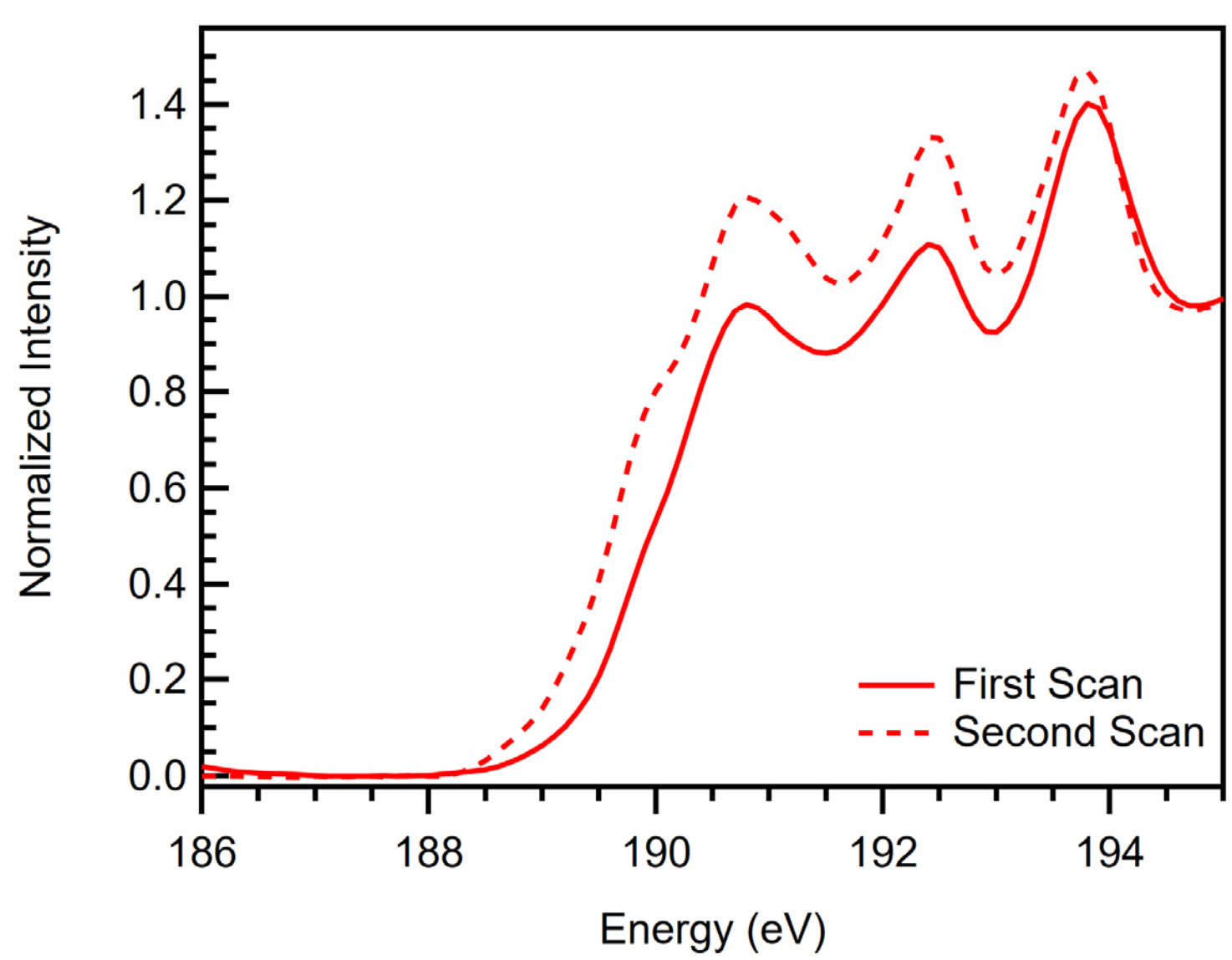

Figure S16. B K-edge XAS step scan spectra (FLY) of **1** (M = Th) collected on the same sample position using 0.5 s dwell times.

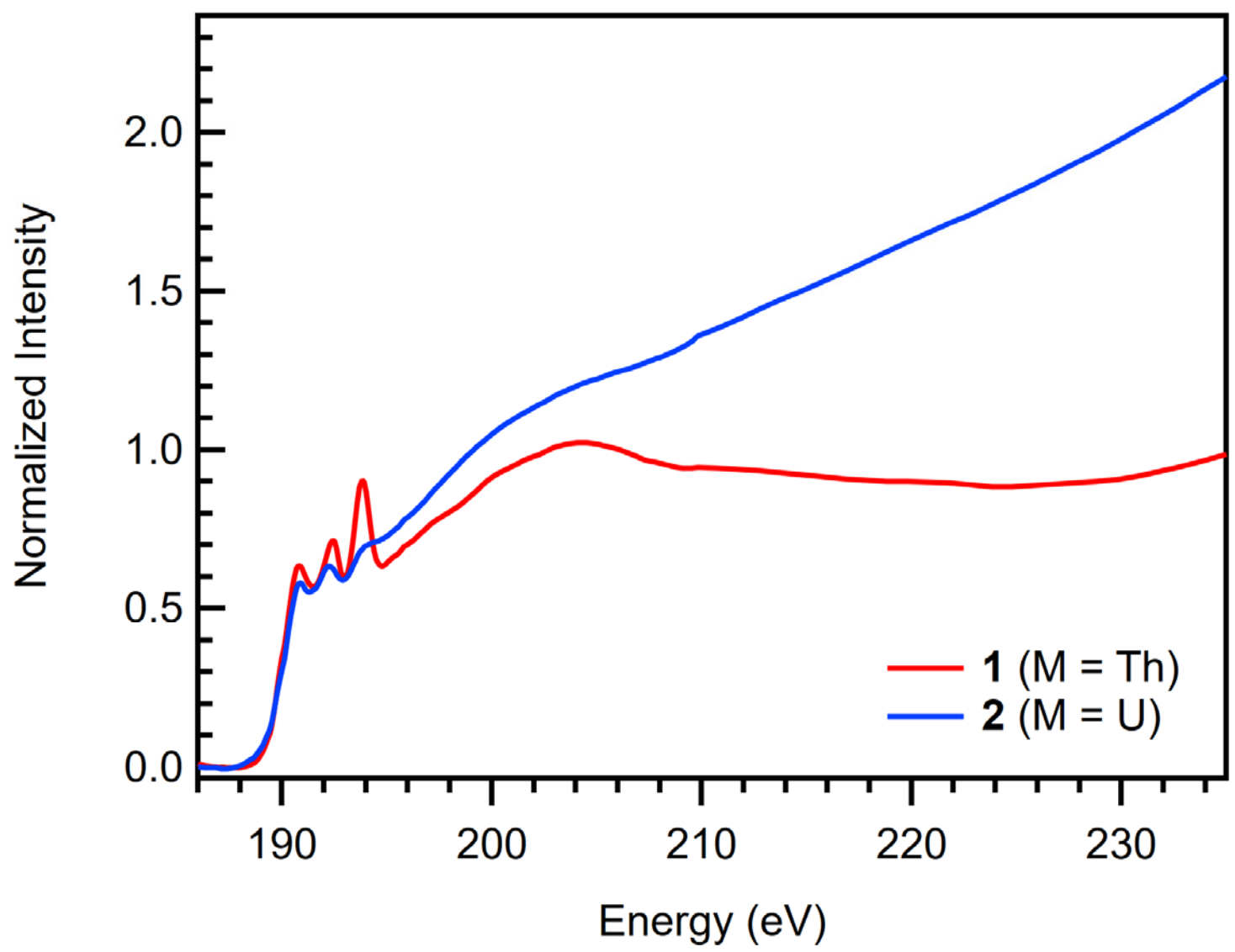

Figure S17. Full B K-edge XAS spectra (FLY) of **1** (Th; red) and **2** (U; blue) collected using step scans and 0.5 s dwell times in 2026 at CLS. The relatively large increase in normalized intensity as a function of energy in **2** is due to pre-edge and post-edge fitting challenges.

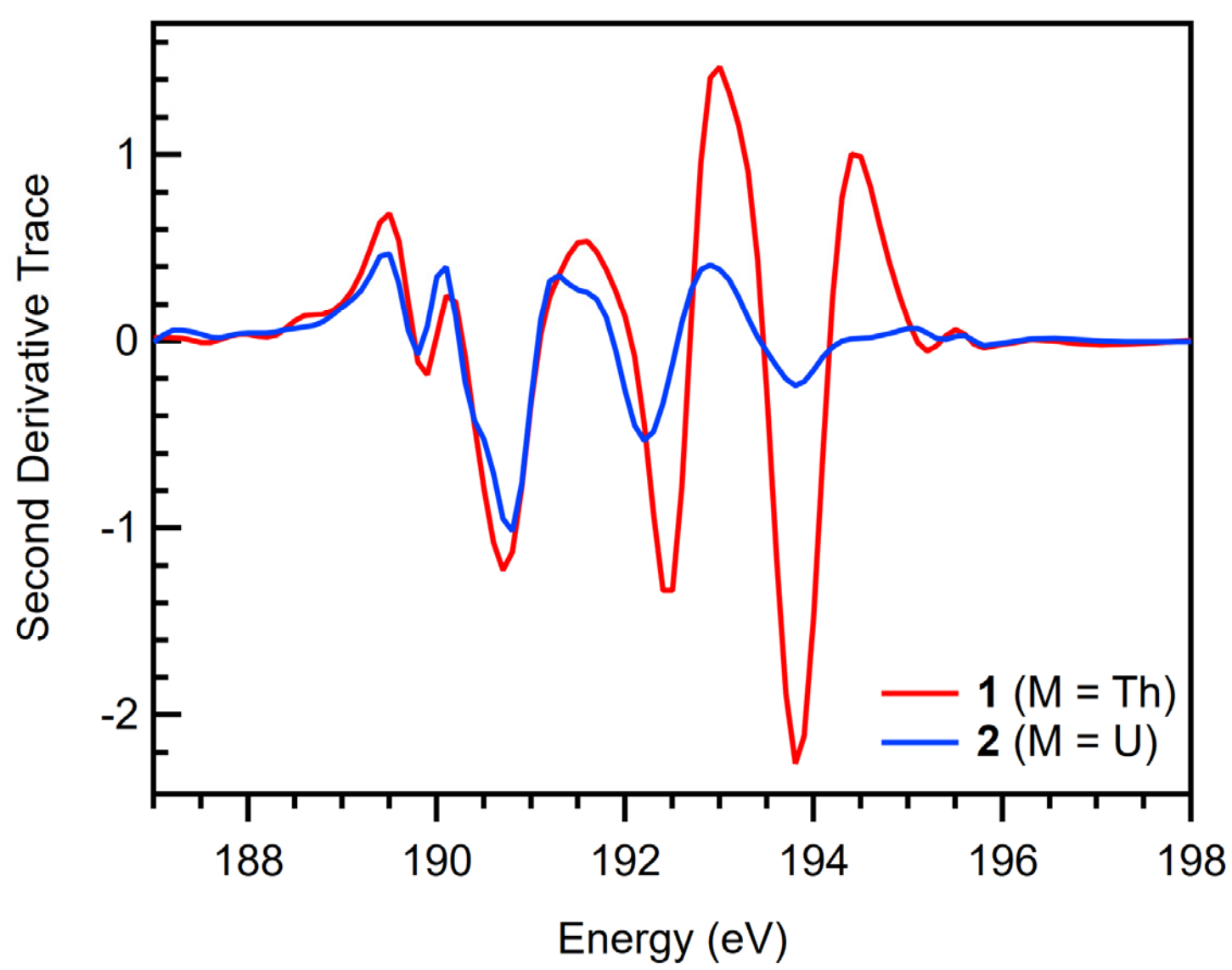

Figure S18. 2nd derivative traces of B K-edge XAS spectra (FLY) for **1** (Th; red) and **2** (U; blue) shown in Figure S17 and collected using step scans and 0.5 s dwell times in 2026 at CLS.

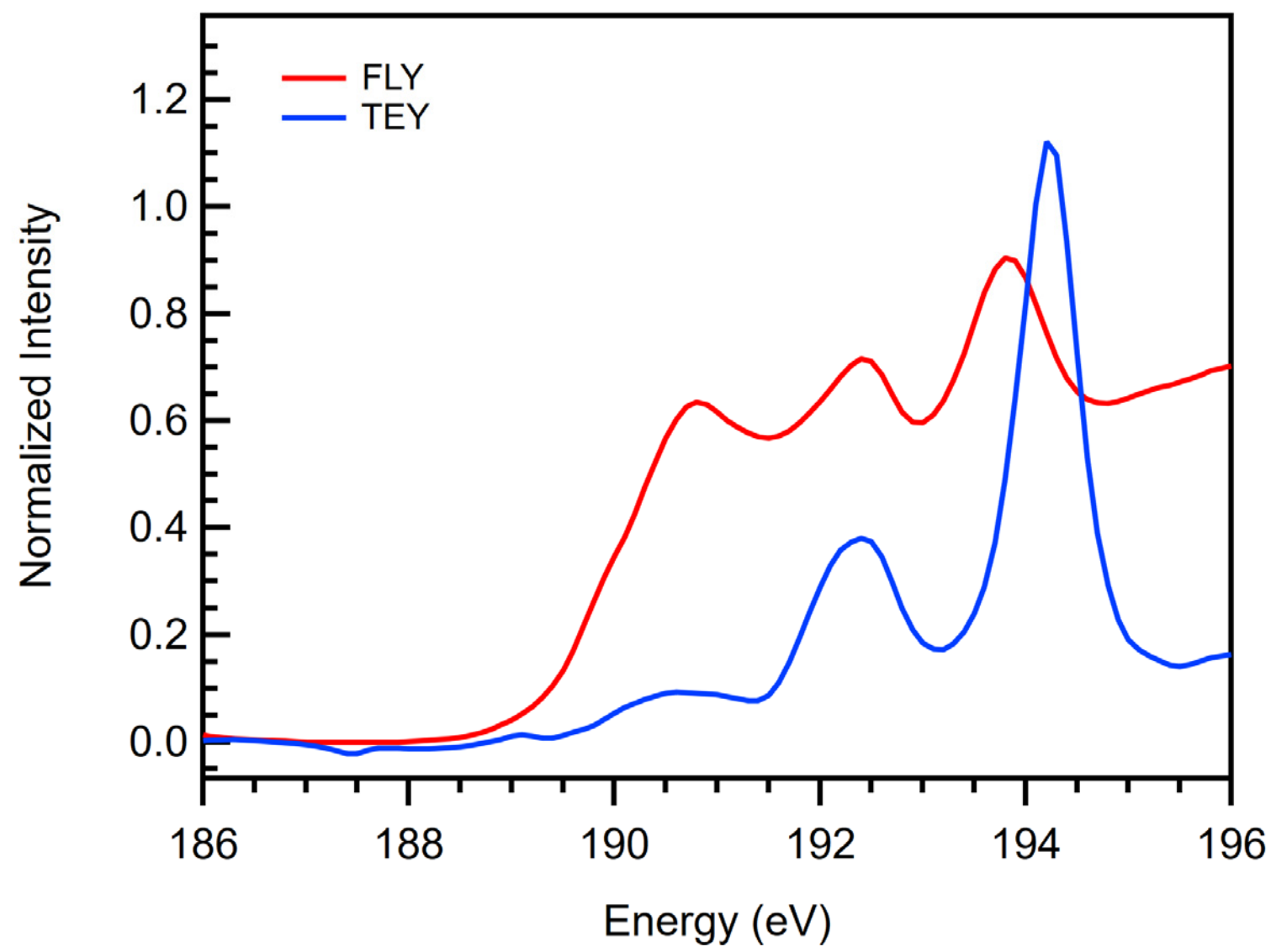

Figure S19. B K-edge XAS step scan spectra of **1** (M = Th) collected using 0.5 s dwell times in 2026 at CLS comparing total fluorescence yield (FLY; red) and total electron yield (TEY; blue). The features above 191.5 eV increase in intensity in the TEY spectrum due to increased hydrolysis on the surface of the sample.

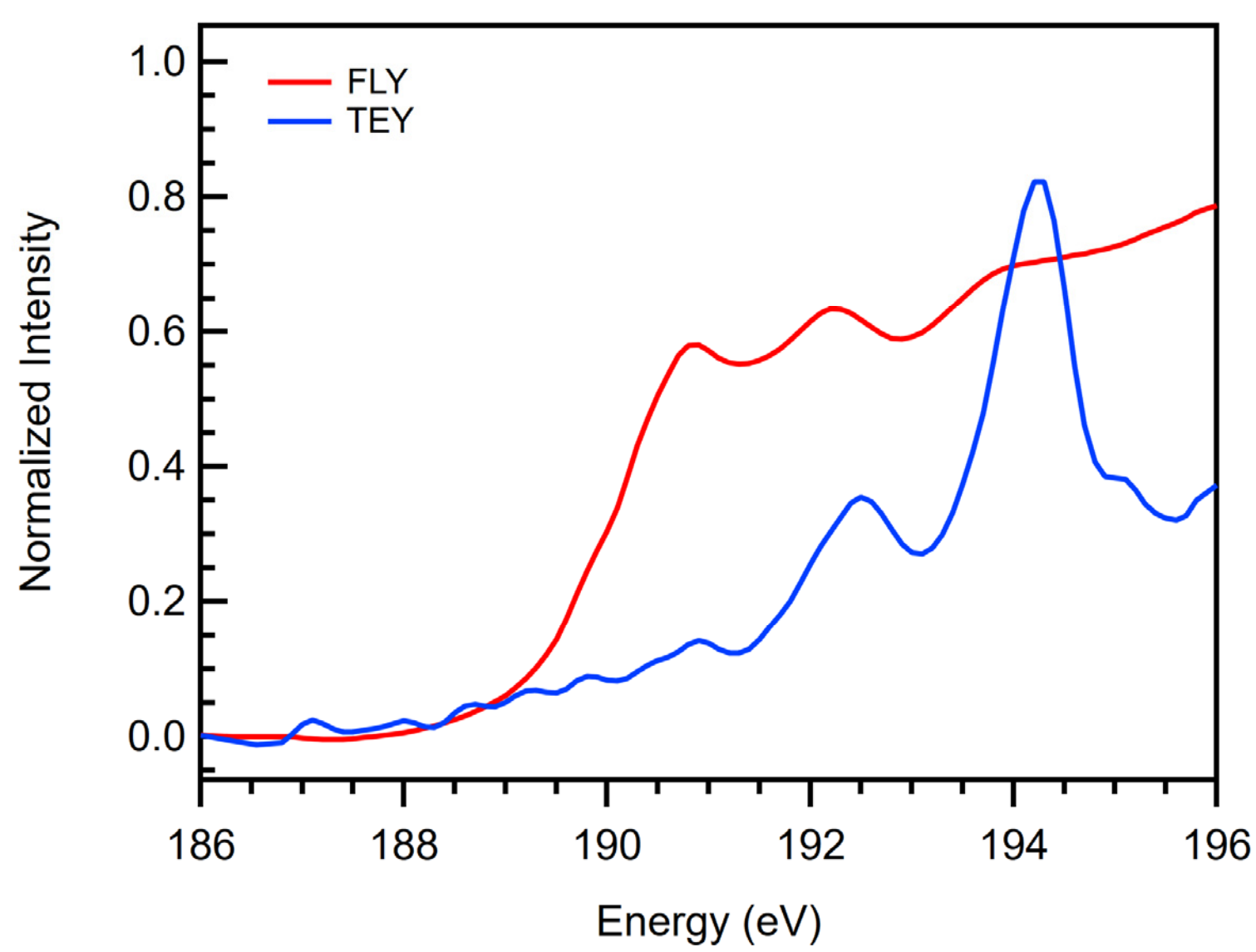

Figure S20. B K-edge XAS step scan spectra of **2** (M = U) collected using 0.5 s dwell times in 2026 at CLS comparing total fluorescence yield (FLY; red) and total electron yield (TEY; blue). The features above 191.5 eV increase in intensity in the TEY spectrum due to increased hydrolysis on the surface of the sample.

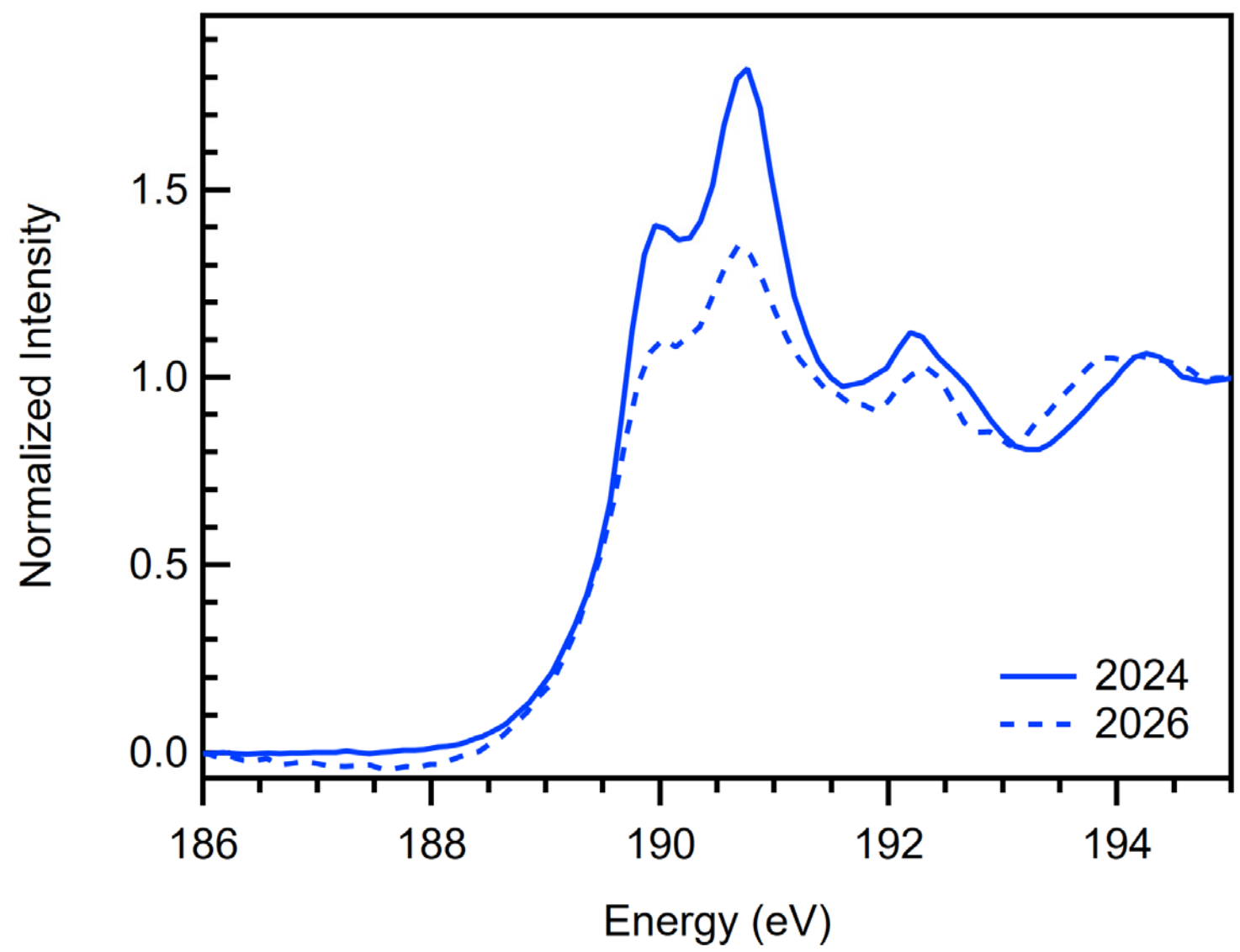

Figure S21. Comparison of B K-edge XAS continuous scan (FLY) spectra of **2** (M = U) collected using 1.0 s dwell times at CLS during 2024 and 2026 trips.

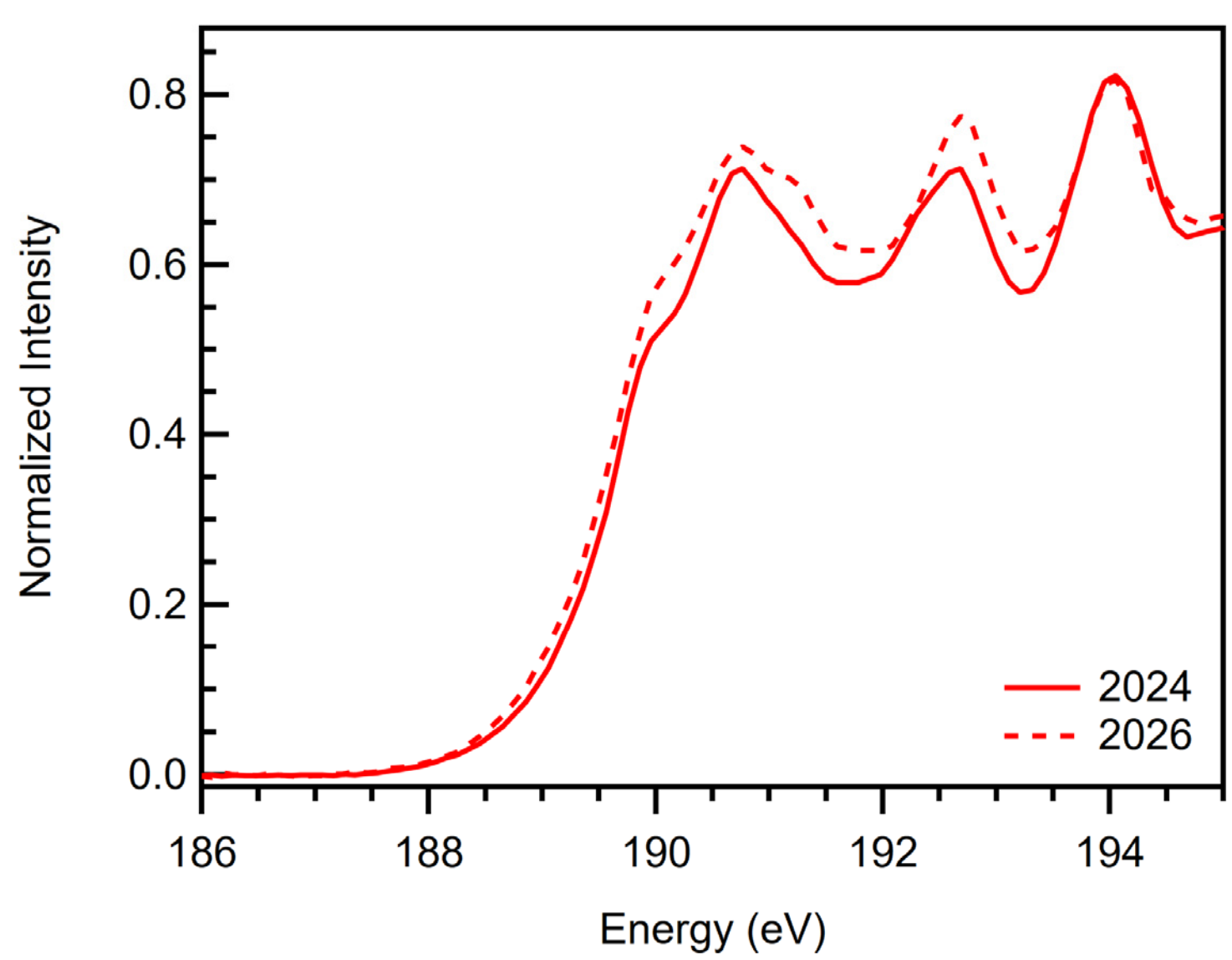

Figure S22. Comparison of B K-edge XAS continuous scan (FLY) spectra of **1** (M = Th) collected using 1.0 s dwell times at CLS during 2024 and 2026 trips.

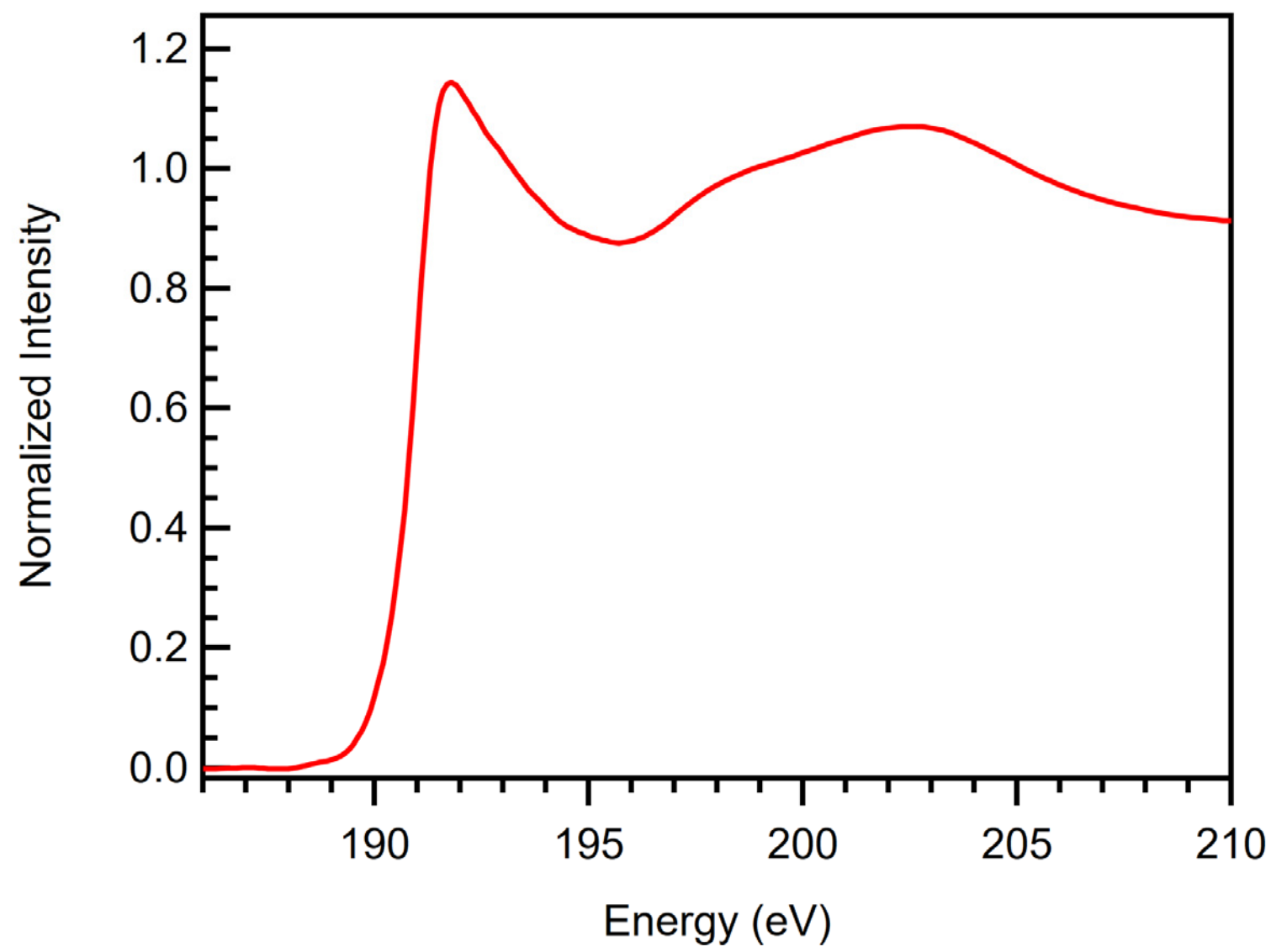

Figure S23. B K-edge XAS spectrum (FLY) of $Na(BH_4)$ collected using step scans and 0.5 s dwell times.

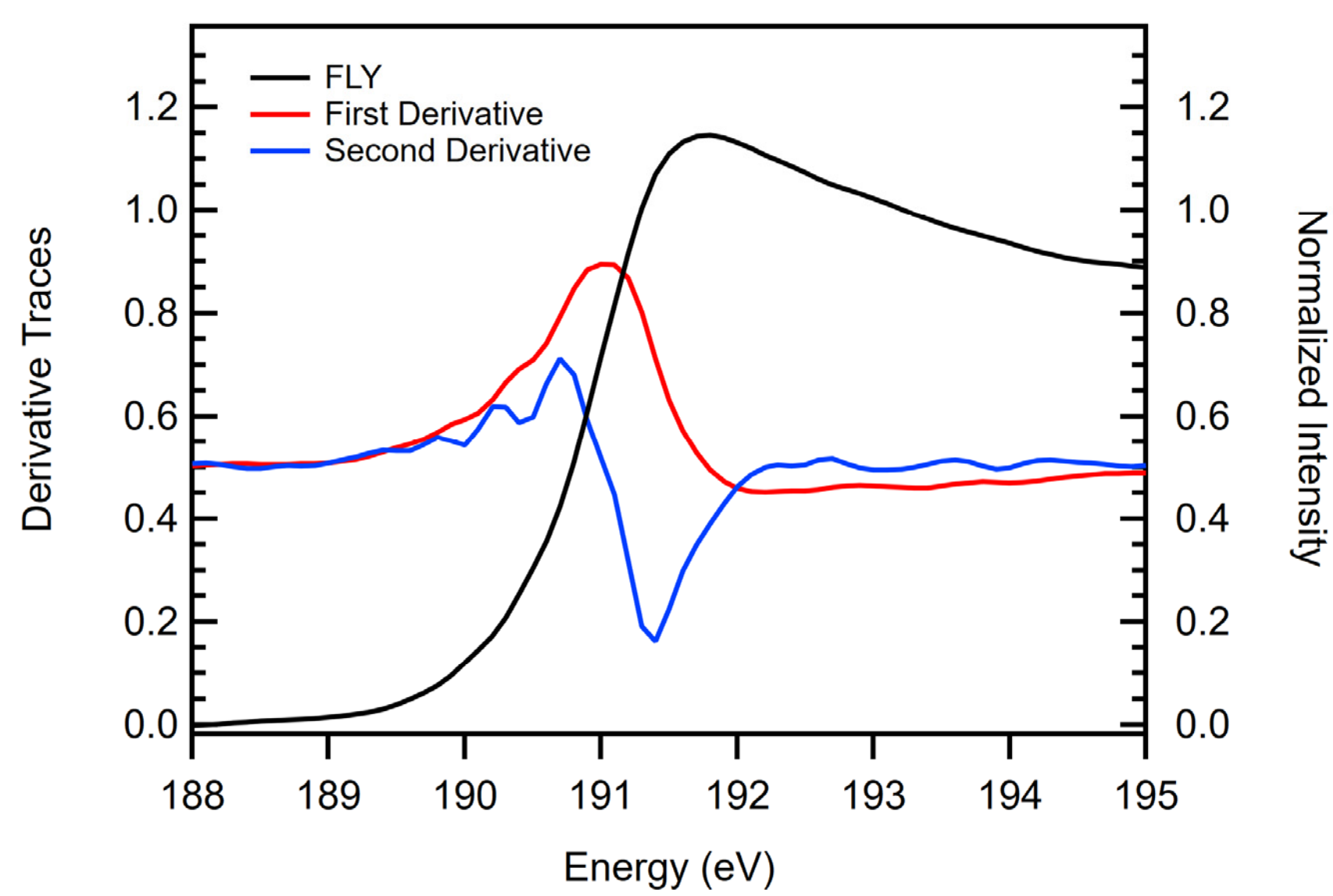

Figure S24. B K-edge XAS spectrum (FLY) of $Na(BH_4)$ and corresponding first and second derivative traces.

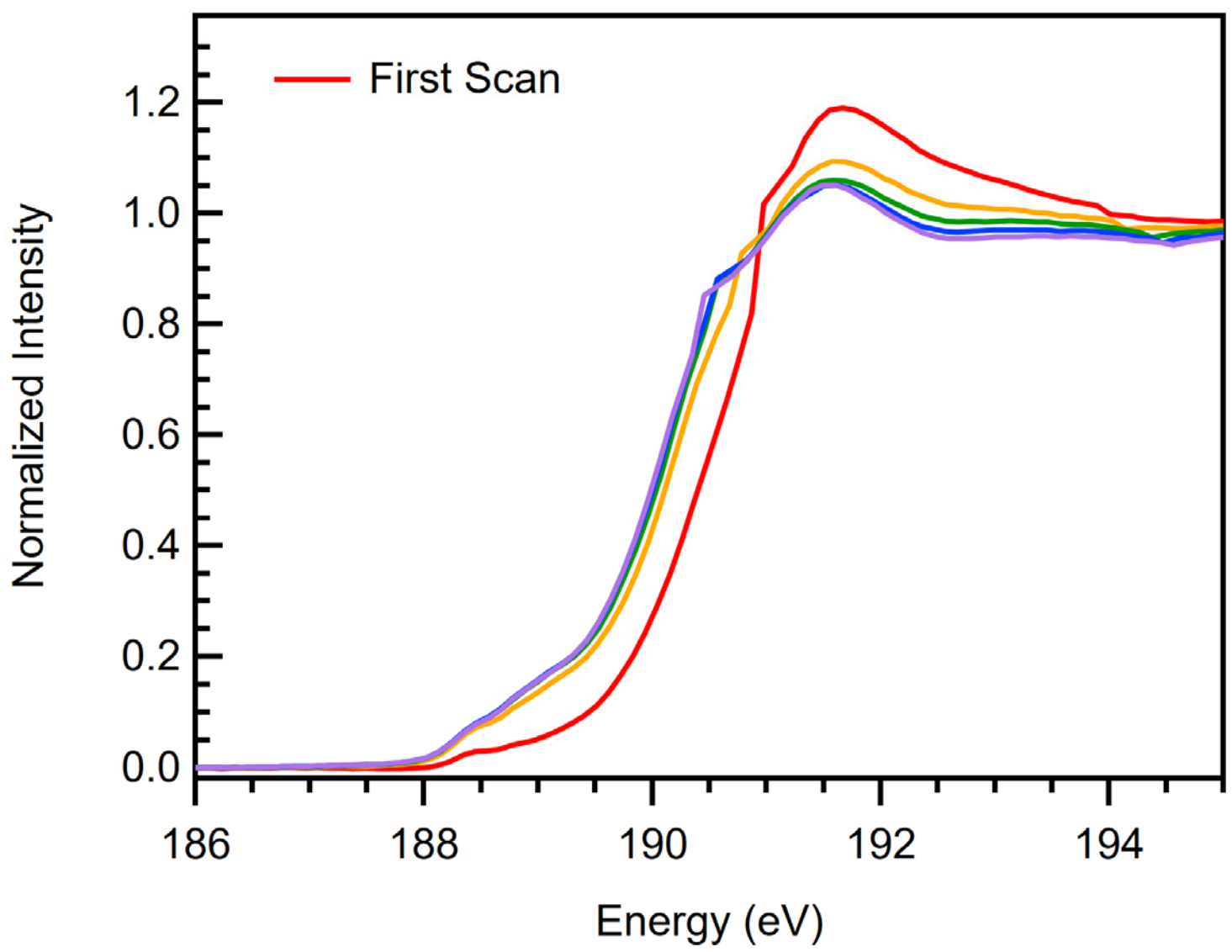

Figure S25. B K-edge XAS continuous scans (FLY) of $Na(BH_4)$ collected using 1.0 s dwell times on the same sample position.

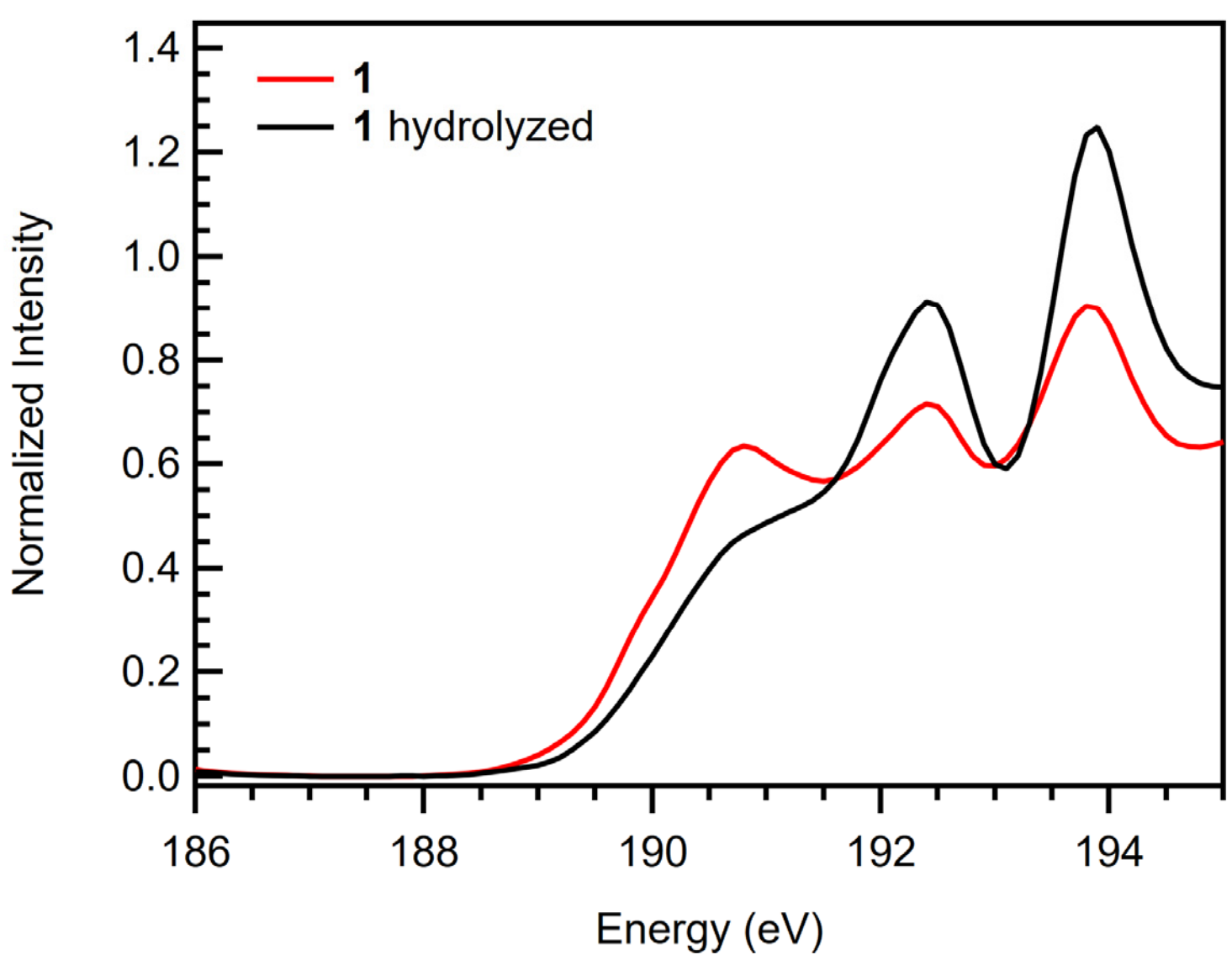

Figure S26. B K-edge XAS step scan spectra (FLY) of **1** (M = Th; red) and a sample of **1** representative of increased hydrolysis collected using 0.5 s dwell times. Hydrolysis causes the features below 191.5 eV to decrease in intensity and resolution while those above 191.5 eV increase in intensity.

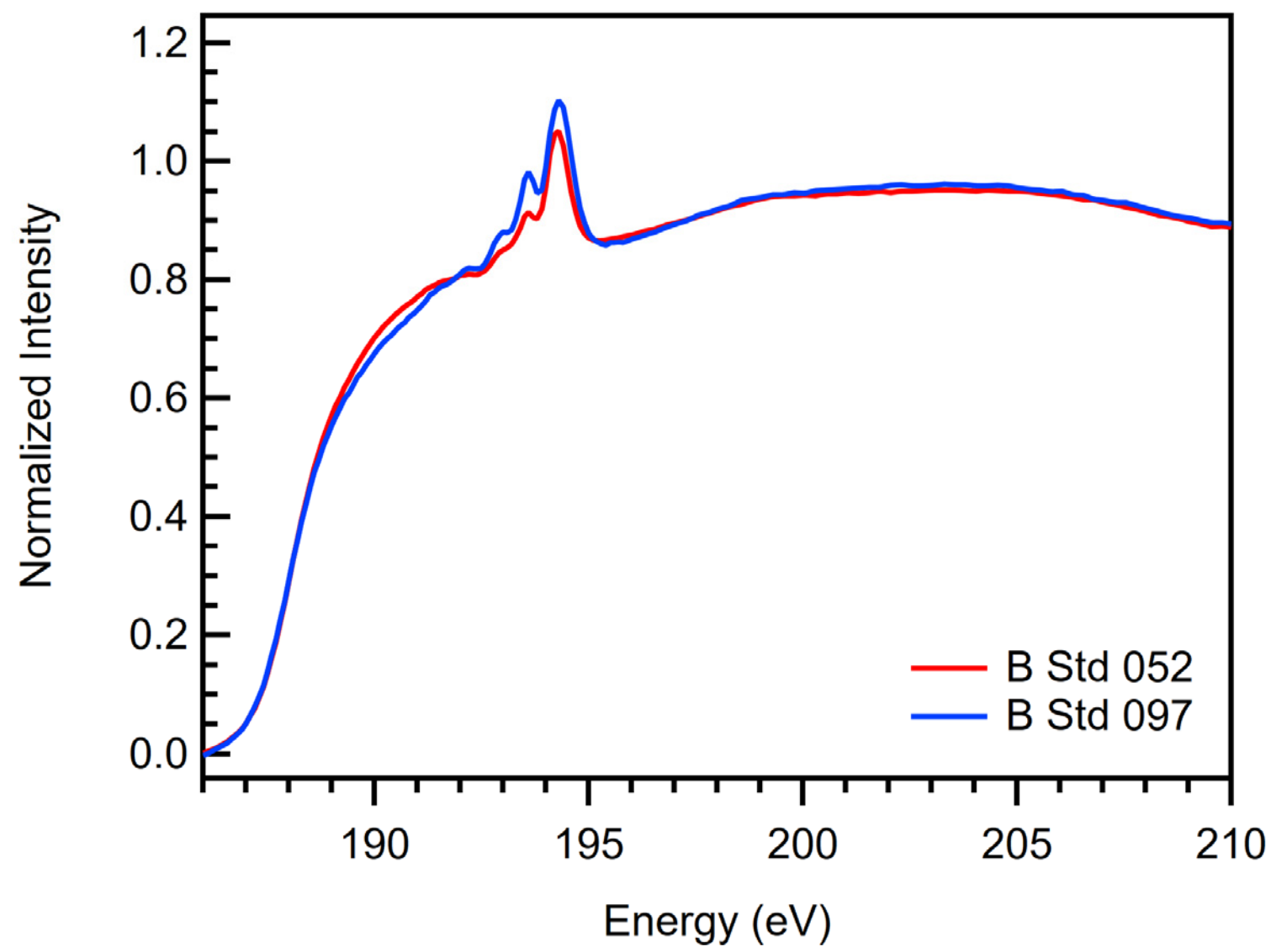

Figure S27. B K-edge XAS step scan spectra (FLY) of elemental boron standards collected using 0.5 s dwell times during the 2026 experimental run. The peaks on top of the rising edge close to 194 eV are due to oxidation.

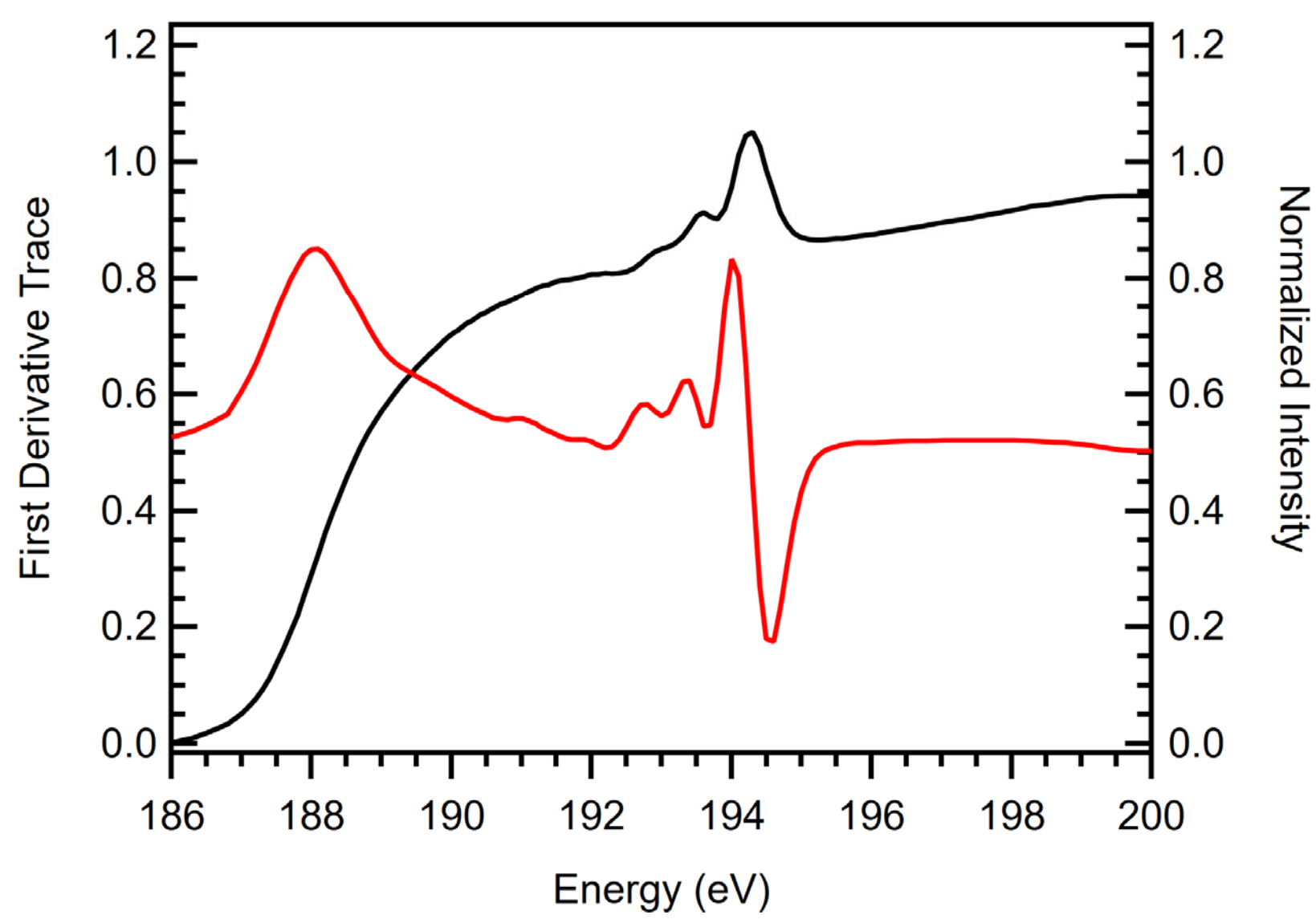

Figure S28. B K-edge XAS spectrum (FLY) of elemental boron standard (black) collected using step scans and 0.5 s dwell times and corresponding first derivative trace (red). The peaks on top of the rising edge close to 194 eV are due to oxidation.

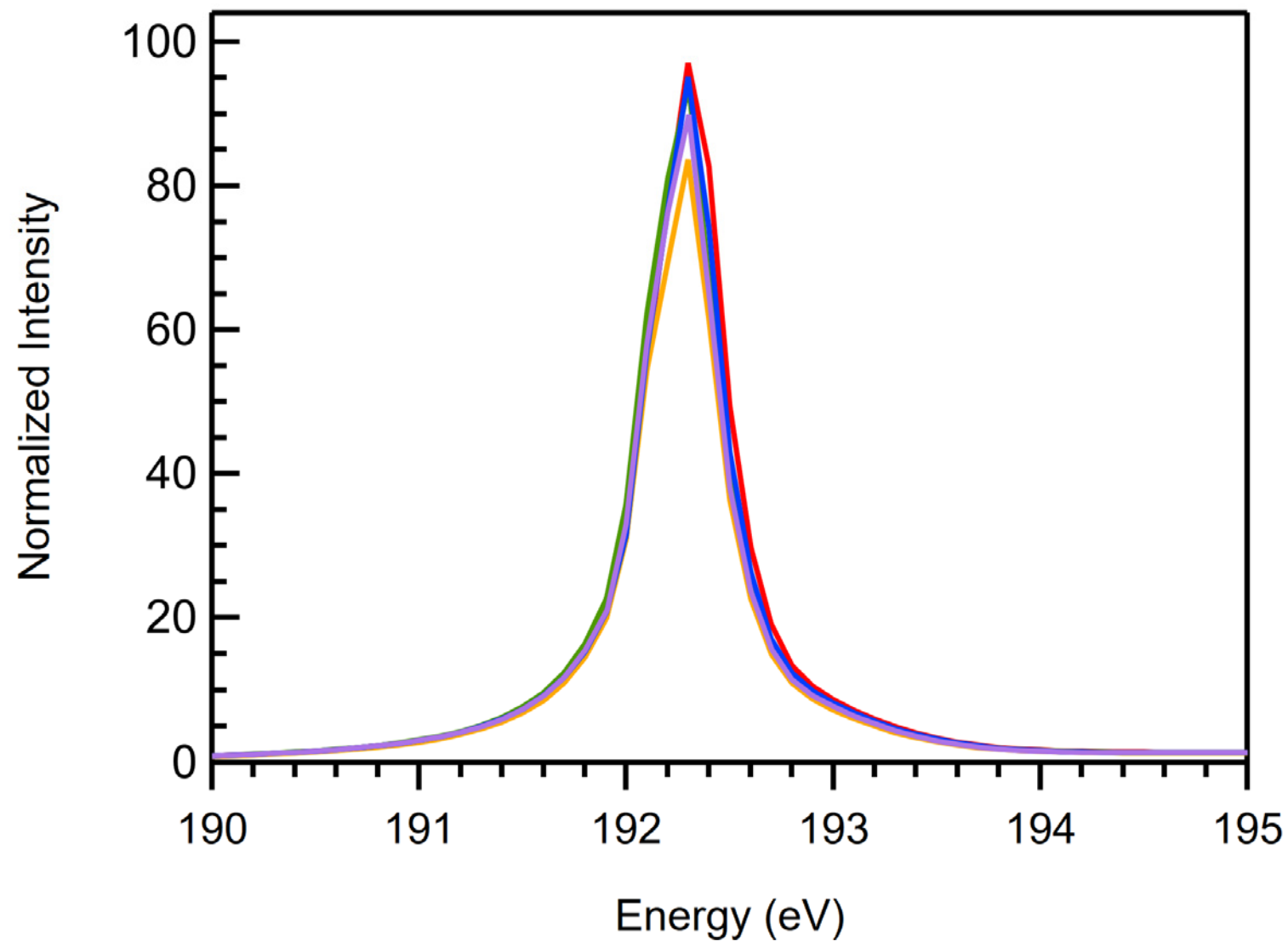

Figure S29. B K-edge XAS step scans (FLY) of a boron nitride (BN) standard collected using 0.5 s dwell times each day during the 2026 experimental run to assess and monitor beam energy stability at VLSPGM.

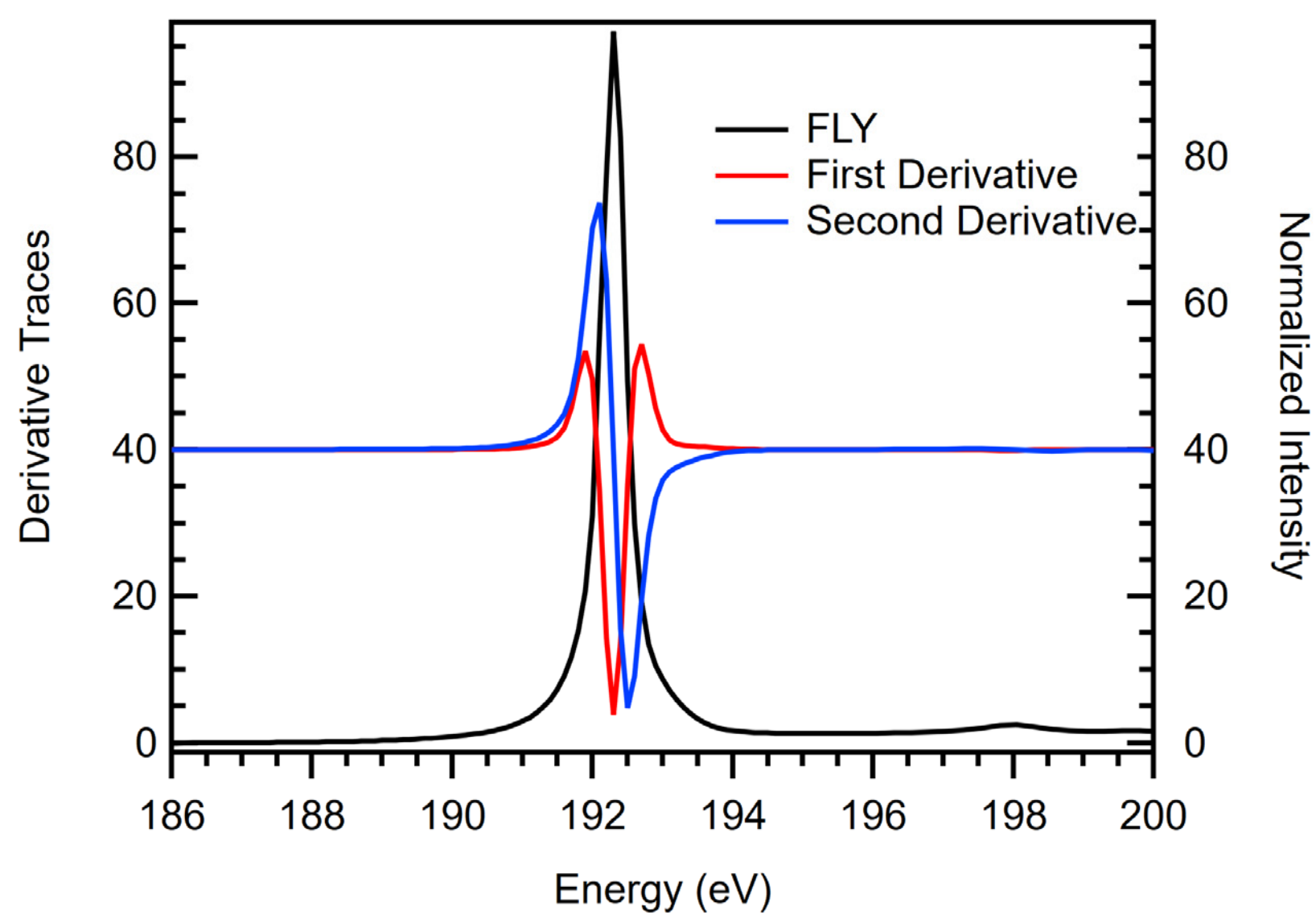

Figure S30. B K-edge XAS step scan spectrum (FLY) of BN standard collected using 0.5 s dwell times and corresponding first and second derivative traces.

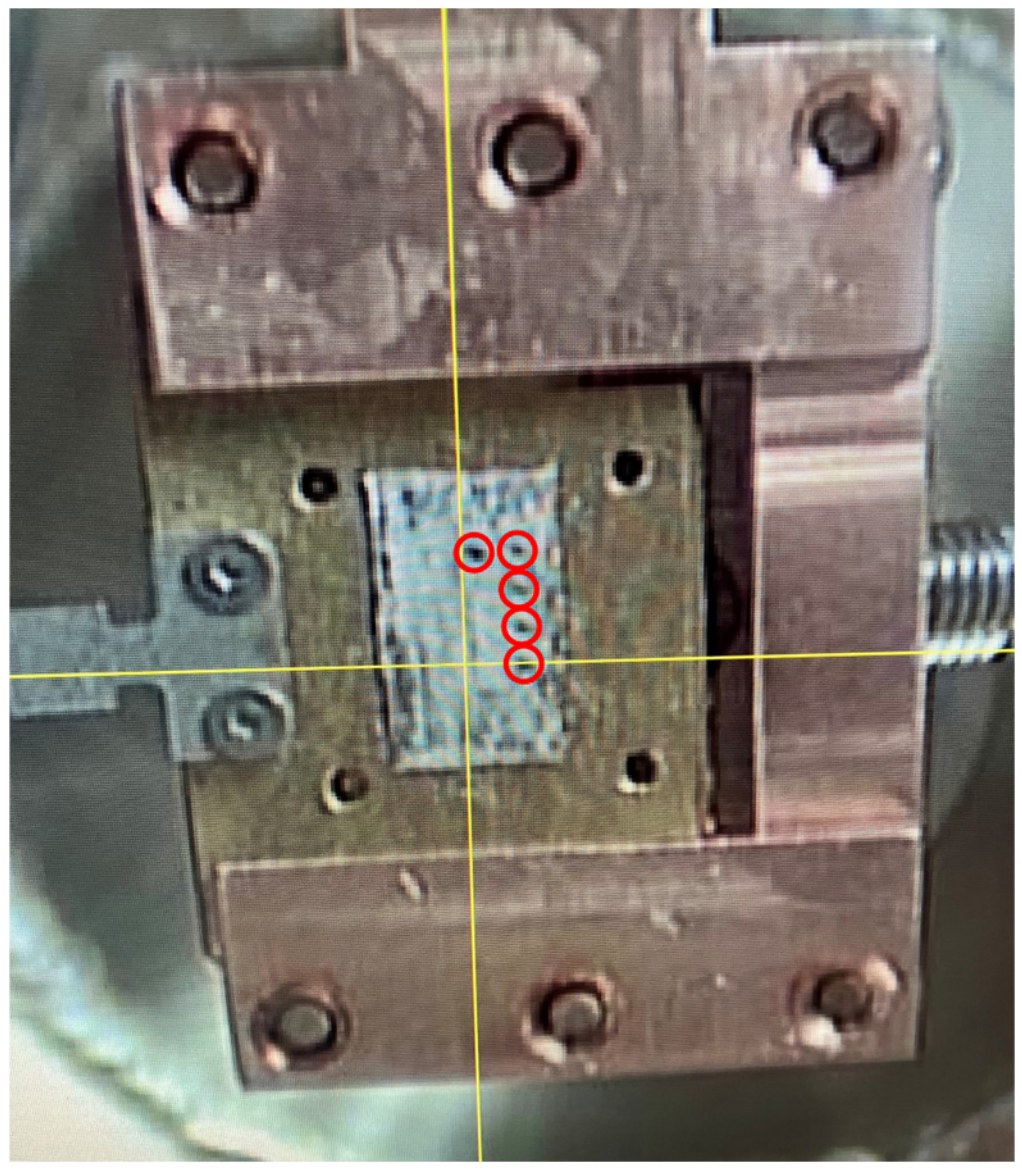

Figure S31. "Holes" observed on a sample of **1** (M = Th) after exposure to the beam. The holes are highlighted with red circles.

Computational Details

Geometry optimizations of **1** ($Th(N^*)_3BH_4$) and **2** ($U(N^*)_3BH_4$) were carried out using density functional theory (DFT) starting from crystal structures obtained from XRD. A singlet and triplet ground state were found for **1** and **2**, respectively. Gas phase geometry optimizations were performed for all structures using the functionals shown in **Figure S32** as implemented in the Turbomole program package V7.8.1.[1,2] The def-TZVP basis set was used for An=Th, U along with the corresponding effective core potential, while the def2-TZVP was used for all other atom types.[17-18] The cartesian gradient was converged to 1 x 10^{-4} a.u., the SCF energy was converged to 1 x 10^{-7} a.u., and the grid size was set to m4.[19] The resolution of identity (RI) approximation was employed for integral evaluation.[20] Geometries and structures were confirmed as minimum by harmonic vibrational analysis. Functionals which were not chosen for further analysis may have small negative modes (less than 100 cm-1). After functional testing (vide infra), the PBE optimized geometries were utilized for further calculations.

Optimized structures were subjected to Time-Dependent Density Functional Theory (TDDFT) calculations as implemented in ORCA 6.1.[21-22] The ZORA Hamiltonian is utilized along with the SARC-ZORA-TZVP basis for An=Th/U with ZORA-def2-TZVP basis sets for all other atoms.[23-25] The Resolution of the Identity Chain of Spheres exchange method (RIJCOSX) was utilized.[26-28] Excitations were restricted to arise from only the alpha or beta B 1s orbital. For computing these transitions, the Lischner group indicates improved accuracy with hybrid functionals.[29] The GGA hybrid DFT functional b3-lyp and the meta-GGA hybrid DFT functional TPSSh were each employed with Grimme's D4 dispersion correction.[30-34]

Kohn-sham density functional calculations were performed on **1** with the 2024 release of Amsterdam Modeling Suite (AMS).[35-36] The computations of the NMR shielding constants (σ) were performed with the NMR module of ADF, using both the scalar relativistic (SR) and spin-orbit (SO) all electron ZORA Hamiltonian.[37-46] Functionals used for the NMR calculations were the non-hybrid functional Perdew-Burke-Ernzerhof (PBE) and the hybrid variant (PBE0) with 25% exact exchange.[47-52] The Slater-type atomic orbital (STO) basis sets of triple-ζ doubly polarized (TZ2P) quality were used for all atoms.[53] The conductor-like screening model (COSMO) was used to describe solvent effects (THF).[54] To quantify the composition of chemical bonds of interest, natural localized molecular orbitals (NLMOs) were attained with the NBO 7.0 program as interfaced with AMS.[55-56] ^{1}H and ^{11}B NMR chemical shifts (δ) are computed with tetramethylsilane (TMS) and boron trifluoride diethyl etherate as the reference, respectively. TMS was optimized at the same level of theory as **1** and **2** using the PBE functional, while BTFE was optimized in ADF at the PBE/TZ2P level of theory. The shielding (σ) from **1** is subtracted from that of the reference in accordance with Equation S1.

$$\delta_{probe} = \sigma_{ref} - \sigma_{probe} \text{ (S1)}$$

Utilizing the CPL module of the AMS, the B-H nuclear spin-spin coupling constant (J) was calculated at the PBE0-SO level of theory.[57-59] The slater type TZ2P-J basis set was employed for all atoms. Quantum Theory of Atoms in Molecules (QTAIM) and Energy Decomposition Analysis (EDA), as implemented in ADF 2024, was performed on **1** and **2** based on the PBE density utilizing the TZ2P basis set.[60-65] For EDA, the anionic fragment consisted of the borohydride (BH_4^-). The cationic fragment contained the remaining molecule, including the Th or U metal. Both fragments and the neutral complex are modeled as closed-shell singlet states in **1**. In **2**, the neutral molecule and cationic fragment are treated as open-shell triplets.

Functional Testing

Bond length errors for **1** and **2** are shown in Figure S32. Dispersion effects played a very small role for most choices of functional. The PBE and B3-LYP functionals performed the best for both interatomic distances, regardless of the use of Grimme's D3 dispersion correction. Therefore, the PBE optimized structures were utilized in future calculations. Functional dependance of the PBE, b3lyp, and b3lyp-d3 geometries was tested as seen in Figure S35.

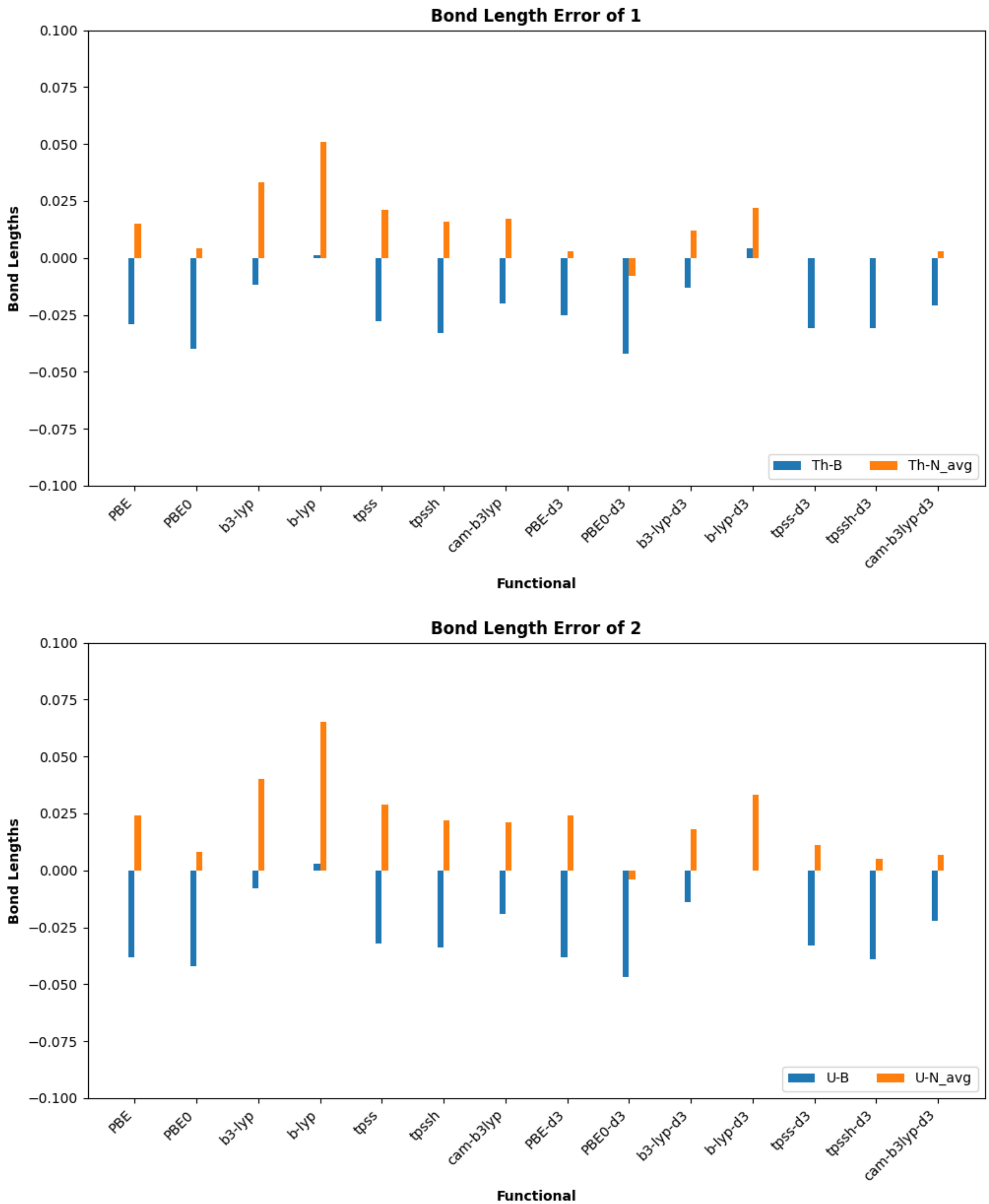

Figure S32. Functional dependence on the geometry optimization of **1** (M = Th; top) and **2** (M = U; bottom) for the interatomic distances of M-N and M-B using triple-ζ quality basis sets.

Geometry and Bonding

Table S2. Selected Bond Lengths and/or Interatomic Distances and the respective Mayer, Gophinatan-Jug, and Nalewajski-Mrozek (N-M) bond orders, variants 1-3, for the PBE optimized geometry of **1** (M = Th).

Atoms	Distance	Mayer	G-J	N-M1	N-M2	N-M3
Th-B	2.6078	0.3624	0.4468	0.5171	1.2792	0.4689
Th-H	2.4231	0.2064	0.1536	0.1761	0.0951	0.1462
Th-H	2.4233	0.2073	0.1537	0.1763	0.0955	0.1463
Th-H	2.4234	0.2063	0.1536	0.1761	0.0958	0.1462
Th-N	2.3210	0.6431	0.8827	1.0900	2.1757	1.0677
Th-N	2.3208	0.6425	0.8831	1.0905	2.1762	1.0682
Th-N	2.3211	0.6432	0.8824	1.0896	2.1757	1.0674
B-H	1.2021	0.7423	0.9537	0.9766	0.9631	1.0241
B-H	1.2405	0.6796	0.8108	0.8245	0.8028	0.8756
B-H	1.2408	0.6789	0.8108	0.8245	0.8026	0.8756
B-H	1.2407	0.6792	0.8109	0.8246	0.8026	0.8758
Si-N	1.7624	0.893	0.9130	1.0142	1.7867	1.0213
Si-N	1.7710	0.8879	0.8950	0.9937	1.7699	1.0011
Si-N	1.7624	0.8918	0.9126	1.0138	1.7873	1.0209
Si-N	1.7710	0.8865	0.8950	0.9938	1.7702	1.0011
Si-N	1.7627	0.8916	0.9129	1.0141	1.7871	1.0213
Si-N	1.7713	0.8881	0.8951	0.9939	1.7701	1.0014

Table S3. Selected Bond Lengths and/or Interatomic Distances and the respective Mayer, Gophinatan-Jug, and Nalewajski-Mrozek (N-M) bond orders, variants 1-3, for the PBE optimized geometry of **2** (M = U).

Atoms	Distance	Mayer	G-J	N-M1	N-M2	N-M3
U-B	2.5117	0.4854	0.5740	0.7577	1.1500	0.7479
U-H	2.3265	0.2718	0.1989	0.2608	0.1972	0.2387
U-H	2.3191	0.2715	0.2020	0.2650	0.2003	0.2424
U-H	2.3266	0.2823	0.2027	0.2659	0.2041	0.2436
U-N	2.2704	0.7093	1.0799	1.4907	2.0193	1.5421
U-N	2.2576	0.7264	1.1043	1.5229	2.0409	1.5731
U-N	2.2685	0.7060	1.0804	1.4911	2.0195	1.5426
B-H	1.2021	0.7218	0.9593	0.9825	0.9721	1.0246
B-H	1.2416	0.6280	0.7609	0.7758	0.7773	0.8053
B-H	1.2414	0.6273	0.7564	0.7715	0.7737	0.8004
B-H	1.2439	0.6210	0.7578	0.7730	0.7735	0.8032
Si-N	1.7757	0.8661	0.8752	0.9574	1.6403	0.9403
Si-N	1.7664	0.8745	0.8932	0.9772	1.6553	0.9599

Si-N	1.7808	0.8596	0.8679	0.9482	1.6226	0.9295
Si-N	1.7697	0.8660	0.8887	0.9712	1.6411	0.9520
Si-N	1.7761	0.8694	0.8740	0.9558	1.6388	0.9388
Si-N	1.7682	0.8666	0.8911	0.9747	1.6528	0.9575

Table S4. QTAIM (3, -1) bond critical points located between atom centers listed in column 1 for complex **1** (M = Th). Topological analysis is based on the PBE density computed in ADF.

Bond	ρ	$\nabla^2\rho$	V(r)	G(r)	E(r)
B-H1	0.170131	-0.34455	-0.27125	0.092558	-0.17869
B-H2	0.148375	-0.16358	-0.22517	0.092136	-0.13303
B-H3	0.148454	-0.16403	-0.22534	0.092168	-0.13318
B-H4	0.148450	-0.16403	-0.22534	0.092170	-0.13318
Th-B	0.046230	0.124054	-0.044534	0.037774	-0.00676
Th-N1	0.092976	0.191401	-0.125527	0.086689	-0.03884
Th-N2	0.093023	0.191482	-0.125626	0.086748	-0.03888
Th-N3	0.092937	0.191547	-0.125463	0.086675	-0.03879

Table S5. QTAIM (3, -1) bond critical points located between atom centers listed in column 1 for complex **2** (M = U). Topological analysis is based on the PBE density computed in ADF.

Bond	ρ	$\nabla^2\rho$	V(r)	G(r)	E(r)
B-H1	0.170556	-0.34771	0.000737	-0.27224	0.092656
B-H2	0.149019	-0.16918	0.083854	-0.22643	0.092068
B-H3	0.148940	-0.16558	0.083325	-0.22652	0.092562
B-H4	0.148022	-0.16380	0.084657	-0.2242	0.091627
U-B	0.055652	0.136271	0.070252	-0.05794	0.046003
U-N1	0.103124	0.221983	0.146496	-0.14873	0.102111
U-N2	0.101528	0.224727	0.246444	-0.14561	0.100896
U-N3	0.101022	0.227696	0.069650	-0.14481	0.100865

Table S6. Energy Decomposition Analysis (EDA) using the PBE functional on the PBE optimized geometries of **1** (M = Th) and **2** (M = U). Energies in kcals/mol.

	INT E	Pauli	Elect	Orb	Disp	%Elec	%Orb	%Disp
**1**	-153.10	112.04	-176.59	-83.02	-5.53	66.6	31.3	2.1
**2**	-152.07	134.20	-189.69	-90.72	-5.86	66.3	31.7	2.0

Table S7. ETS-NOCV analysis results for the interaction of the [BH4]- ligand with the remainder of the molecule (PBE functional). NOCV pair orbital interaction energies are given for the strongest three interactions (all others are smaller than -5 kcal/mol). For each pair, the NOCV eigenvalue, $|v_e|$, which corresponds to the number of electrons transferred is given in parentheses. The total of all orbital interaction energies is also reported. Energies in kcal/mol. Note that **1** (M = Th) is a closed-shell (spin restricted) calculation and therefore the deformation densities are for the total interaction. For **2** (M = U), a spin polarized calculation is performed, and the interactions are decomposed into alpha, beta, and net contributions.

	**1** (M = Th)		**2** (M = U)
		alpha	-9.3 (0.28)
			-10.6 (0.26)
			-11.8 (0.21)
		Total alpha:	-48.9
		beta	-10.1 (0.21)
			-10.1 (0.21)
			-10.9 (0.20)
		Total beta:	-41.5
closed shell	-23.6 (0.42)	*alpha+beta*	-19.4 (0.49)
	-19.1 (0.41)		-20.6 (0.47)
	-19.1 (0.40)		-22.7 (41)
Total	***-82.7***		***-90.3***

Table S8. ETS-NOCV Deformation Densities using the PBE functional on the PBE optimized geometries of **1** (M = Th; left) and **2** (M = U; right). Orbital Interaction Energies (kcals/mol) shown below each. For each pair, the NOCV eigenvalue, $|v_e|$, which corresponds to the number of electrons transferred is given in parentheses. An isosurface of 0.001 a.u. was used. Blue surfaces represent regions of charge depletion and orange represent regions of charge accumulation.

**1 (M = Th)**	**2 (M = U; alpha)**	**2 (M = U; beta)**
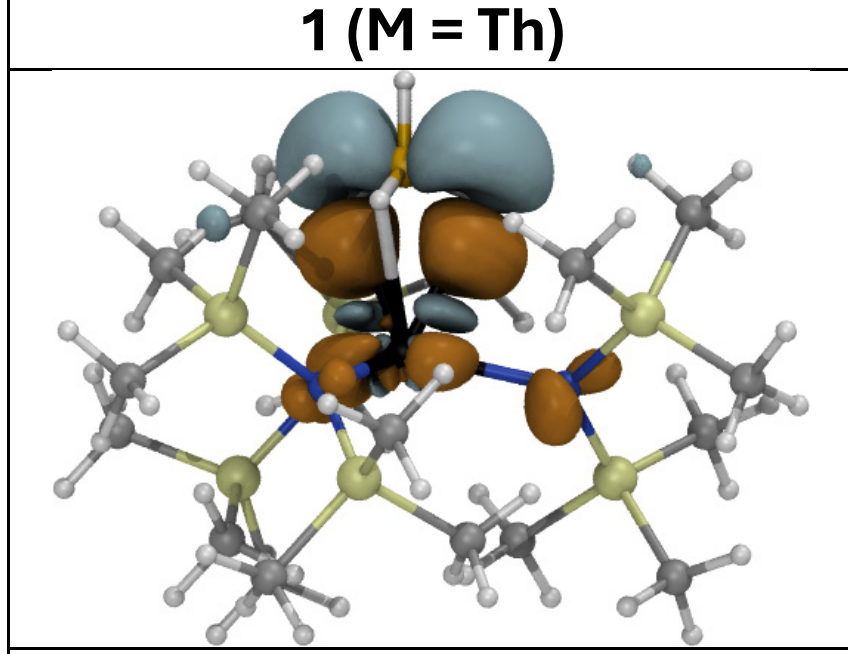	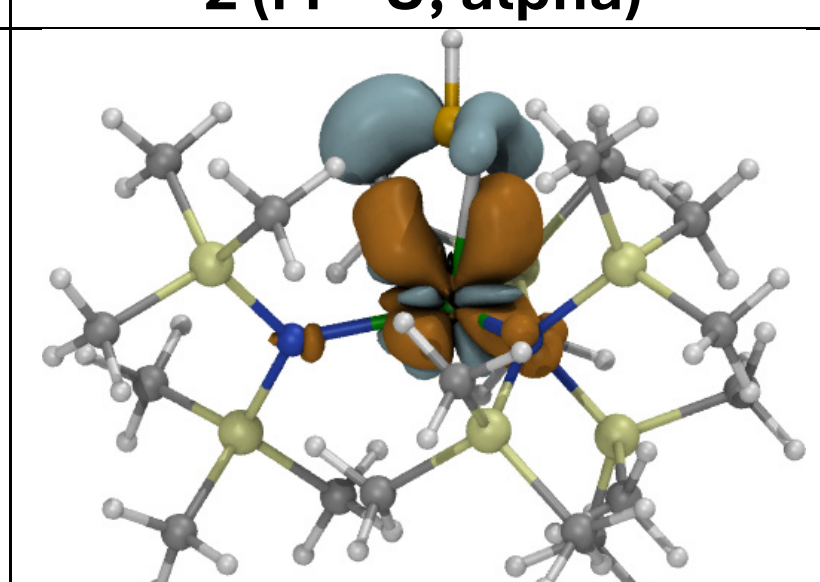	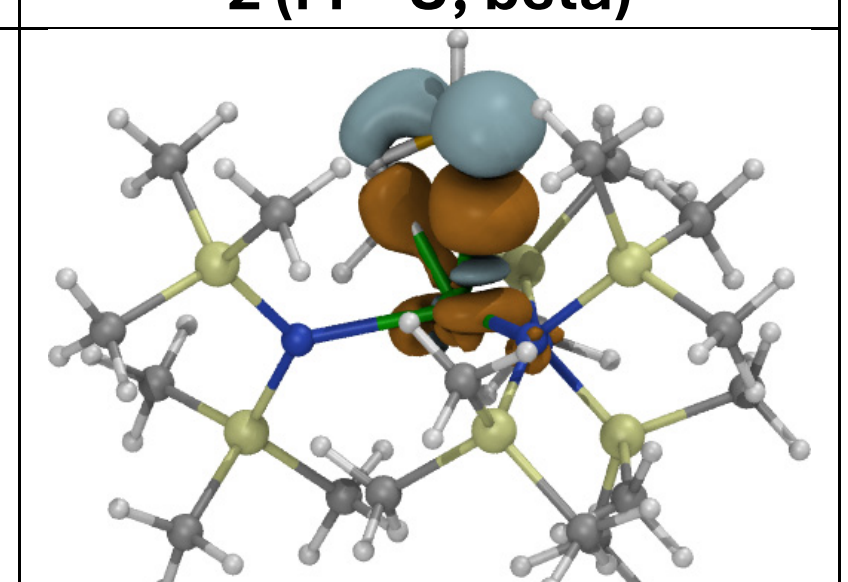
-19.1 kcal/mol (0.41)	-9.3 kcal/mol (0.28)	-10.1 kcal/mol (0.21)

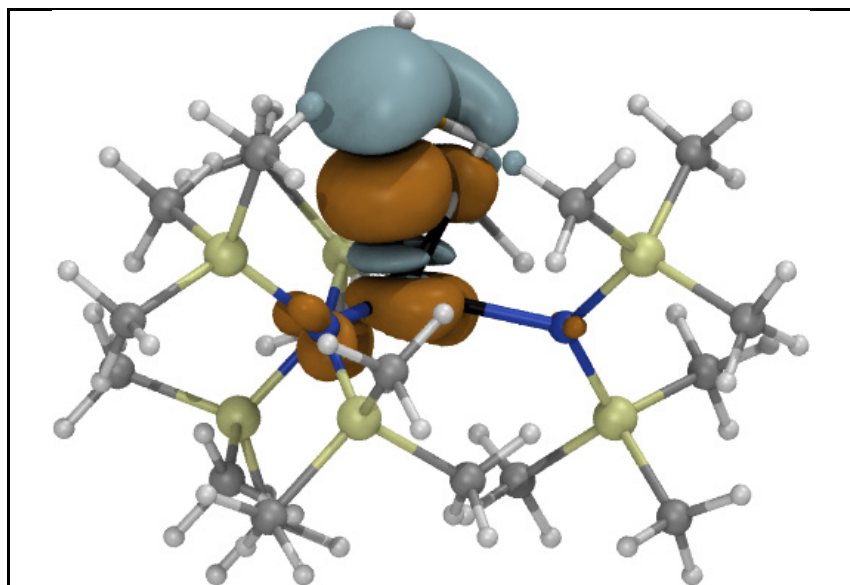	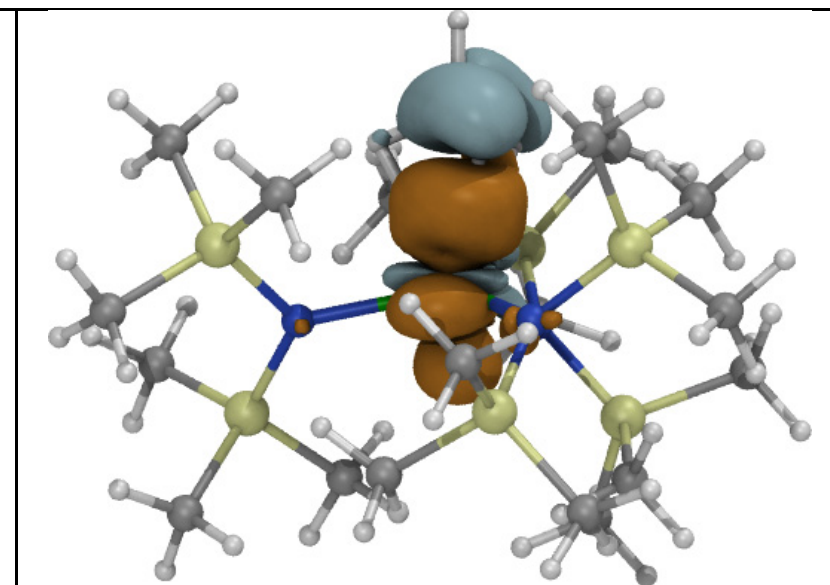	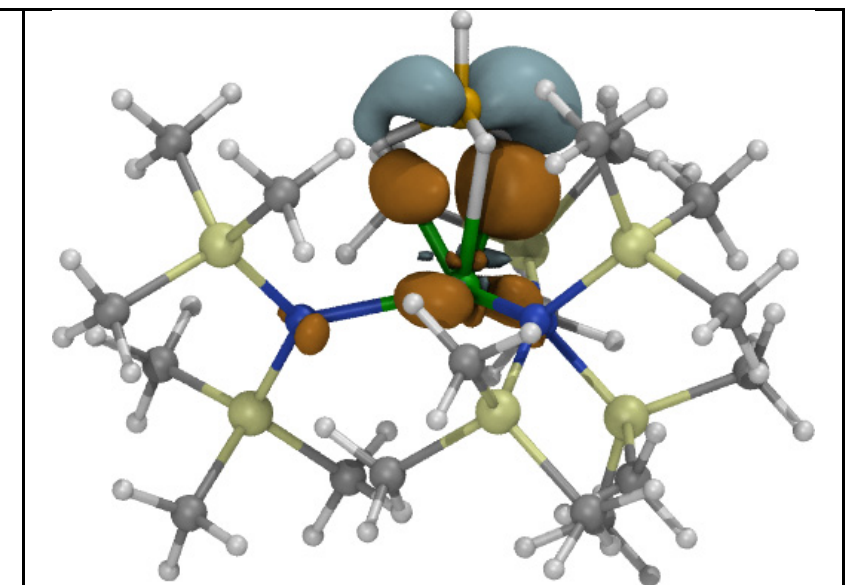
-19.1 kcal/mol (0.40)	-10.6 kcal/mol (0.26)	-10.1 kcal/mol (0.21)
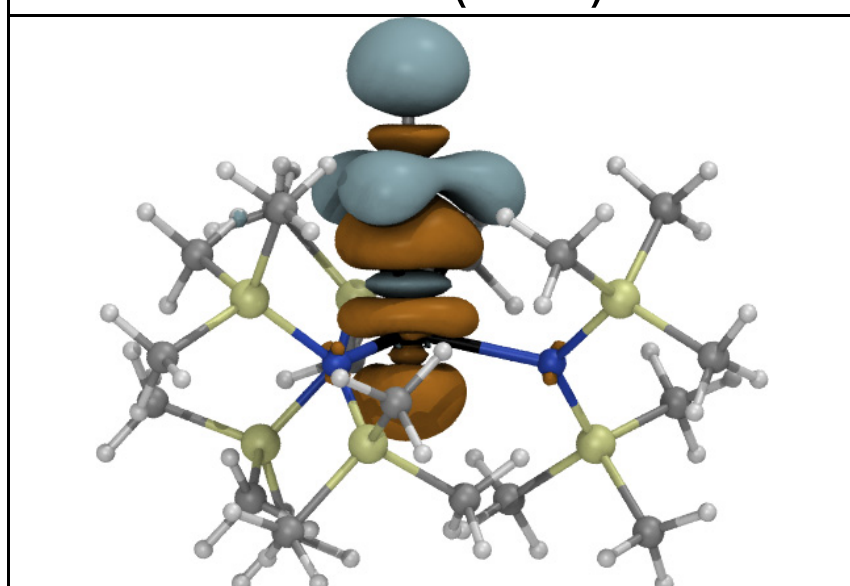	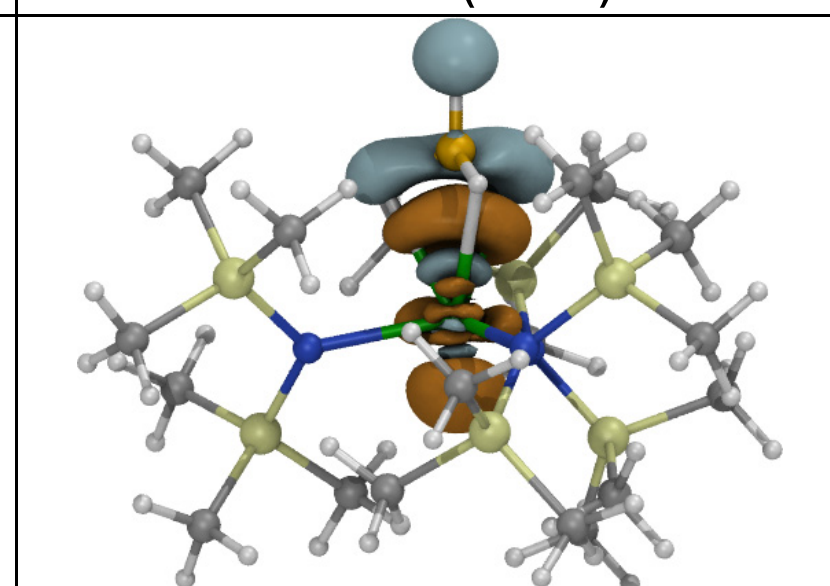	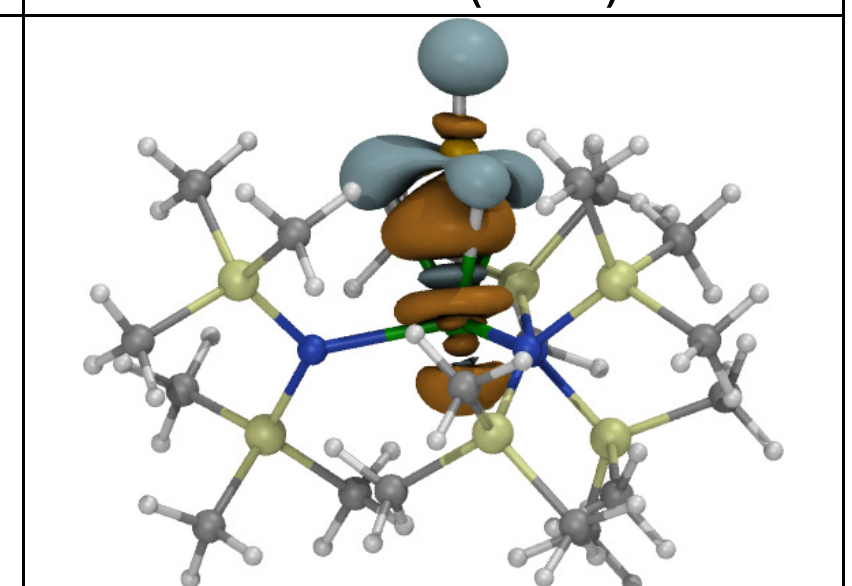
-23.6 kcal/mol (0.42)	-11.8 kcal/mol (0.21)	-10.9 kcal/mol (0.20)

Table S9. Delocalization Indices ($\boldsymbol{\delta}_{DI}$) of selected atom pairs in **1** (M = Th) and **2** (M = U) based on the PBE density in ADF 2024.

	**1 (Th)**	**2 (U)**
An-B	0.1513	0.1854
An-H2	0.1976	0.2433
An-H3	0.1991	0.2438
An-H4	0.1981	0.2388
B-H1	0.6517	0.6513
B-H2	0.5264	0.5193
B-H3	0.5307	0.5136
B-H4	0.5280	0.5160

Time-Dependent Density Functional Theory K-Edge

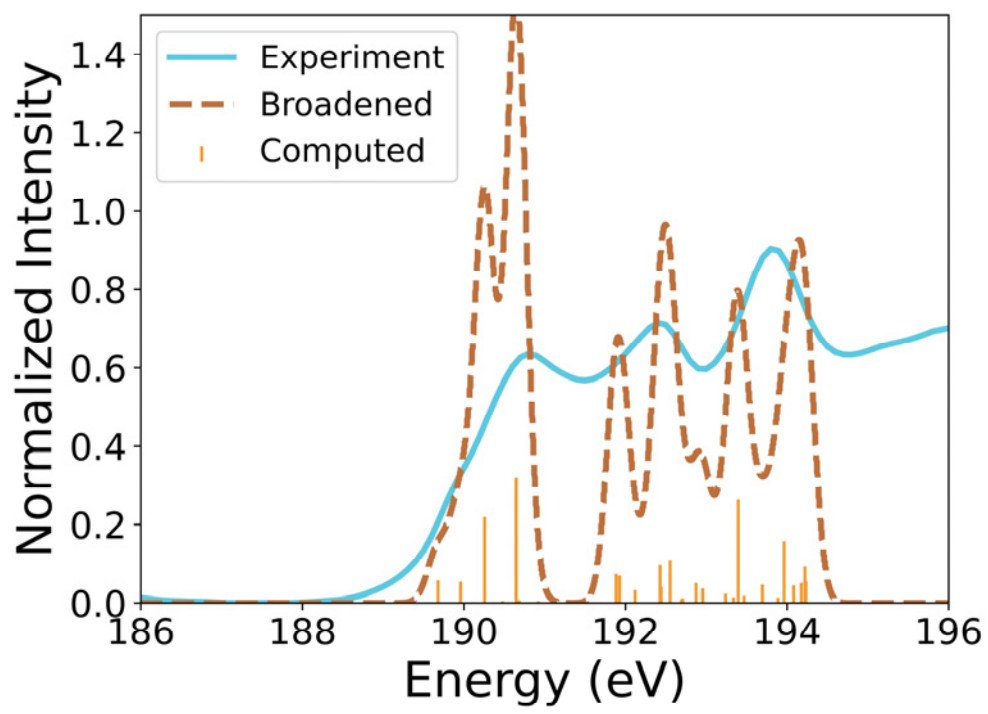

1 (Th); FWHM=0.3 eV

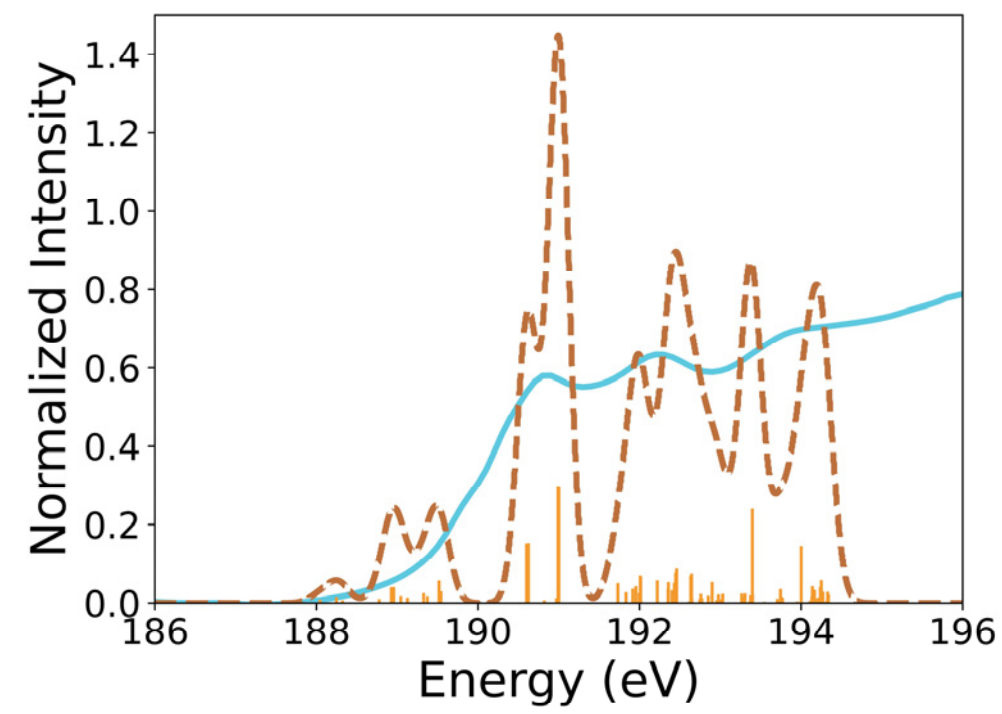

2 (U); FWHM=0.3 eV

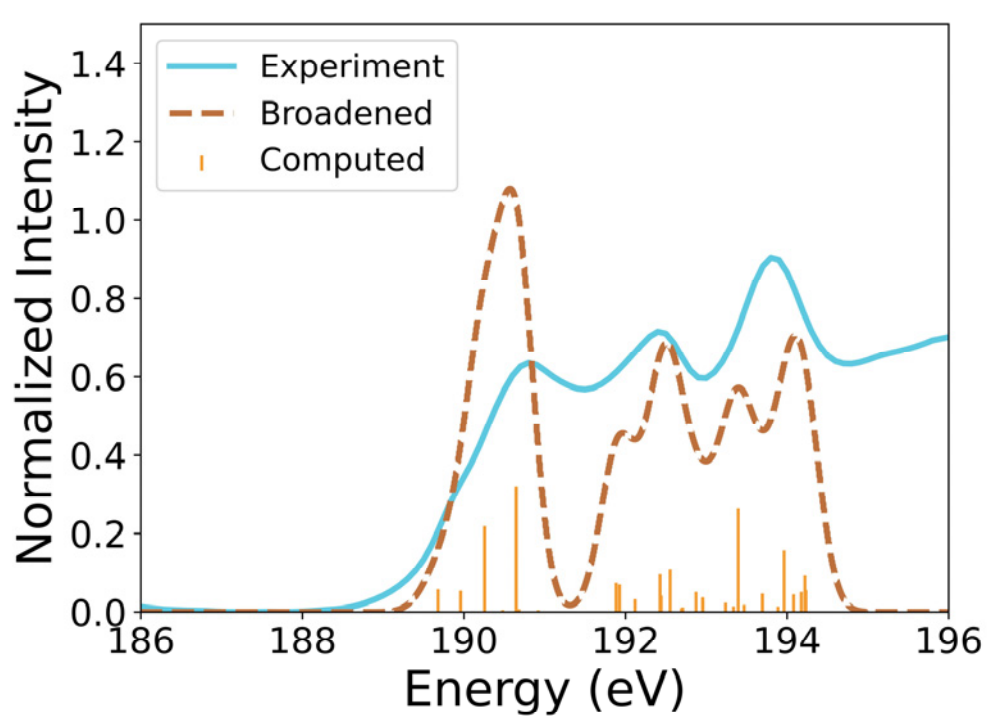

1 (Th); FWHM=0.5 eV

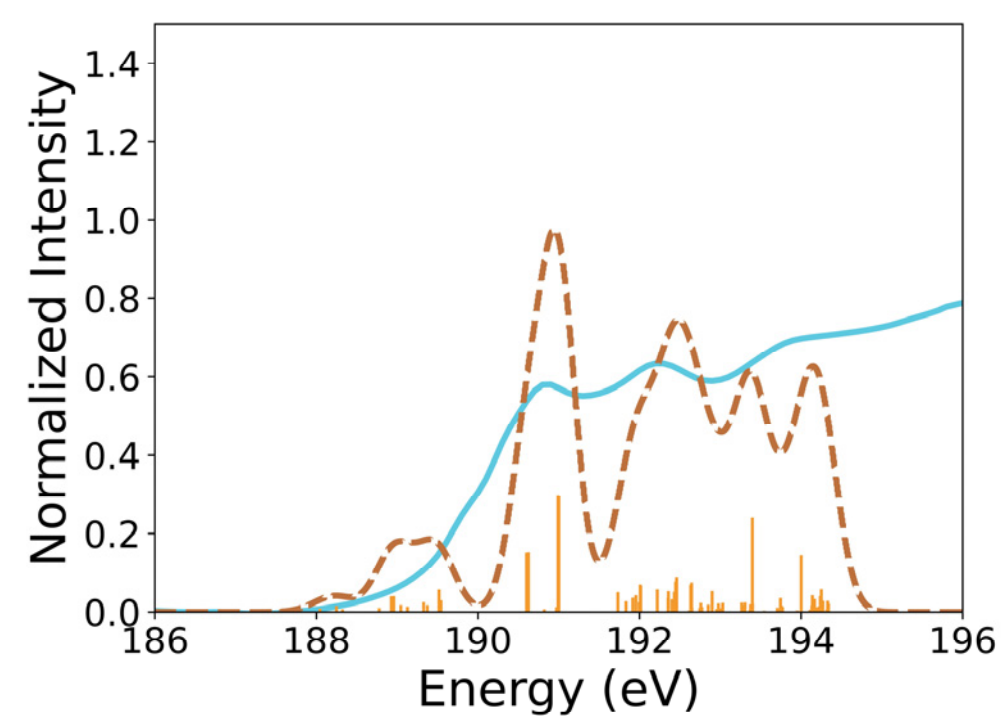

2 (U); FWHM=0.5 eV

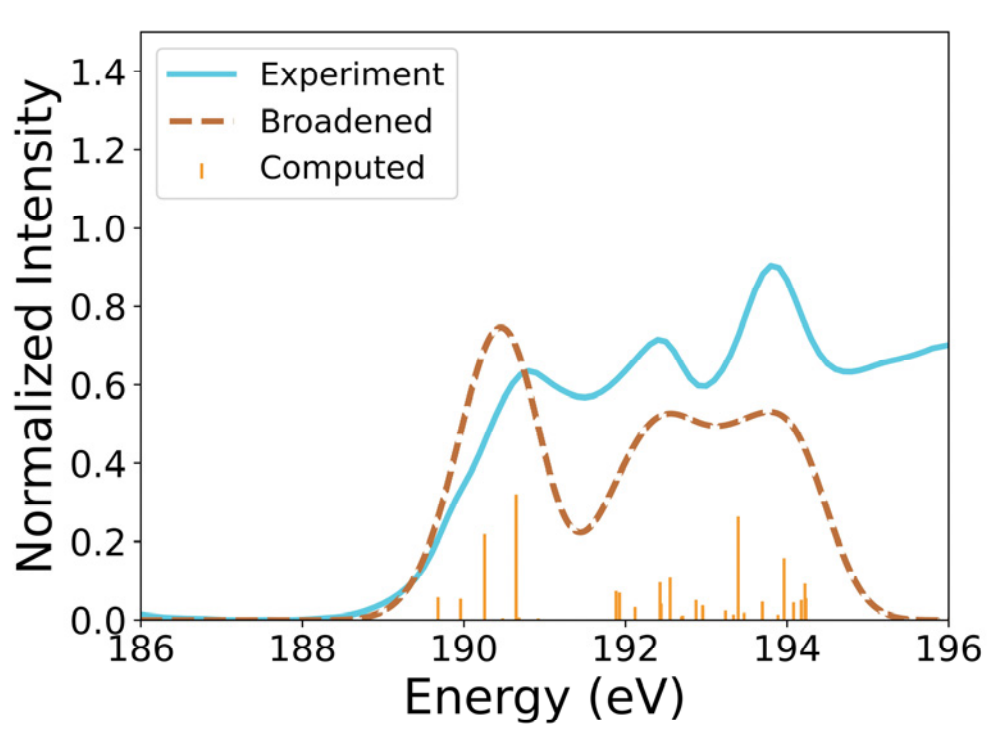

1 (Th); FWHM=1.0 eV

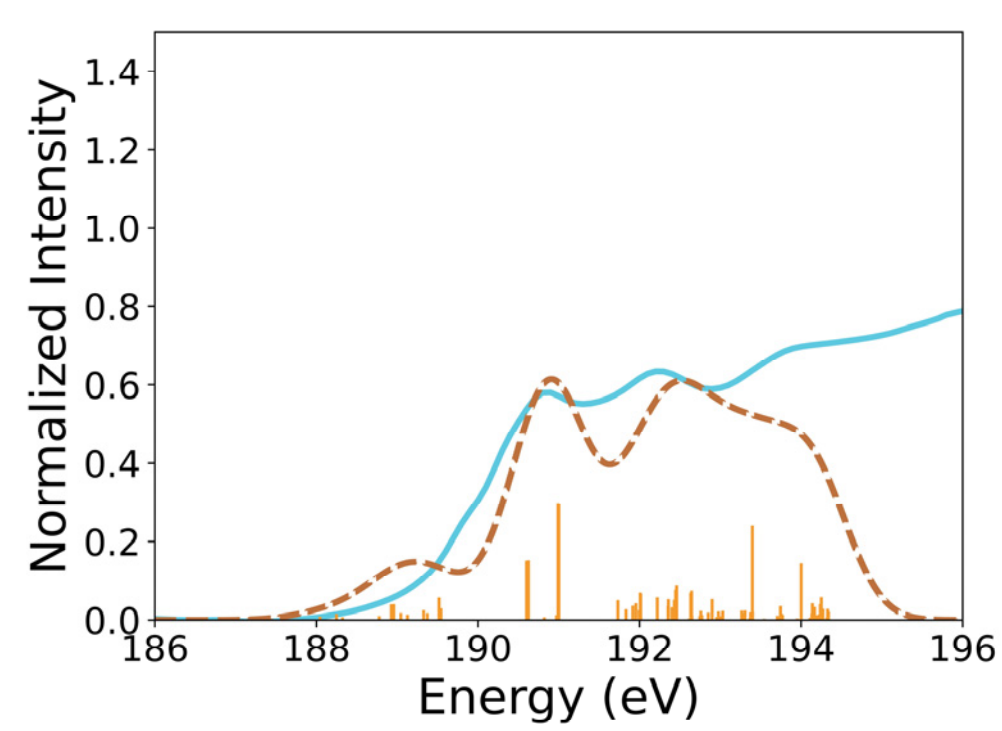

2 (U); FWHM=1.0 eV

Figure S33. TDDFT computed spectra of **1** (M = Th; left) and **2** (M = U; right) compared with the experimental spectra at the B3-LYP-d4 level of theory on PBE optimized geometry. Theory shifted by 9.3 eV. Broadening full width half maximum (FWHM) values of 0.3, 0.5, and 1.0 eV (top, middle, bottom).

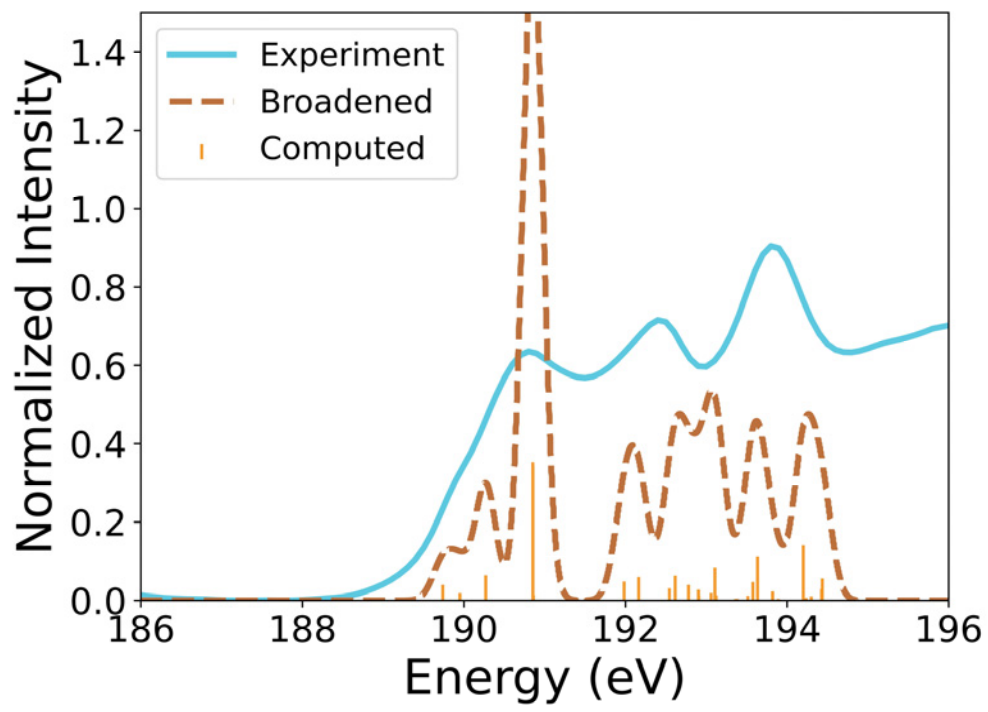

1 (Th); FWHM=0.3 eV

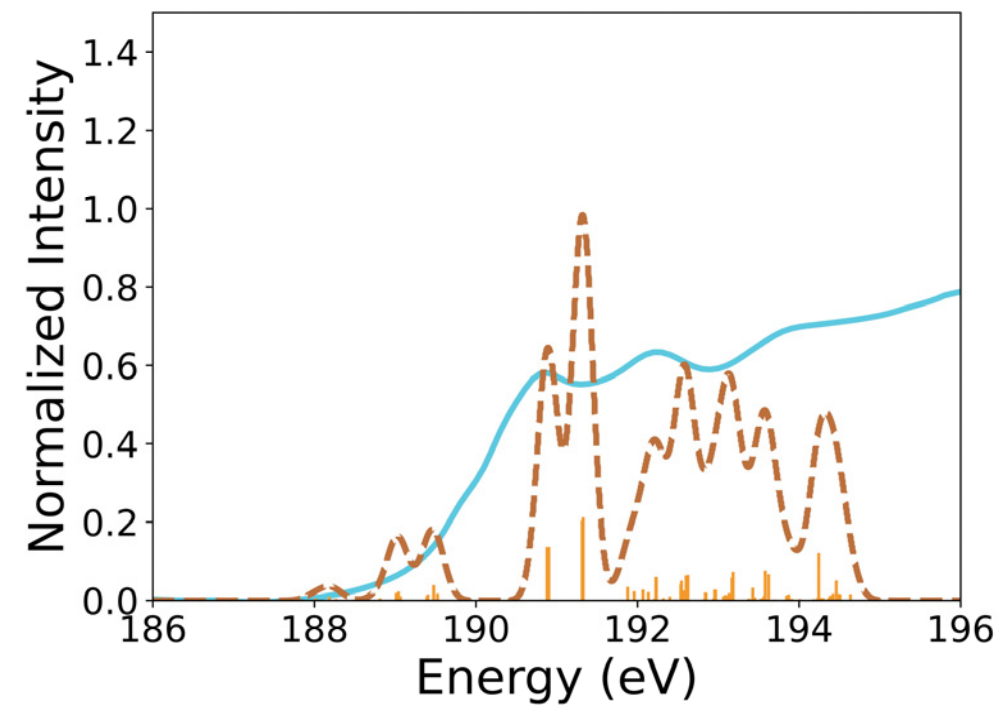

2 (U); FWHM=0.3 eV

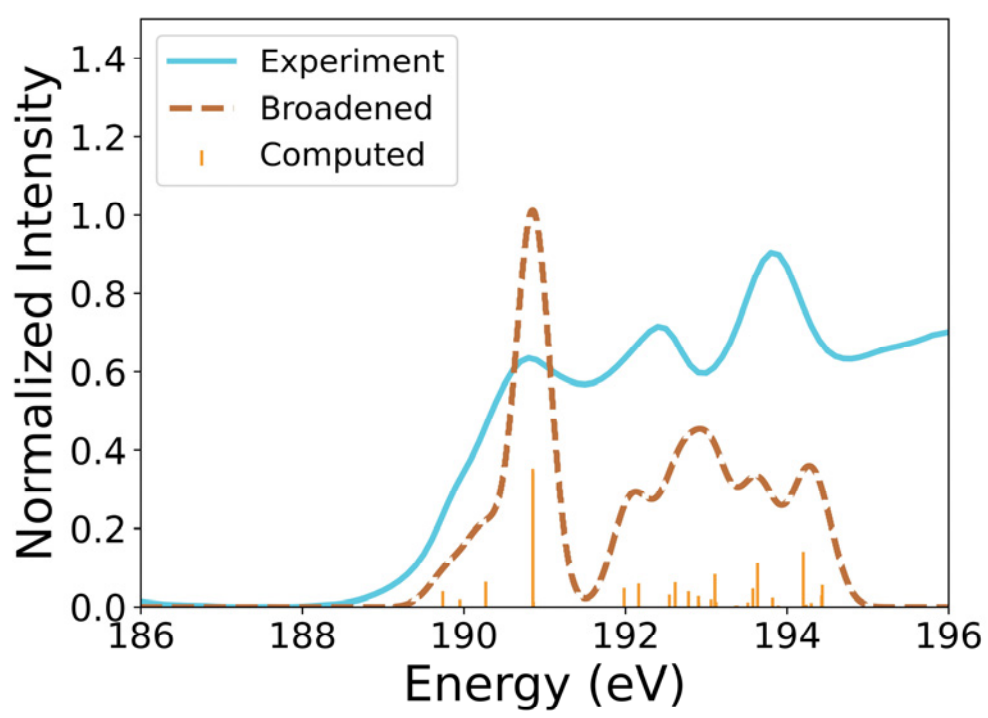

1 (Th); FWHM=0.5 eV

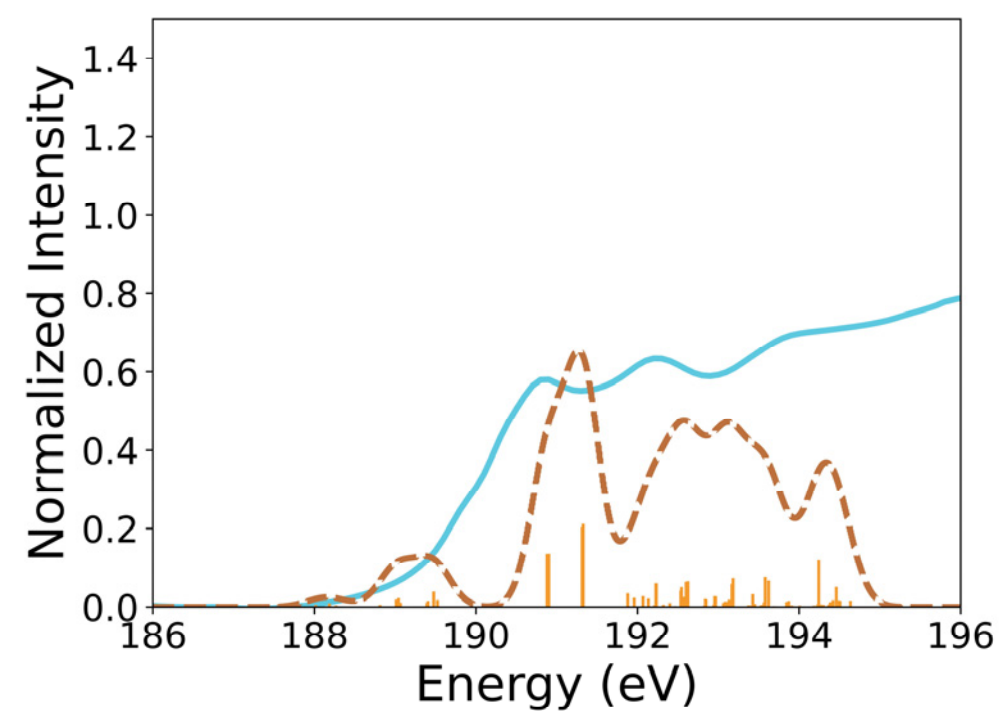

2 (U); FWHM=0.5 eV

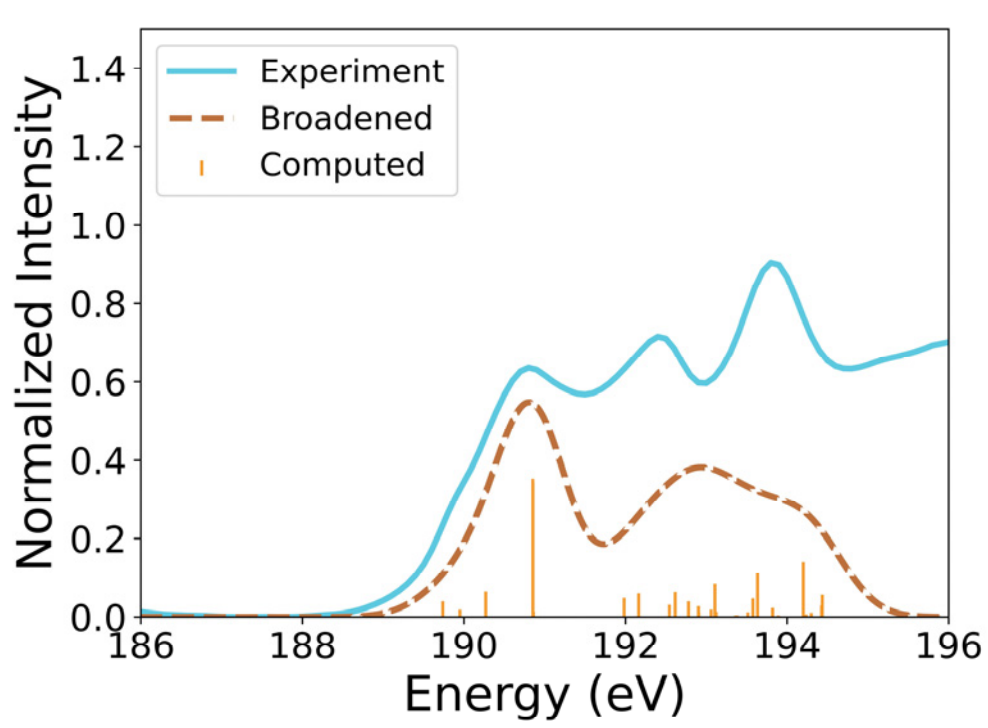

1 (Th); FWHM=1.0 eV

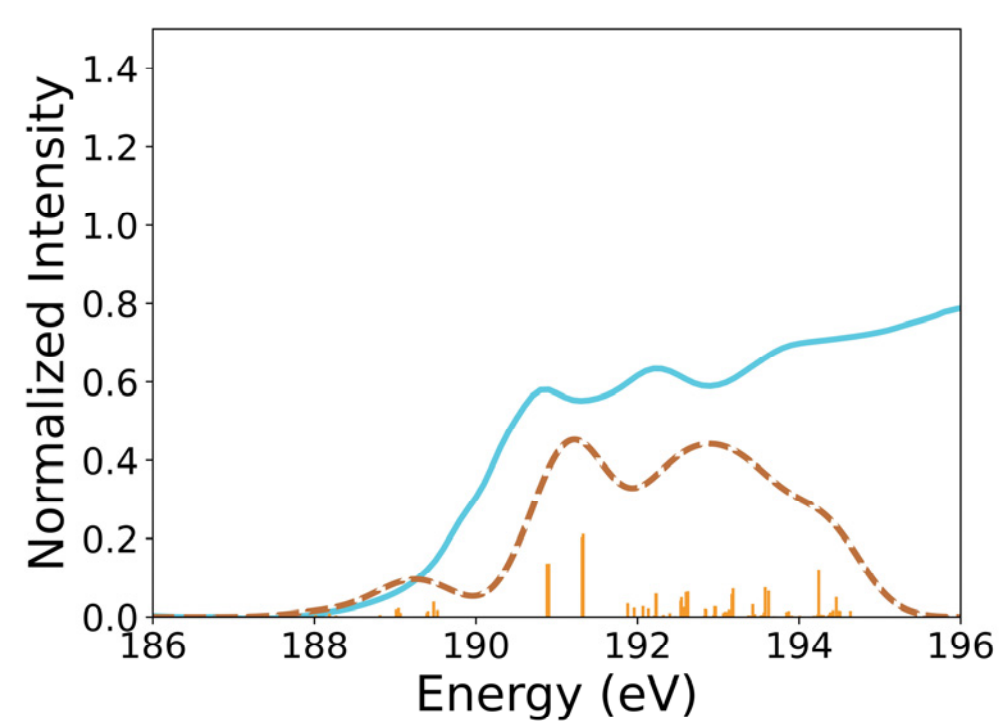

2 (U); FWHM=1.0 eV

Figure S34. TDDFT computed spectra of **1** (M = Th; left) and **2** (M = U; right) compared with the experimental spectra at the TPSSh-d4 level of theory on PBE optimized geometry. Theory shifted by 10.6 eV. Broadening full width half maximum (FWHM) values of 0.3, 0.5, and 1.0 eV (top, middle, bottom).

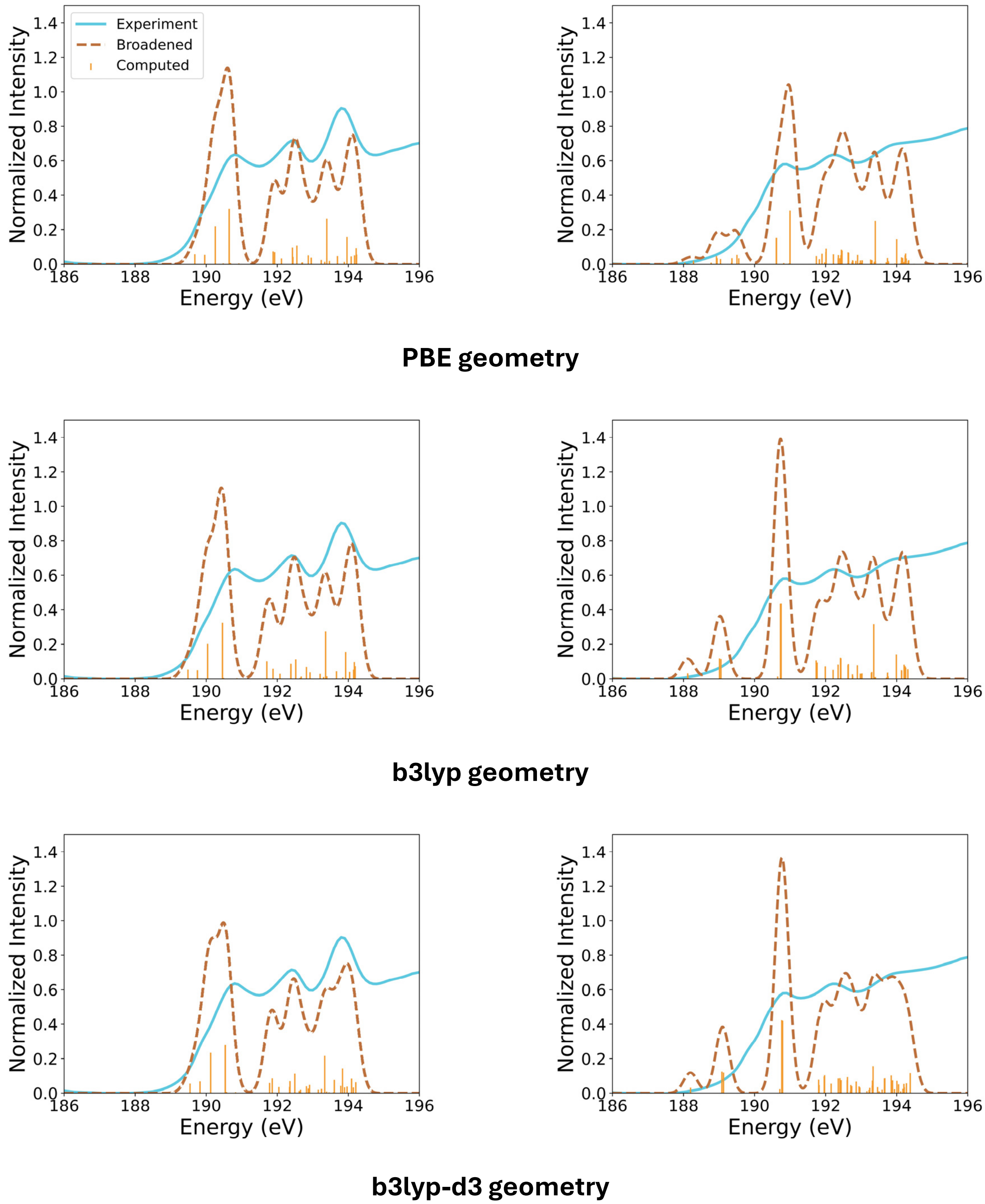

Figure S35. TDDFT computed spectra of **1** (M = Th; left) and **2** (M = U; right) compared with the experimental spectra at the b3lyp-d4 level of theory on three different geometries (PBE, b3lyp, and b3lyp-d3). Theory shifted by 9.3 eV. Broadening full width half maximum (FWHM) values of 0.45 eV.

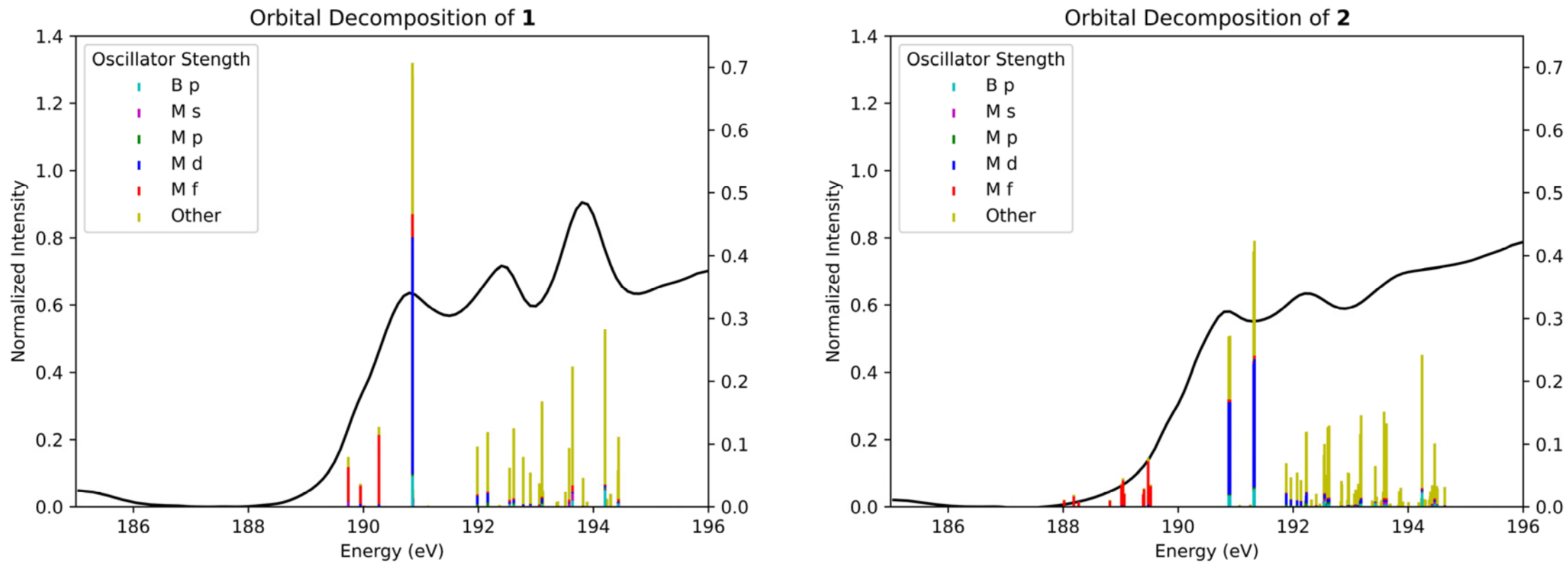

TPSSh-d4 on PBE geometry

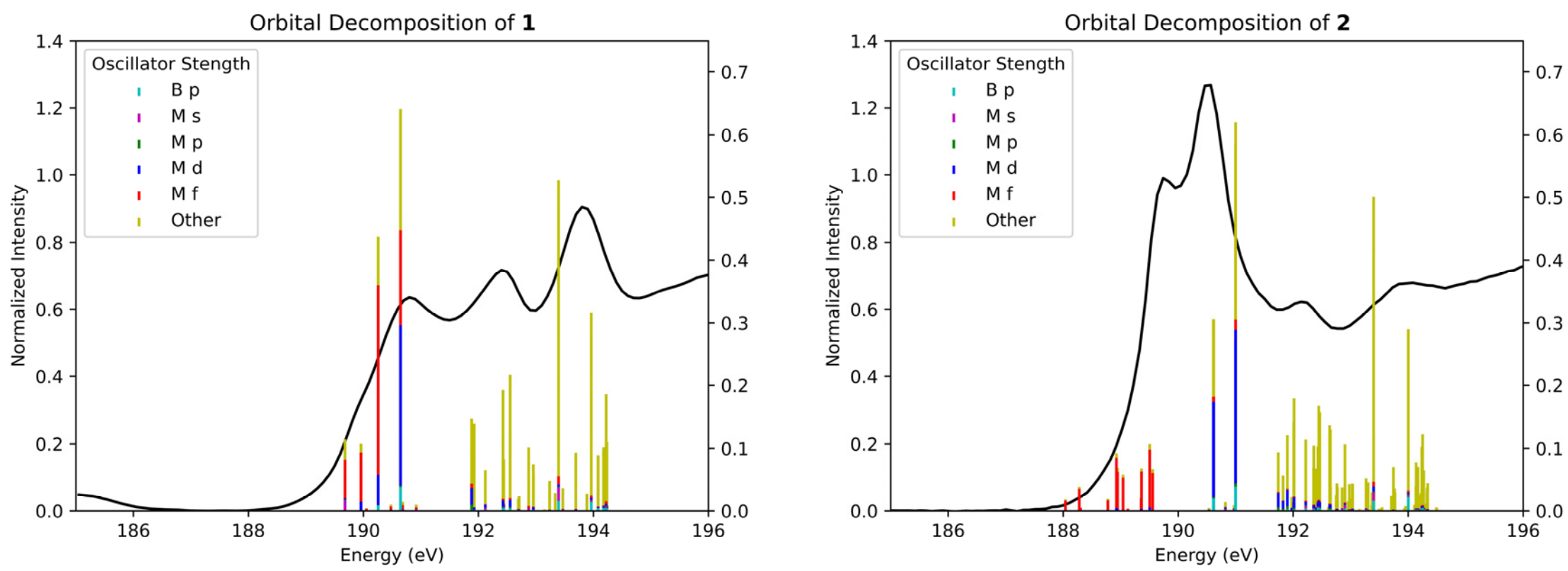

b3lyp-d4 on PBE geometry

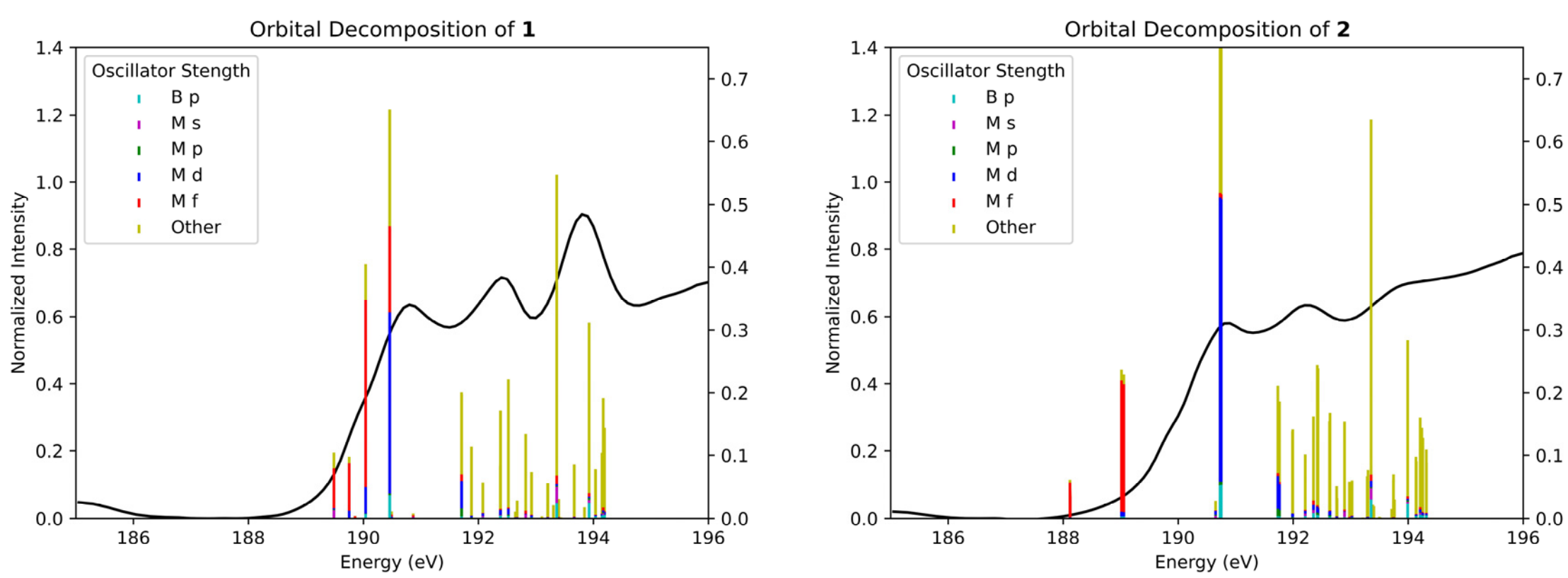

b3lyp-d4 on b3lyp geometry

Figure S36. Contribution of metal orbitals and boron p character as percent contribution to the computed oscillator strength for **1** (M = Th; left) and **2** (M = U; right) on the PBE optimized structure. Computed spectra (TPSSh-d4, top; b3lyp-d4, bottom) are shifted by 10.6 and 9.3 eV, respectively. Experimental spectrum shown in black.

Table S10. Energies (eV) and Oscillator Strengths of the transitions corresponding to each state in the TDDFT b3lyp-d4 calculations on the PBE geometry of **1** (M = Th). The summed Reduced Löwdin Orbital Character of the orbitals in each transition comprising each state is expressed scaled by the percent contribution to each state by the intensity of the transition. The shift of 9.3 eV is applied.

State	Energy	Oscillator Strength	B	Th			
			p	s	p	d	f
1	188.109	1.77E-11	0.4	0.3	0.0	1.7	92.3
2	188.1104	1.02E-10	0.5	4.1	0.2	0.8	83.2
3	188,1132	6.51E-11	0.4	0.3	0.0	1.8	90.0
4	188.1179	0.00061	0.4	0.0	0.0	1.8	91.0
5	188.1210	0.000461	0.4	1.7	0.1	1.5	88.8
6	188.1231	0.000387	0.5	2.9	0.1	1.2	86.0
7	188.7993	6.86E-10	0.0	0.3	0.1	1.3	89.1
8	188.7995	6.48E-06	0.0	0.3	0.1	1.3	89.2
9	188.9788	8.24E-12	1.4	0.0	0.0	3.0	87.2
10	189.0131	0.002365	1.4	0.0	0.0	3.1	88.5
11	189.0210	8.09E-10	1.5	0.0	0.0	4.0	87.6
12	189.0544	0.002292	1.4	0.0	0.0	3.0	88.8
13	190.5991	1.91E-11	5.4	1.3	1.2	48.0	3.2
14	190.6092	1.87E-11	4.6	4.0	1.9	43.5	3.2
15	190.6405	1.62E-11	1.9	13.1	4.0	28.4	2.1
16	190.6500	0.000277	0.2	15.2	6.2	21.9	2.6
17	190.7288	0.008694	6.0	0.0	0.6	52.2	0.8
18	190.7477	0.008716	6.1	0.0	0.6	51.7	0.9
19	190.8785	9E-14	0.3	28.5	3.4	11.5	2.2
20	190.9253	1.87E-06	0.4	33.0	2.8	9.0	2.2
21	191.7027	3.77E-12	2.0	0.0	5.4	26.2	2.2
22	191.7331	3.51E-11	2.0	0.0	5.4	23.8	1.9
23	191.7339	0.00211	1.8	0.0	5.5	24.6	2.2
24	191.7609	0.001862	1.7	0.0	5.4	22.0	1.9
25	191.9696	0	1.0	0.0	0.4	3.1	0.1
26	191.9730	1E-14	1.0	0.0	0.5	2.6	0.1
27	191.9884	0.001386	1.1	0.0	0.7	3.8	0.2
28	191.9888	1.52E-11	4.0	5.2	0.4	3.6	1.4
29	191.9919	0.001418	1.1	0.0	0.7	3.5	0.2
30	192.2104	0.001014	3.8	3.7	0.3	4.0	1.6
31	192.3152	4E-14	5.1	2.8	0.1	4.7	4.1
32	192.3520	0.001626	5.3	2.9	0.1	5.0	4.2
33	192.3837	1.32E-12	2.3	0.0	1.8	5.3	1.0
34	192.3959	1.47E-12	2.0	0.1	1.5	4.7	1.0
35	192.4185	0.00244	2.3	0.0	1.5	3.8	0.9

36	192.4308	0.002386	2.0	0.1	1.1	3.3	0.9
37	192.6120	0	1.7	0.0	1.0	3.6	0.6
38	192.6185	0	1.8	0.0	1.1	3.8	0.6
39	192.6312	0.001549	1.8	0.0	1.1	3.9	0.7
40	192.6395	0.001683	1.8	0.0	1.2	3.9	0.6
41	192.6522	8.5E-13	5.4	2.4	0.7	3.7	0.9
42	192.7490	7E-14	1.7	0.0	1.2	3.1	0.8
43	192.7548	0.00051	2.0	0.2	1.1	3.2	0.7
44	192.7550	5.73E-11	1.7	0.0	1.1	3.0	0.5
45	192.7578	0.000287	3.7	1.0	0.9	3.5	0.6
46	192.7625	0.000321	3.4	0.8	1.0	3.5	0.6
47	192.8488	1.5E-13	2.0	4.2	0.2	1.0	1.8
48	192.8922	0.001547	1.5	5.3	0.2	0.4	1.7
49	192.9648	9E-14	0.8	3.0	0.1	0.5	1.5
50	192.9764	0.000583	0.6	2.0	0.0	0.7	1.5
51	193.0087	0	2.3	0.0	0.6	3.0	0.8
52	193.0150	8.6E-13	2.5	0.0	0.7	3.3	0.8
53	193.0163	0.000569	2.4	0.0	0.6	3.1	0.8
54	193.0228	0.000601	2.6	0.0	0.6	3.3	0.8
55	193.1453	1E-14	7.3	2.9	0.4	1.9	0.7
56	193.2090	1.72E-05	9.2	3.7	0.3	1.4	0.7
57	193.2600	2.6E-13	4.4	2.7	0.2	1.4	1.4
58	193.2791	3.1E-13	1.4	0.2	0.2	1.5	0.5
59	193.2883	0.000668	1.3	0.1	0.2	1.5	0.5
60	193.2906	1.39E-12	1.6	0.2	0.3	1.5	0.5
61	193.3004	0.000767	1.5	0.0	0.3	1.5	0.5
62	193.3545	0.006355	4.7	2.8	0.3	1.6	1.5
63	193.3798	5E-14	0.6	0.0	0.1	1.5	0.5
64	193.3822	0.000218	0.6	0.0	0.1	1.4	0.4
65	193.4008	2.1E-13	0.5	0.0	0.1	1.2	0.4
66	193.4033	0.000195	0.6	0.0	0.1	1.2	0.4
67	193.4524	1E-14	1.4	1.8	0.0	1.5	1.2
68	193.4730	8.1E-06	0.9	1.6	0.0	1.7	1.3
69	193.4970	1E-14	4.1	0.8	0.4	2.5	0.8
70	193.4987	2.83E-05	4.0	0.7	0.4	2.5	0.8
71	193.5124	3E-14	4.1	2.0	0.3	2.0	1.2
72	193.5161	1.84E-05	4.2	2.2	0.3	2.0	1.3
73	193.6493	2E-14	1.3	0.9	0.2	0.5	1.3
74	193.7140	0.000151	0.3	0.6	0.3	0.3	1.0
75	193.7232	4.4E-13	0.5	0.2	0.3	1.1	0.7
76	193.7306	1E-13	0.6	0.1	0.3	1.4	0.6
77	193.7415	0.000608	0.6	0.1	0.2	1.2	0.8
78	193.7442	0.000699	0.5	0.2	0.2	1.3	0.8
79	193.7526	2.7E-13	0.8	0.5	0.3	0.7	1.1

80	193.7591	0.000301	0.8	0.5	0.2	0.8	1.2
81	193.9137	0	1.1	0.9	0.2	0.7	0.8
82	193.9230	1.08E-05	0.2	0.9	0.1	0.7	0.8
83	193.9414	1E-14	5.9	1.2	0.6	0.9	1.3
84	193.9909	0.002833	8.2	1.1	0.8	0.9	1.3
85	194.1153	0	4.1	0.0	0.2	1.7	0.8
86	194.1251	5E-14	4.0	0.2	0.3	1.9	0.8
87	194.1355	0.000976	4.0	0.0	0.2	1.6	0.8
88	194.1415	0.000906	4.1	0.2	0.3	2.3	0.9
89	194.1800	3.3E-13	4.0	1.7	0.6	2.3	2.2
90	194.2087	0.001608	3.8	1.7	0.6	2.7	2.3
91	194.2134	1.77E-12	3.4	0.2	0.4	2.2	1.0
92	194.2345	1.02E-12	3.5	0.0	0.4	2.0	0.8
93	194.2369	0.001442	3.3	0.3	0.4	2.3	1.1
94	194.2539	0.00128	3.5	0.1	0.5	2.3	0.9
95	194.3021	3E-14	3.5	0.2	0.5	2.5	1.1
96	194.3066	0	2.3	0.1	0.6	2.6	0.8
97	194.3152	0.001091	4.5	0.1	0.4	2.2	1.0
98	194.3162	0.000877	3.3	0.1	0.5	2.5	0.9
99	194.4865	0	3.3	0.1	0.2	1.5	1.0
100	194.4945	2E-14	2.3	0.2	0.2	1.3	1.1

Table S11. Energies (eV) and Oscillator Strengths of the transitions corresponding to each state in the TDDFT b3lyp-d4 calculations on the PBE geometry of **2** (M = U). The summed Reduced Löwdin Orbital Character of the orbitals in each transition comprising each state is expressed scaled by the percent contribution to each state by the intensity of the transition. The shift of 9.3 eV is applied.

State	Energy	Oscillator Strength	B	U			
			p	s	p	d	f
1	189.6082	0	0.7	13.7	1.4	1.7	55.1
2	189.6794	0.001146	0.7	13.9	1.3	1.4	53.9
3	189.9353	0	0.8	0.0	0.0	15.4	71.2
4	189.9363	0	0.8	0.0	0.0	15.4	71.2
5	189.9577	0.001075	0.4	0.0	0.0	13.0	73.6
6	189.9585	0.001062	0.4	0.0	0.0	13.0	73.7
7	190.0492	0	0.1	1.1	0.9	17.1	68.9
8	190.0529	3.99E-05	0.1	1.1	0.9	17.1	68.5
9	190.1822	0	2.6	0.0	0.1	16.4	60.1
10	190.1829	0	2.6	0.0	0.1	16.3	60.3
11	190.2549	0.004375	2.0	0.0	0.1	11.0	69.1
12	190.2555	0.004373	2.0	0.0	0.1	11.0	69.2
13	190.4728	0	0.2	10.3	2.4	5.8	50.5
14	190.4787	0.0001	0.2	9.8	2.4	5.9	51.4
15	190.5681	0	4.6	0.0	0.3	31.0	36.9
16	190.5700	0	4.6	0.0	0.3	31.1	36.8
17	190.6452	0.006409	5.9	0.0	0.4	40.0	23.7
18	190.6471	0.006412	5.8	0.0	0.4	40.0	23.6
19	190.6795	0	0.2	2.0	3.8	10.5	47.6
20	190.6819	0.000142	0.2	1.9	3.8	10.5	47.6
21	190.8907	0	0.8	29.8	0.8	1.9	22.5
22	190.9197	9.65E-05	0.8	30.9	0.7	1.9	22.3
23	191.8532	0	1.5	0.0	4.8	18.2	4.6
24	191.8542	0	1.5	0.0	4.7	18.1	4.5
25	191.8826	0.001471	1.2	0.0	5.3	18.1	4.7
26	191.8832	0.001455	1.2	0.0	5.2	18.0	4.6
27	191.9138	0	1.1	0.0	0.9	3.8	0.5
28	191.9141	0	1.0	0.0	0.7	3.3	0.4
29	191.9256	0.001357	1.0	0.0	0.5	2.4	0.1
30	191.9264	0.001392	1.0	0.0	0.5	2.4	0.1
31	191.9309	0	3.6	4.8	0.5	6.2	1.6
32	192.1184	0.000649	2.9	4.3	0.5	7.6	0.9
33	192.3907	0	5.0	2.2	0.4	4.7	2.1
34	192.3955	0	2.0	0.0	1.6	5.2	1.5
35	192.3969	0	2.0	0.0	1.6	5.1	1.5

36	192.4269	0.001929	2.1	0.0	1.5	4.9	1.4
37	192.4279	0.001902	2.1	0.0	1.5	4.9	1.4
38	192.4406	0.000824	5.5	1.3	0.4	3.8	2.6
39	192.5281	0	1.7	0.0	1.1	5.1	1.2
40	192.5287	0	1.6	0.0	1.0	5.0	1.2
41	192.5522	0.002164	1.8	0.0	1.1	5.0	1.3
42	192.5524	0.002146	1.7	0.0	1.1	5.1	1.3
43	192.6460	0	5.2	3.3	0.7	4.3	1.8
44	192.6925	0.000192	5.5	3.8	0.9	4.8	1.5
45	192.7048	0	1.5	0.0	1.3	3.8	1.5
46	192.7057	0	1.4	0.0	1.2	3.8	1.5
47	192.7074	0.000225	1.4	0.0	1.2	3.6	1.5
48	192.7083	0.000226	1.4	0.0	1.1	3.6	1.5
49	192.8339	0	1.3	3.0	0.2	0.9	3.4
50	192.8736	0.001014	0.7	3.1	0.1	0.3	3.6
51	192.9439	0	2.2	0.0	0.9	4.4	1.4
52	192.9452	0	2.0	0.5	0.7	3.8	1.9
53	192.9458	0	0.9	2.1	0.2	1.3	3.6
54	192.9534	0.000738	2.2	0.0	0.8	4.4	1.4
55	192.9549	0.000743	2.3	0.0	0.8	4.4	1.4
56	192.9564	0.000595	0.7	2.4	0.0	0.8	4.4
57	193.0667	0	7.5	3.5	0.4	1.9	1.2
58	193.1505	2.46E-05	9.1	3.8	0.3	1.6	0.9
59	193.2301	0	1.2	0.0	0.2	1.8	0.8
60	193.2311	0	1.3	0.0	0.2	1.8	0.8
61	193.2367	0.000461	1.2	0.0	0.2	1.8	0.8
62	193.2378	0.000471	1.2	0.0	0.2	1.8	0.8
63	193.3087	0	3.0	3.5	0.2	0.7	2.3
64	193.3326	0	1.1	0.0	0.2	1.6	0.8
65	193.3330	0	1.2	0.0	0.2	1.6	0.8
66	193.3363	0.000279	1.2	0.0	0.2	1.6	0.7
67	193.3367	0.00028	1.3	0.0	0.2	1.6	0.8
68	193.3950	0.005271	3.0	4.1	0.2	0.7	2.4
69	193.4329	0	1.9	3.3	0.2	0.9	2.3
70	193.4681	0.000357	1.4	3.8	0.2	1.0	2.6
71	193.4842	0	4.0	0.0	0.5	3.3	0.8
72	193.4844	7.97E-06	4.1	0.0	0.5	3.3	0.8
73	193.4853	0	4.1	0.0	0.5	3.3	0.8
74	193.4855	8.72E-06	4.1	0.0	0.5	3.3	0.8
75	193.6202	0	1.1	2.5	0.2	0.6	2.2
76	193.6701	2.59E-06	1.3	2.0	0.4	0.4	2.1
77	193.6792	0	0.9	0.0	0.3	1.3	1.0
78	193.6805	0	0.9	0.0	0.3	1.3	1.0
79	193.6929	0.000915	0.9	0.0	0.3	1.3	1.0

80	193.6944	0.000932	0.9	0.0	0.3	1.3	1.0
81	193.7133	0	0.7	1.3	0.3	0.3	2.0
82	193.7488	1.16E-06	0.3	1.3	0.1	0.3	2.2
83	193.8783	0	1.0	0.9	0.3	0.6	1.9
84	193.8867	0.00027	0.2	0.6	0.2	0.6	1.9
85	193.9168	0	3.3	1.0	0.3	0.9	1.5
86	193.9620	0.003162	4.4	0.8	0.4	0.9	1.2
87	194.0623	0	4.1	0.1	0.3	1.6	1.0
88	194.0633	0	4.7	0.2	0.4	1.5	1.0
89	194.0799	0.000893	4.0	0.1	0.3	1.7	1.0
90	194.0808	0.000877	4.8	0.2	0.4	1.6	1.0
91	194.1580	0	4.4	0.1	0.4	2.0	1.2
92	194.1595	0	4.6	0.1	0.4	2.0	1.2
93	194.1747	0.00097	4.3	0.1	0.5	2.3	1.1
94	194.1768	0.001018	4.6	0.1	0.5	2.2	1.1
95	194.1841	0	2.5	1.1	0.6	1.4	2.3
96	194.2206	0	2.5	0.0	0.6	2.4	1.0
97	194.2218	0	2.5	0.0	0.6	2.4	1.0
98	194.2219	0.001856	2.3	1.3	0.7	1.5	2.3
99	194.2327	0.001047	3.1	0.0	0.6	2.4	1.1
100	194.2347	0.001101	3.4	0.0	0.6	2.4	1.1

Table S12. Lowest unoccupied molecular orbitals (LUMO to LUMO+17) and their corresponding Reduced Löwdin atomic percent character for **1** (M = Th) using b3lyp-d4 on the PBE optimized geometry. H atom contribution is the sum of all four hydrogens directly connected to B. The Mulliken symbol labels shown in parentheses in the orbital number column correspond to those shown in Figure 5.

Orbital Number	Orbital Image	Energy (eV)	Composition					
			Th				B	H
			s	p	d	f	p	total
183 (1a)	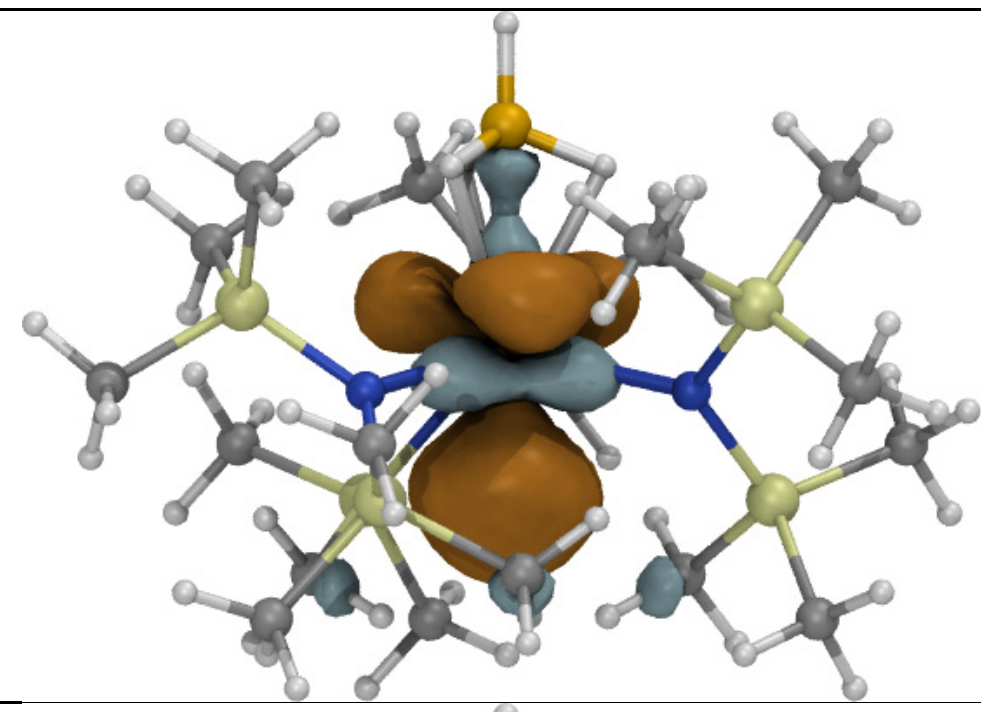	-1.0236	13.6	1.4	1.5	57.4	0.7	0.4
184 (2a)	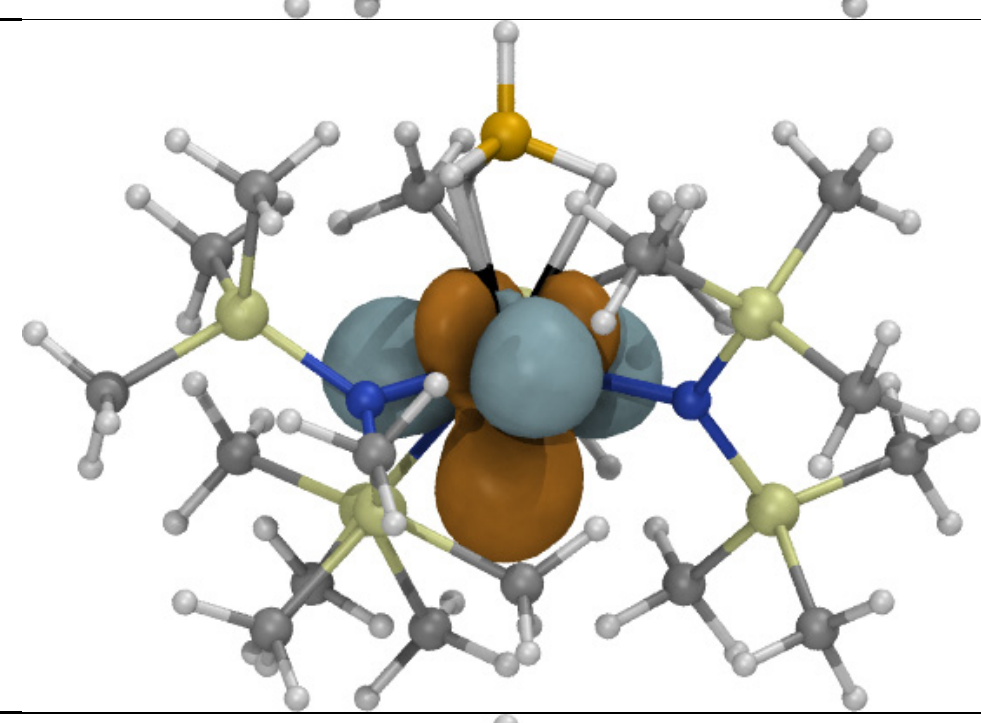	-0.6828	1.1	0.9	17.3	69.5	0.1	0.3
185 (1e)	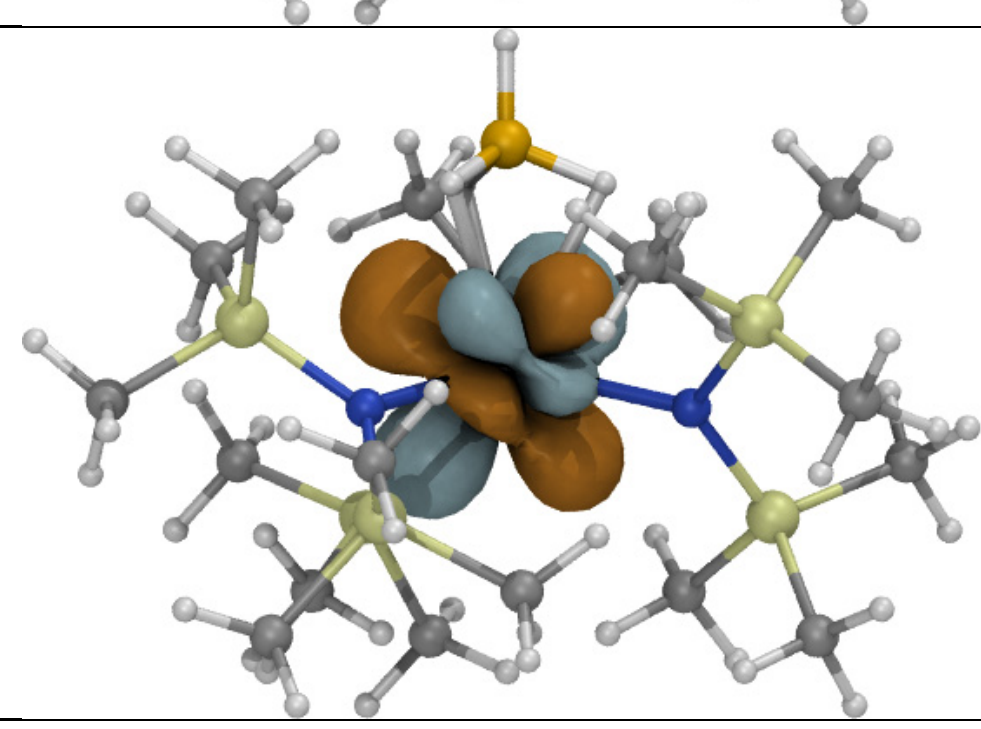	-0.6403	0.0	0.0	13.7	77.4	0.4	0.2

186<br>(1e)	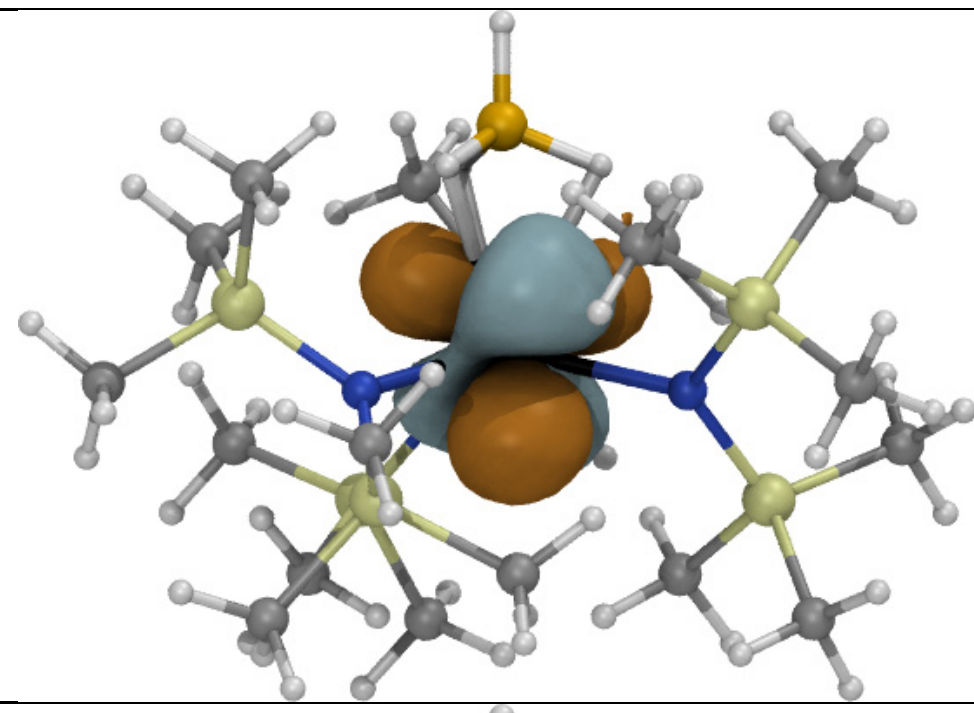	-0.6397	0.0	0.0	13.7	77.4	0.4	0.2
187<br>(2e)	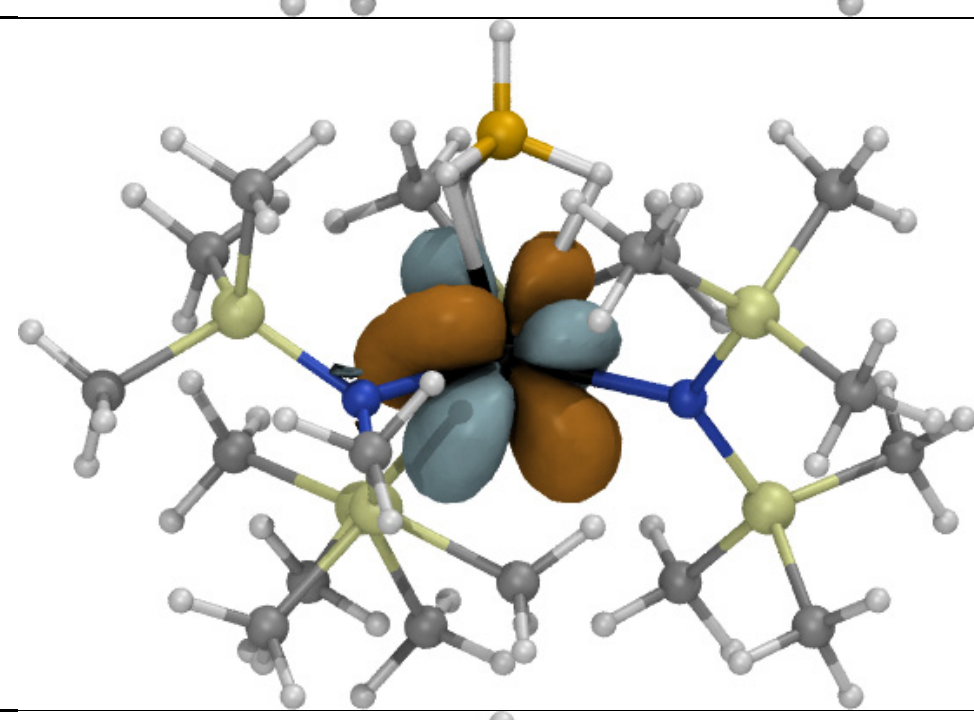	-0.2961	0.0	0.0	3.3	86.4	1.0	0.3
188<br>(2e)	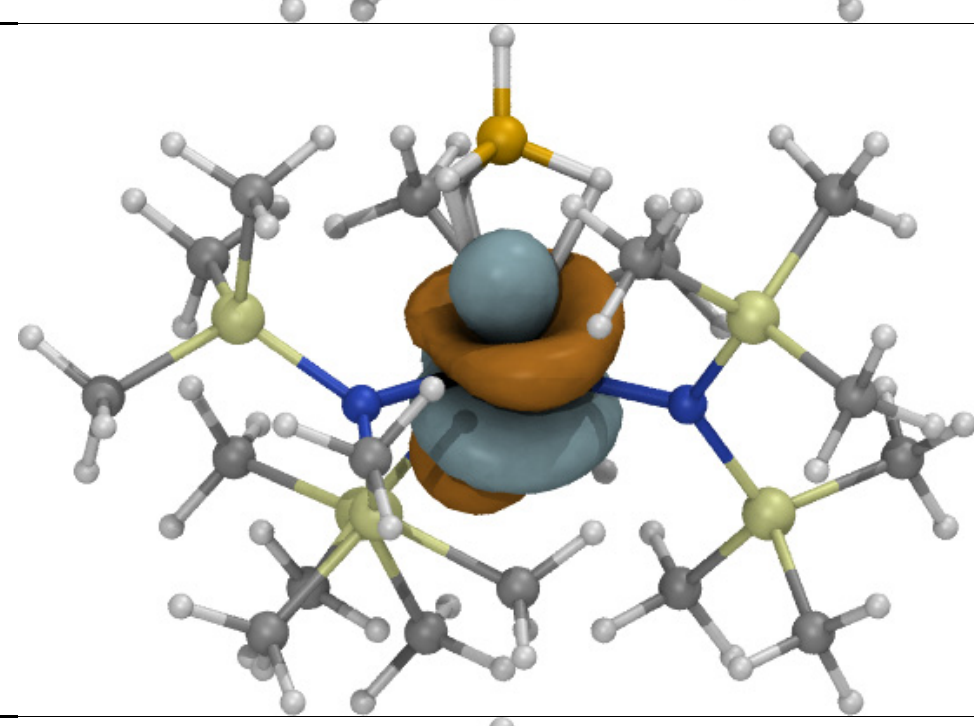	-0.2953	0.0	0.0	3.3	86.4	1.0	0.3
189<br>(3a)	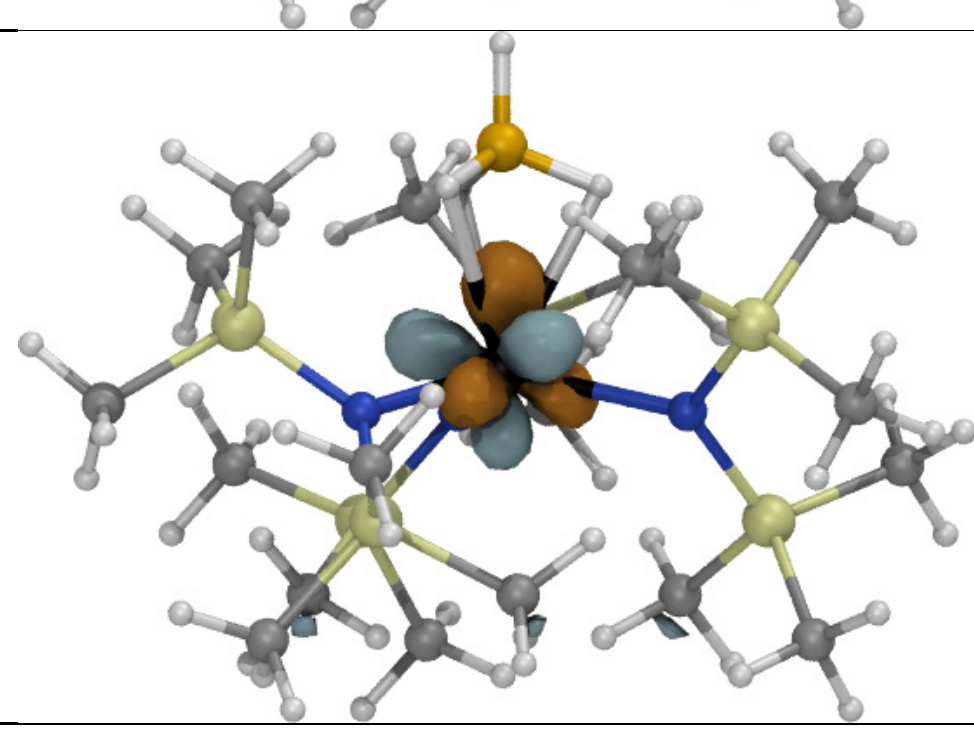	-0.2571	36.8	0.4	1.1	16.9	1.0	0.6

190 (4a)	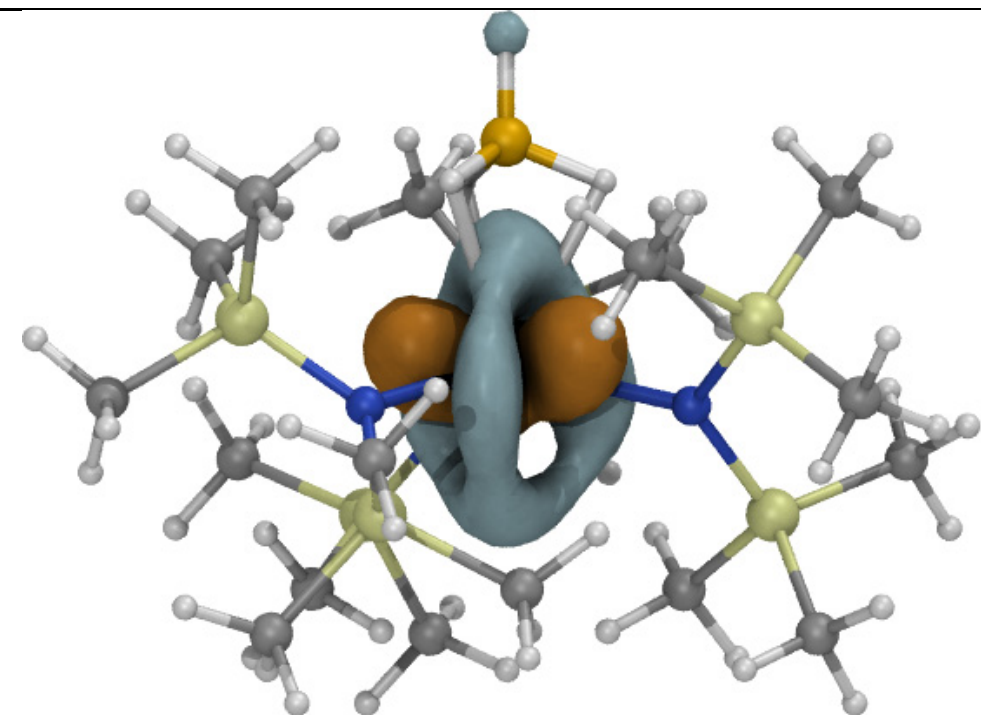	-0.1337	5.7	2.8	6.7	59.0	0.0	1.1
191 (5a)	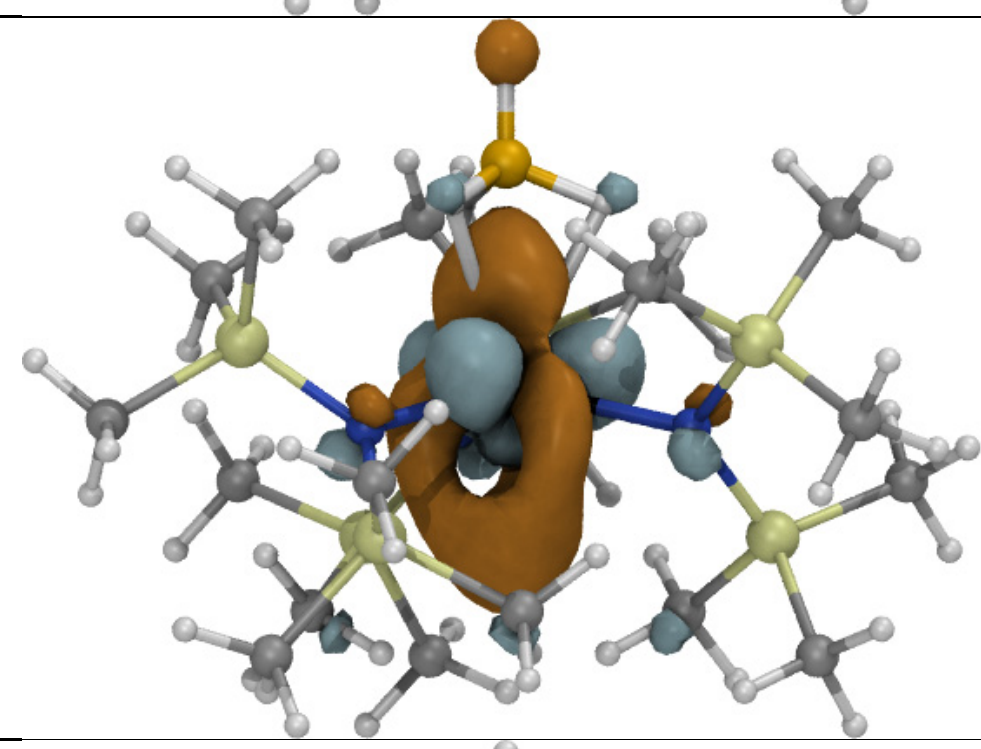	0.0619	1.6	4.0	11.2	47.8	0.2	2.7
192 (3e)	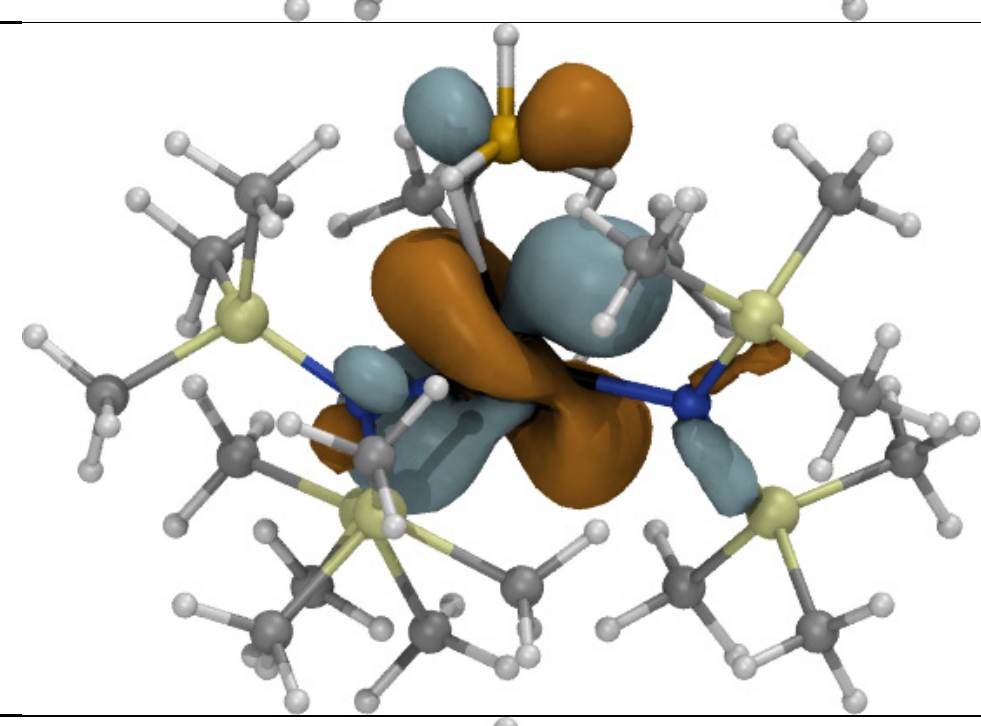	0.1188	0.0	0.5	50.8	8.6	7.4	1.7
193 (3e)	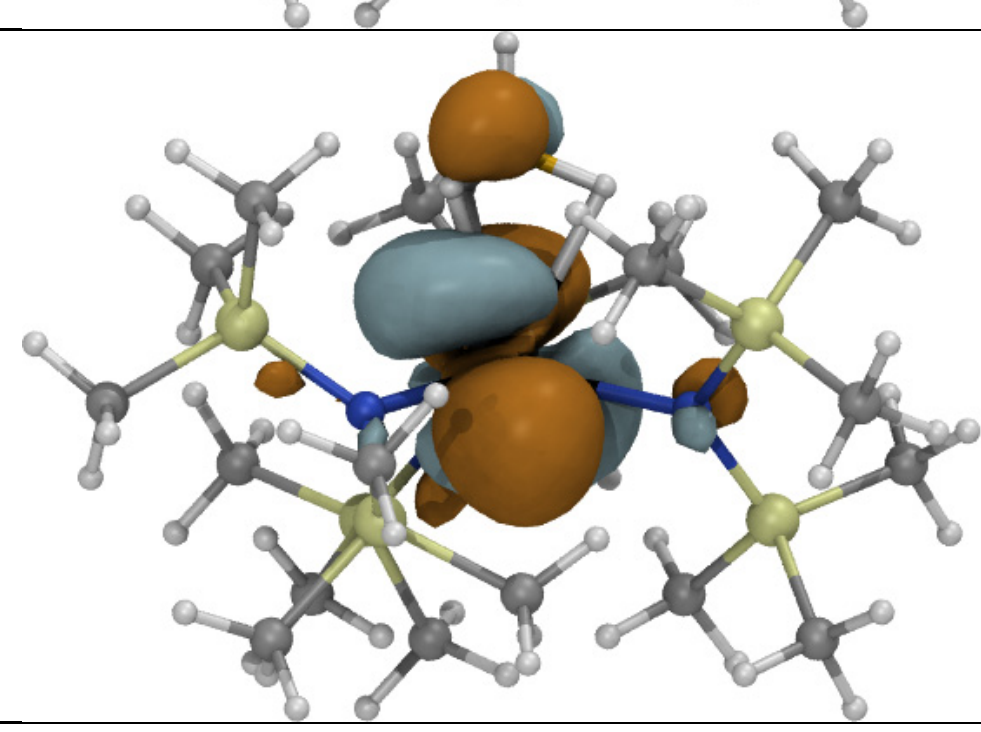	0.1205	0.0	0.5	50.8	8.6	7.3	1.8

194	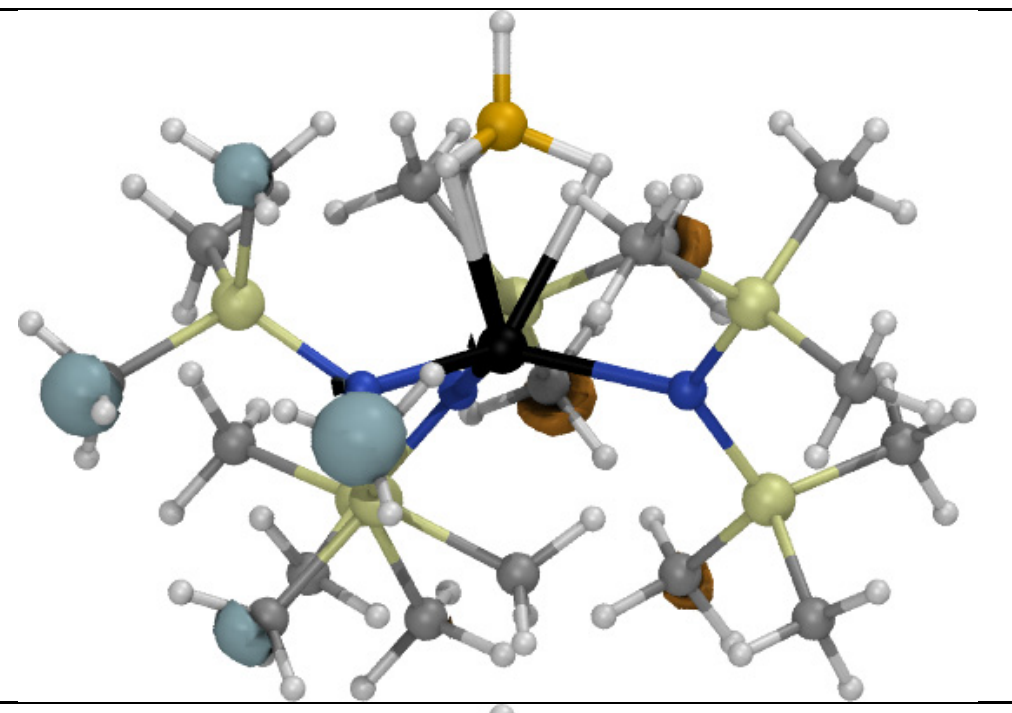	0.7462	0.0	0.5	2.6	0.1	1.1	0.1
195	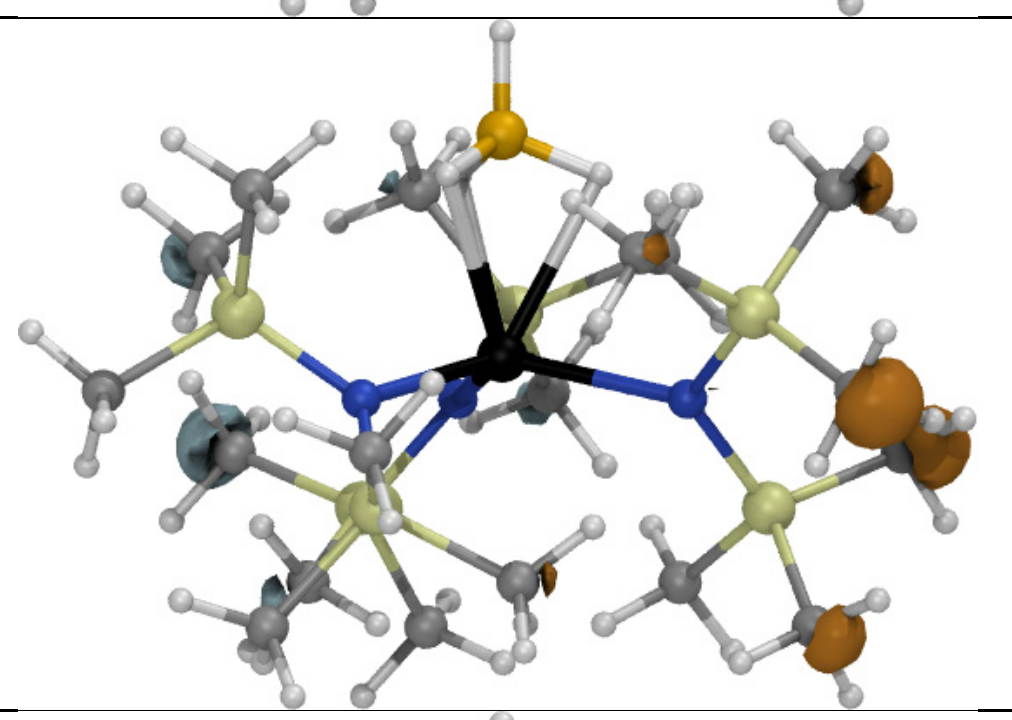	0.7471	0.0	0.5	2.6	0.1	1.1	0.2
196	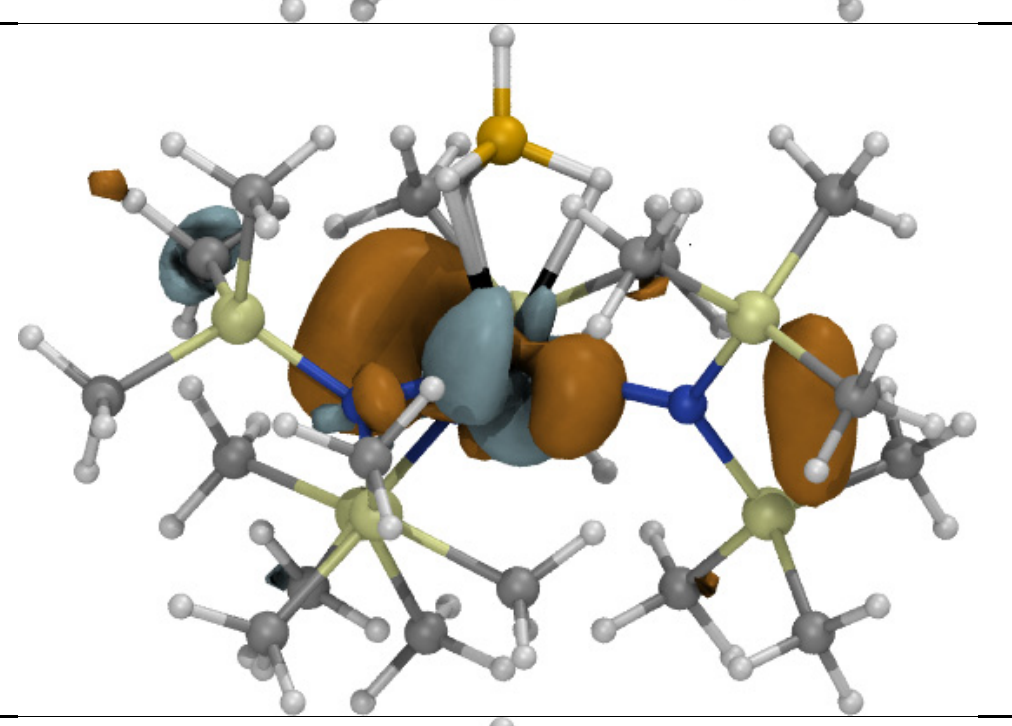	1.0257	0.0	5.9	20.2	5.3	1.3	0.5
197	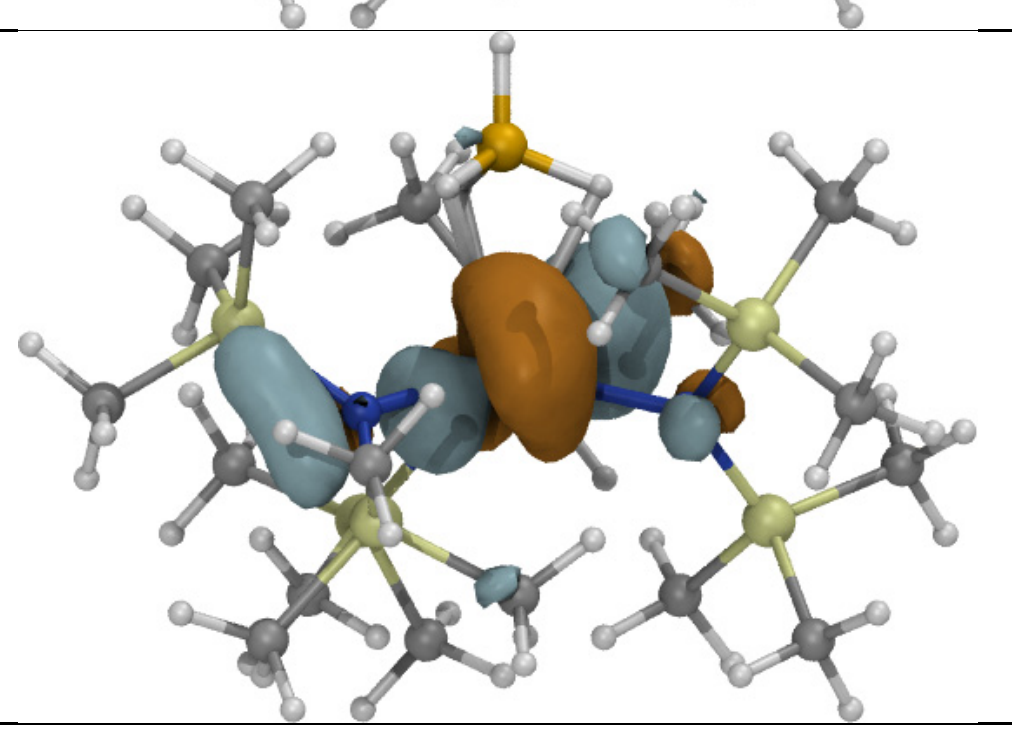	1.0263	0.0	5.8	20.2	5.2	1.3	0.5

198	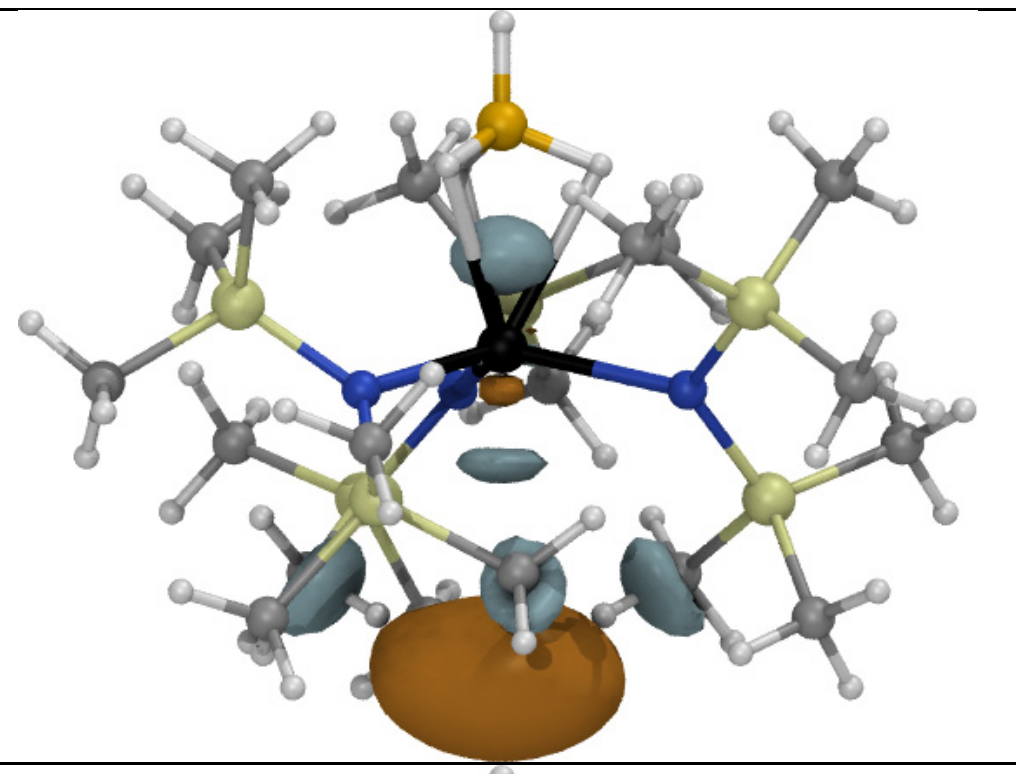	1.0411	4.6	0.5	8.2	0.9	2.9	0.6
199	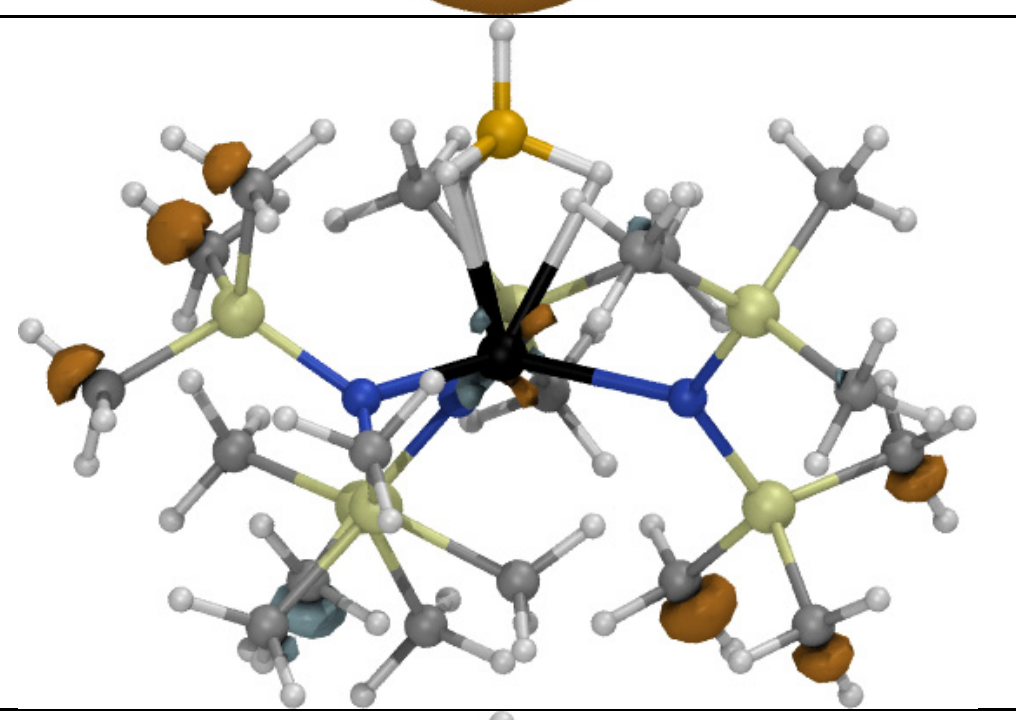	1.3647	0.0	1.1	5.5	1.1	2.1	0.1
200	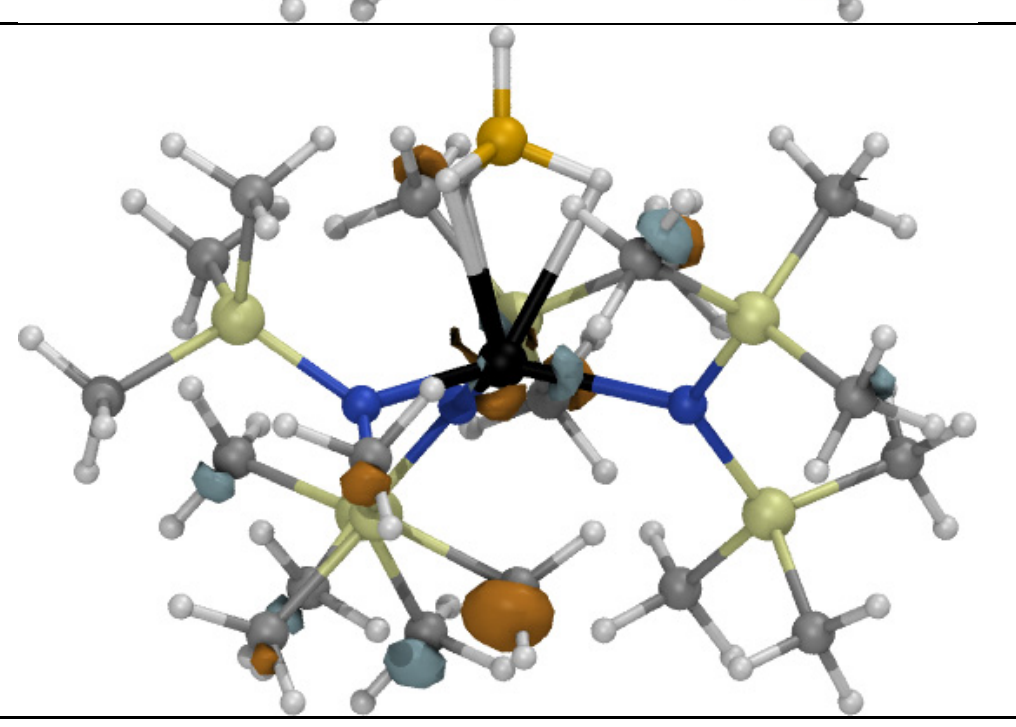	1.3653	0.0	1.1	5.5	1.2	1.9	0.2

Table S13. Lowest unoccupied molecular orbitals (LUMO to LUMO+15) and their corresponding Reduced Löwdin atomic percent character for **2** (M =U) using b3lyp-d4 on the PBE optimized geometry. H atom contribution is the sum of all four hydrogens directly connected to B. The Mulliken symbol labels shown in parentheses in the orbital number column correspond to those shown in Figure 5.

Orbital Number	Orbital Image	Energy (eV)	Composition					
			U				B	H
			s	p	d	f	p	total
185 (1e)	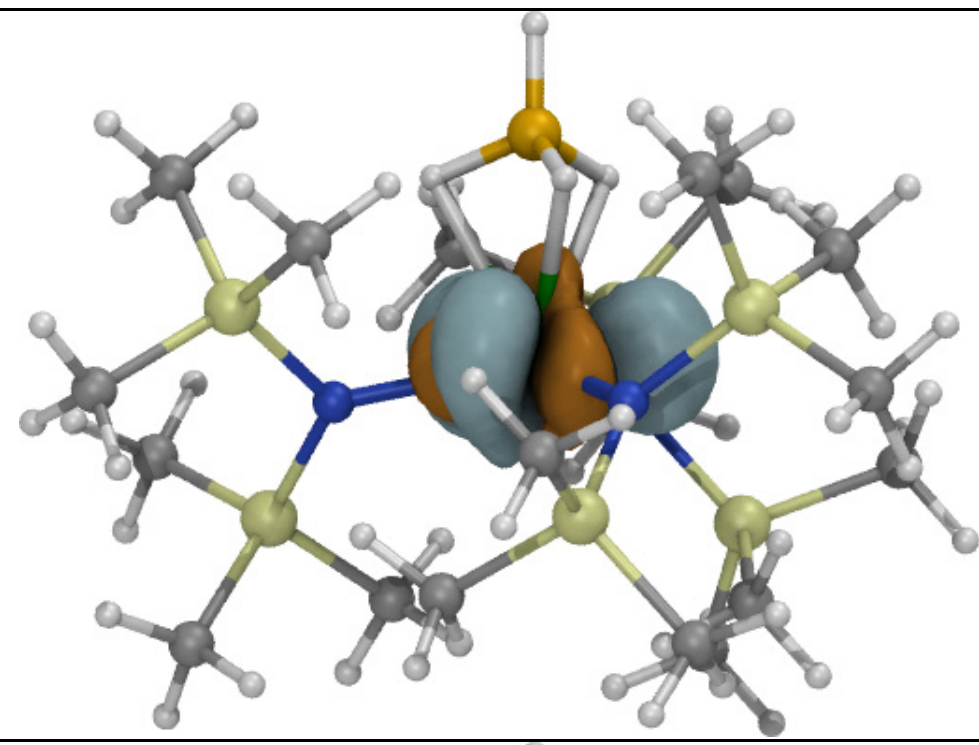	-2.4519	0.0	0.0	1.8	93.2	0.4	0.1
186 (1e)	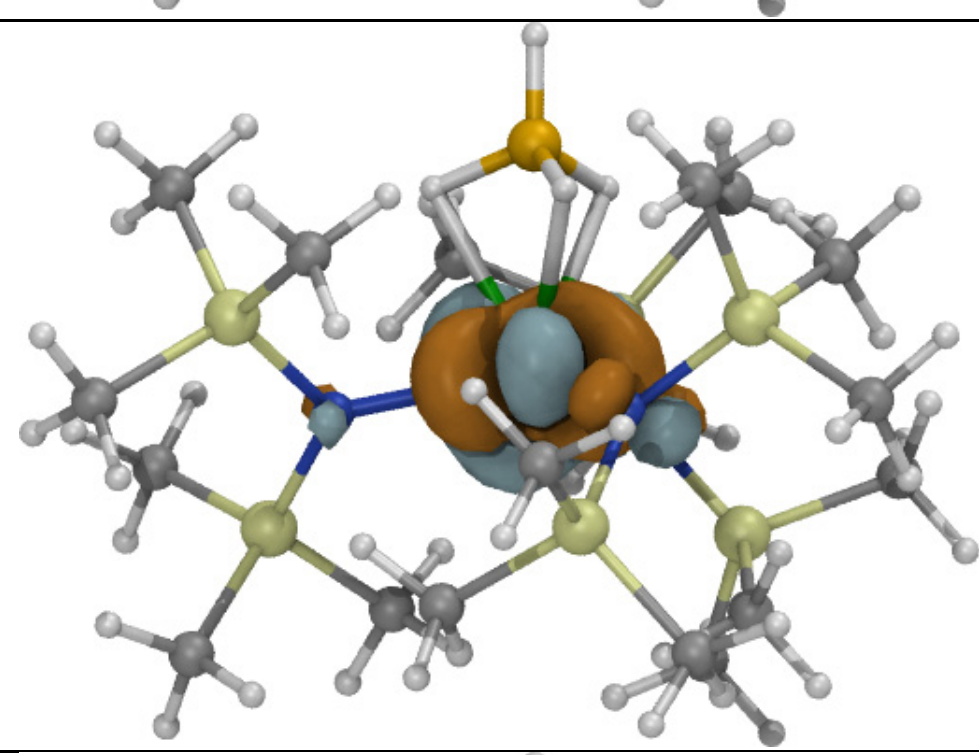	-2.4485	0.0	0.0	1.9	92.9	0.4	0.1
187 (1a)	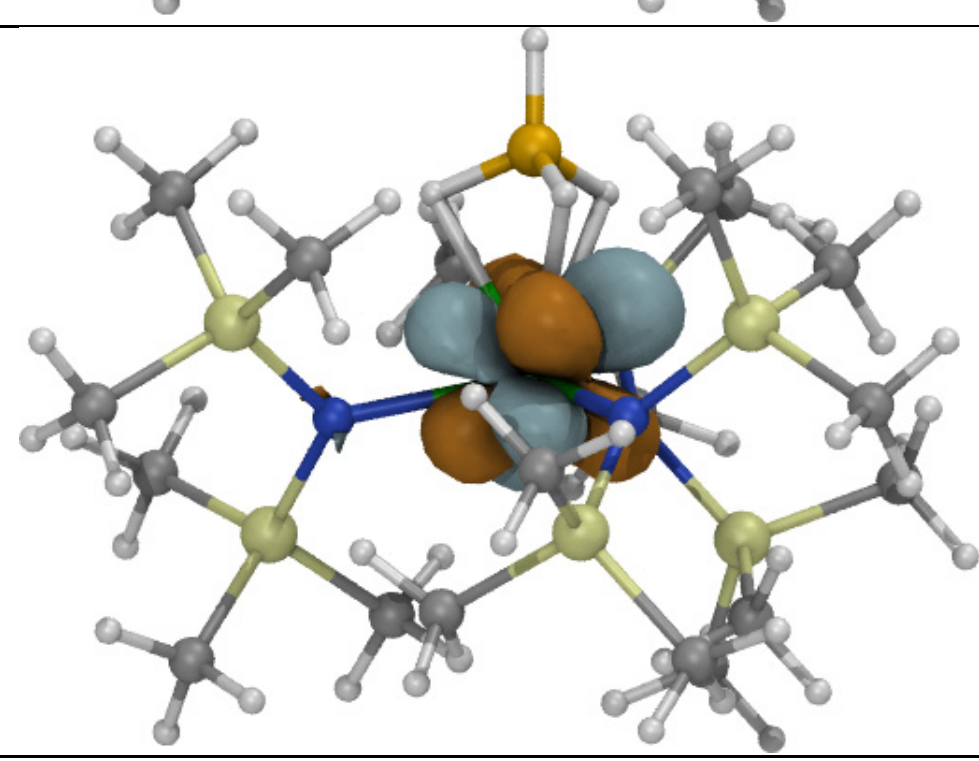	-1.8651	0.3	0.1	1.3	89.2	0.0	0.0

188 (2e)	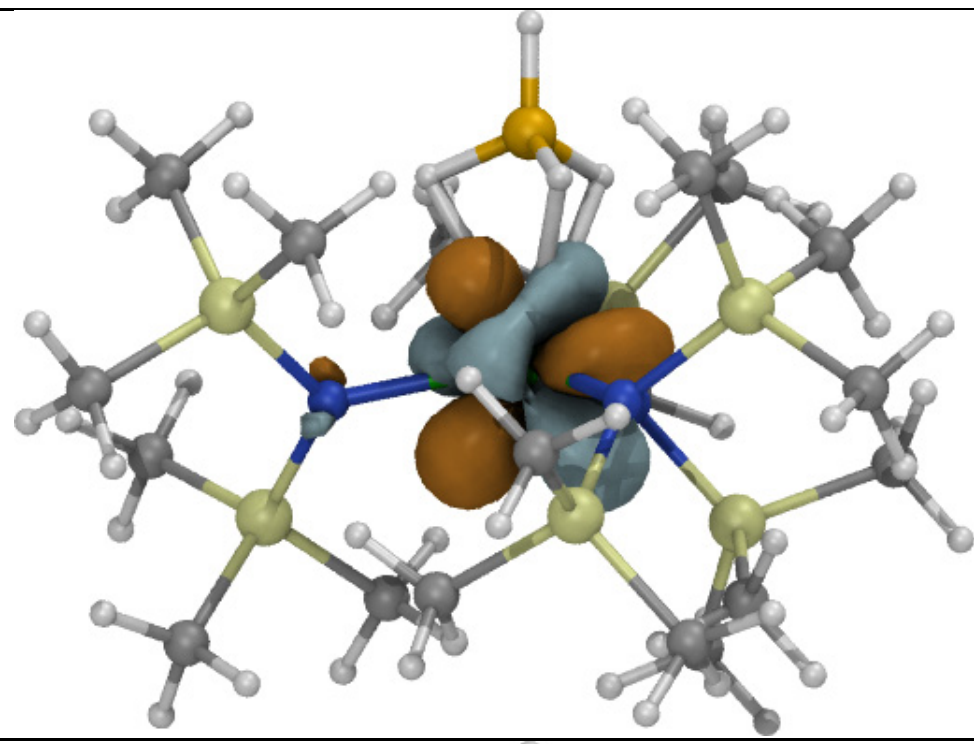	-1.5446	0.0	0.0	3.1	89.8	1.4	0.6
189 (2e)	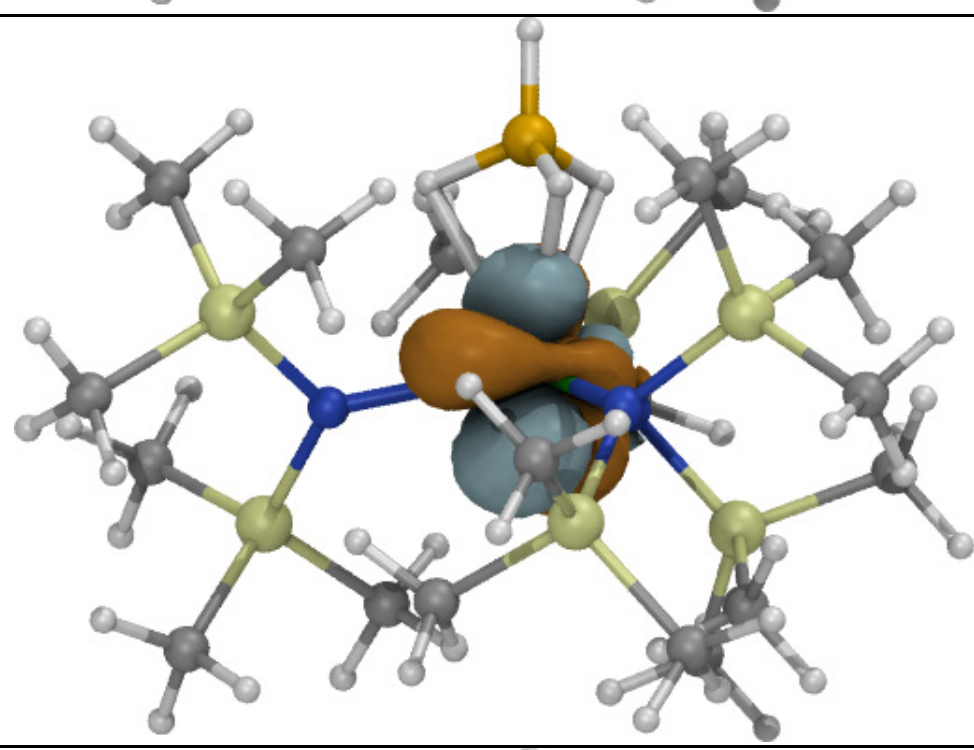	-1.5028	0.0	0.0	3.0	90.2	1.4	0.5
190 (2a)	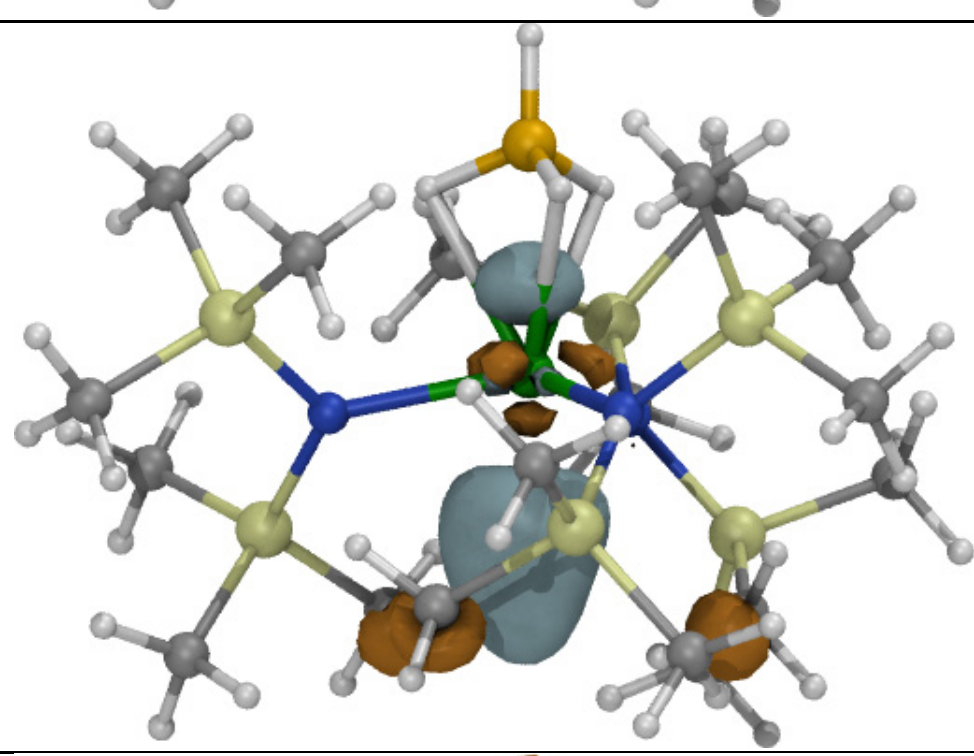	-0.3111	41.5	1.2	2.9	2.0	0.5	0.5
191 (3a)	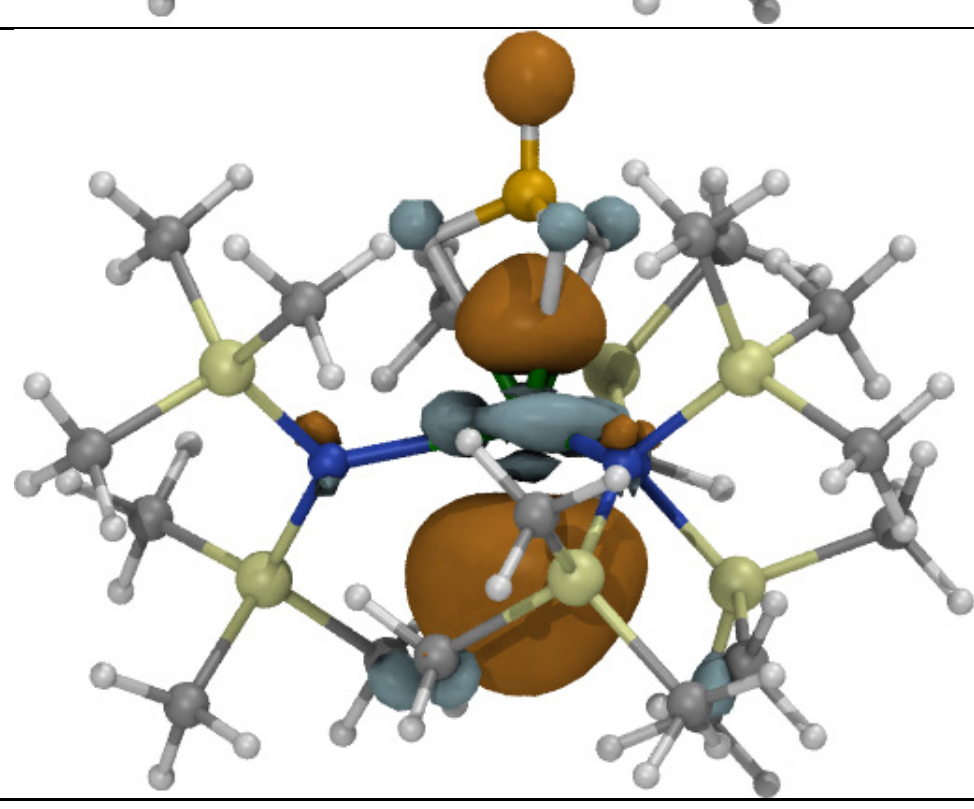	-0.0142	7.8	8.1	29.1	3.0	0.1	4.4

192 (3e)	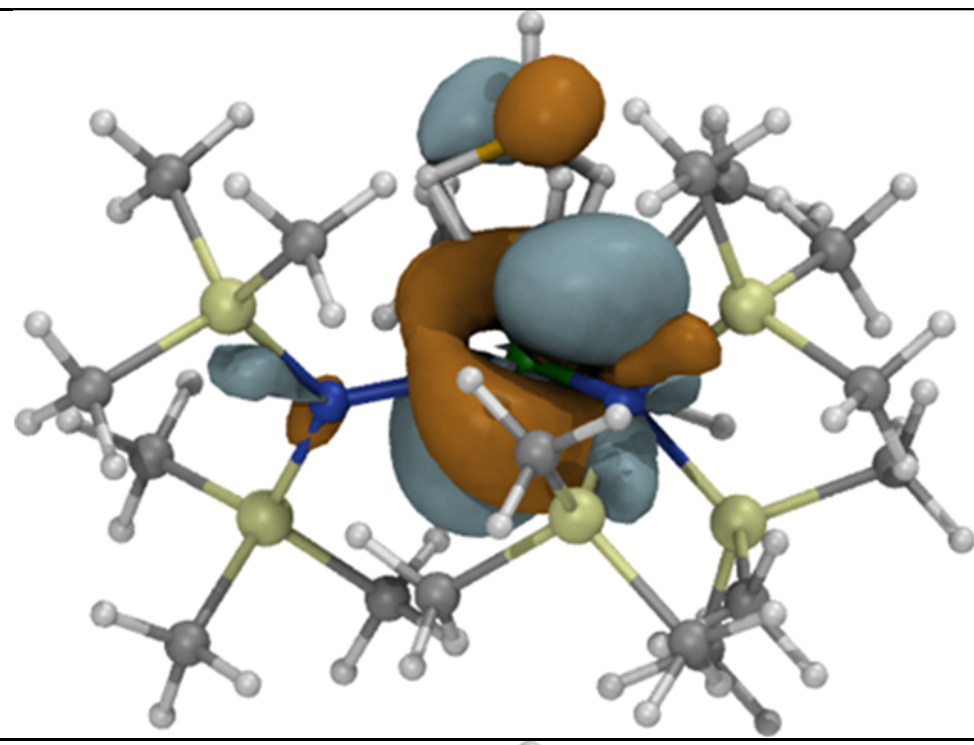	0.1912	0.0	0.7	56.4	0.9	6.5	1.7
193 (3e)	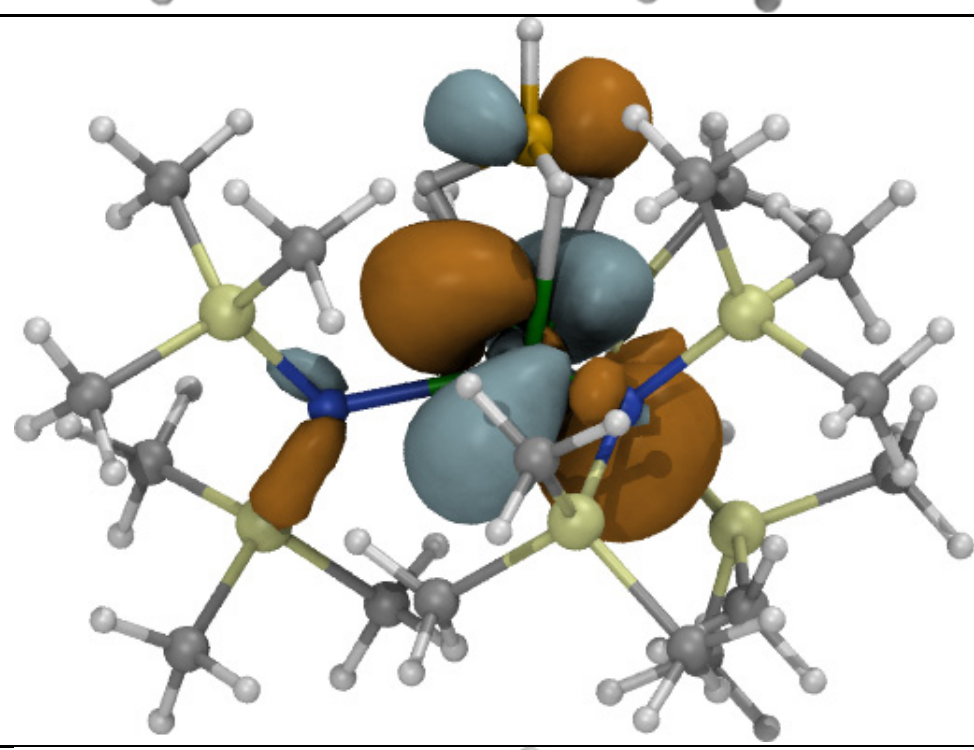	0.2157	0.0	0.6	56.0	1.0	6.6	1.8
194	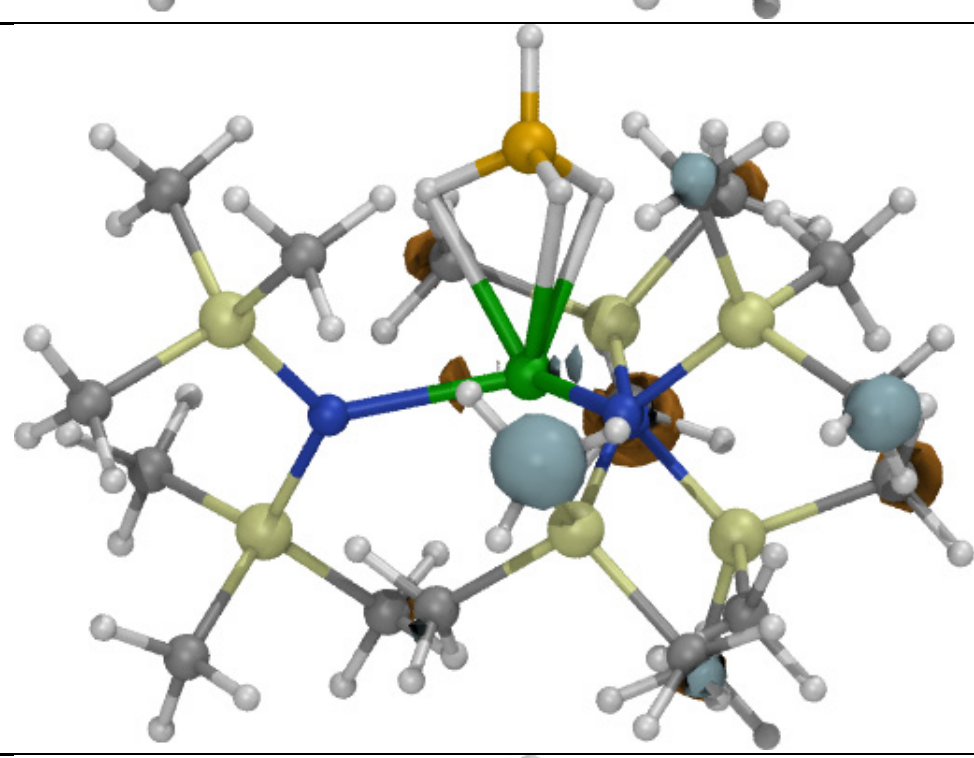	0.7891	0.0	0.5	3.5	0.1	1.1	0.1
195	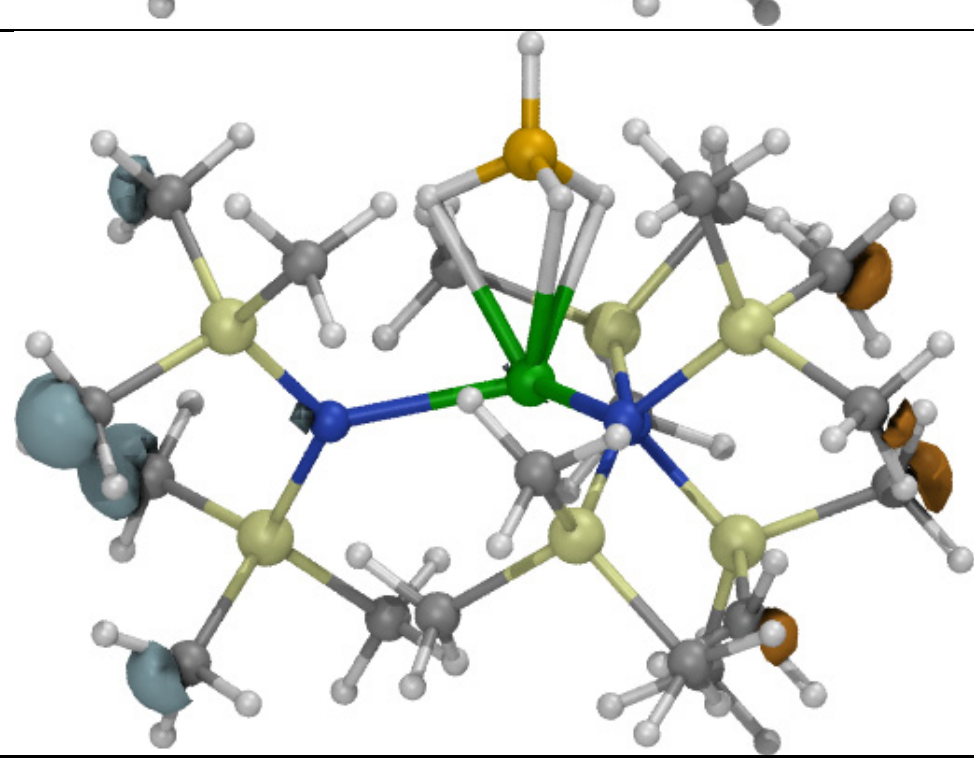	0.7949	0.0	0.6	2.9	0.1	1.1	0.2

196	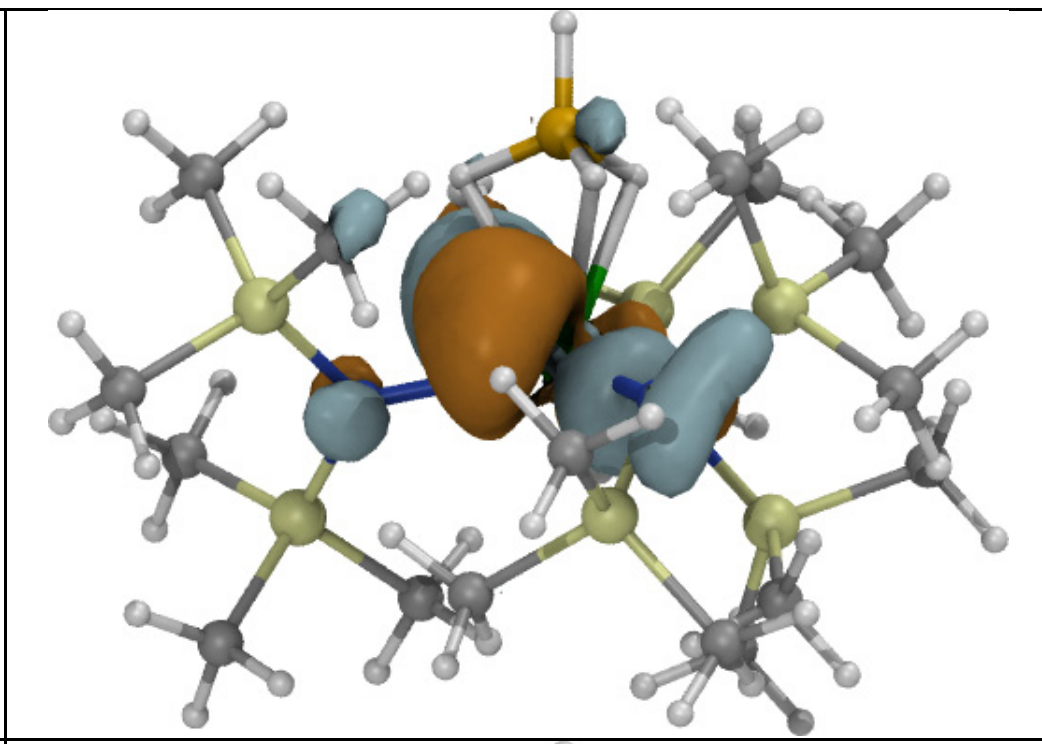	0.9512	0.0	6.2	27.7	2.5	1.9	0.9
197	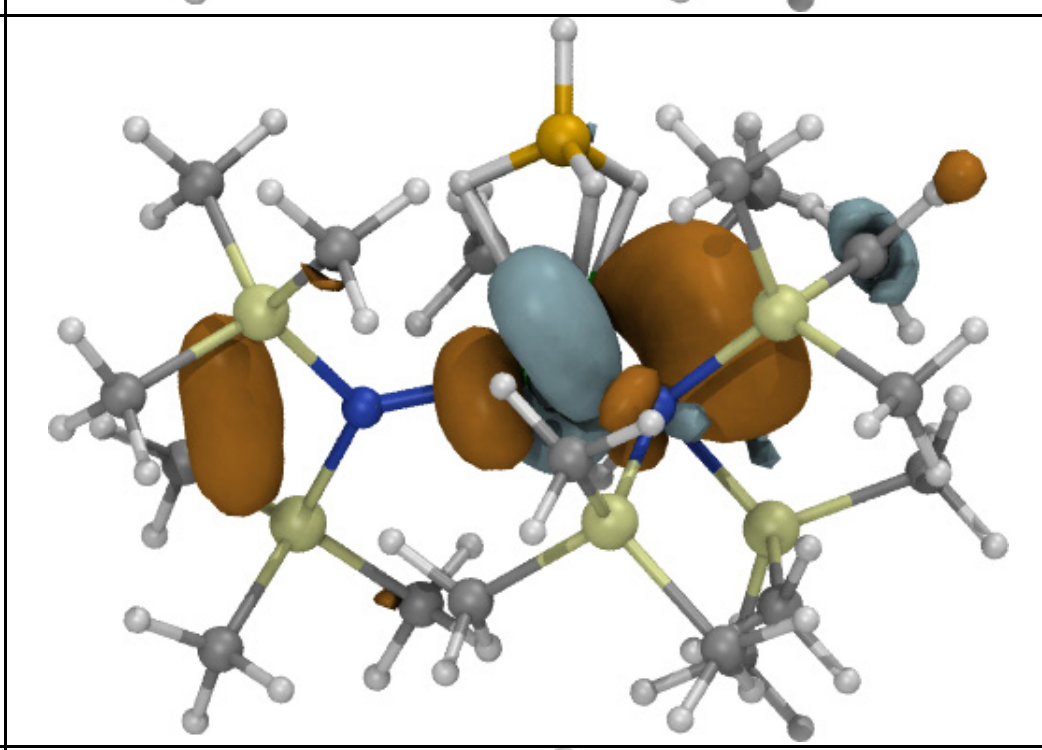	0.9674	0.0	6.3	25.7	2.2	1.9	0.6
198	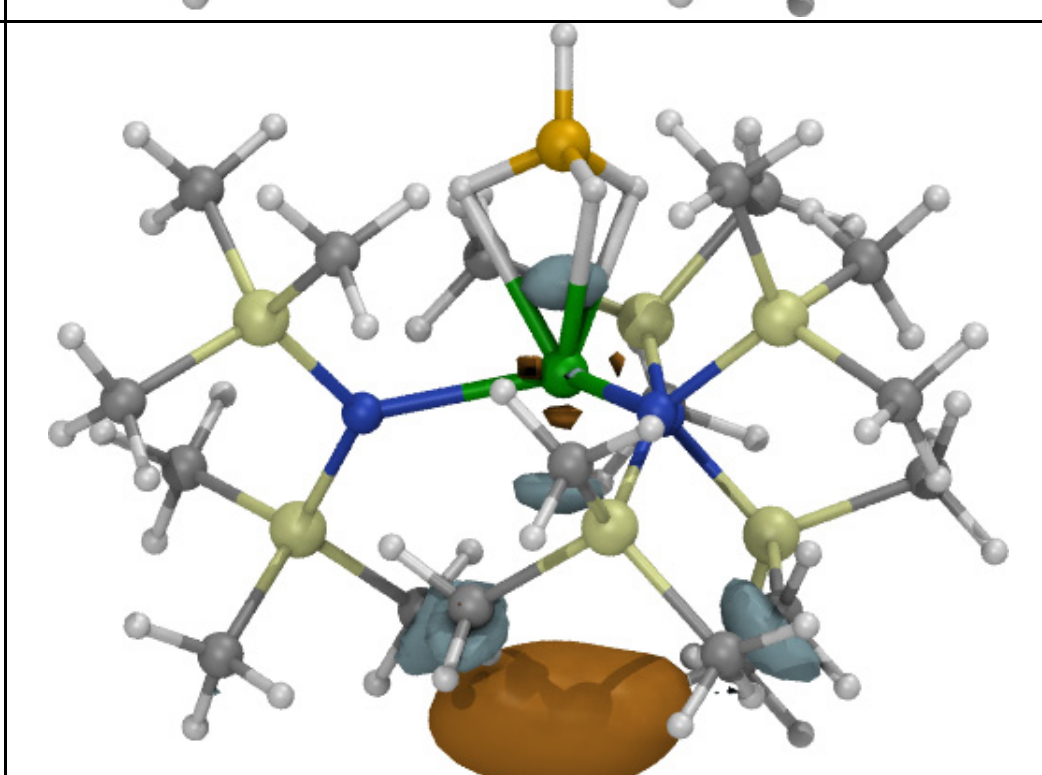	1.1423	4.1	0.3	4.1	1.4	3.8	1.6
199	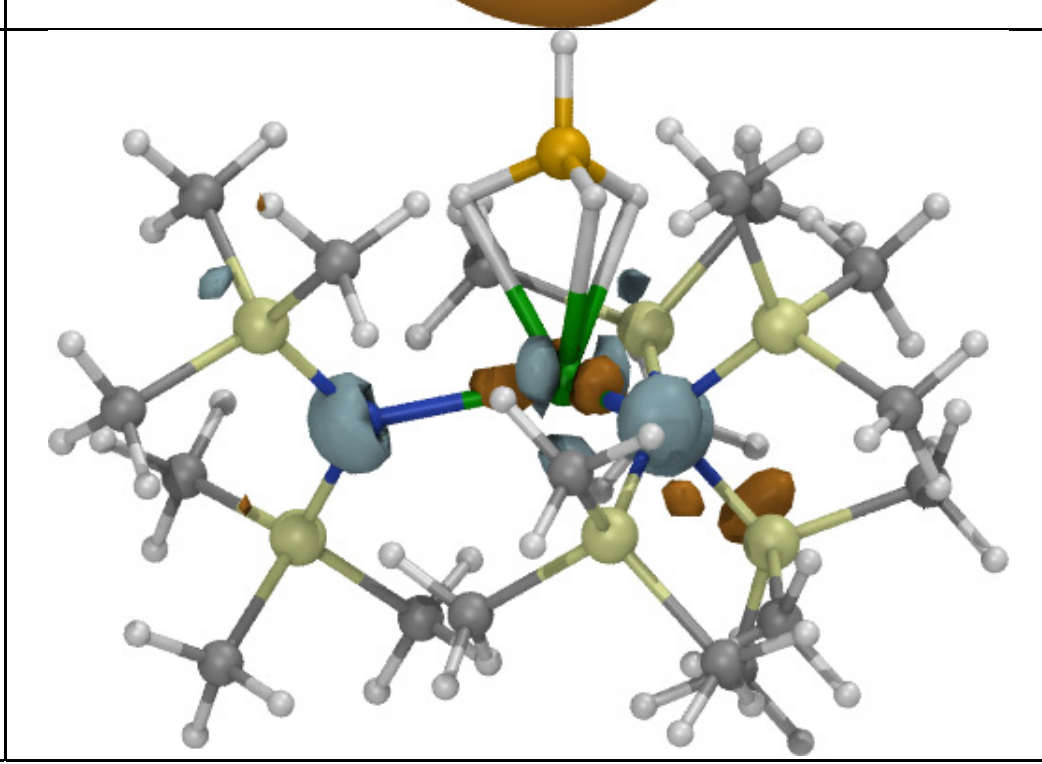	1.3192	2.8	0.1	5.4	4.8	5.8	0.9

200	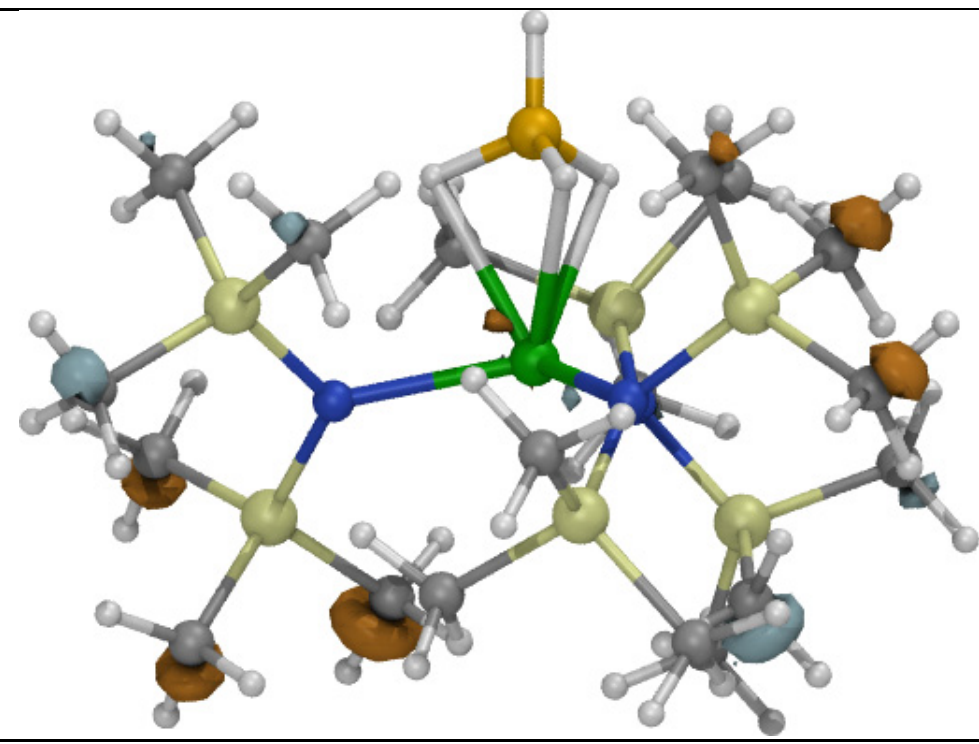	1.4027	0.0	1.0	3.6	0.6	2.0	0.2

Table S14. Natural Transition Orbitals (NTOs) accepting the B3LYP-D3 density on the PBE geometry of **1** (M = Th). Acceptor orbitals and their corresponding states with intensities larger than 0.00005 a.u. displayed. Energies are shifted by 9.3 eV.

State	Energy	Intensity	Acceptor Orbital
2	189.679	0.001146	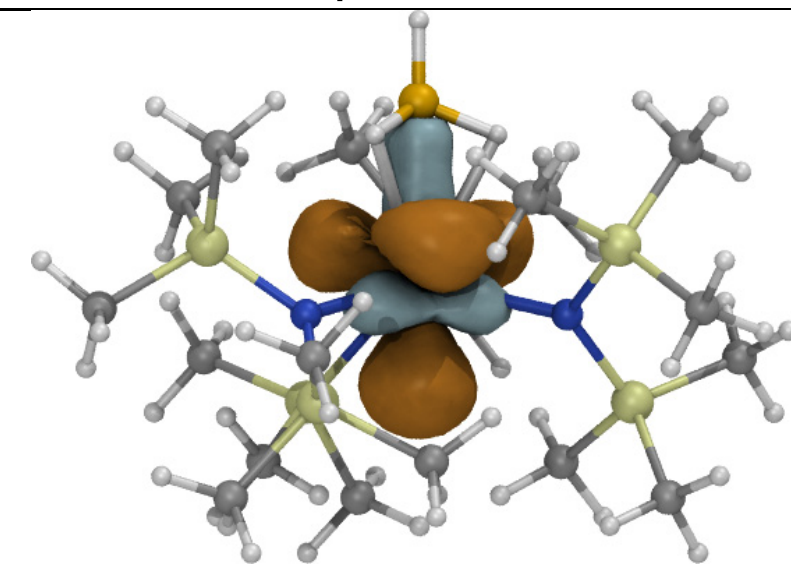 50% α, 50% β
5	189.958	0.001075	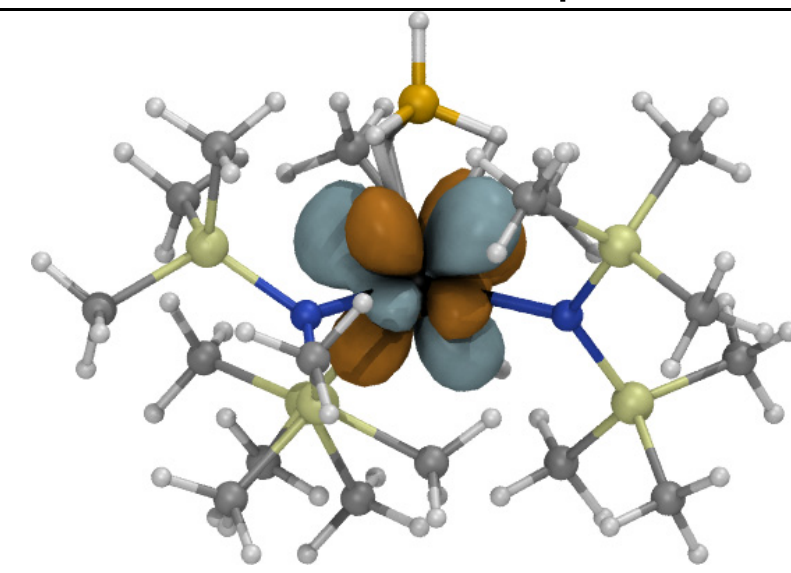 50% α, 50% β
6	189.959	0.001062	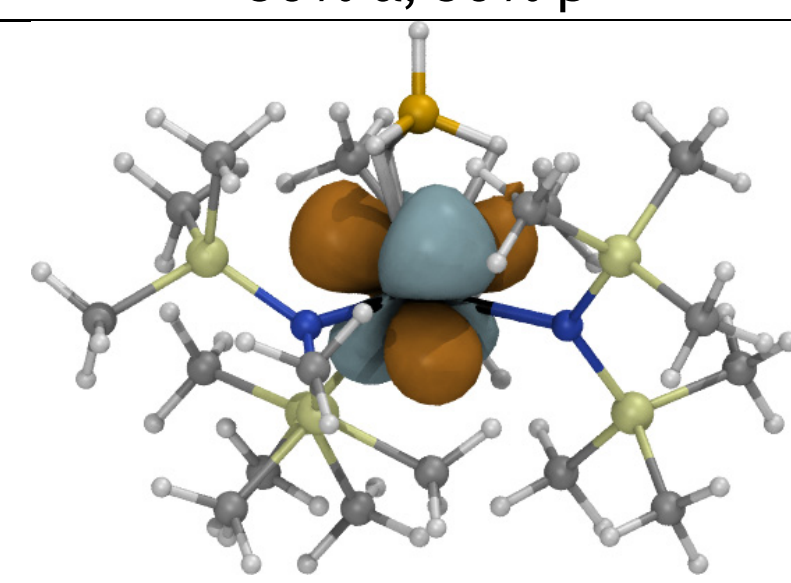 50% α, 50% β
11	190.255	0.004375	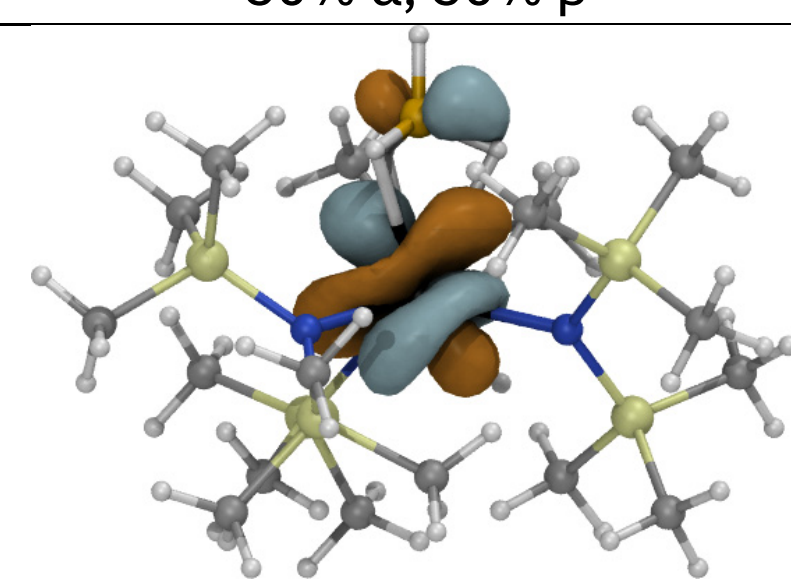 50% α, 50% β

12	190.255	0.004373	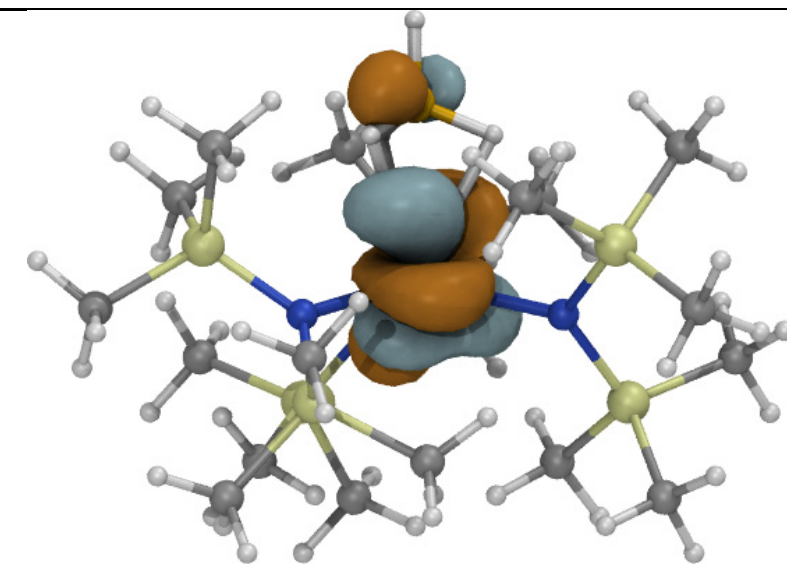 50% α, 50% β
14	190.479	0.000100	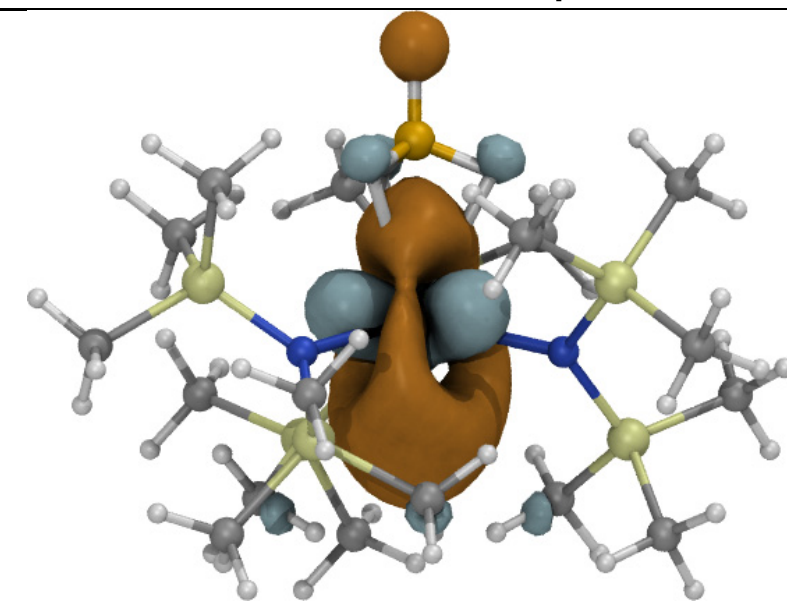 50% α, 50% β
17	190.645	0.006409	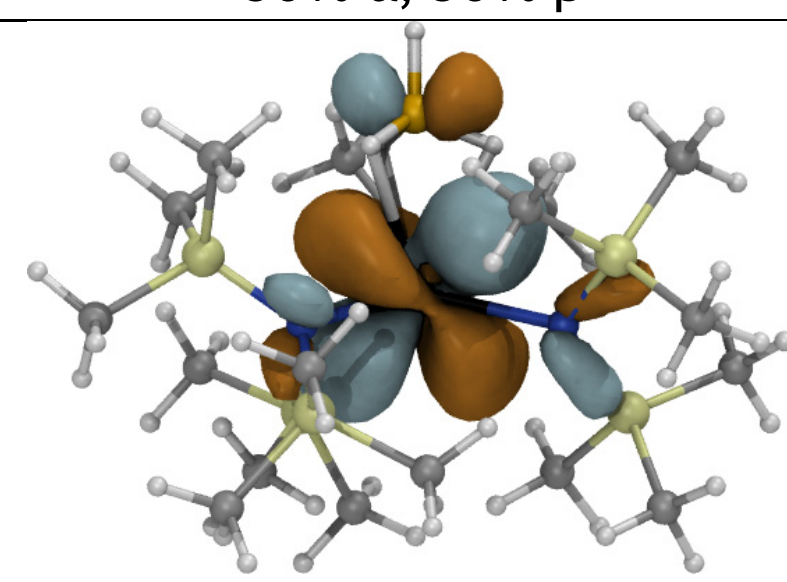 50% α, 50% β
18	190.647	0.006412	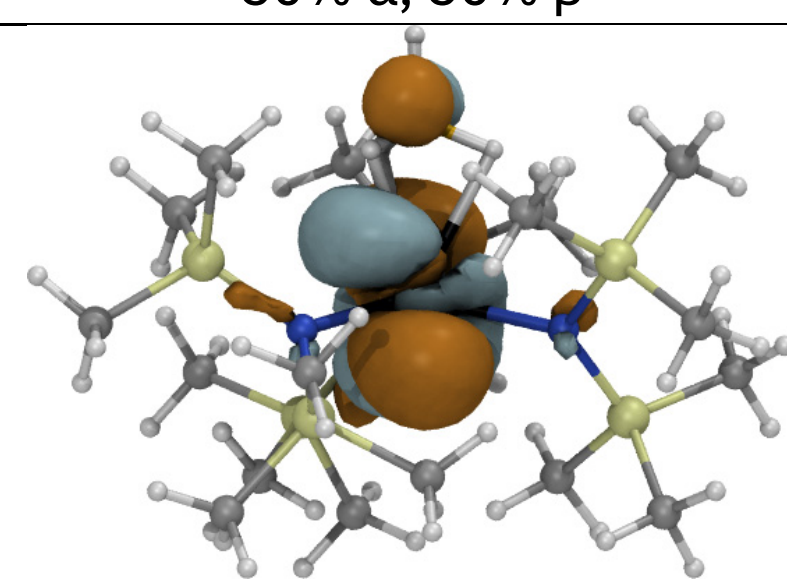 50% α, 50% β
20	190.682	0.000142	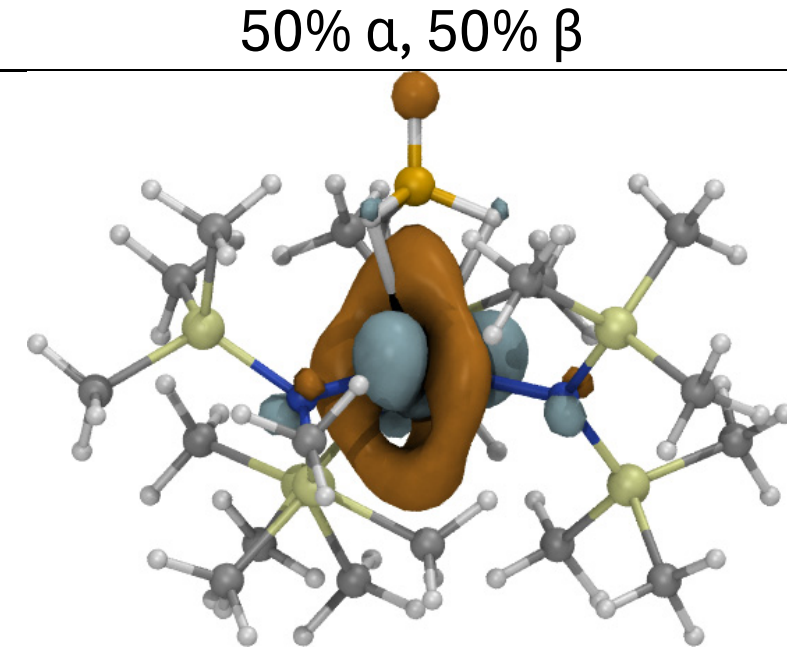 50% α, 50% β

22	190.920	0.000097	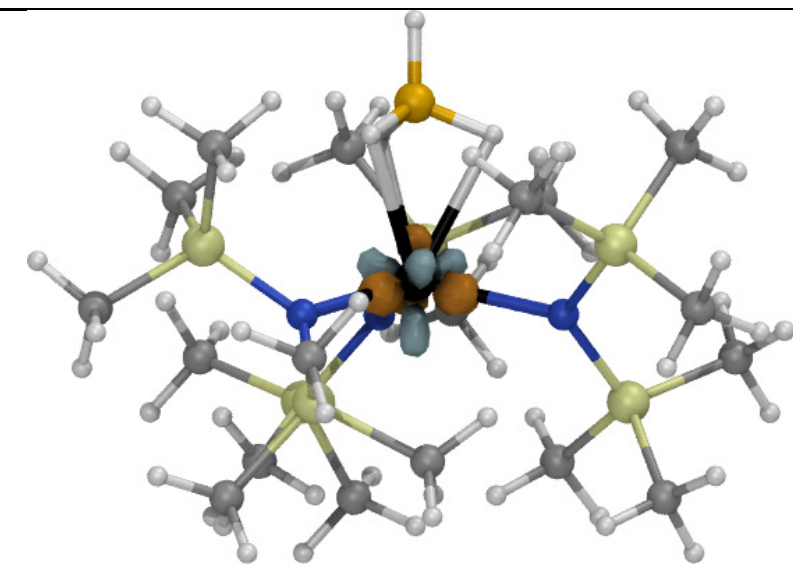 50% α, 50% β
25	191.883	0.001471	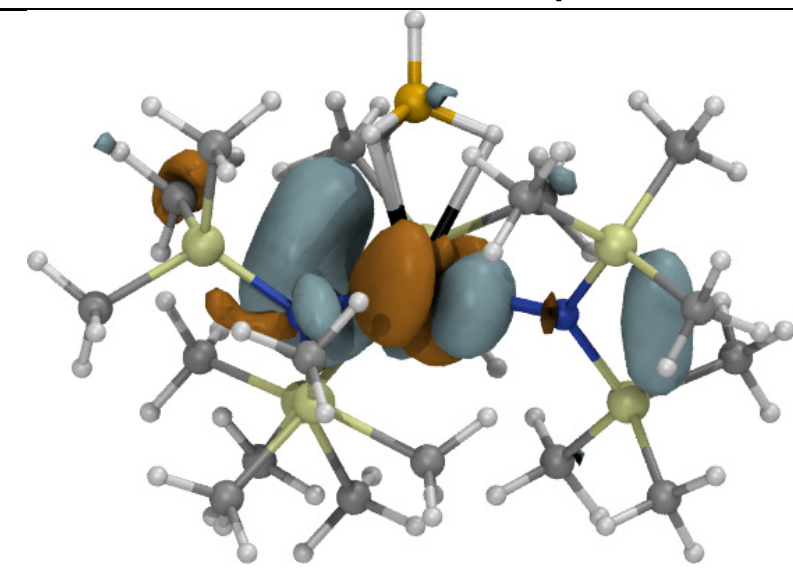 50% α, 50% β
26	191.883	0.001455	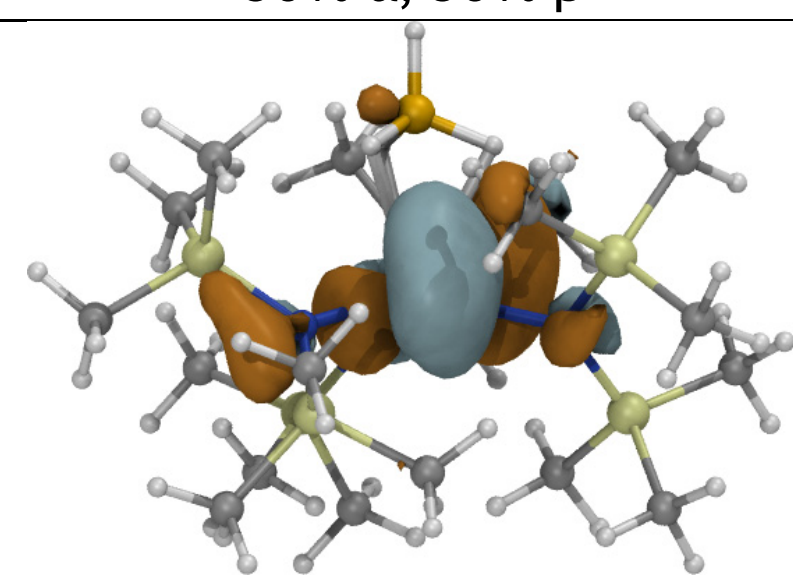 50% α, 50% β
29	191.926	0.001357	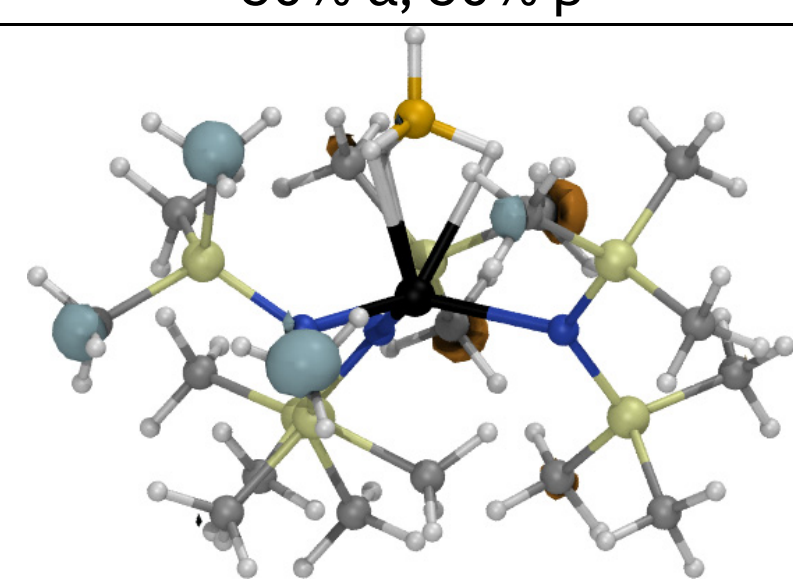 50% α, 50% β
30	191.926	0.001392	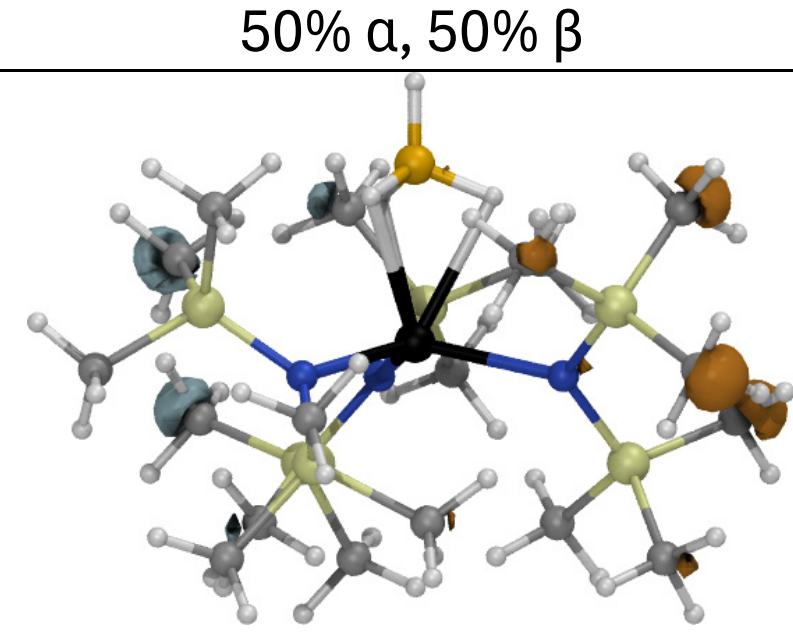 50% α, 50% β

32	192.118	0.000649	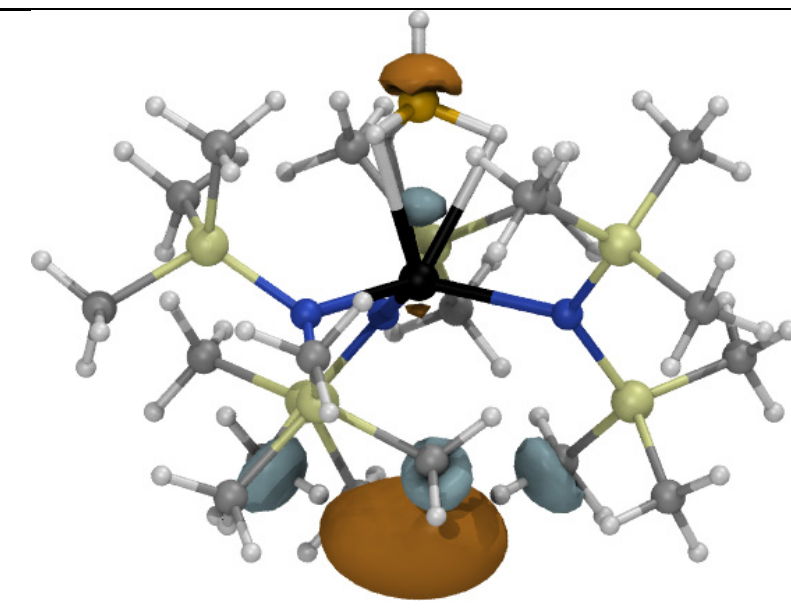 50% α, 50% β
36	192.427	0.001929	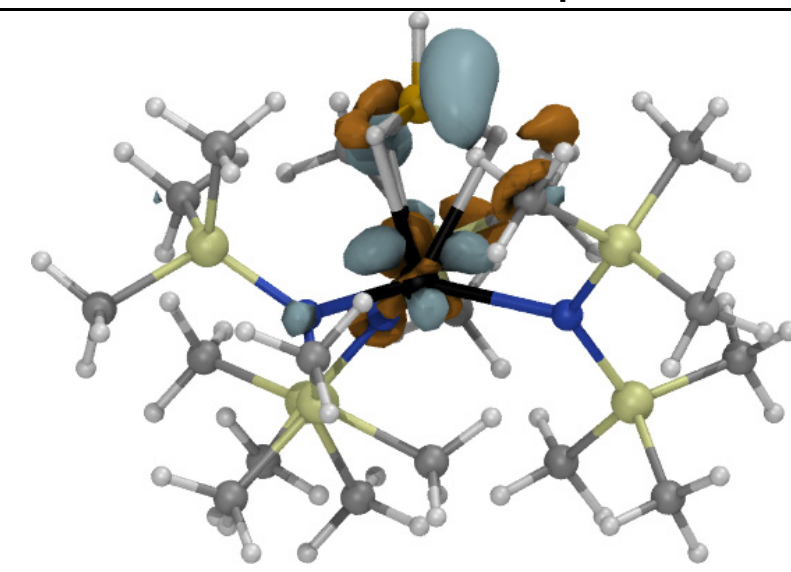 50% α, 50% β
37	192.428	0.001902	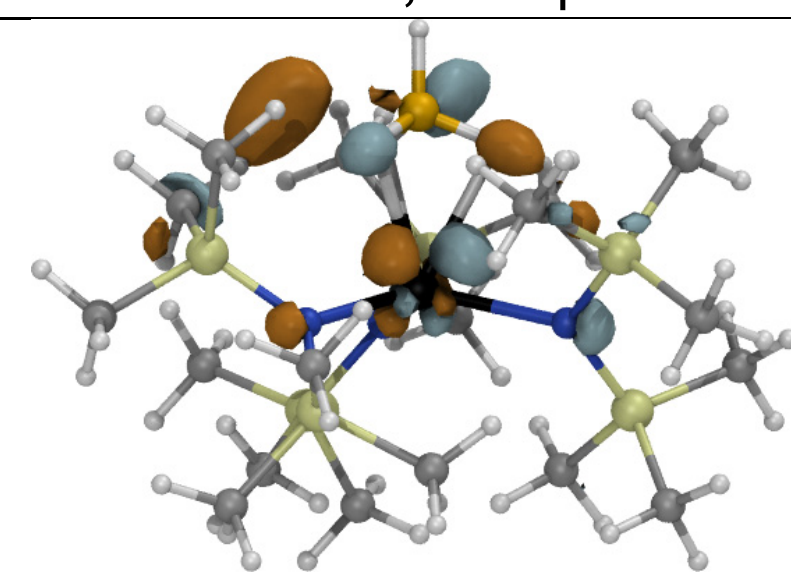 50% α, 50% β
38	192.441	0.000824	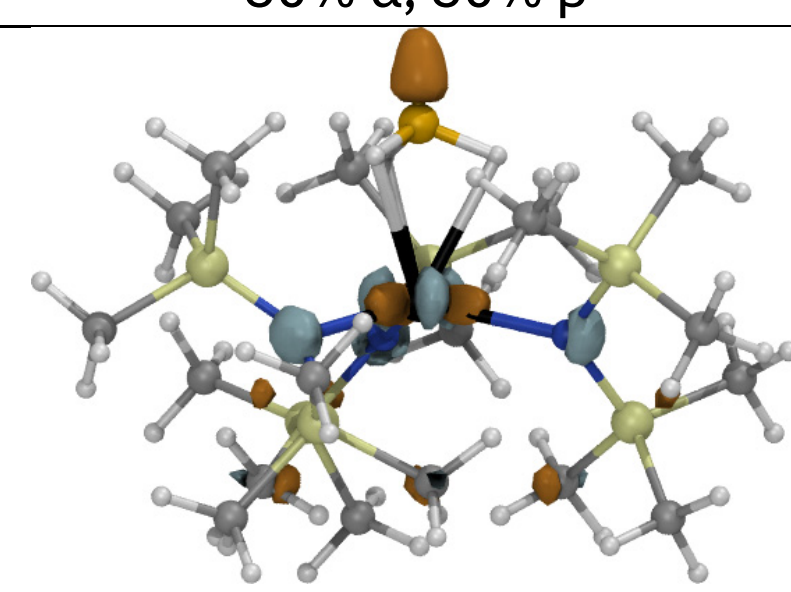 50% α, 50% β
41	192.552	0.002164	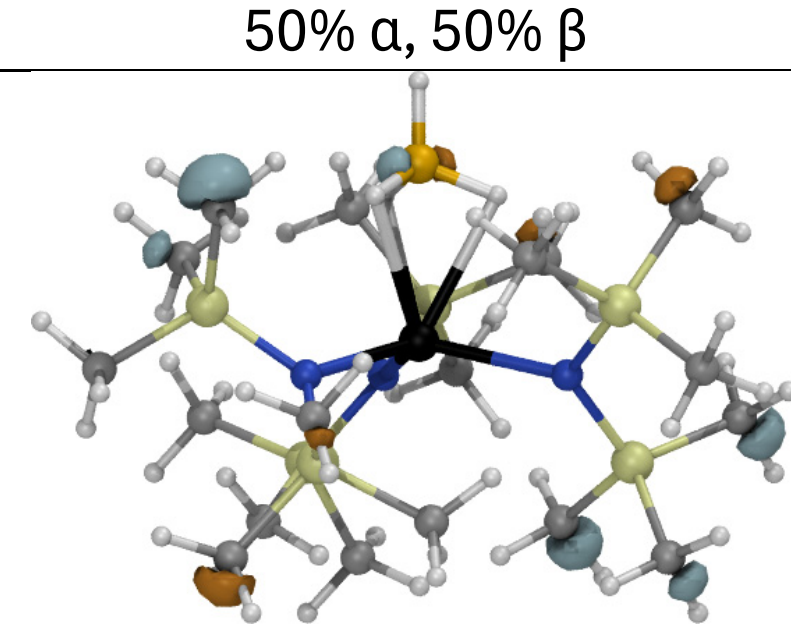 50% α, 50% β

42	192.552	0.002146	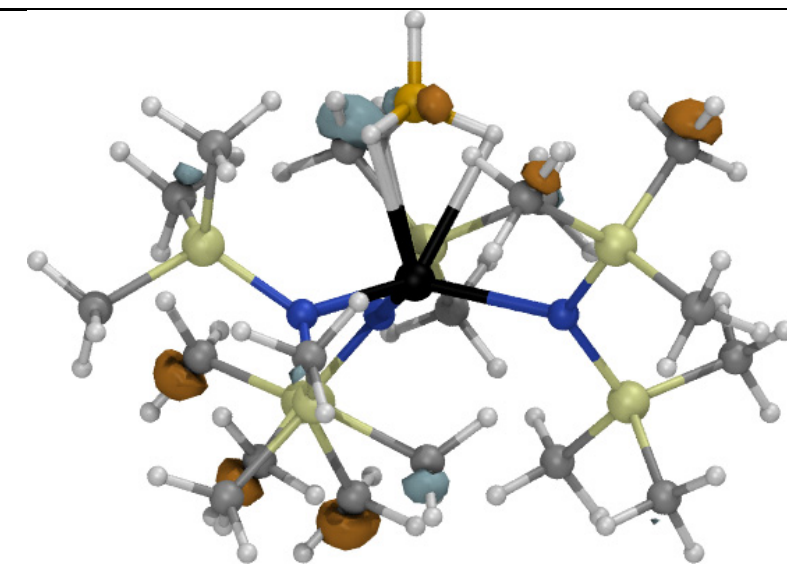 50% α, 50% β
44	192.692	0.000192	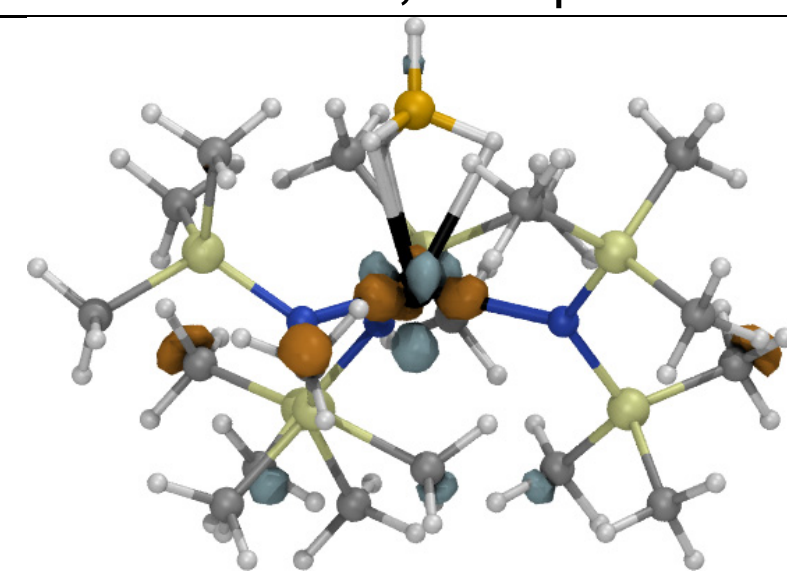 50% α, 50% β
47	192.707	0.000225	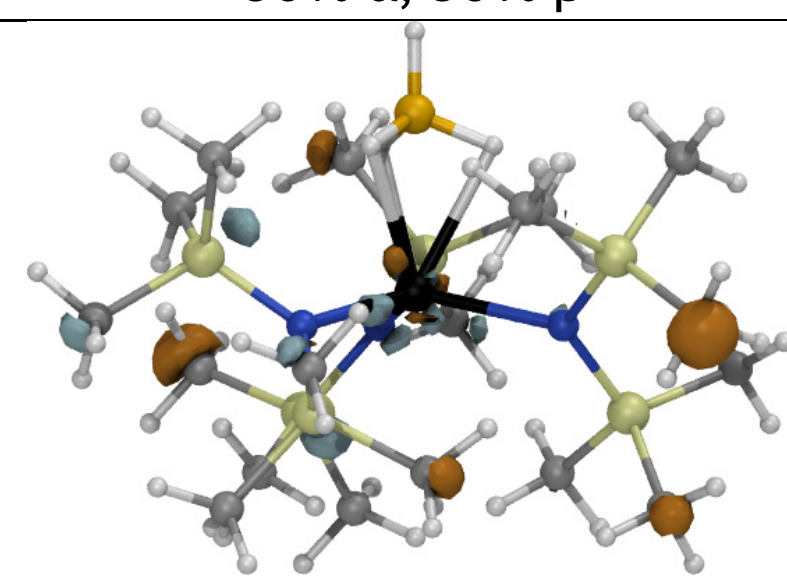 50% α, 50% β
48	192.708	0.000226	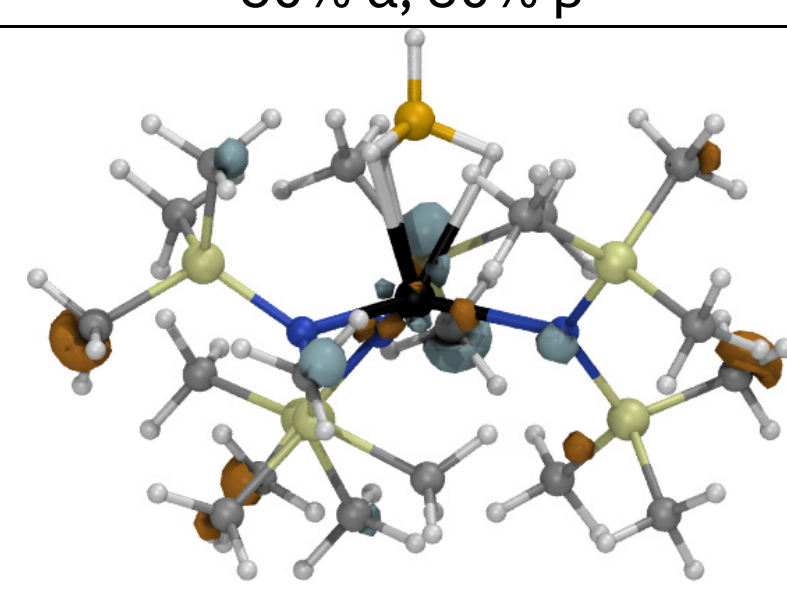 50% α, 50% β
50	192.874	0.001014	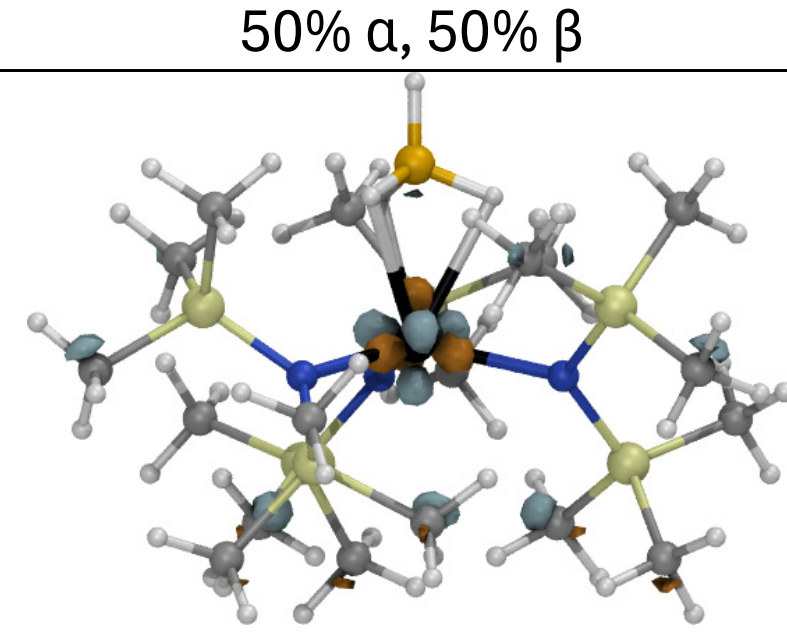 50% α, 50% β

54	192.953	0.000738	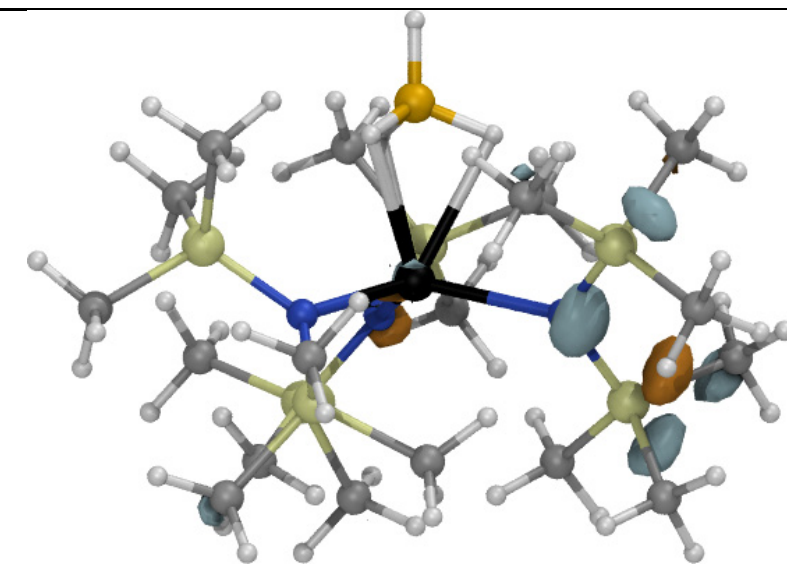 50% α, 50% β
55	192.955	0.000743	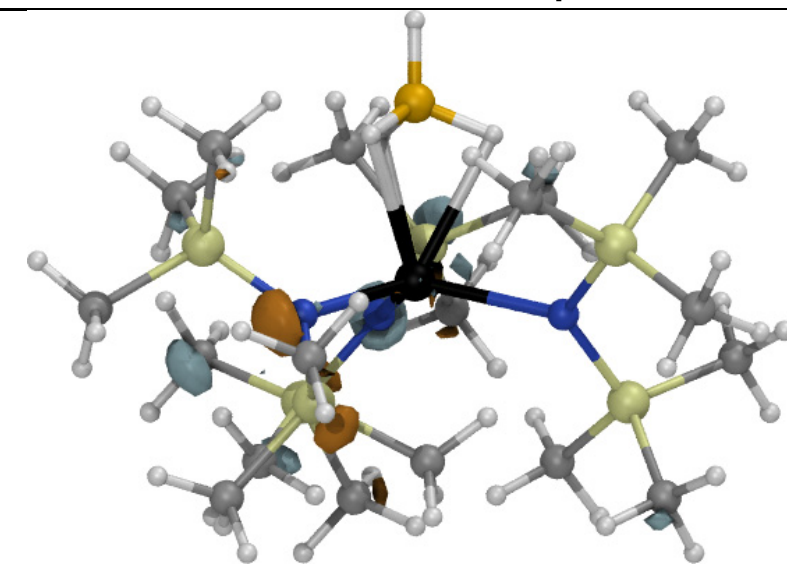 50% α, 50% β
56	192.956	0.000595	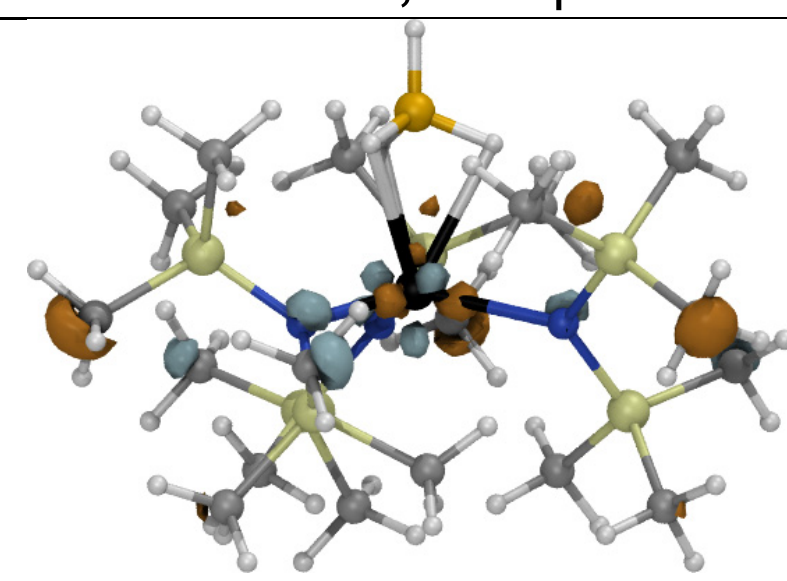 50% α, 50% β
61	193.237	0.000461	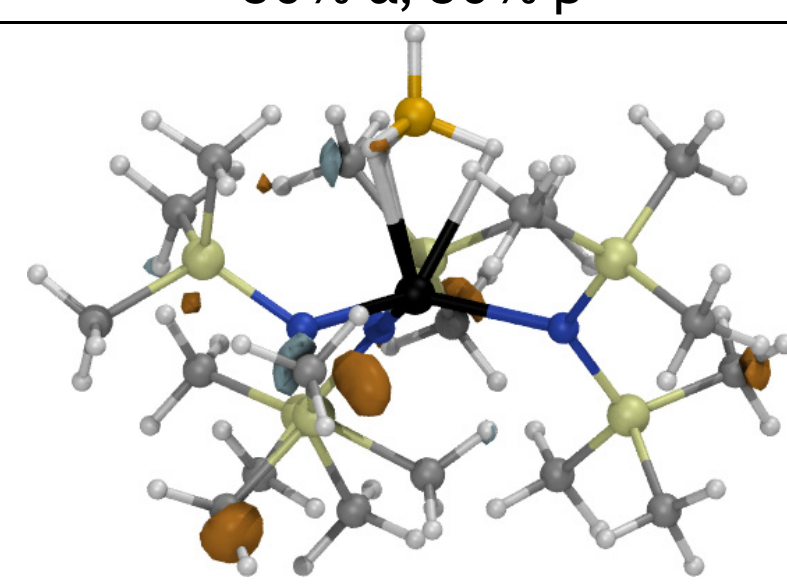 50% α, 50% β
62	193.238	0.000471	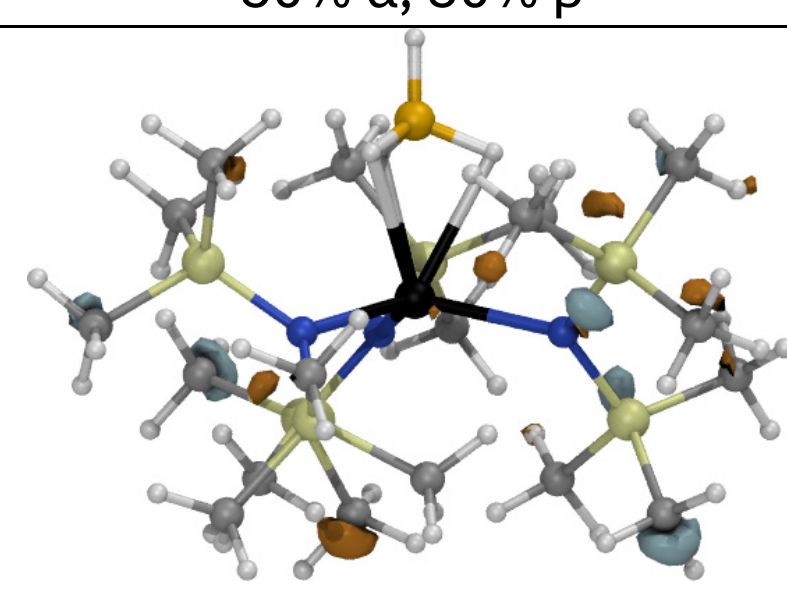 50% α, 50% β

66	193.336	0.000279	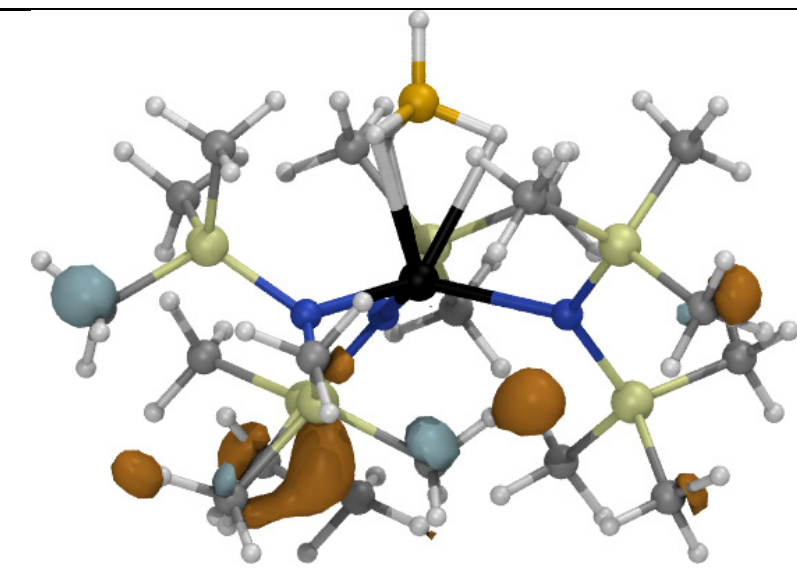 50% α, 50% β
67	193.337	0.000280	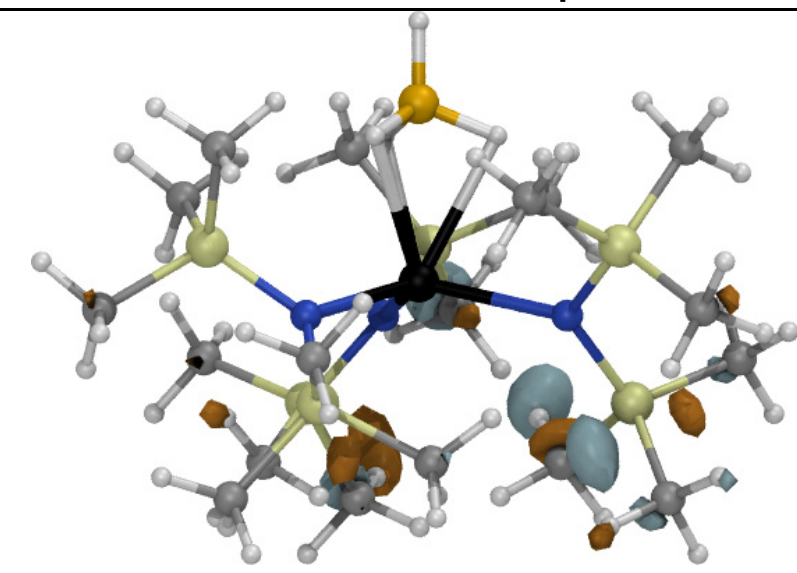 50% α, 50% β
68	193.395	0.005271	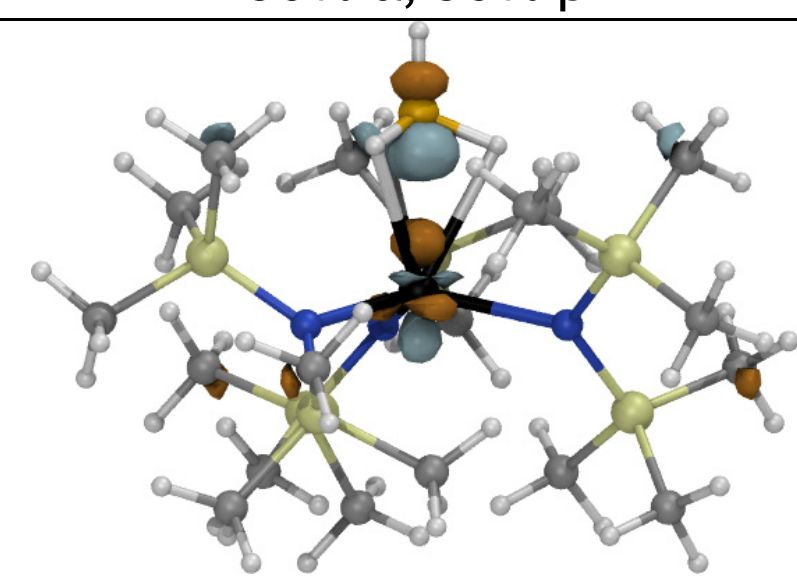 50% α, 50% β
70	193.468	0.000357	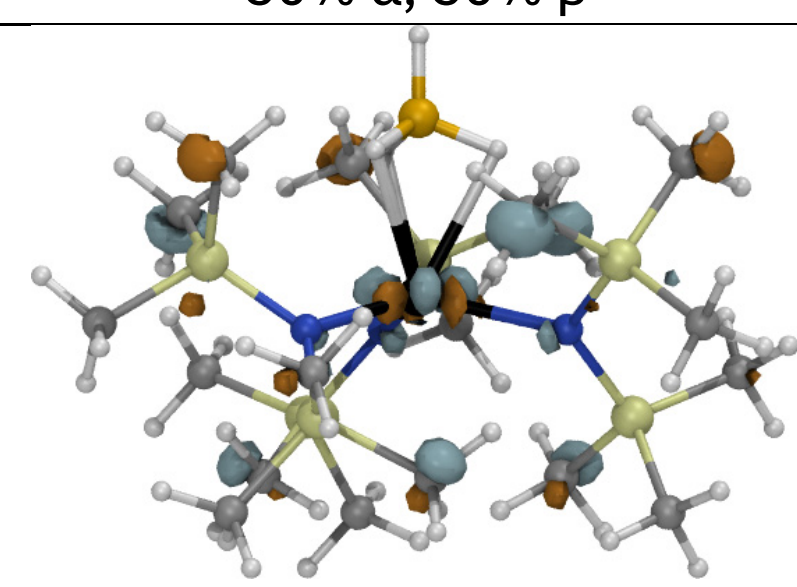 50% α, 50% β
79	193.693	0.000915	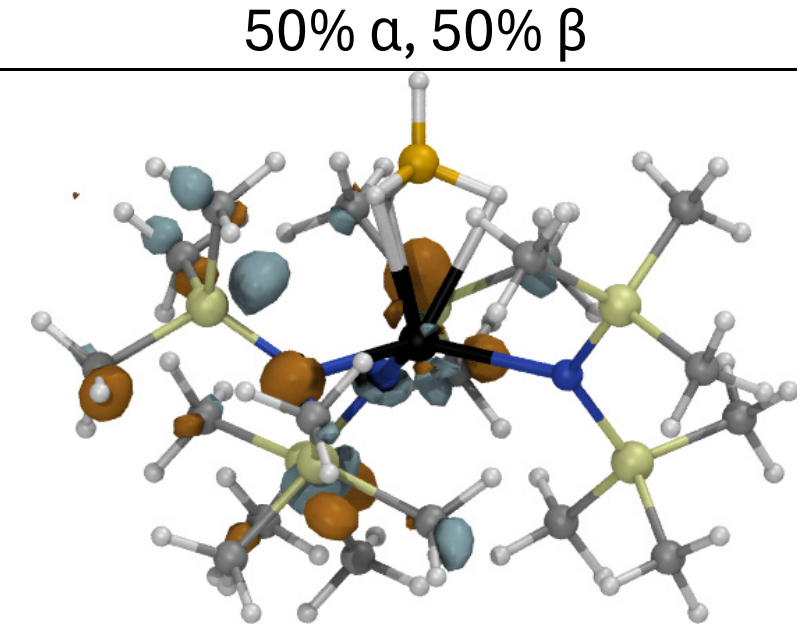 50% α, 50% β

80	193.694	0.000932	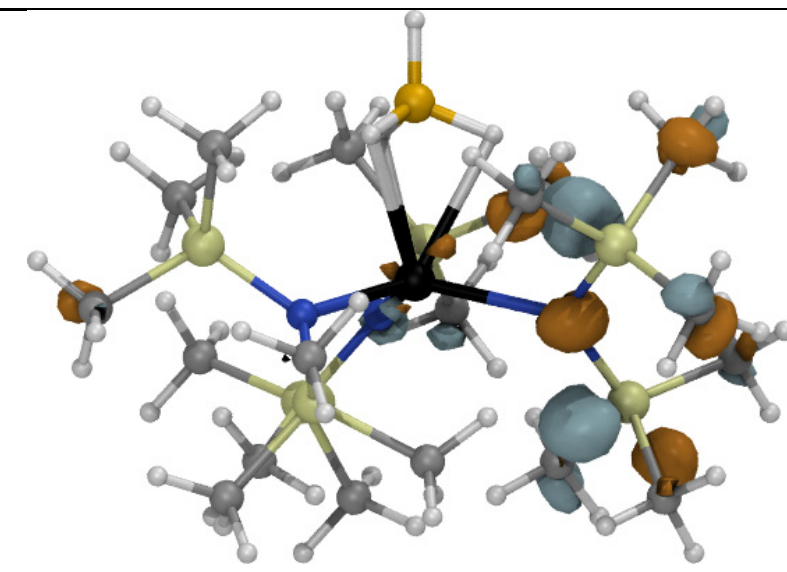 50% α, 50% β
84	193.887	0.000270	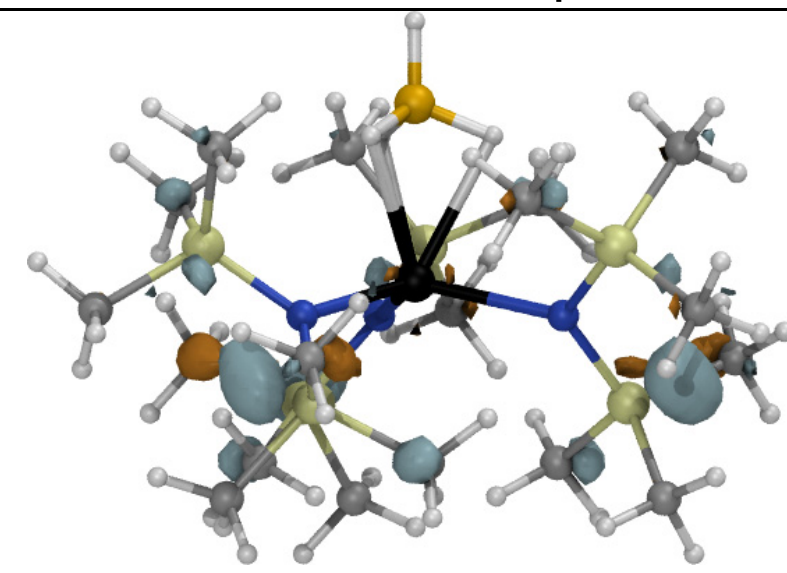 50% α, 50% β
86	193.962	0.003162	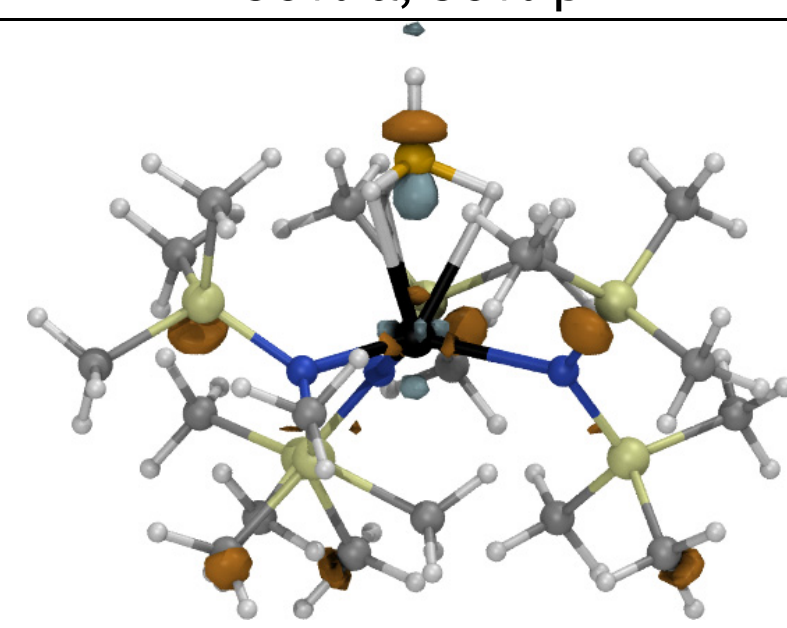 50% α, 50% β
89	194.080	0.000893	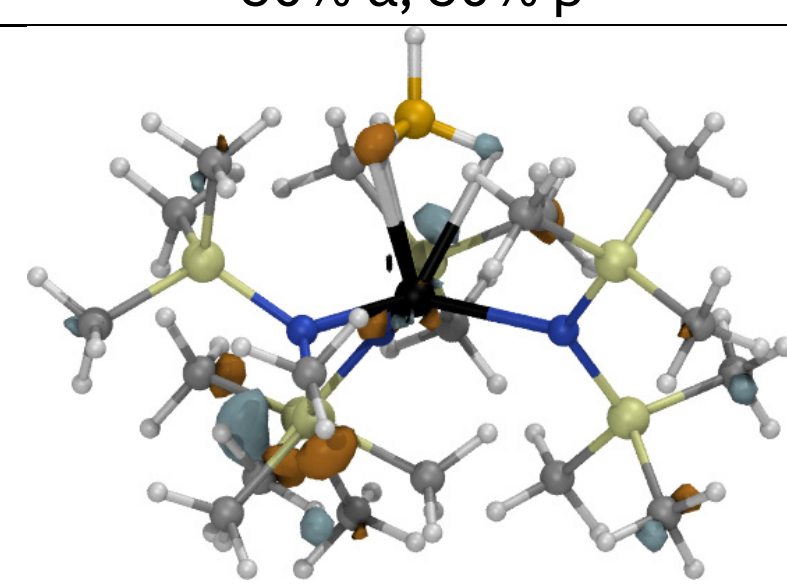 50% α, 50% β
90	194.081	0.000877	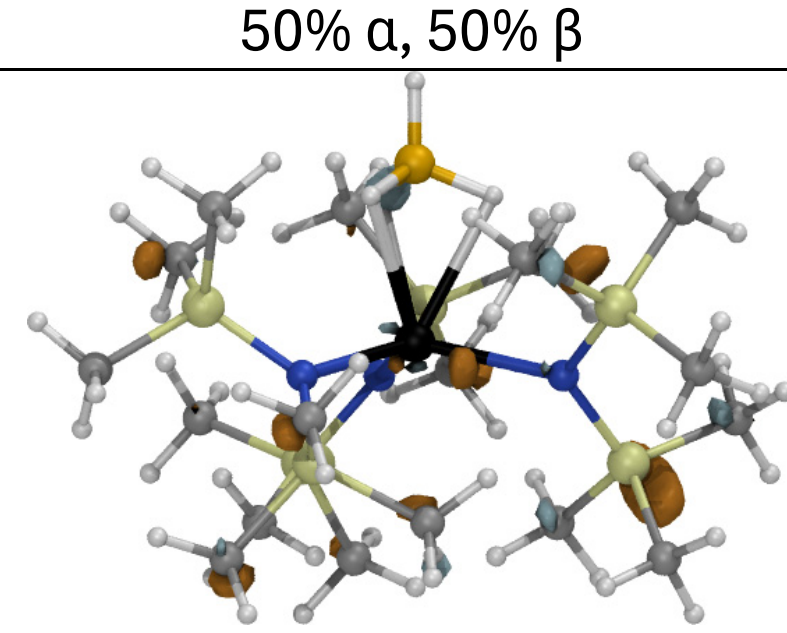 50% α, 50% β

93	194.175	0.000970	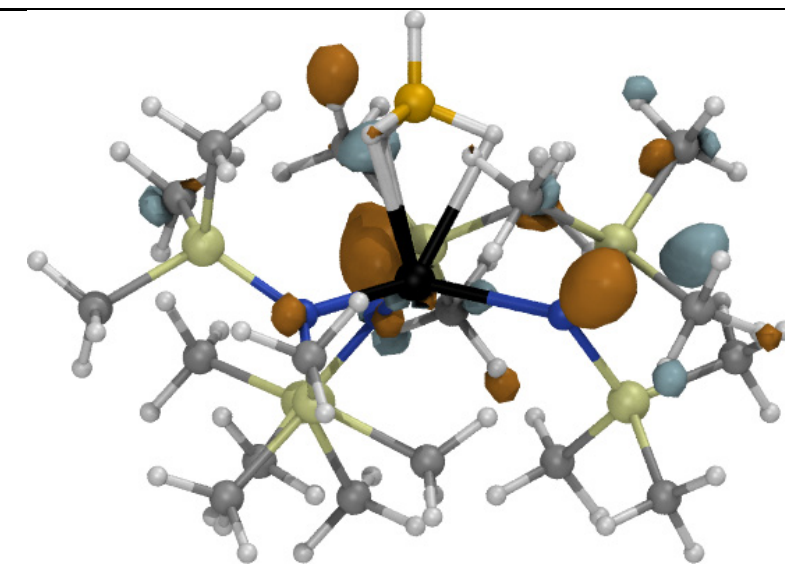 50% α, 50% β
94	194.177	0.001018	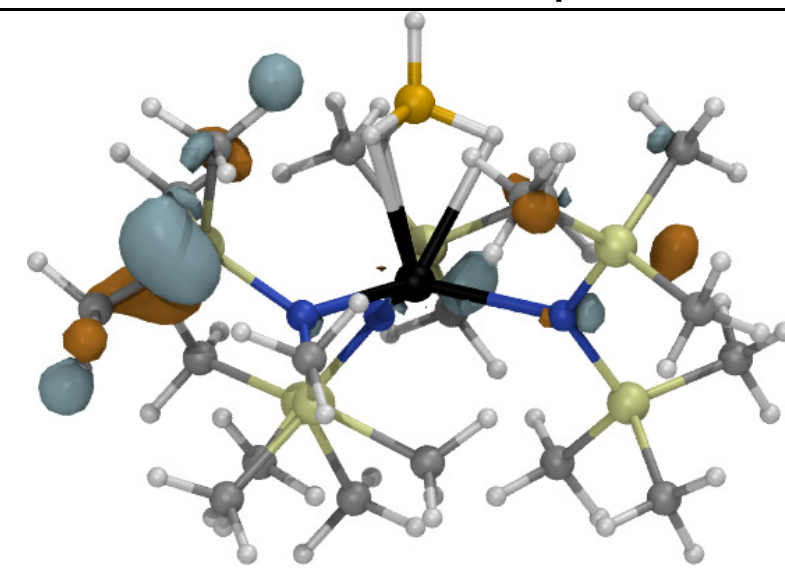 50% α, 50% β
98	194.222	0.001856	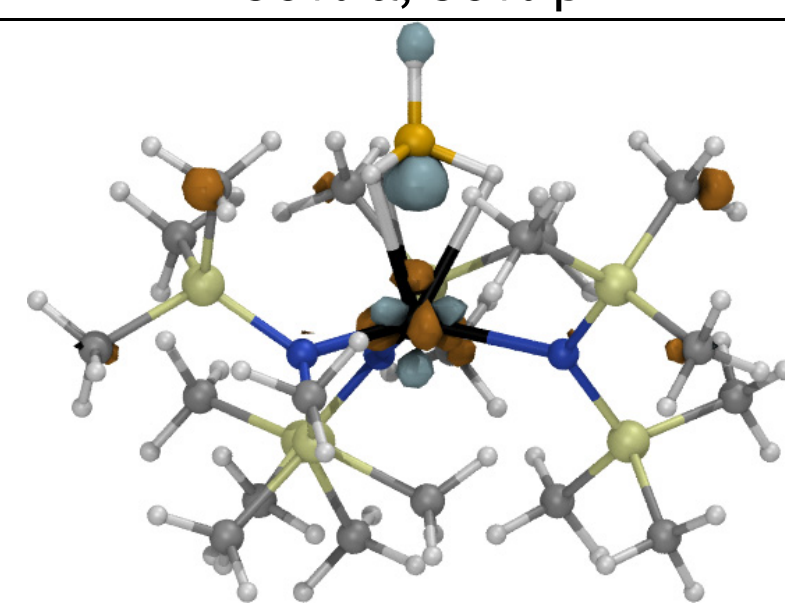 50% α, 50% β
99	194.233	0.001047	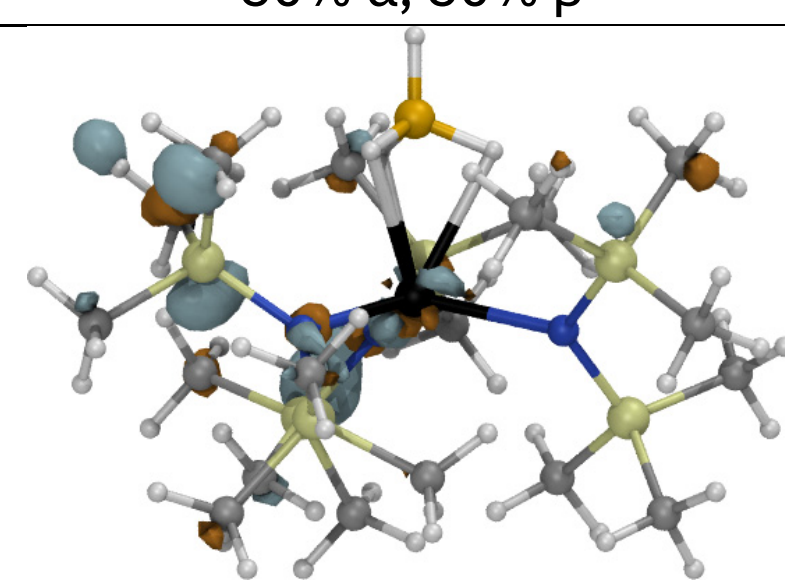 50% α, 50% β
100	194.235	0.001101	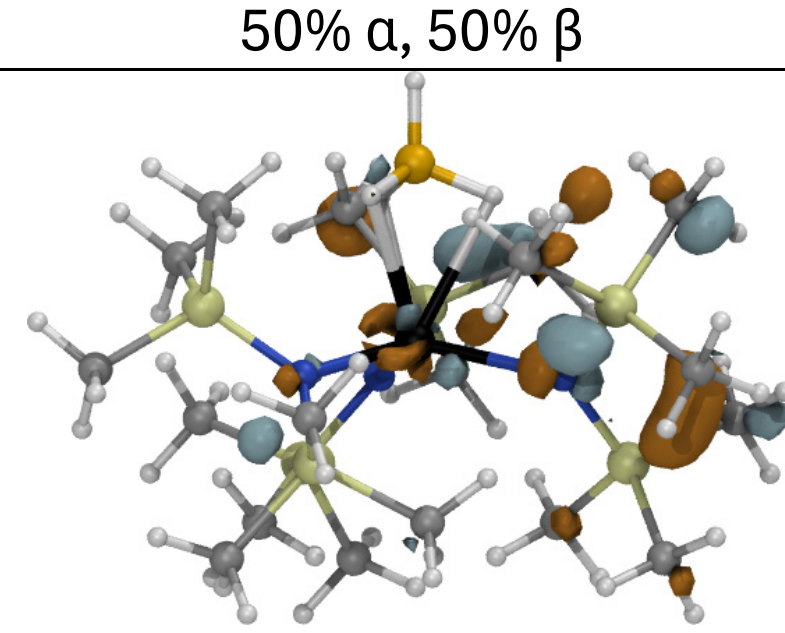 50% α, 50% β

Table S15. Natural Transition Orbitals (NTOs) accepting the B 1s orbital based on the B3LYP-D3 density on the PBE geometry of **2** (M = U). Acceptor orbitals and their corresponding states with intensities larger than 0.00005 a.u. displayed. Energies are shifted by 9.3 eV.

State	Energy	Intensity	Acceptor Orbital
1	188.038	0.000182	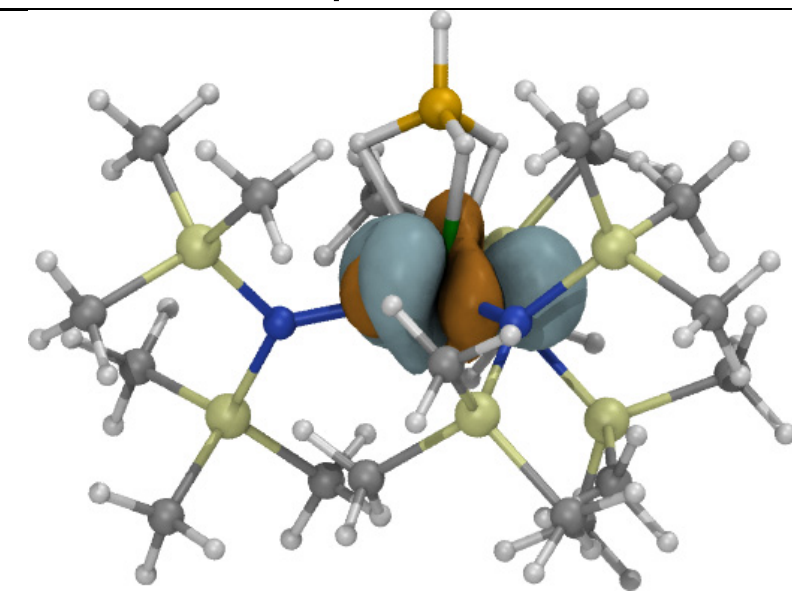 100.0%
2	188.280	0.000379	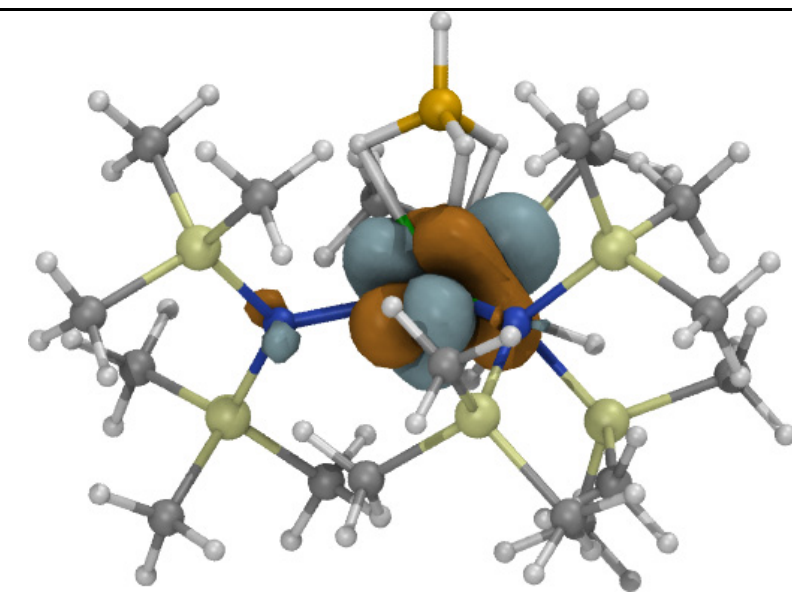 100.0%
3	188.302	0.000051	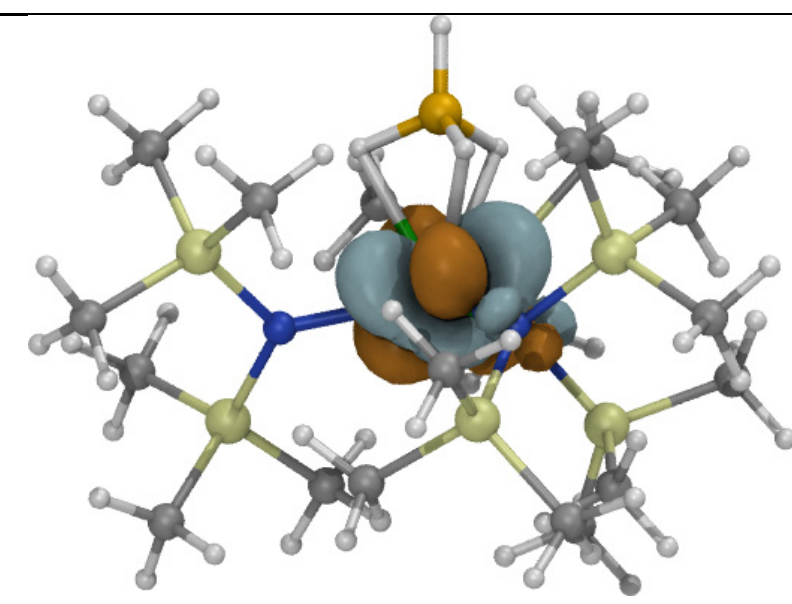 100.0%
5	188.776	0.000190	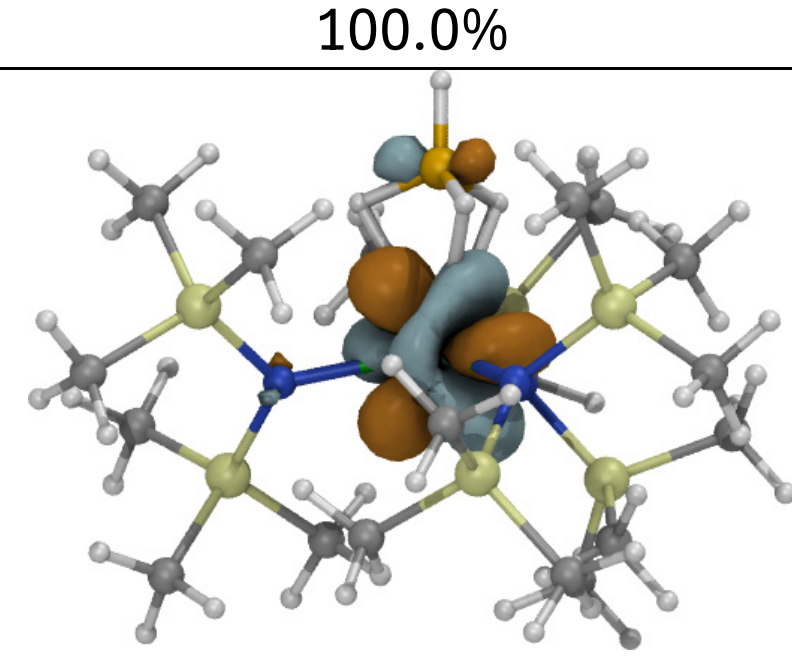 99.8%

6	188.926	0.000923	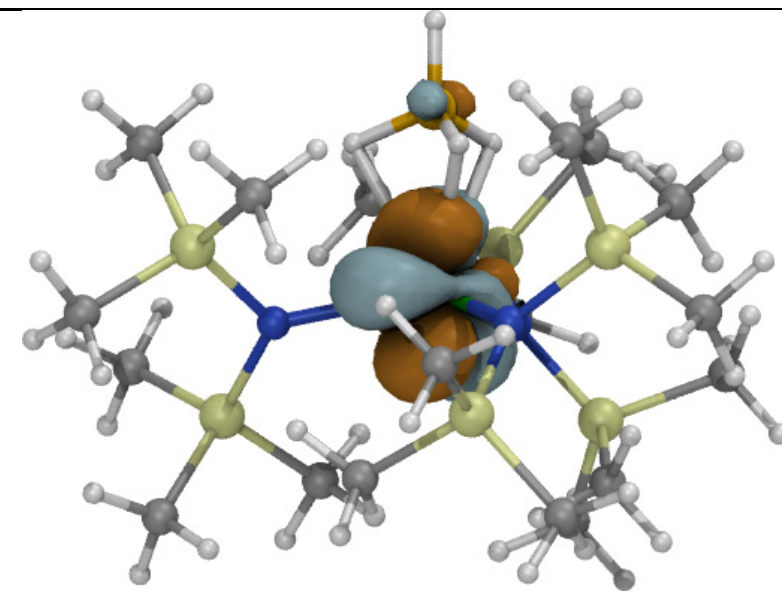 99.1%
7	188.945	0.000685	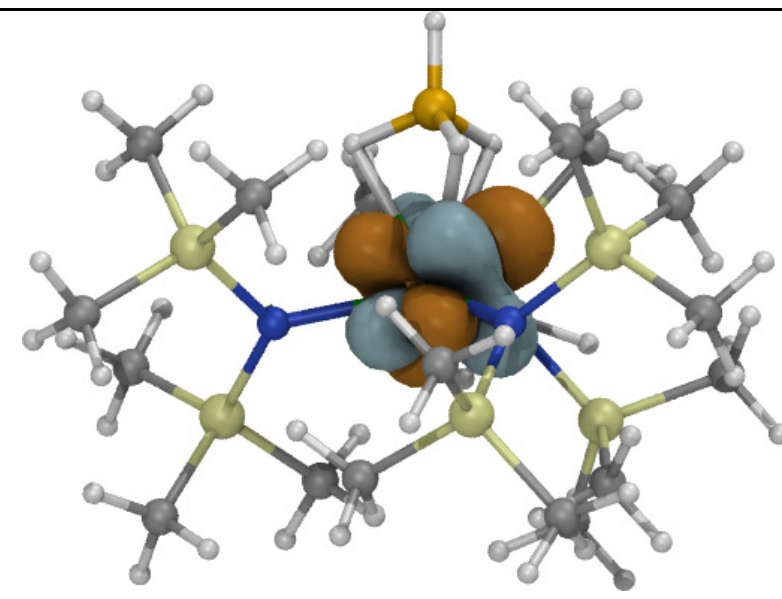 99.1%
10	189.041	0.000574	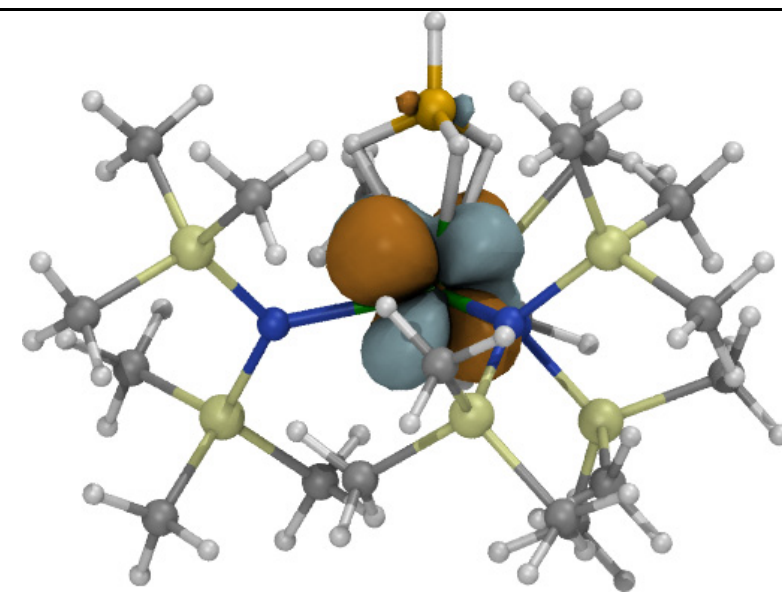 99.9%
11	189.356	0.000207	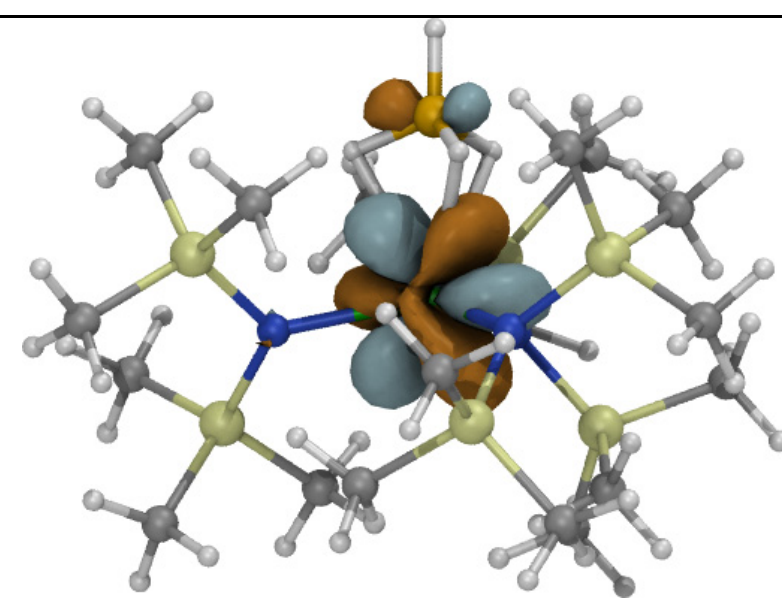 99.8%
12	189.362	0.000674	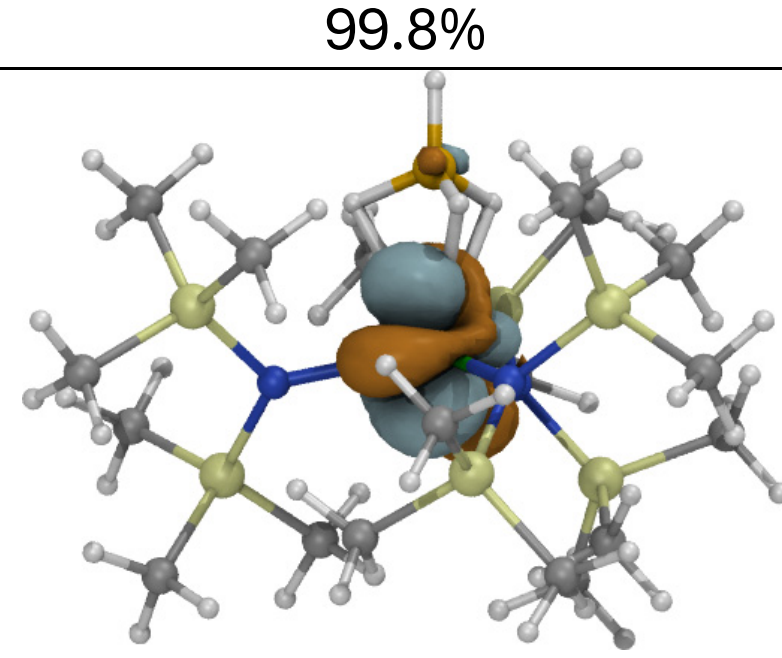 99.9%

14	189.503	0.001070	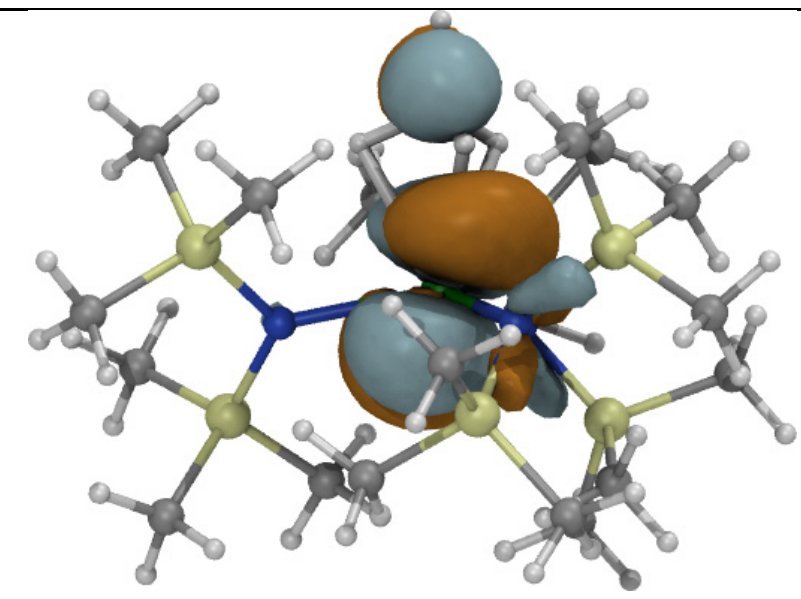<br>96.6%
15	189.556	0.000663	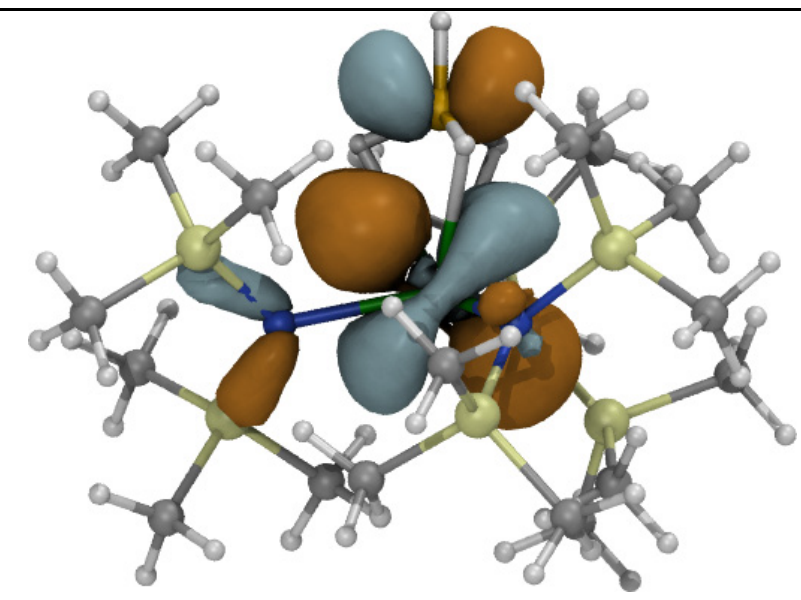<br>96.4%
16	190.610	0.003057	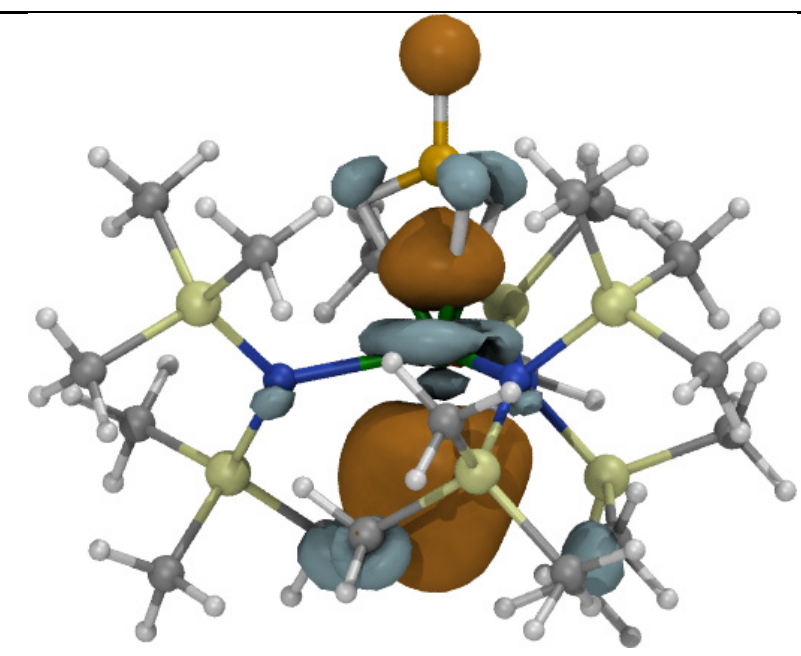<br>96.7%
18	190.618	0.003056	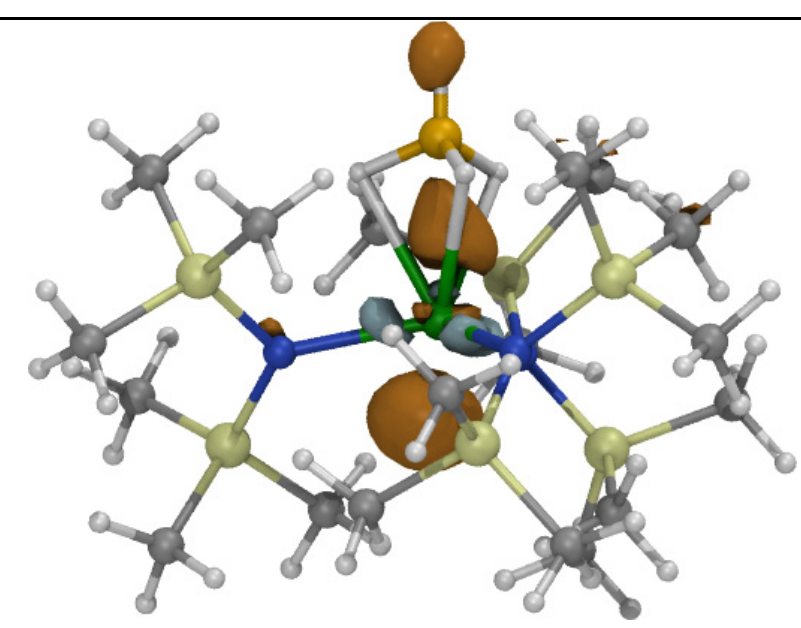<br>92.1%
19	190.821	0.000138	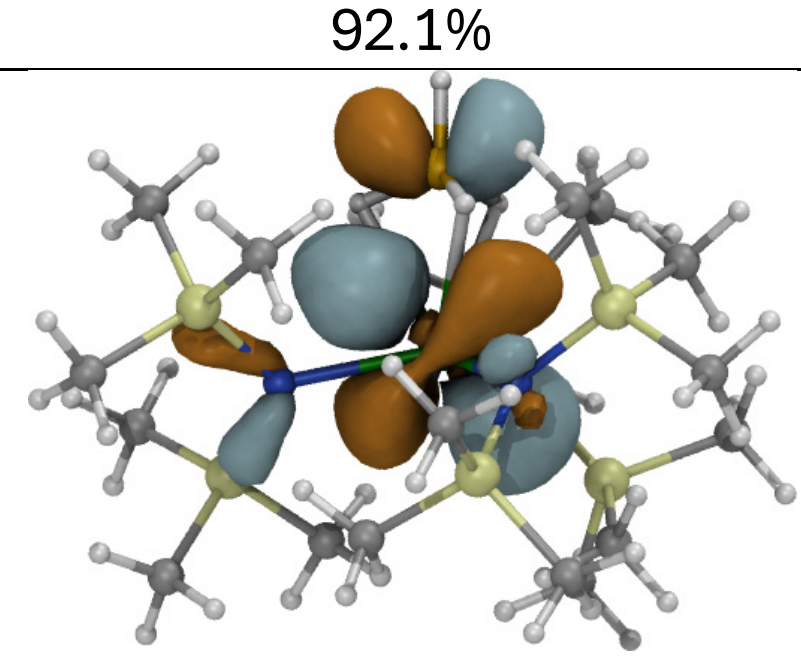<br>96.4%

20	190.972	0.000090	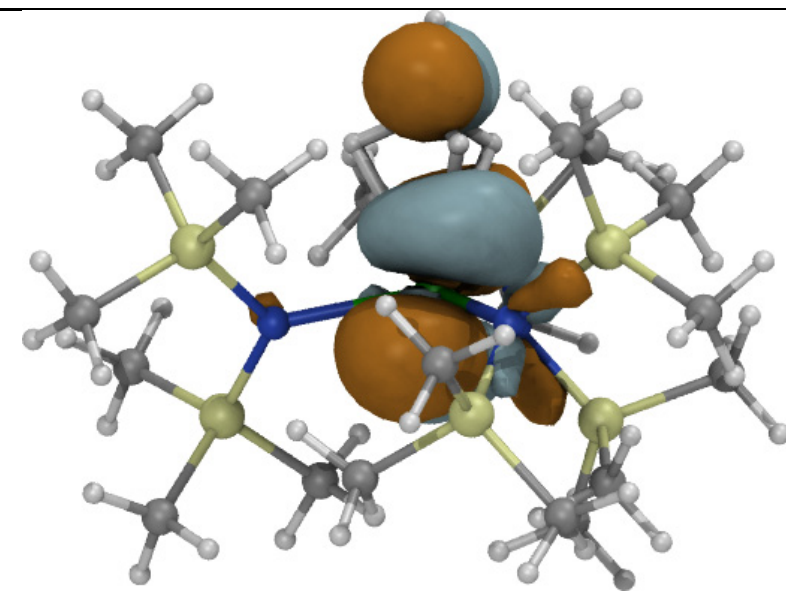<br>96.4%
21	190.996	0.005845	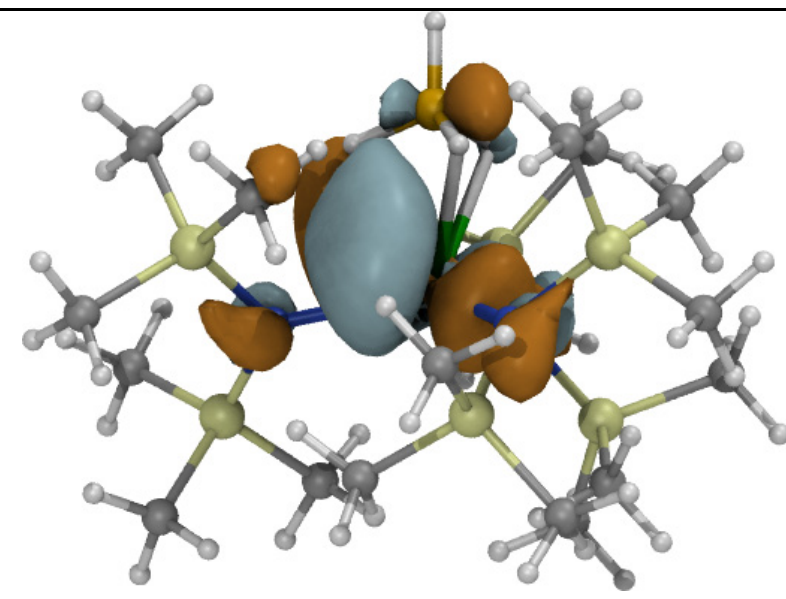<br>98.9%
22	190.999	0.006202	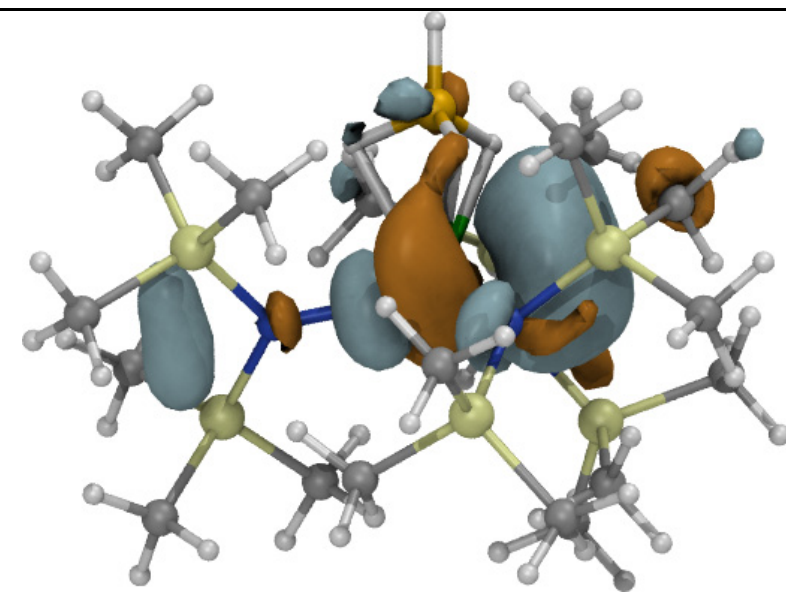<br>96.8%
23	191.739	0.000936	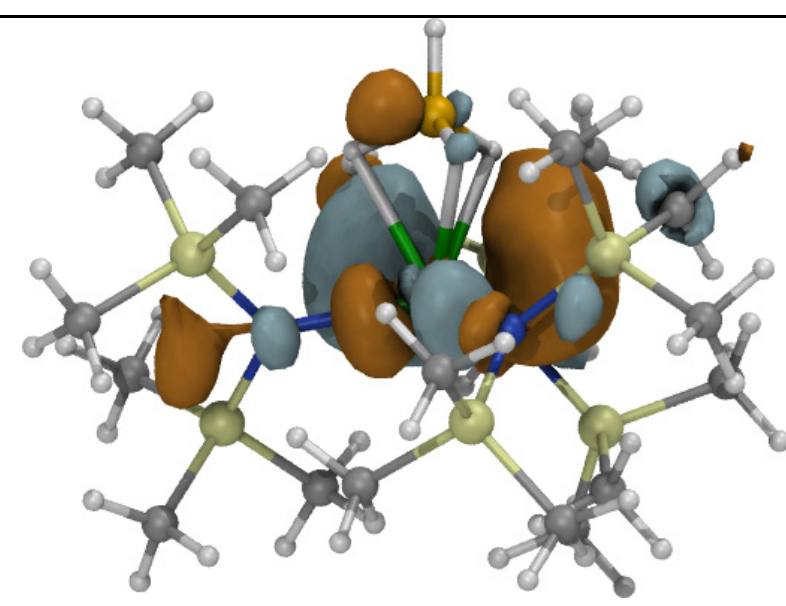<br>95.5%
24	191.824	0.000558	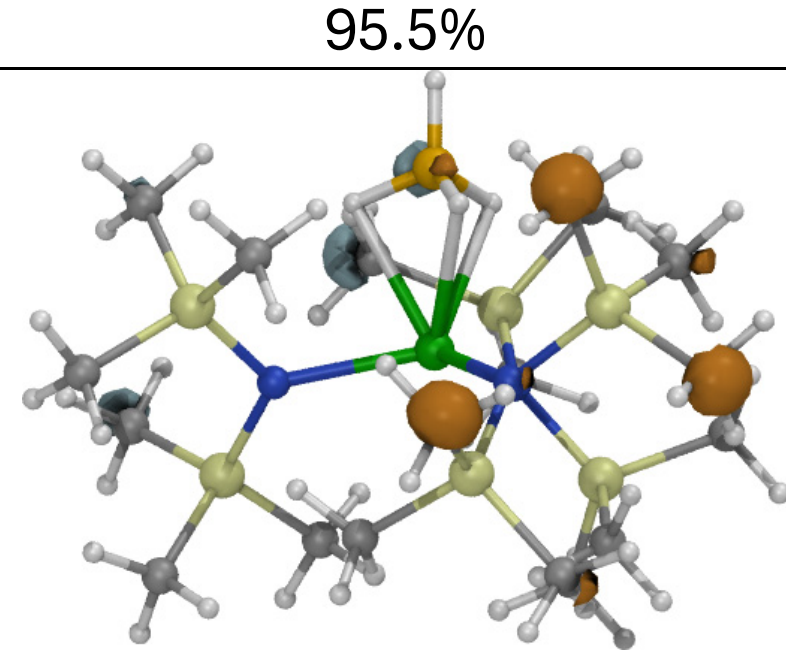<br>22.9%

			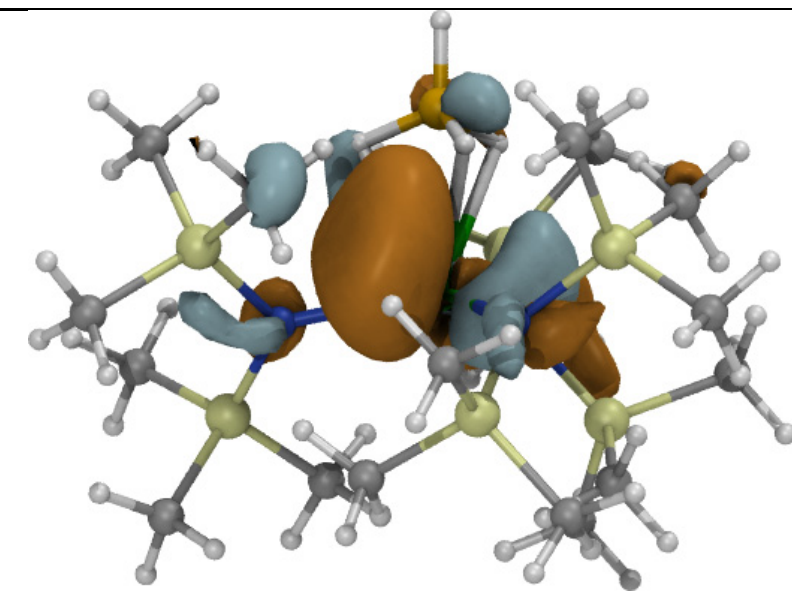 77.1%
25	191.898	0.001206	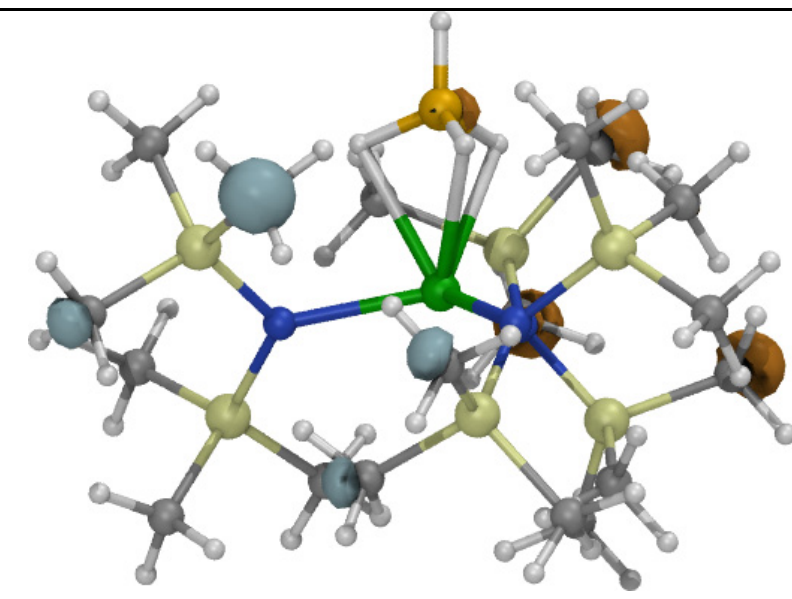 58.0%
			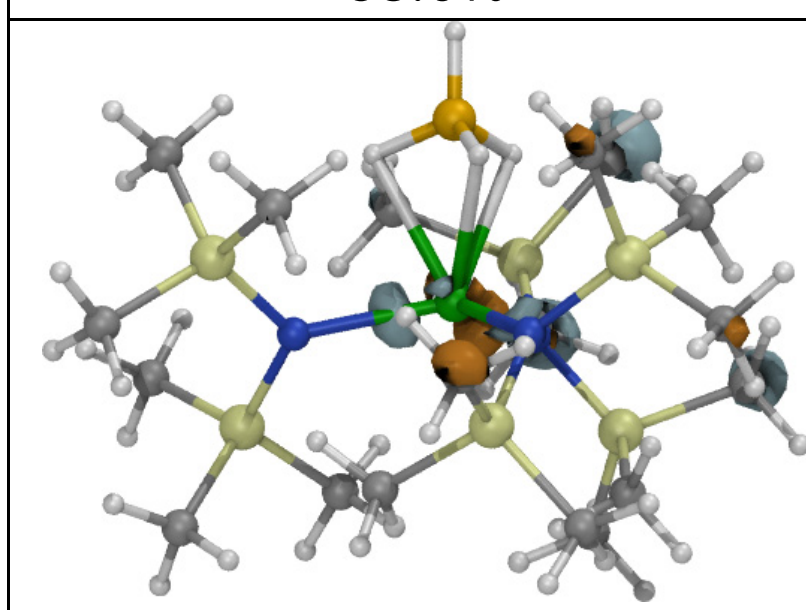 42.0%
26	191.975	0.000220	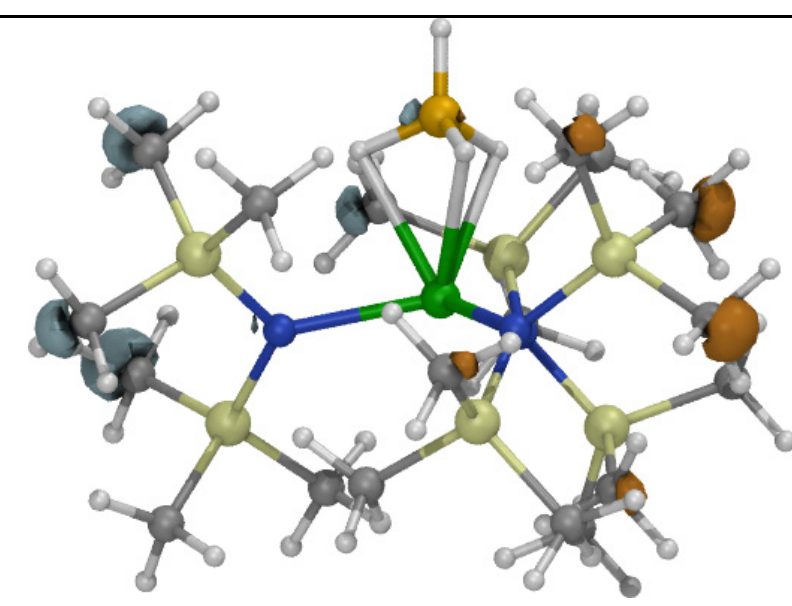 50.5%
			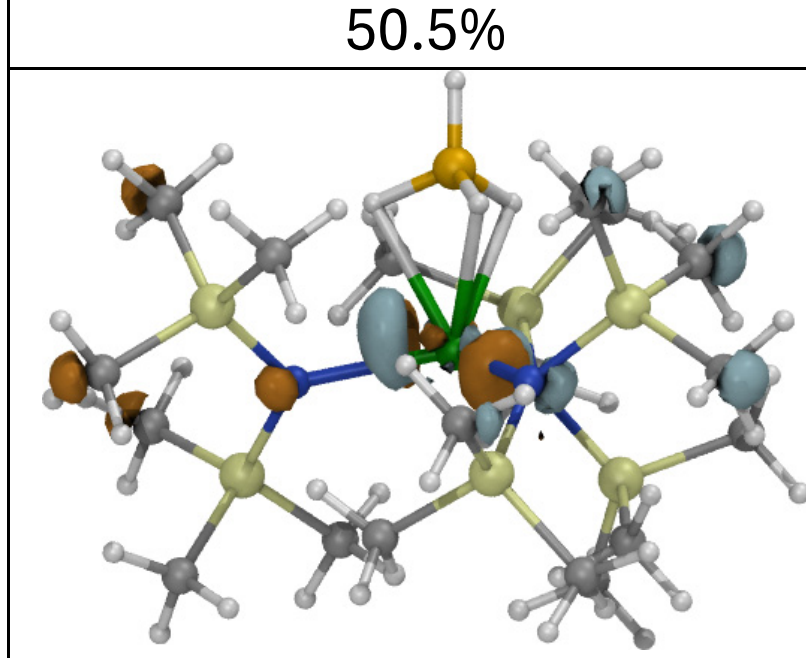 49.5%

27	191.989	0.000051	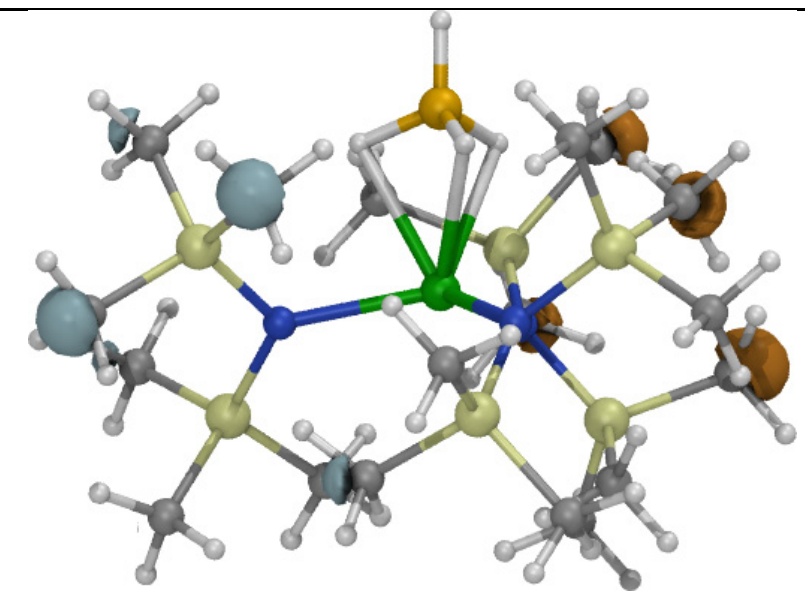<br>32.3%
			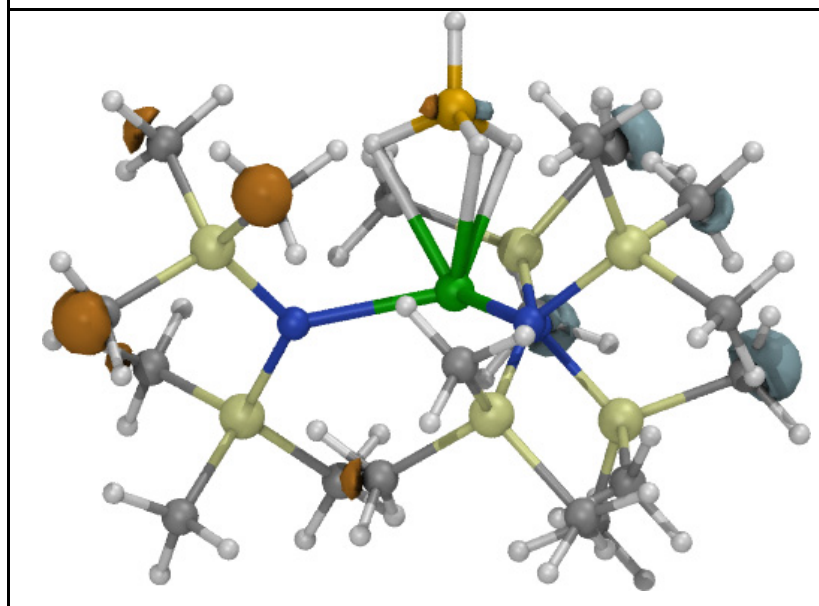<br>67.7%
28	191.994	0.000156	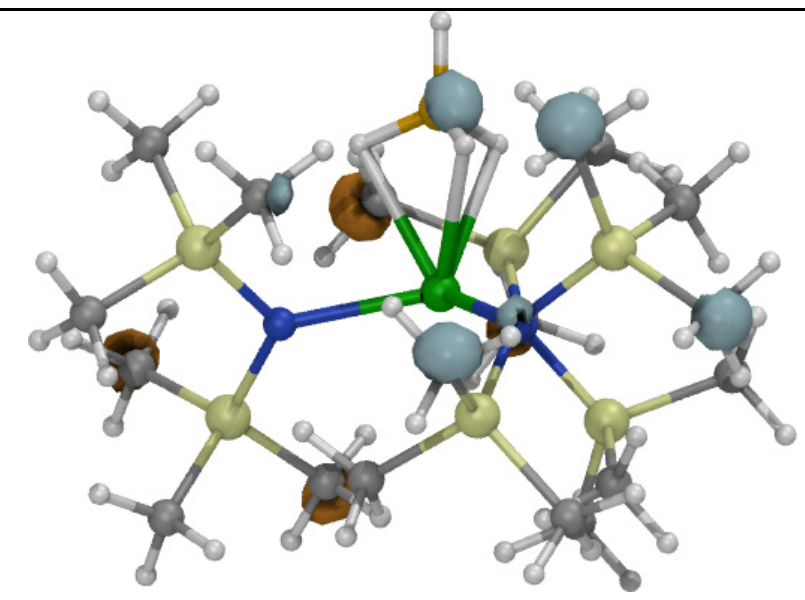<br>39.3%
			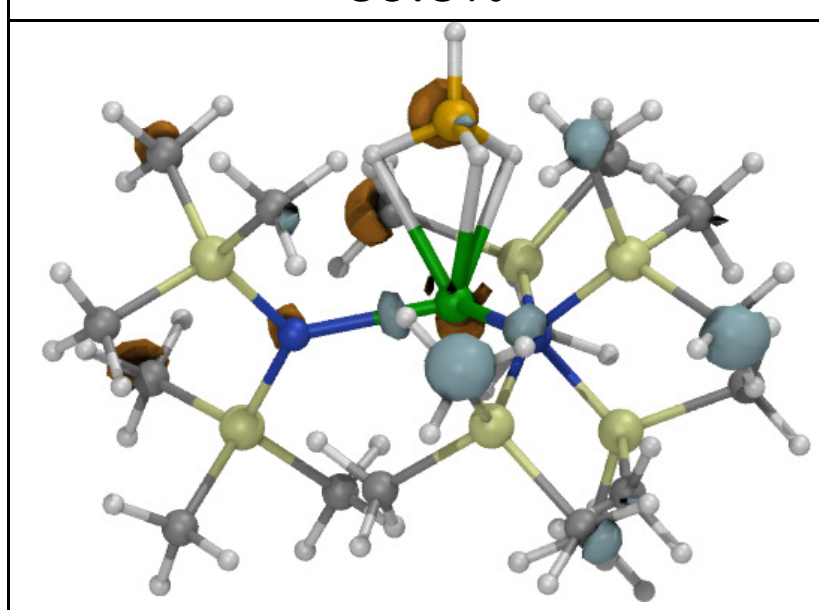<br>60.7%
29	192.009	0.001415	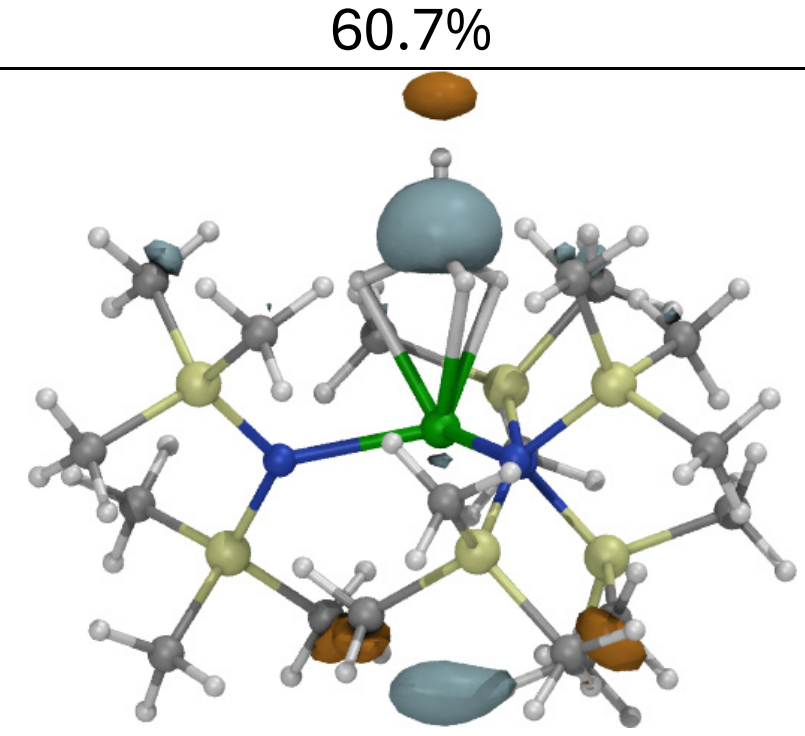<br>52.2%

			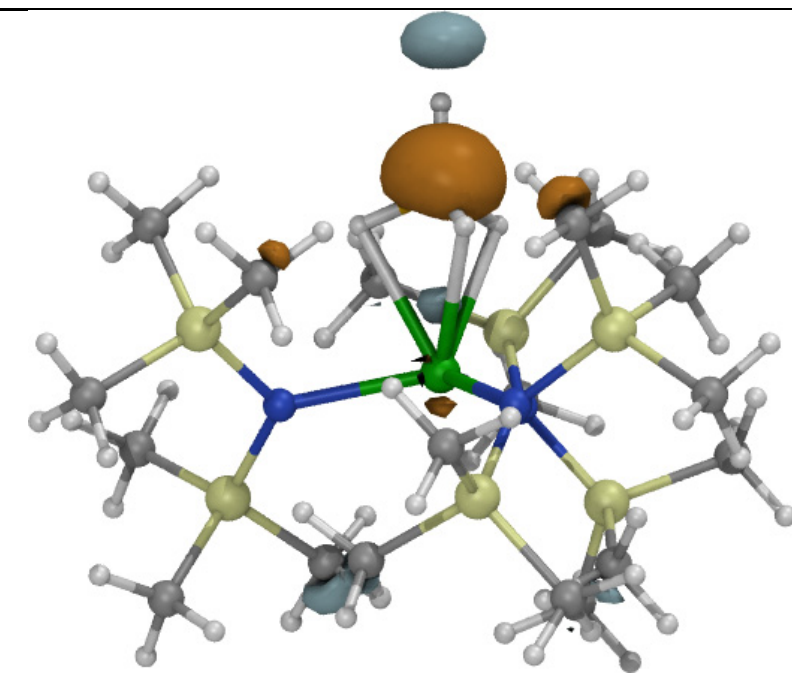 47.8%
30	192.014	0.001794	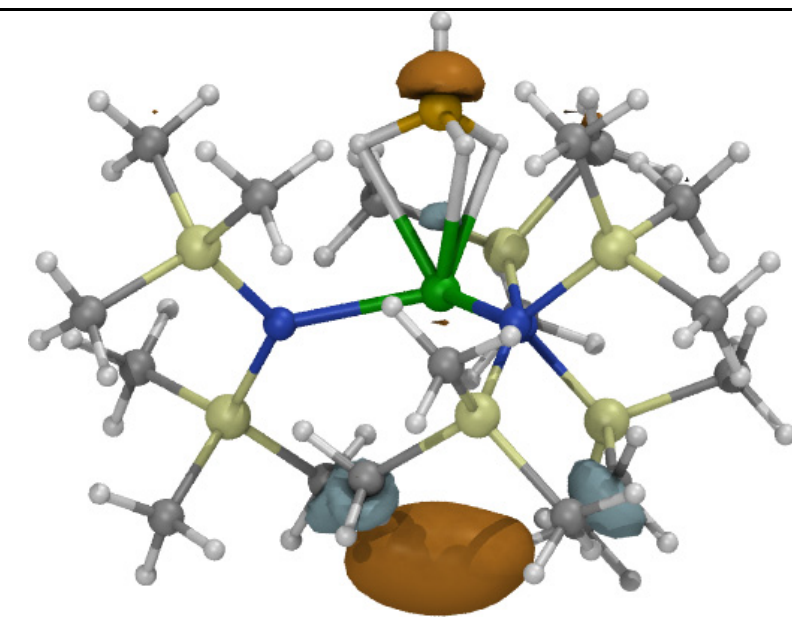 45.2%<br>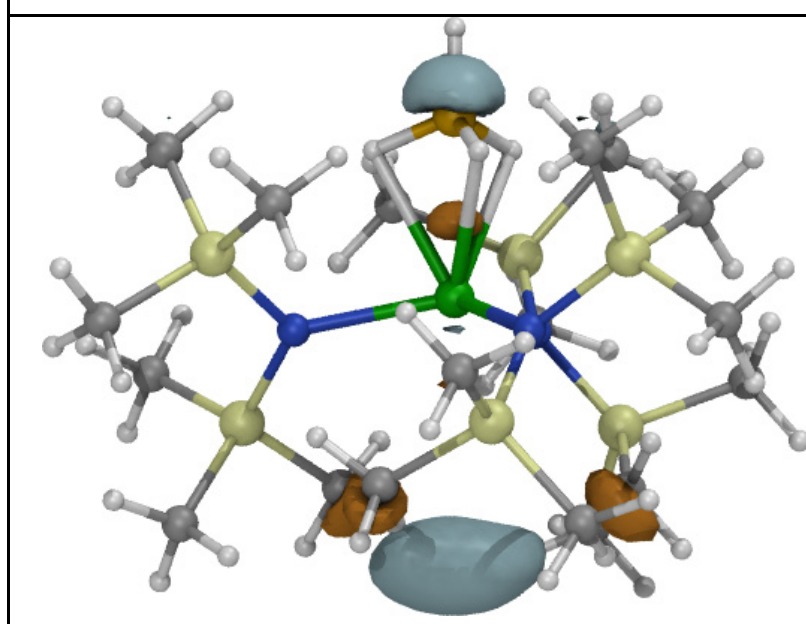 54.8%
31	192.017	0.000121	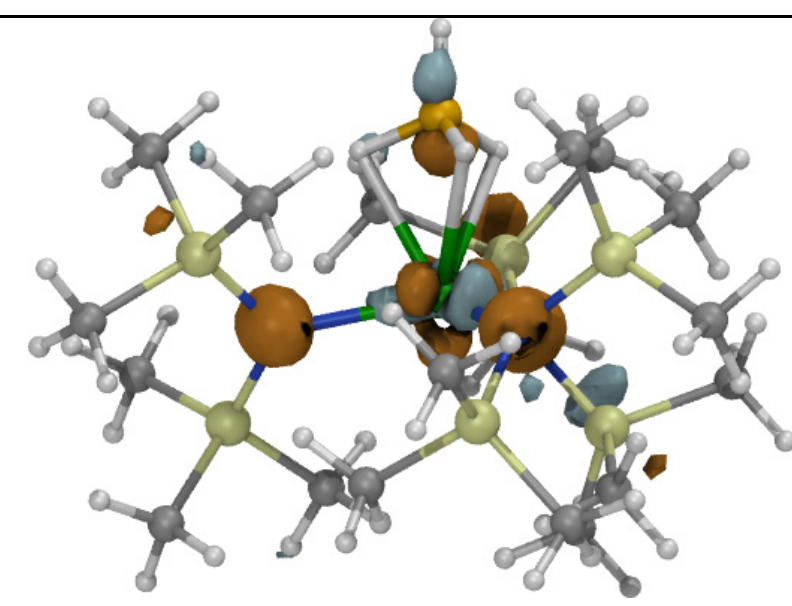 96.2%
32	192.220	0.001141	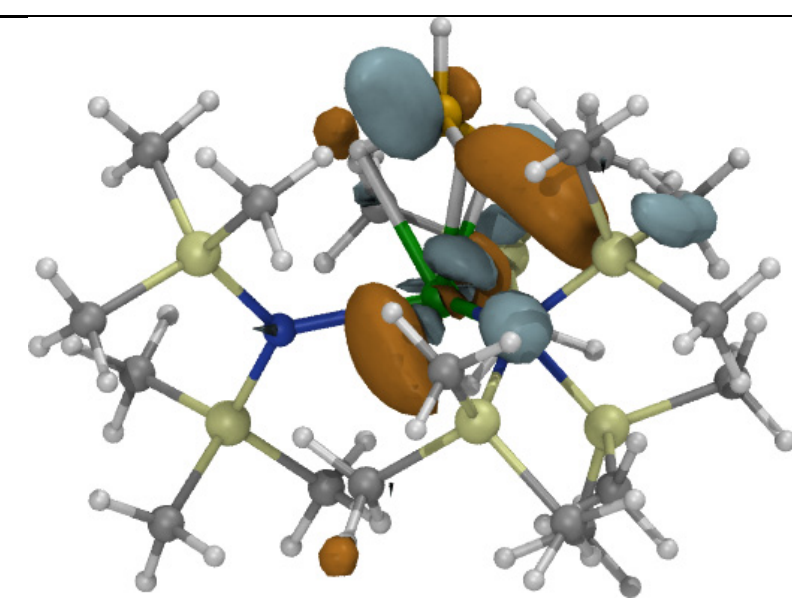 94.5%

33	192.353	0.000686	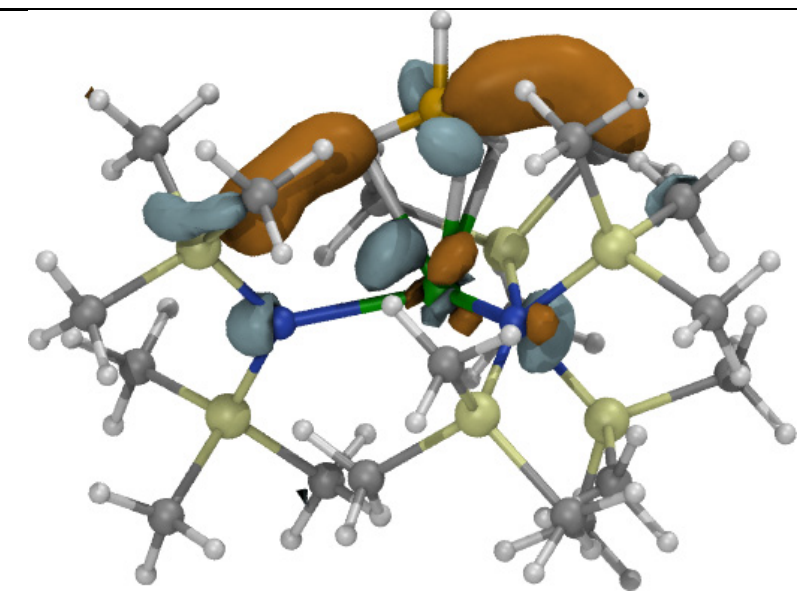<br>89.8%<br>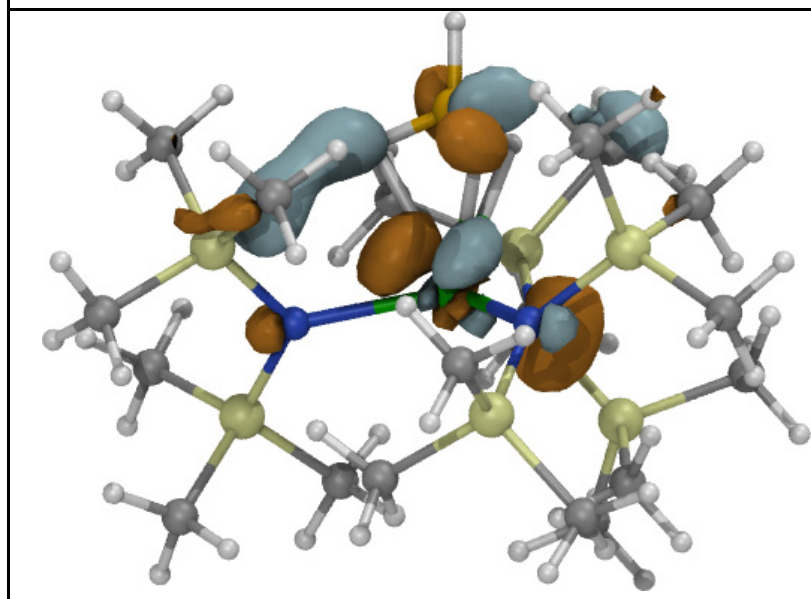<br>10.2%
34	192.359	0.001044	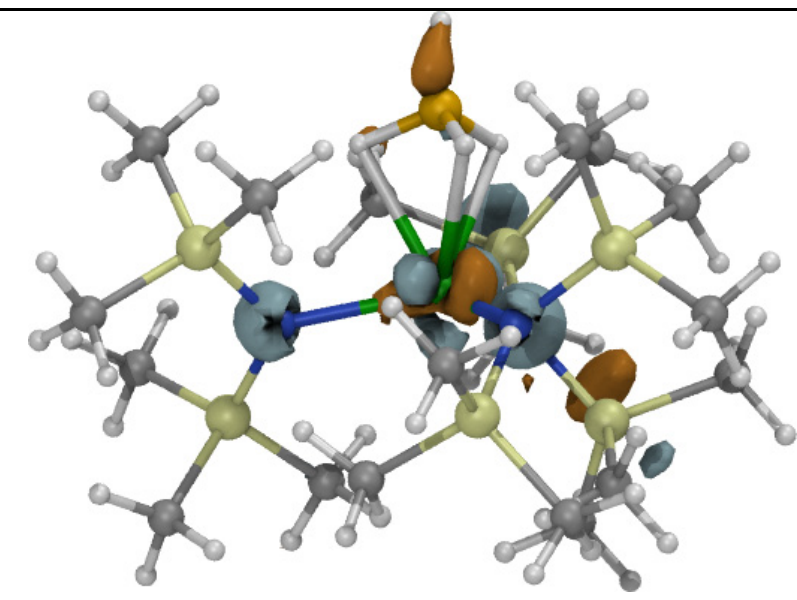<br>96.1%
35	192.401	0.000671	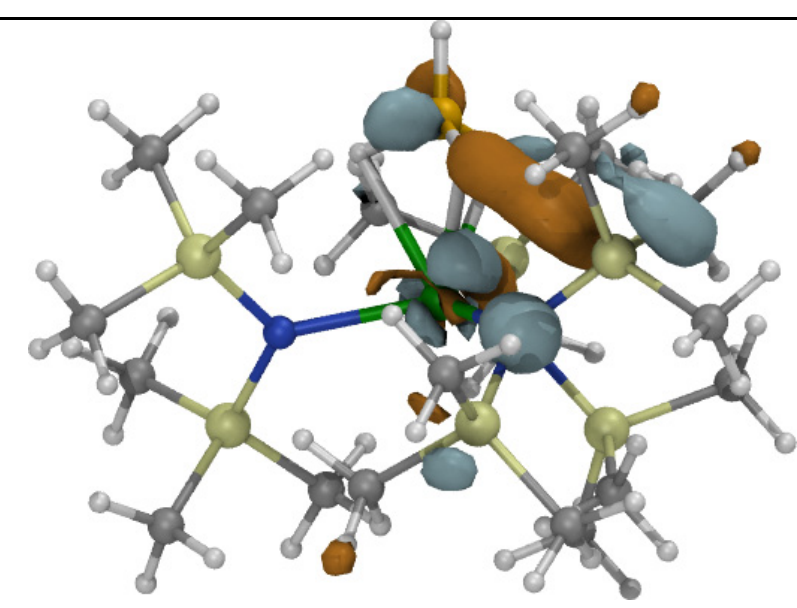<br>92.6%
36	192.430	0.001028	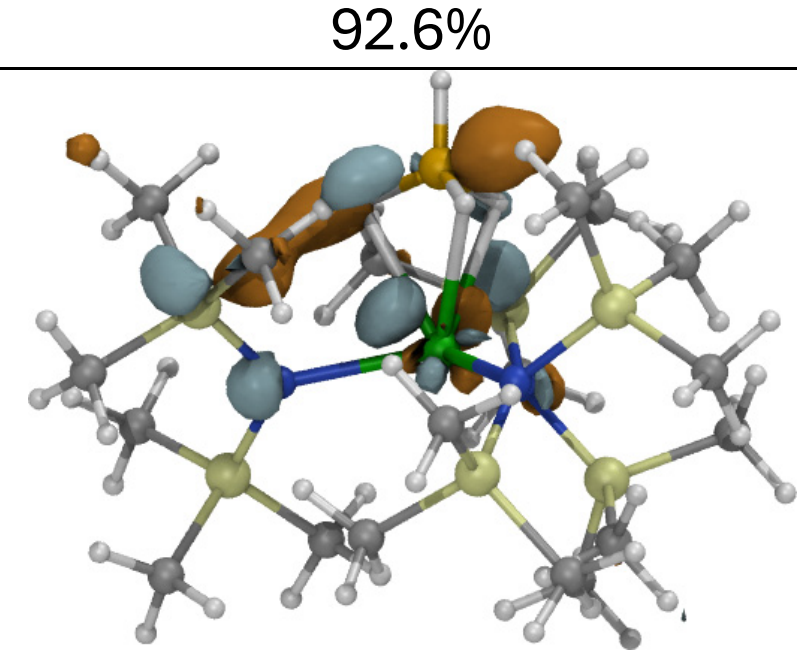<br>92.0%

37	192.444	0.001676	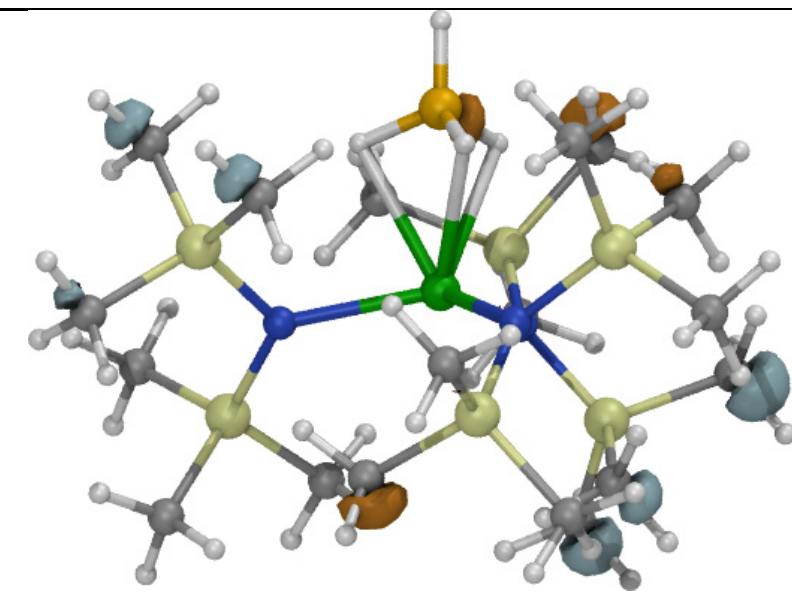 68.4%
			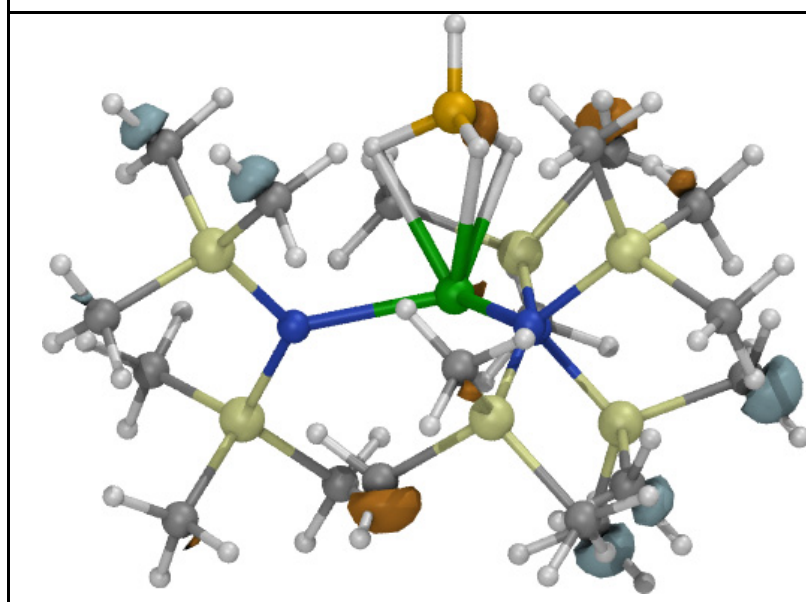 31.6%
38	192.463	0.001575	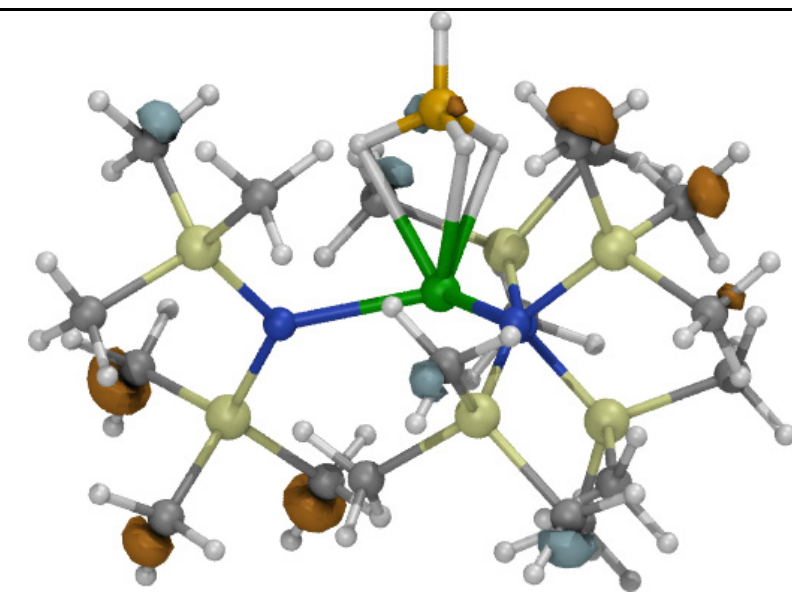 67.9%
			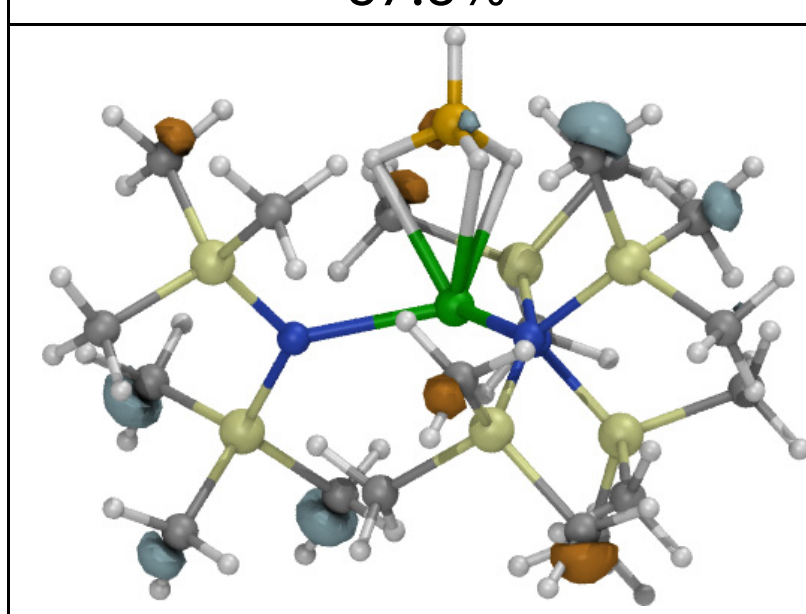 32.1%
39	192.614	0.000061	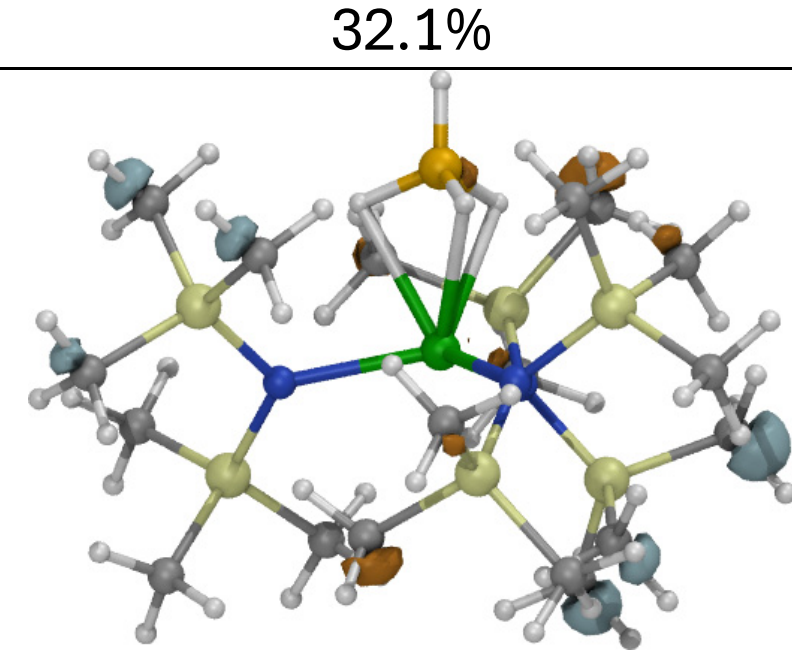 30.6%

			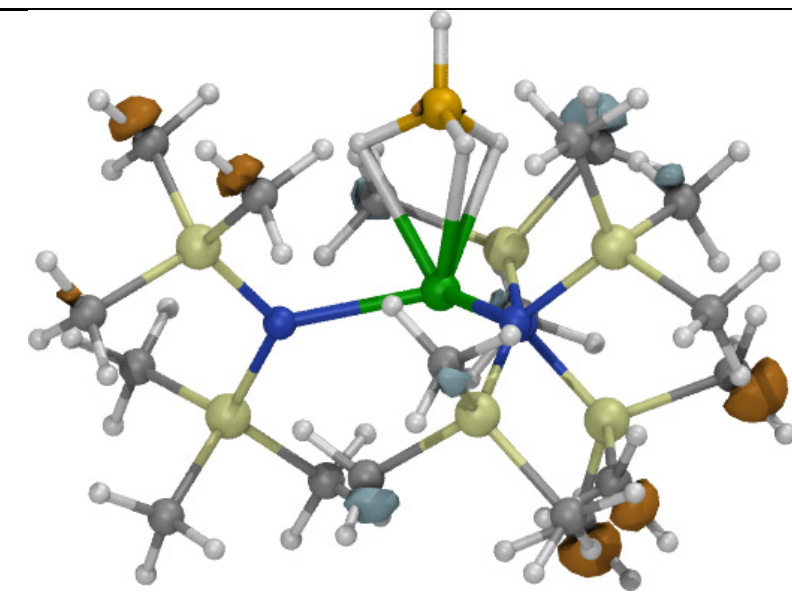 59.4%
40	192.624	0.000058	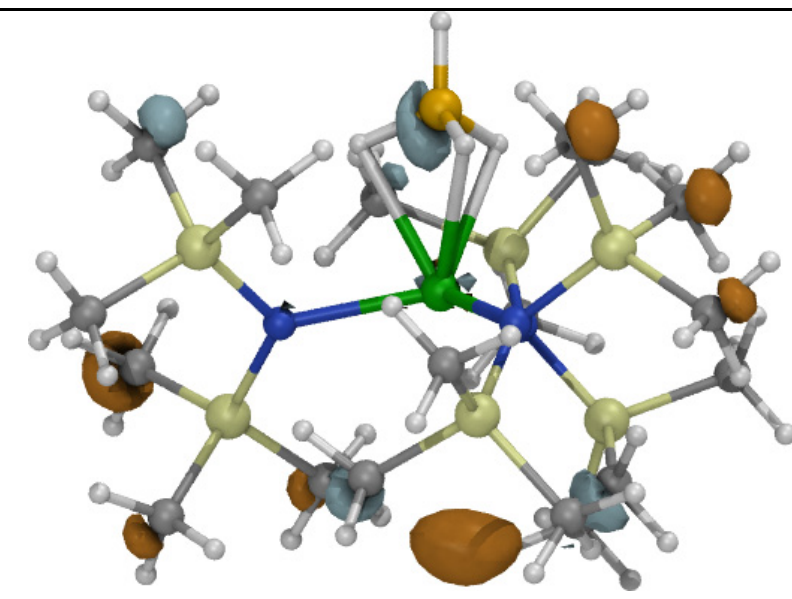 38.3%
			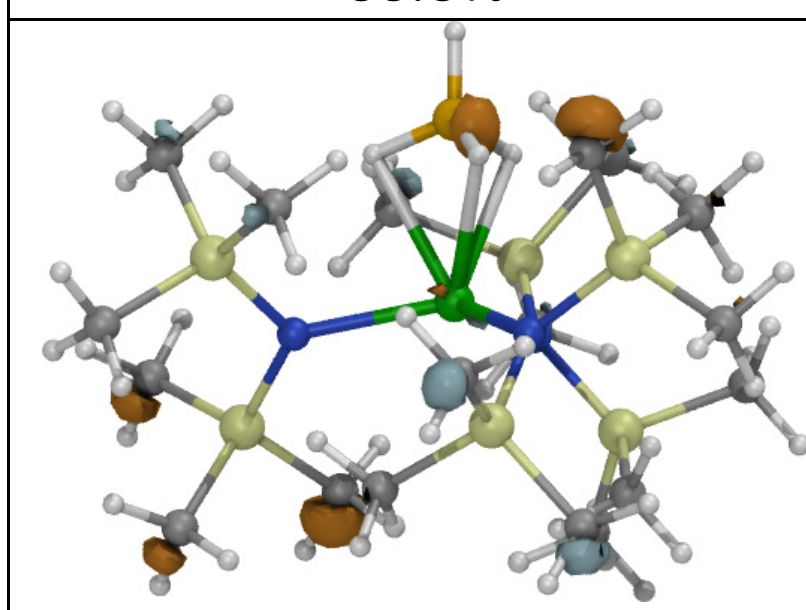 61.7%
41	192.634	0.001368	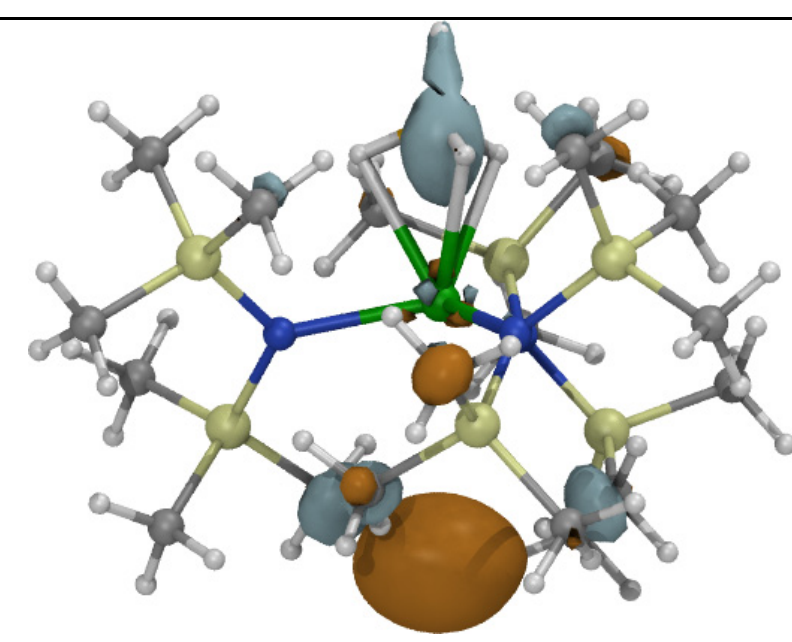 65.5%
			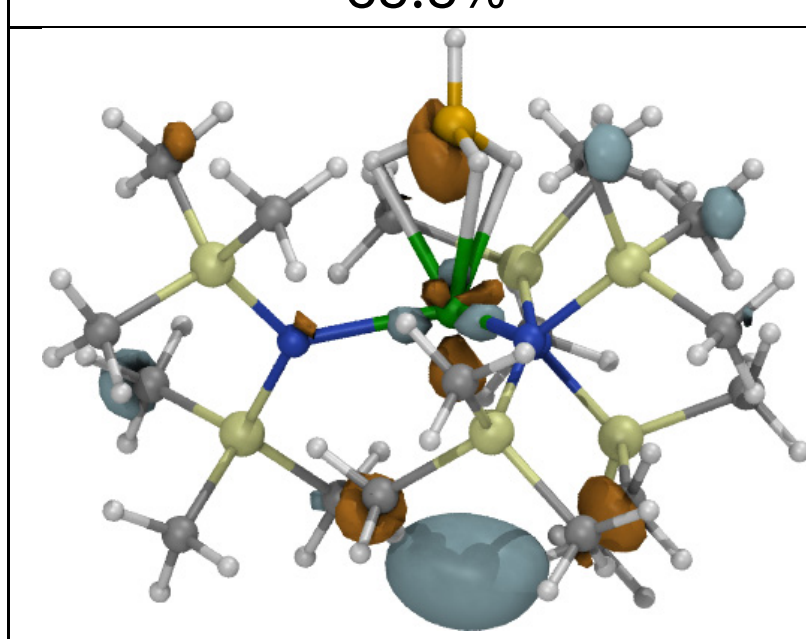 34.5%

42	192.647	0.001298	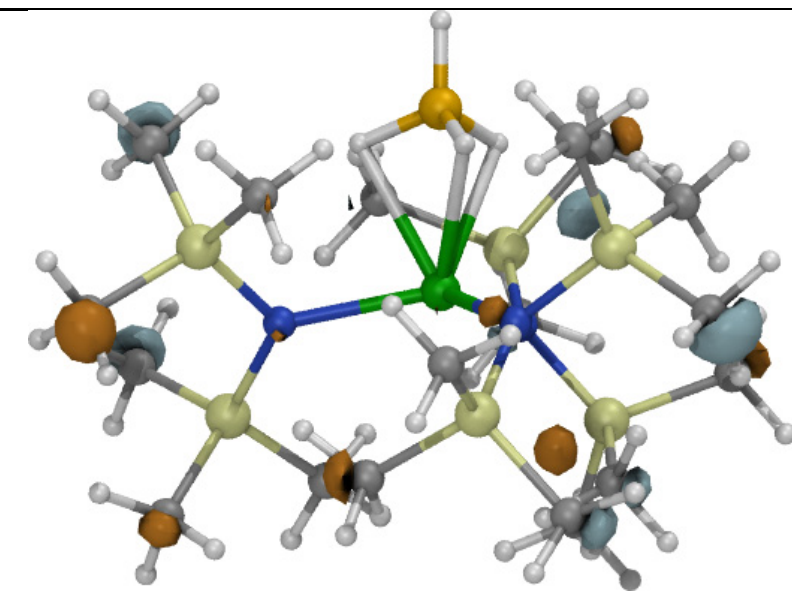<br>92.2%
43	192.649	0.000378	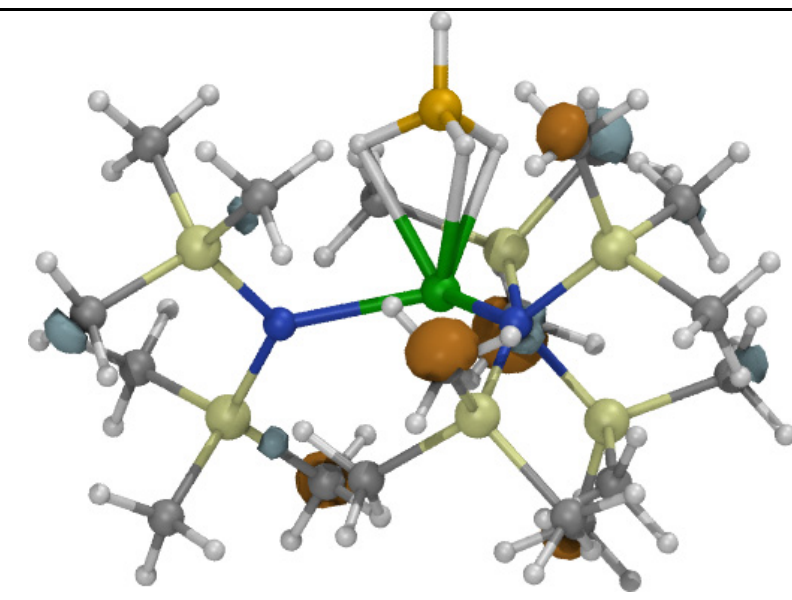<br>82.8%<br>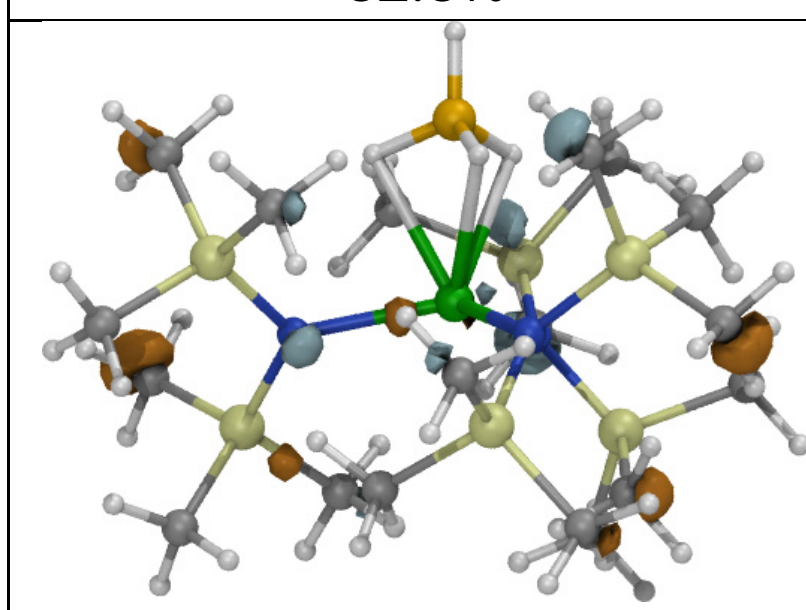<br>17.2%
44	192.747	0.000250	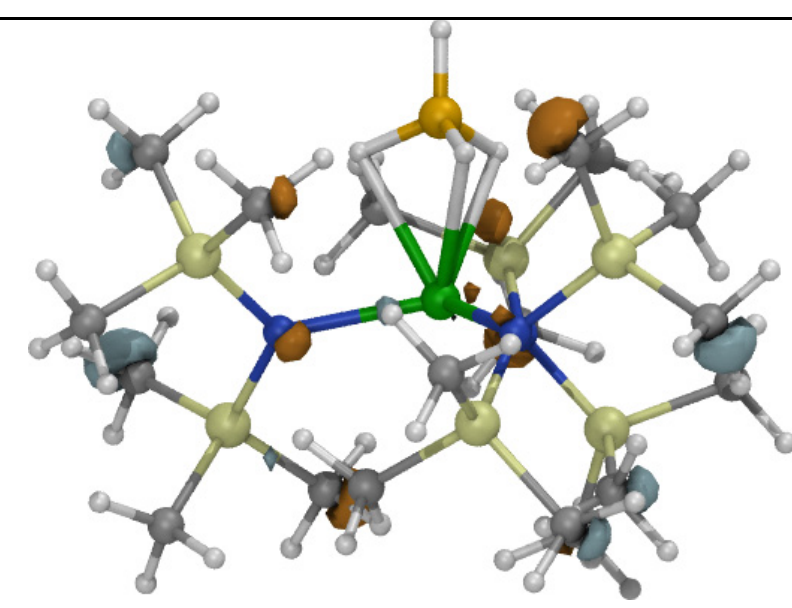<br>18.5%<br>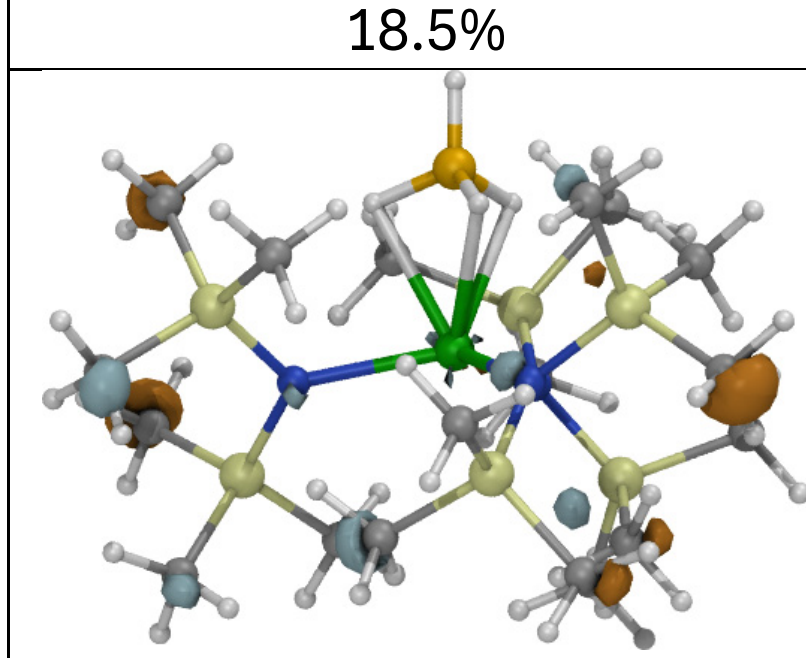<br>81.5%

<table>
<tr><td rowspan="2">45</td><td rowspan="2">192.758</td><td rowspan="2">0.000138</td><td>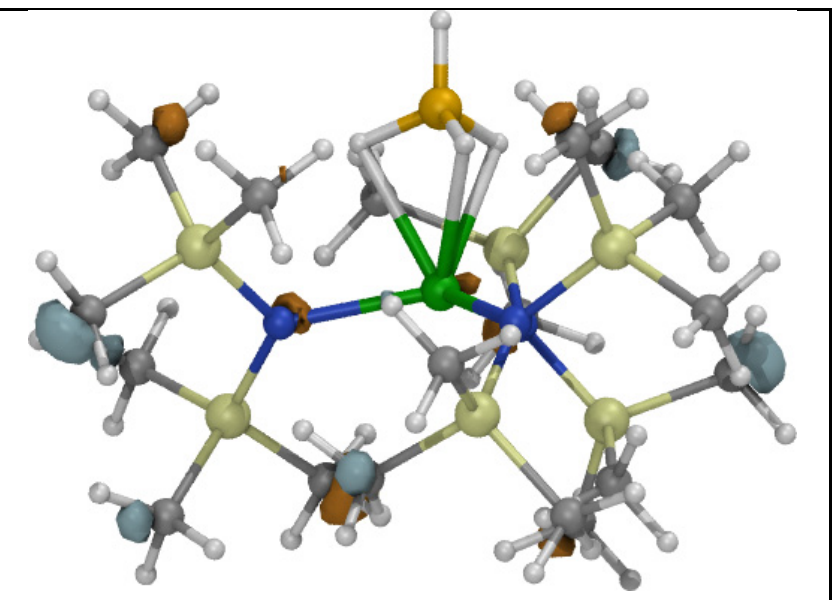
12.4%</td></tr>
<tr><td>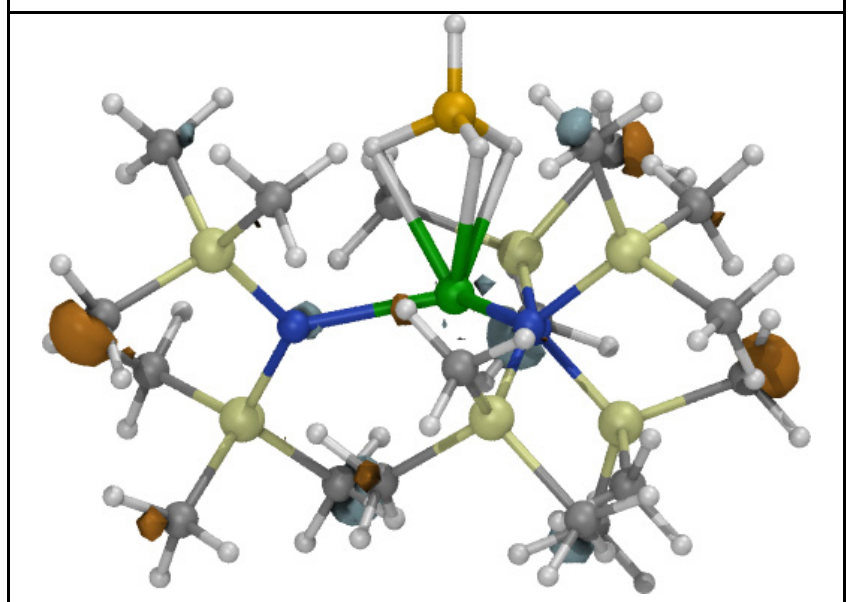
87.6%</td></tr>
<tr><td rowspan="2">46</td><td rowspan="2">192.759</td><td rowspan="2">0.000435</td><td>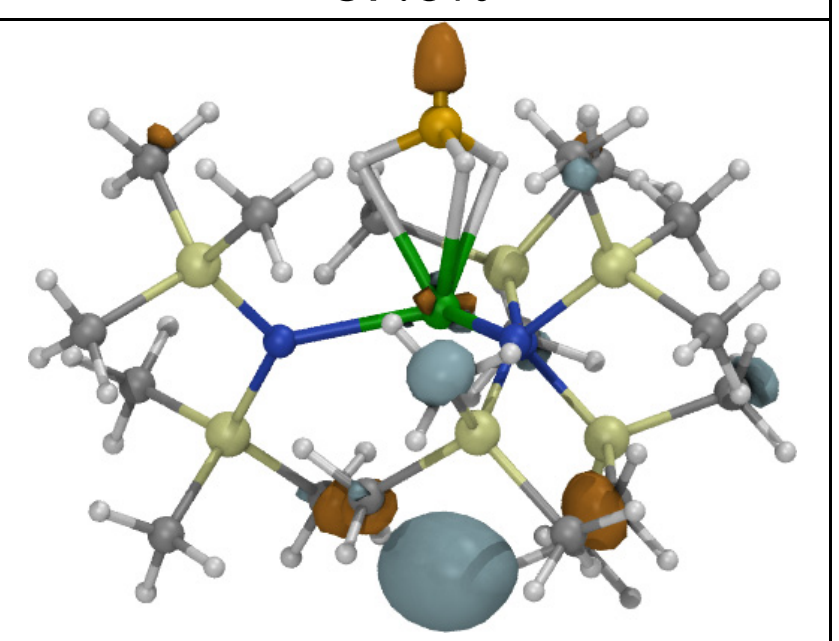
22.4%</td></tr>
<tr><td>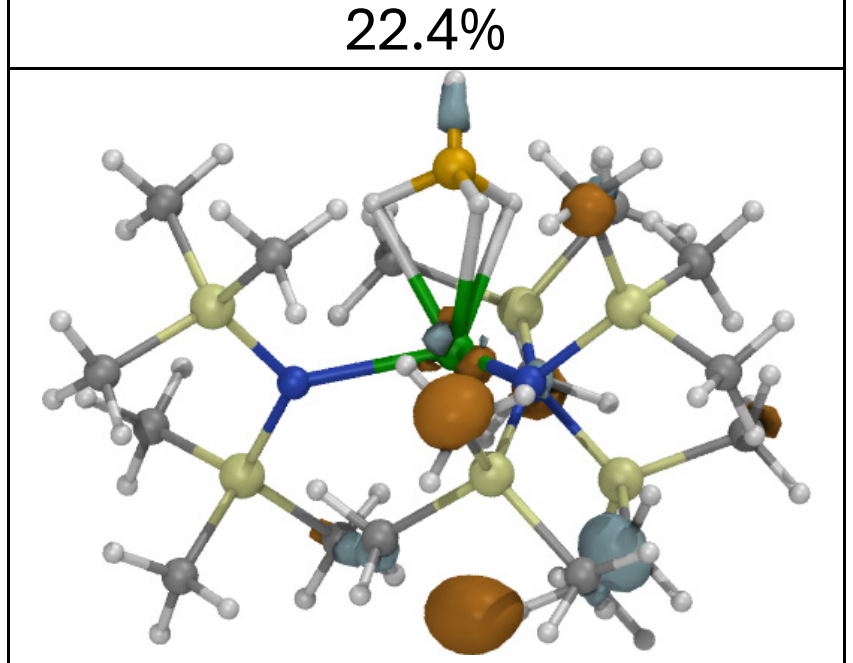
77.6%</td></tr>
<tr><td>47</td><td>192.768</td><td>0.000373</td><td>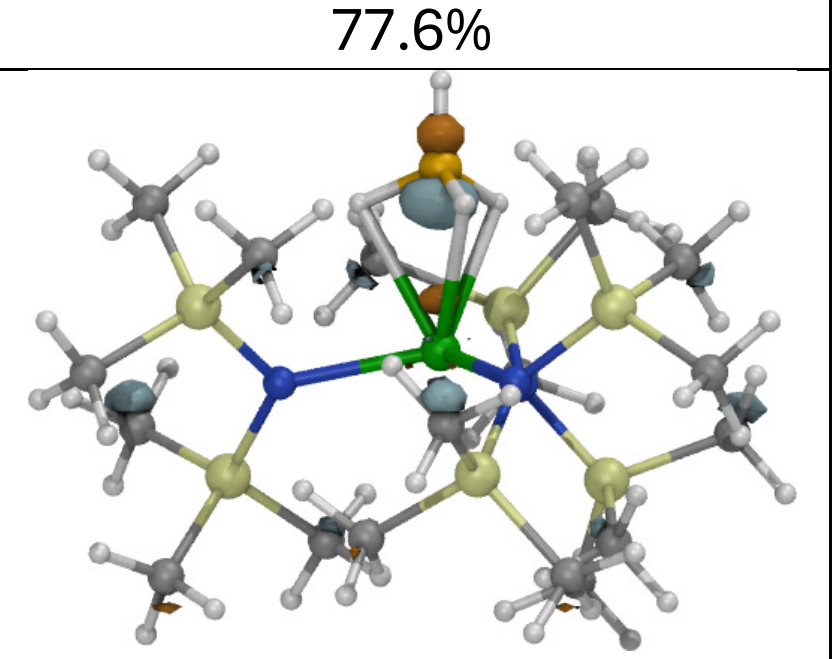
82.5%</td></tr>
</table>

			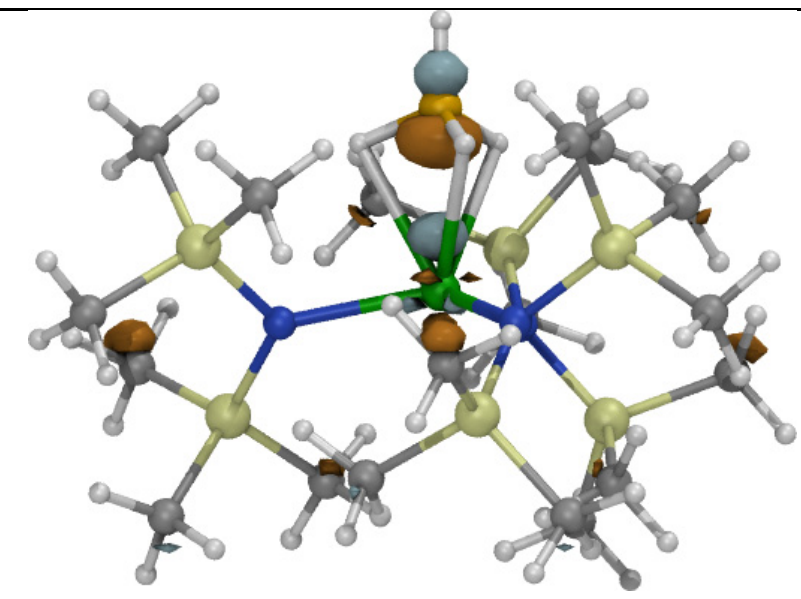 17.5%
48	192.771	0.000157	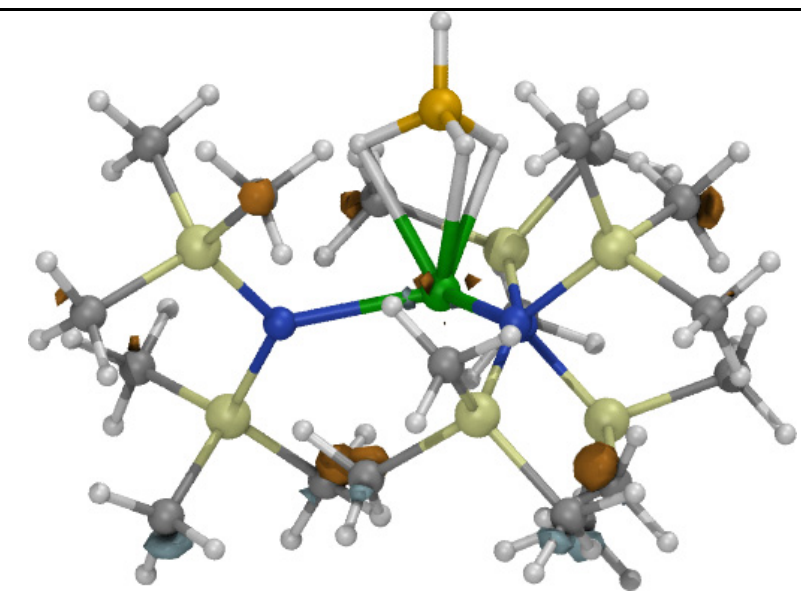 18.6%
			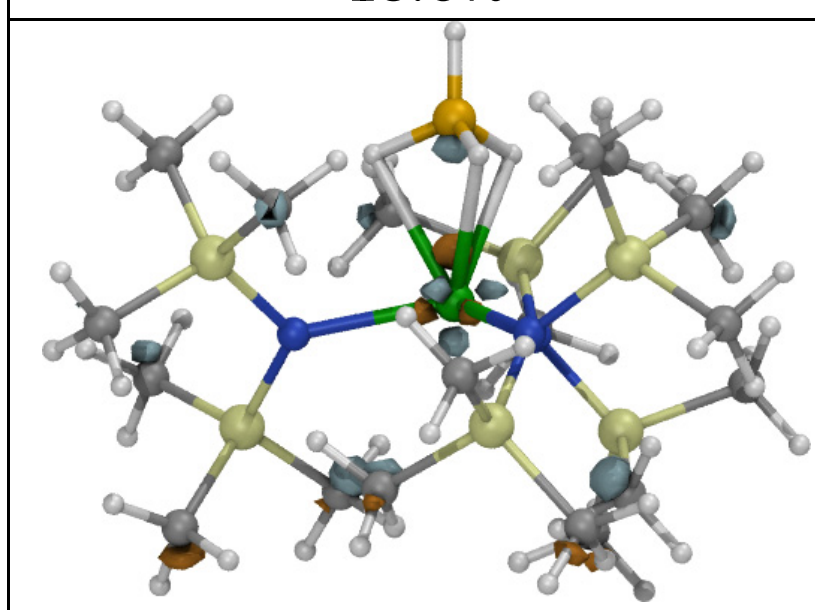 81.4%
49	192.851	0.000371	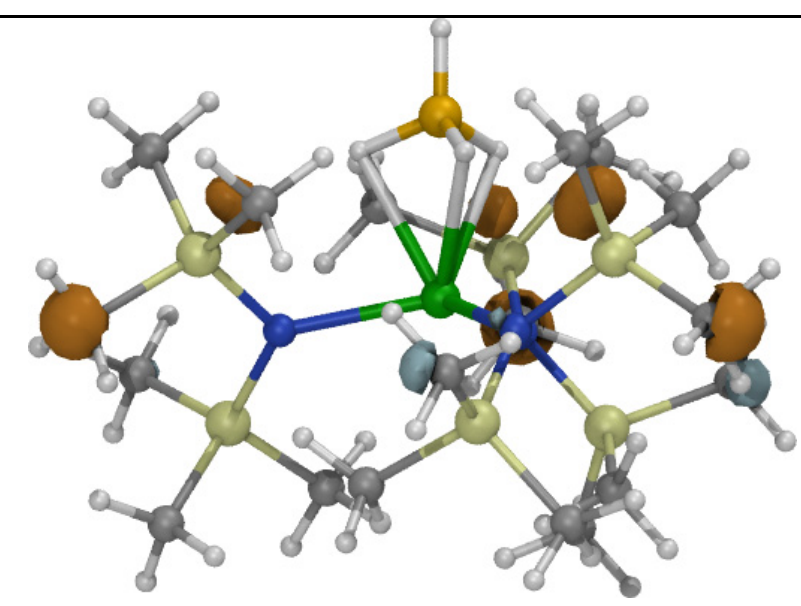 90.9%
50	192.899	0.001066	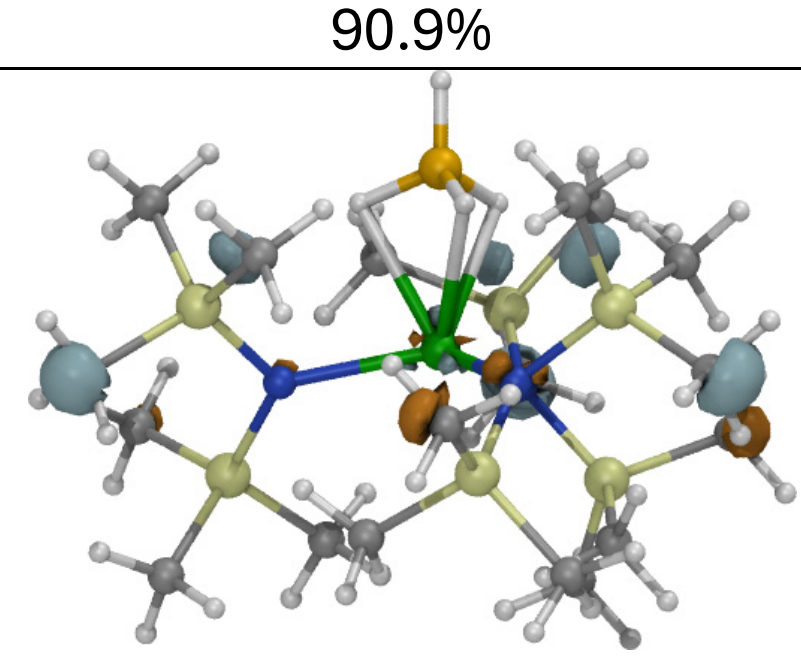 91.5%

51	192.955	0.000153	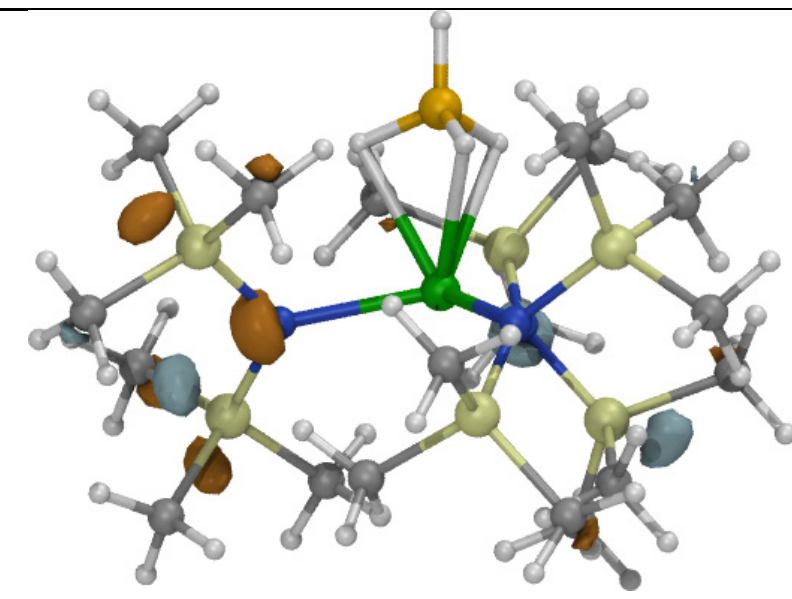 95.4%
52	192.978	0.000422	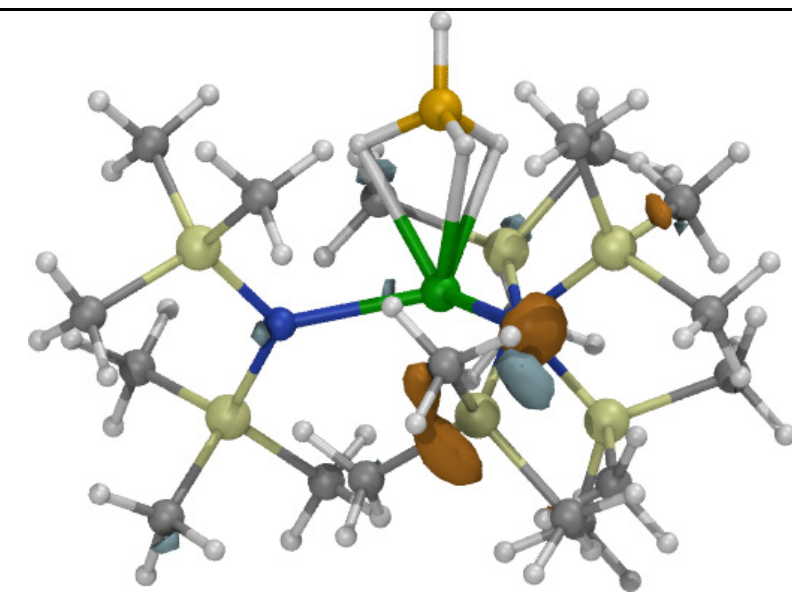 95.8%
53	193.010	0.000163	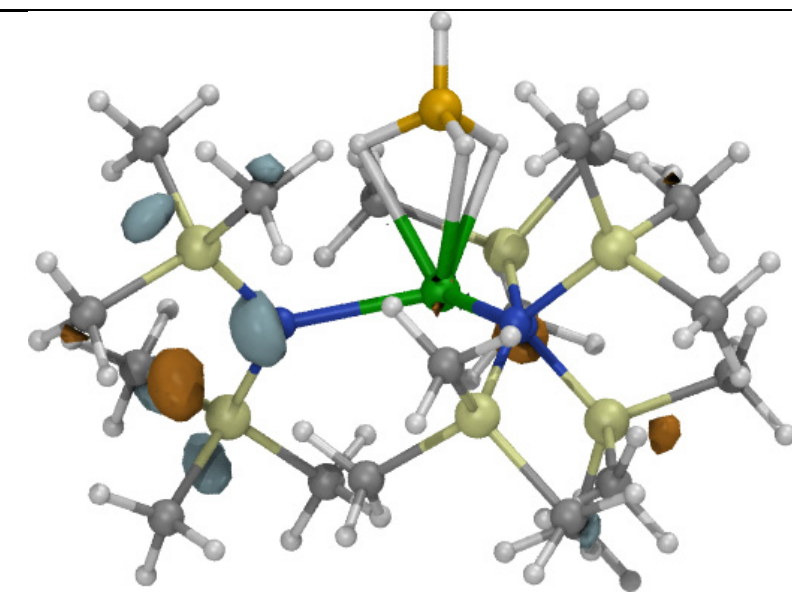 95.1%
54	193.011	0.000194	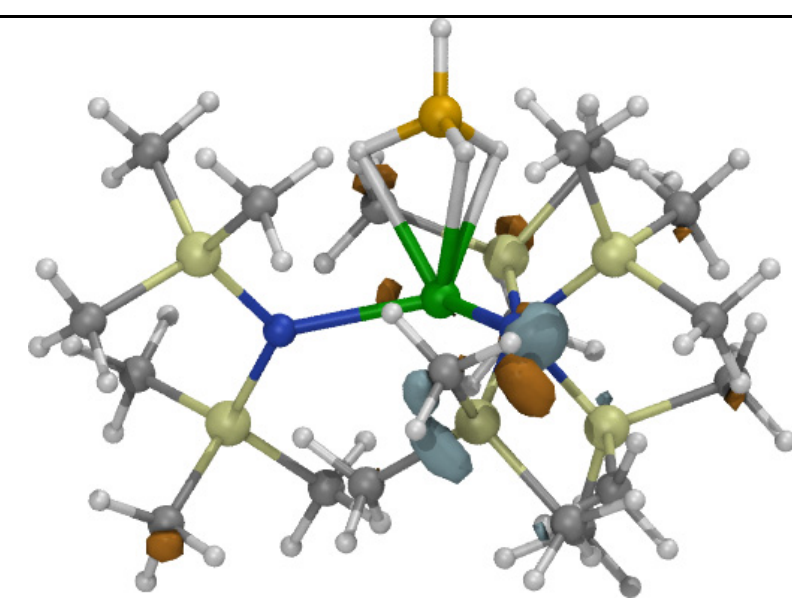 96.1%
57	193.028	0.000431	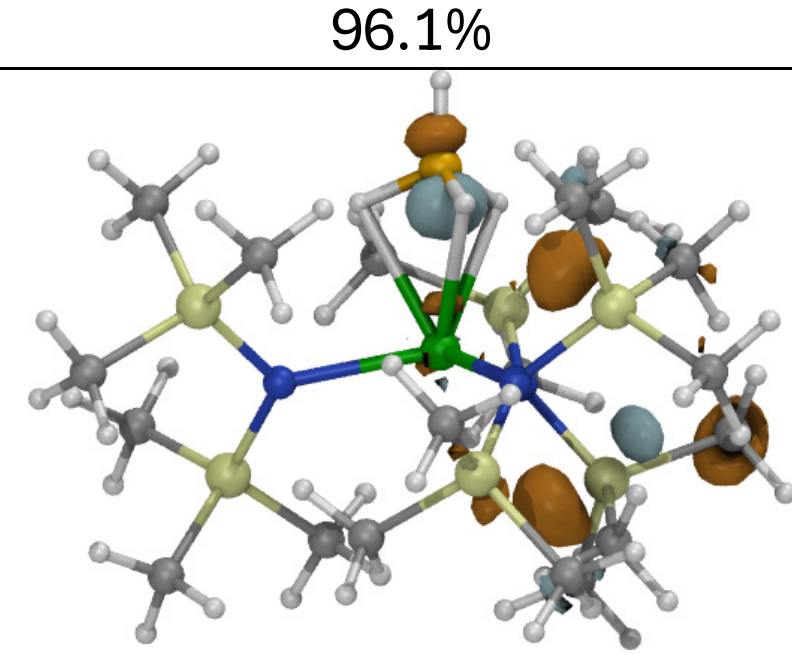 83.1%

			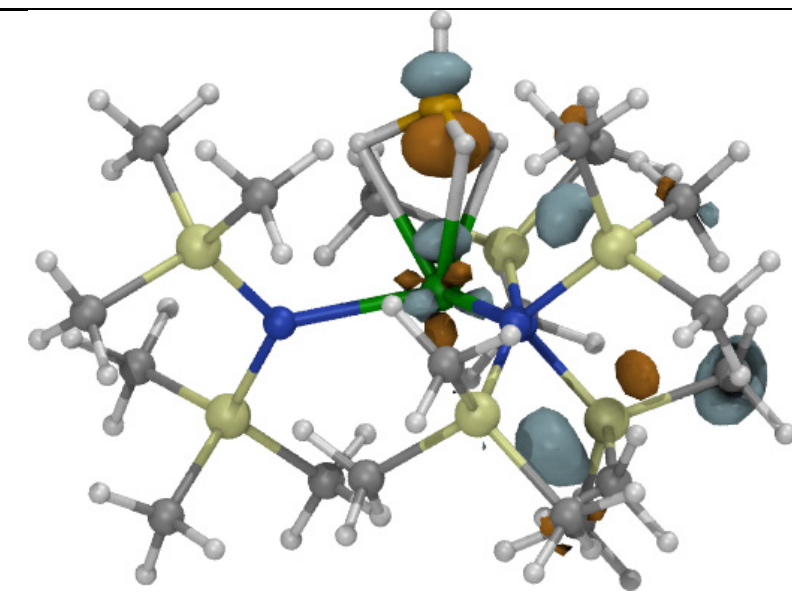 16.9%
58	193.032	0.000407	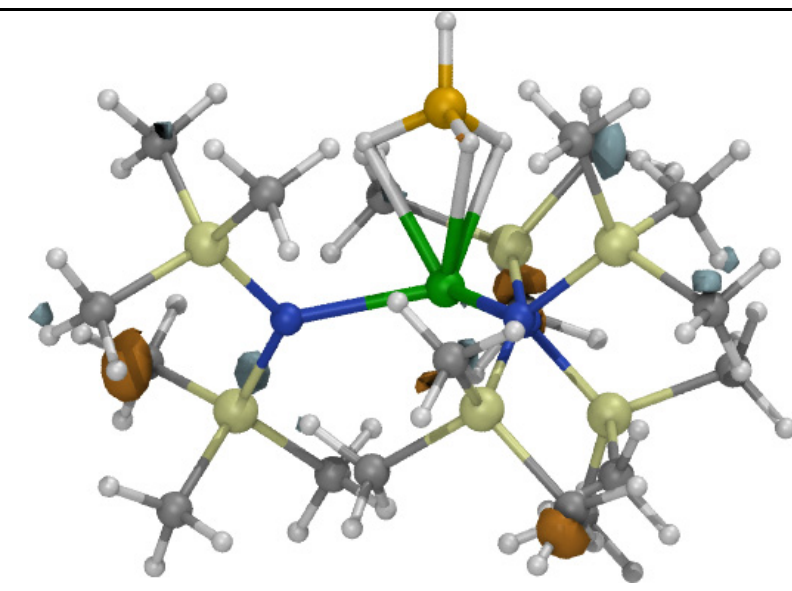 96.0%
59	193.268	0.000511	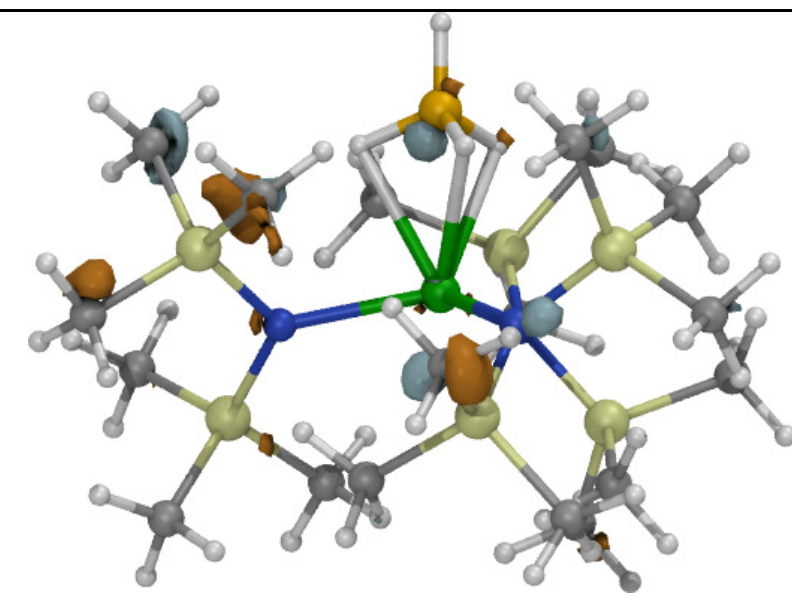 48.3%<br>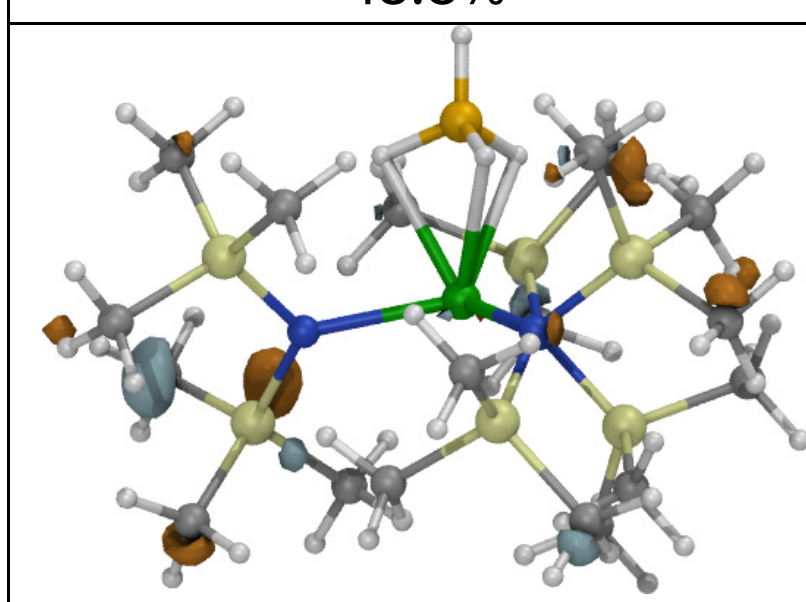 51.7%
60	193.276	0.000196	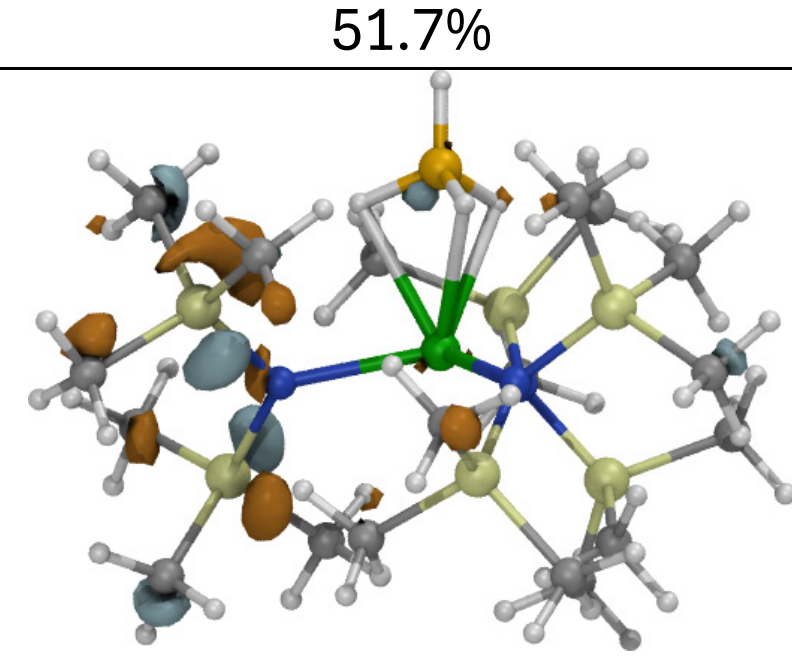 48.7%

			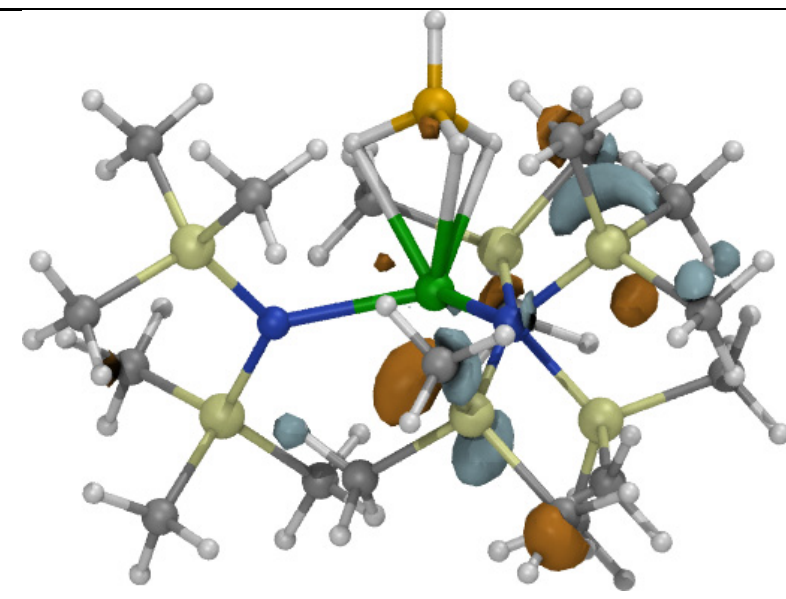 51.3%
61	193.297	0.000268	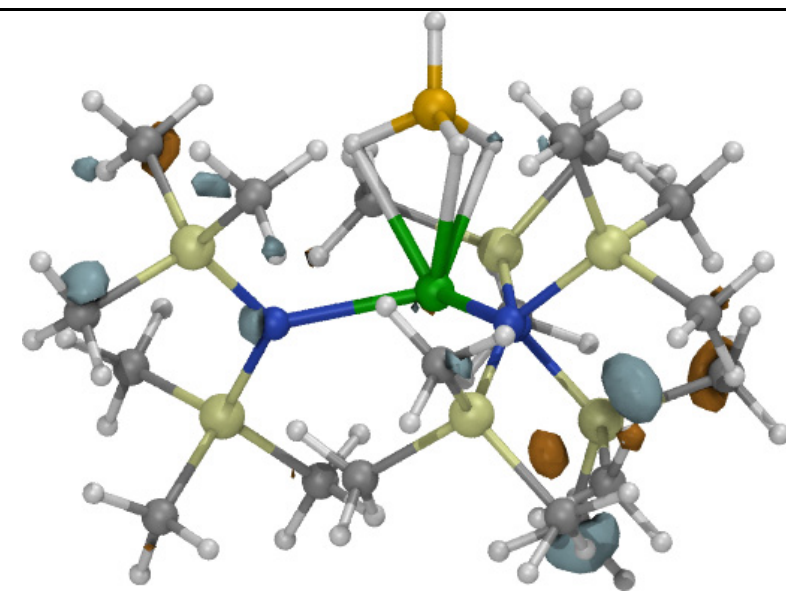 94.9%
62	193.297	0.000352	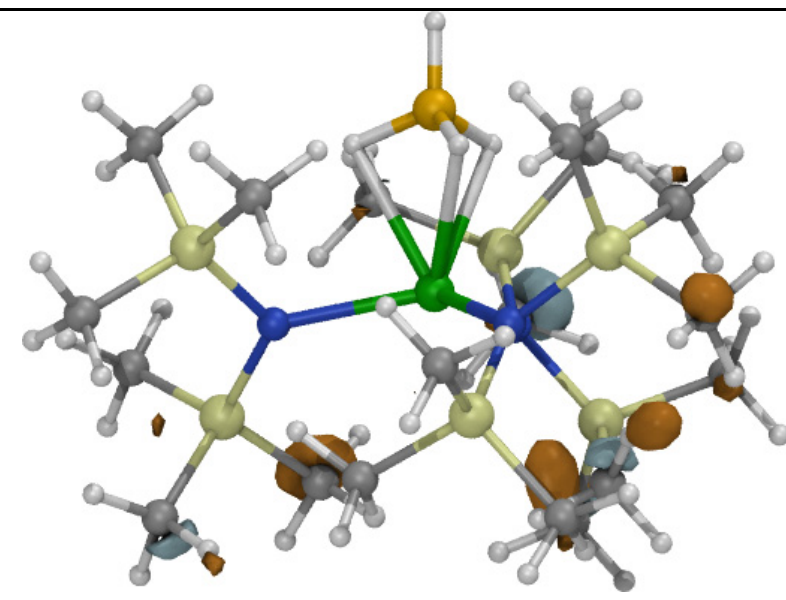 97.4%
63	193.304	0.000450	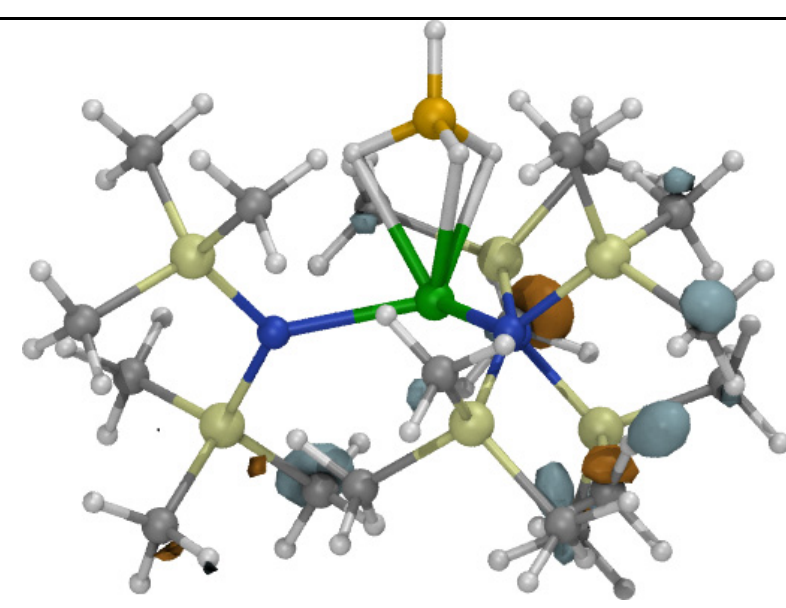 97.1%
64	193.364	0.000090	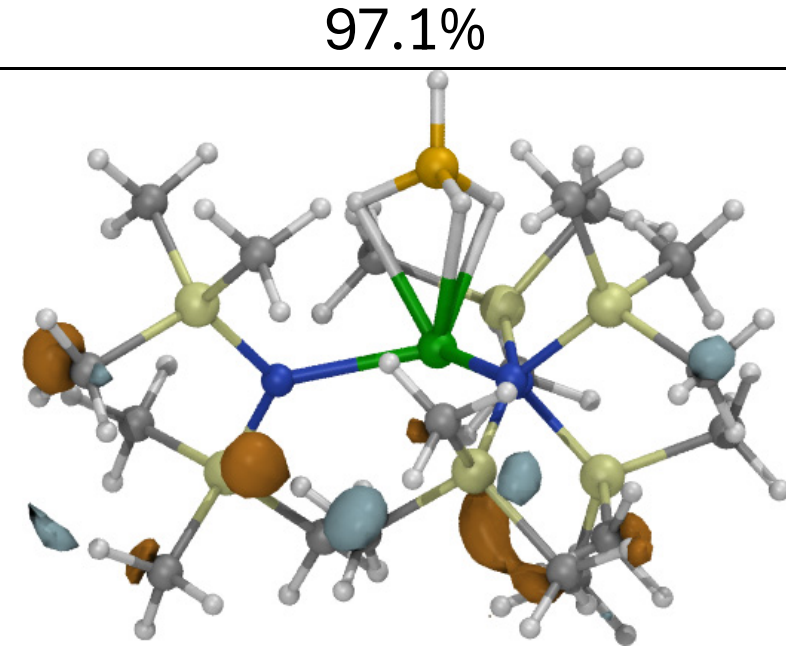 95.5%

65	193.373	0.000296	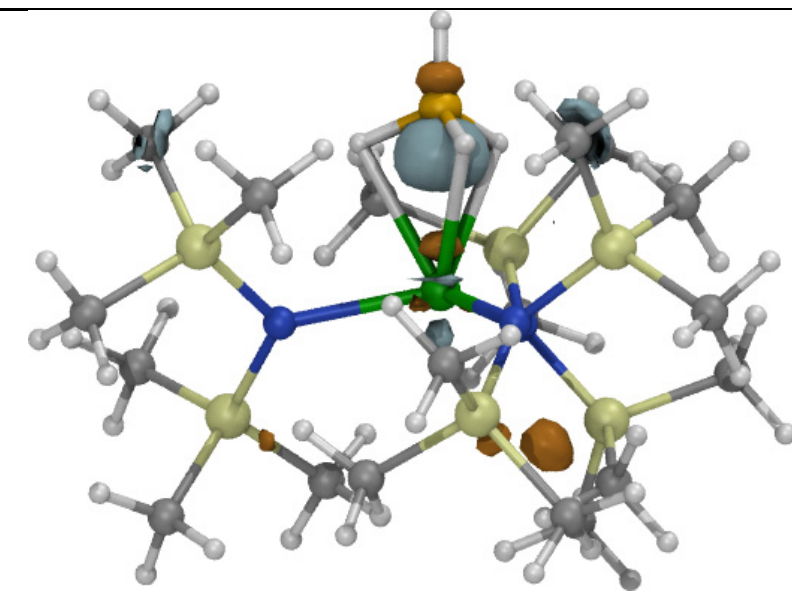<br>19.5%<br>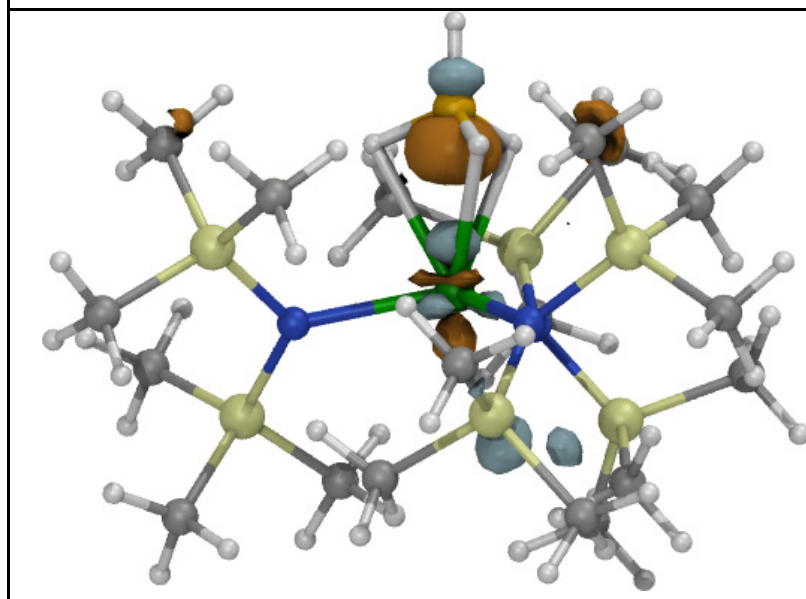<br>80.5%
66	193.390	0.000127	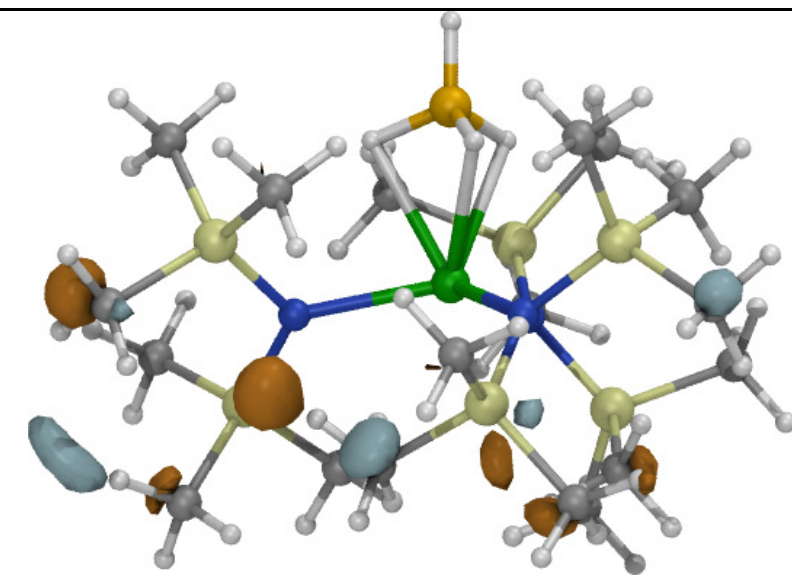<br>95.5%
72	193.398	0.005007	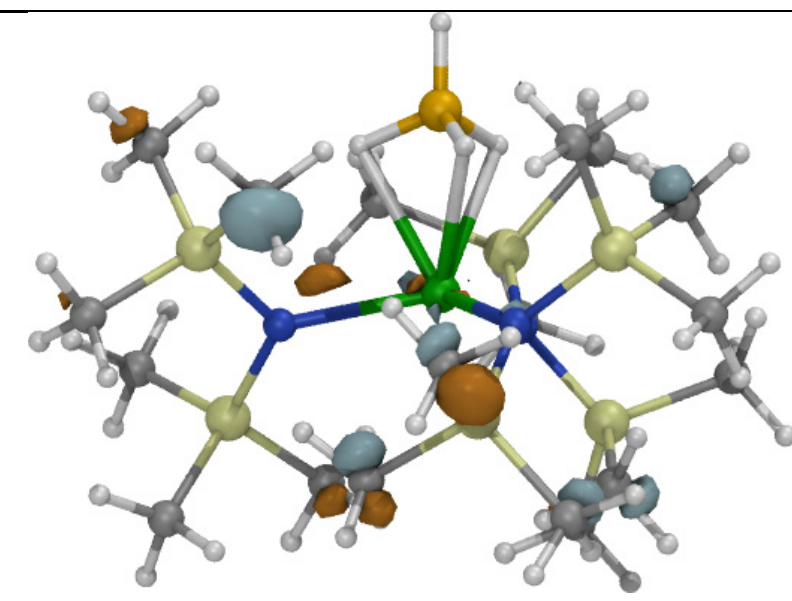<br>93.8%
74	193.403	0.000315	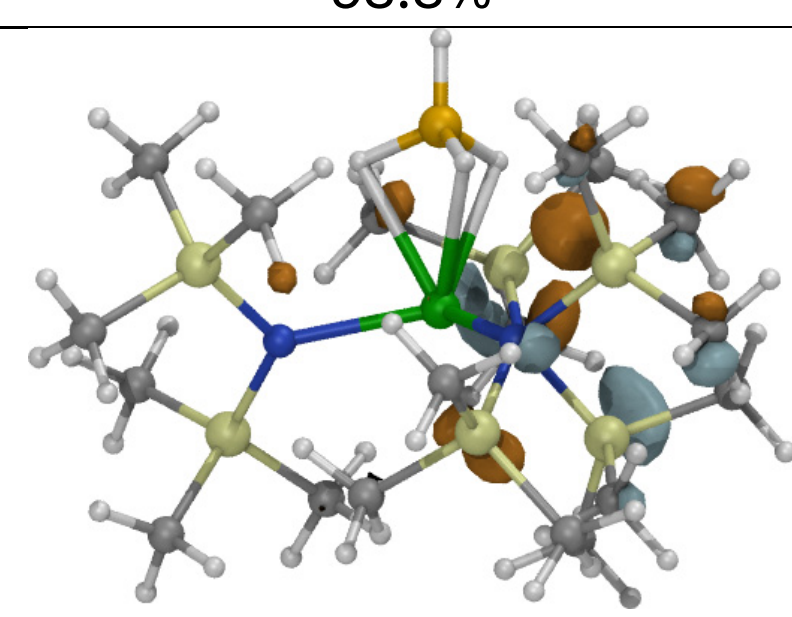<br>84.7%

			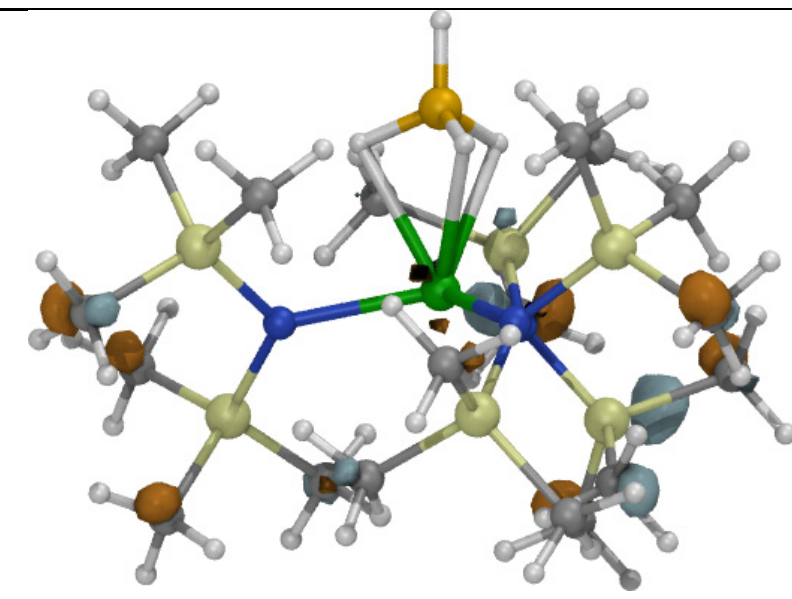 15.3%
76	193.545	0.000056	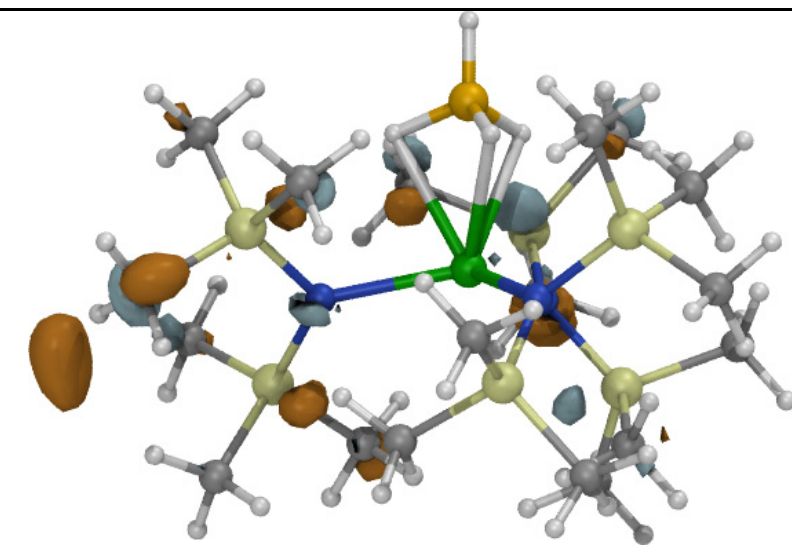 83.8%
			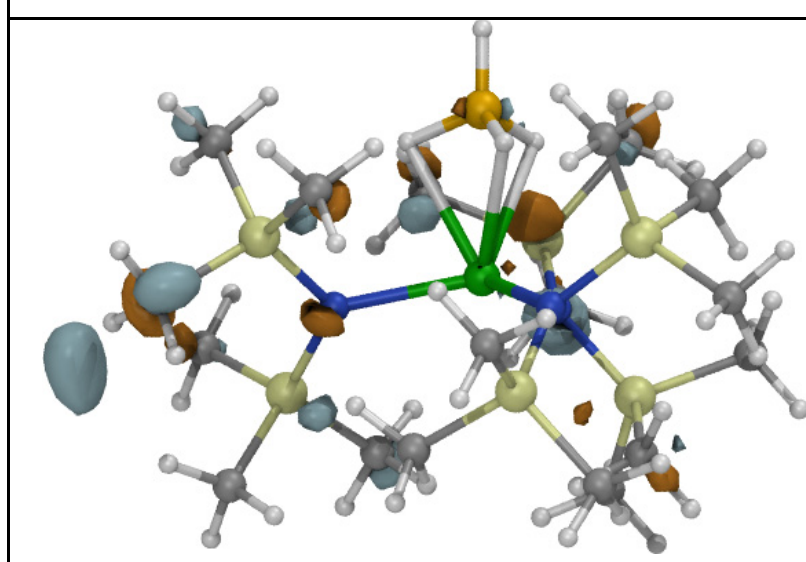 16.2%
77	193.707	0.000237	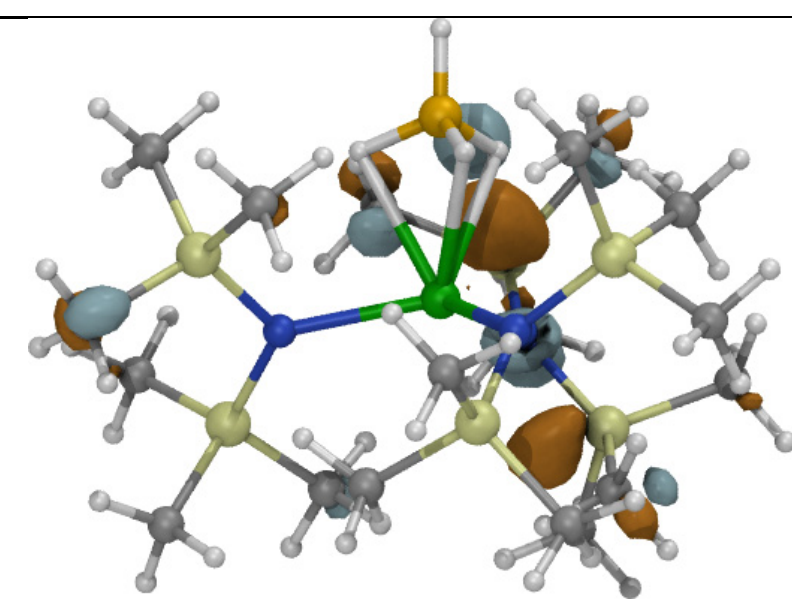 16.6%
			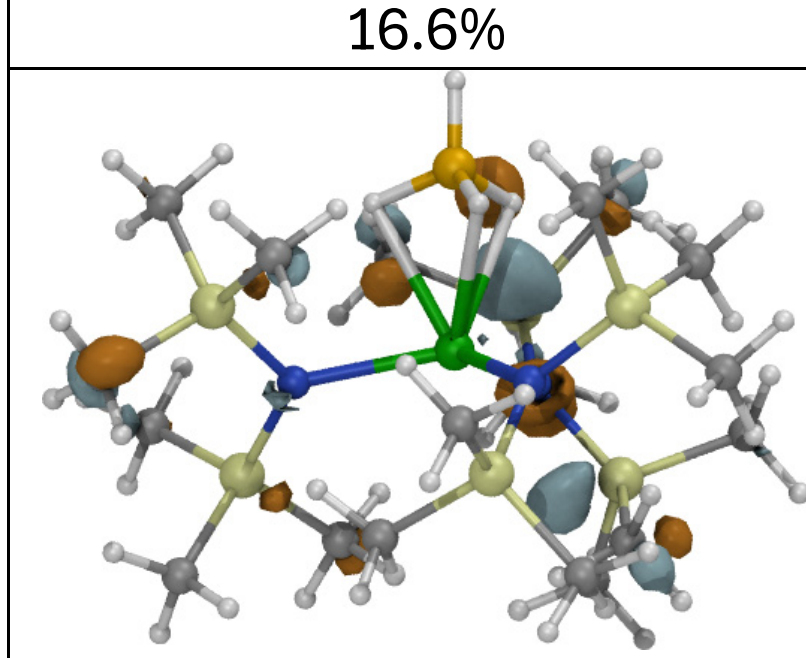 83.4%

78	193.726	0.000115	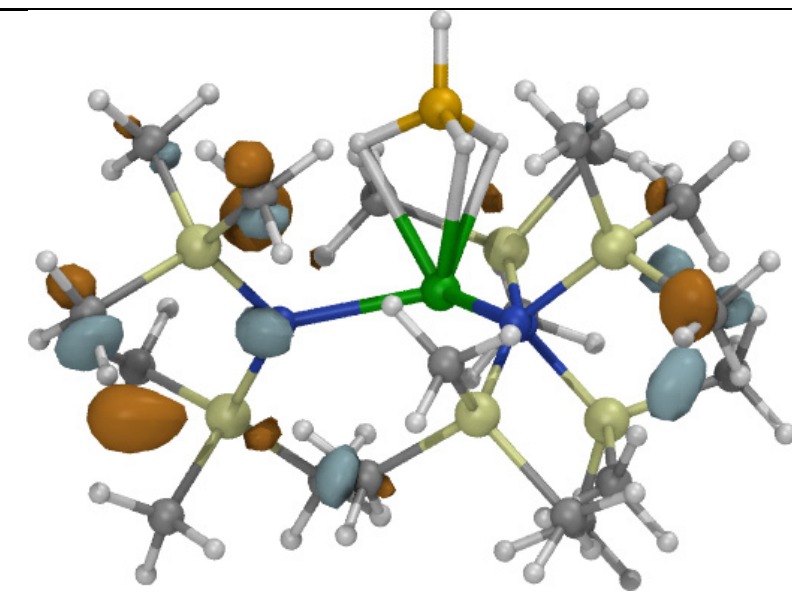<br>88.2%
			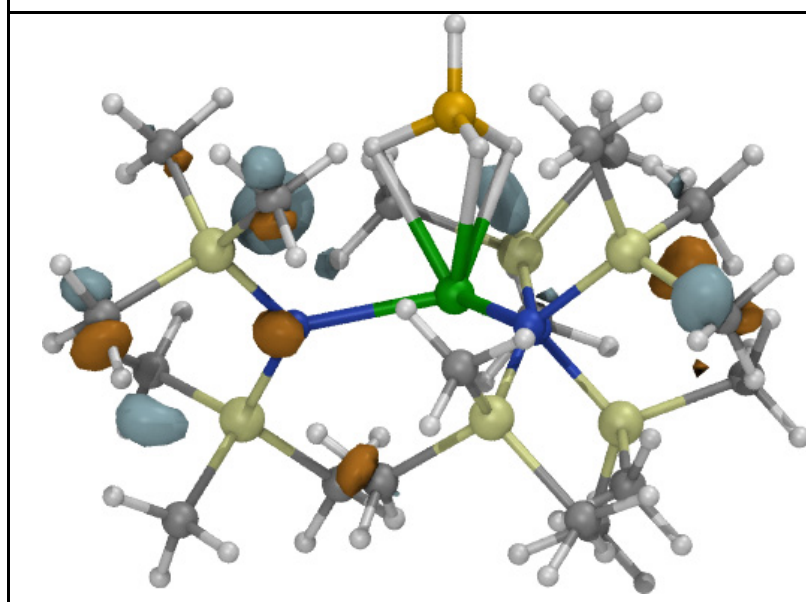<br>11.8%
79	193.742	0.000699	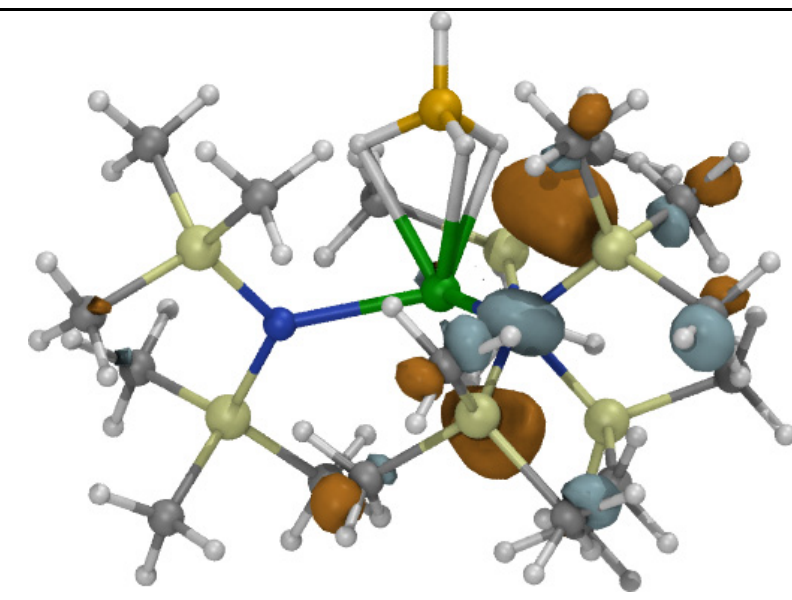<br>22.1%
			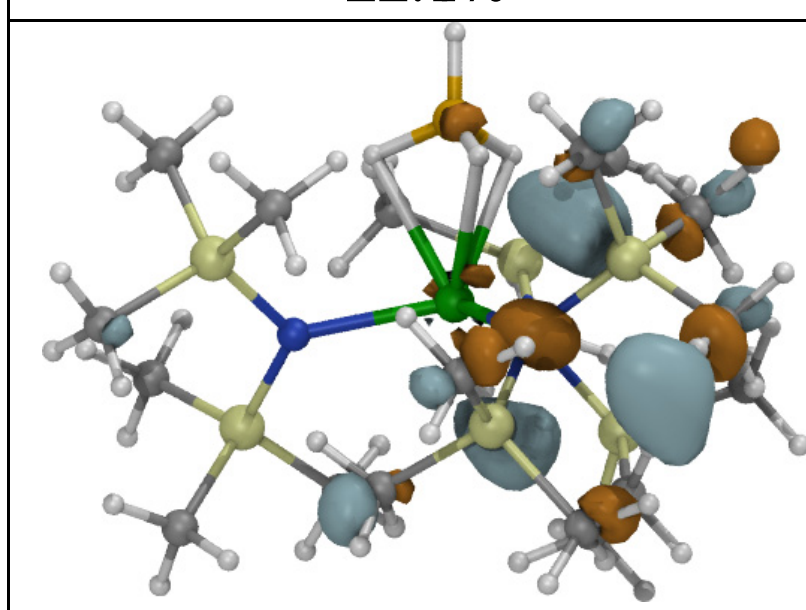<br>77.9%
80	193.752	0.000138	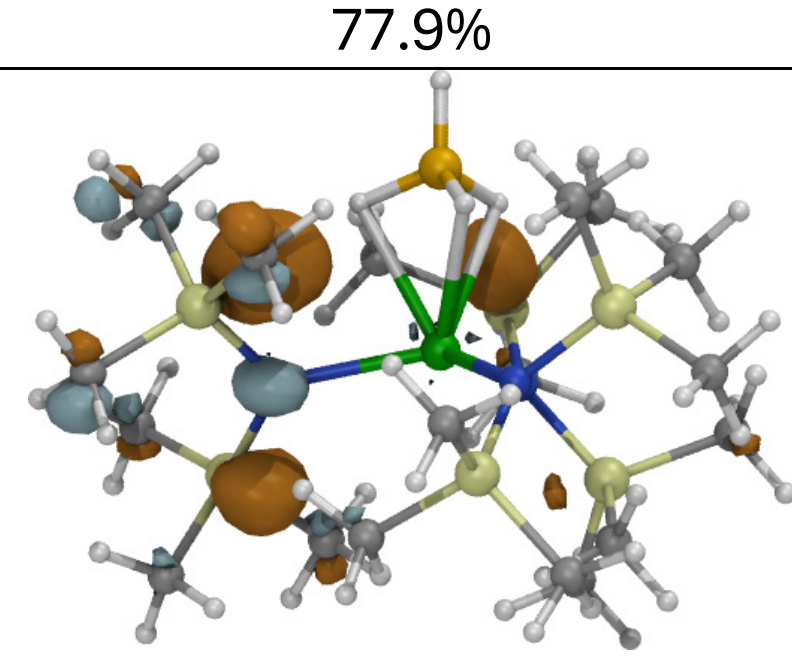<br>18.7%

			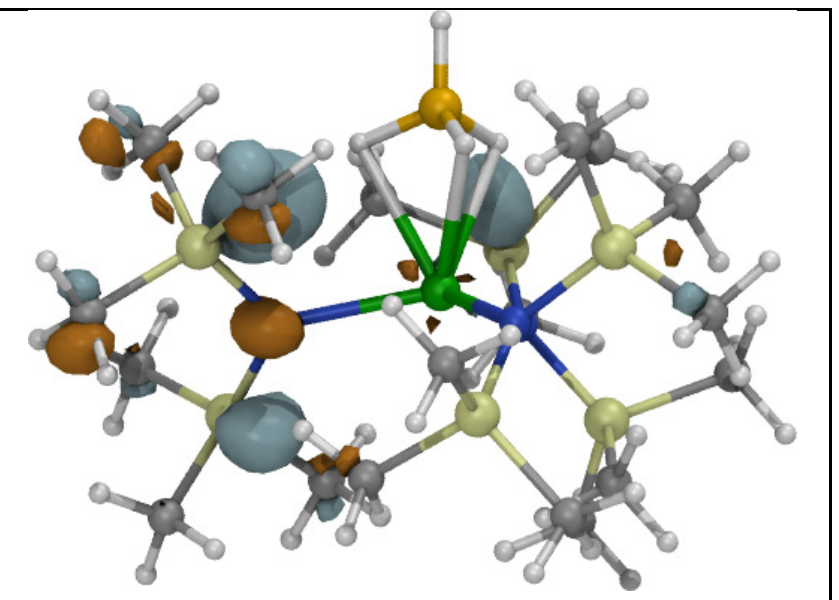<br>81.3%
83	193.761	0.000353	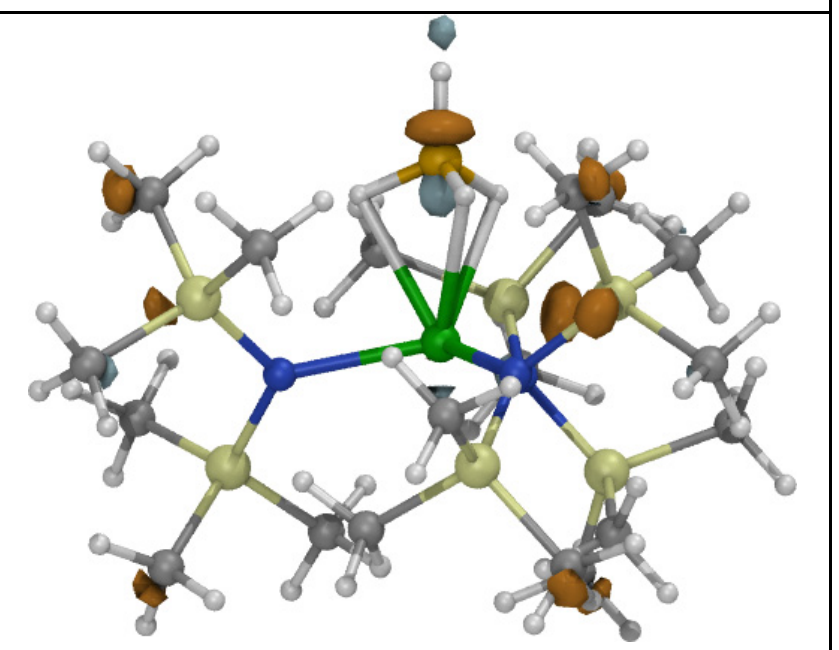<br>74.3%<br>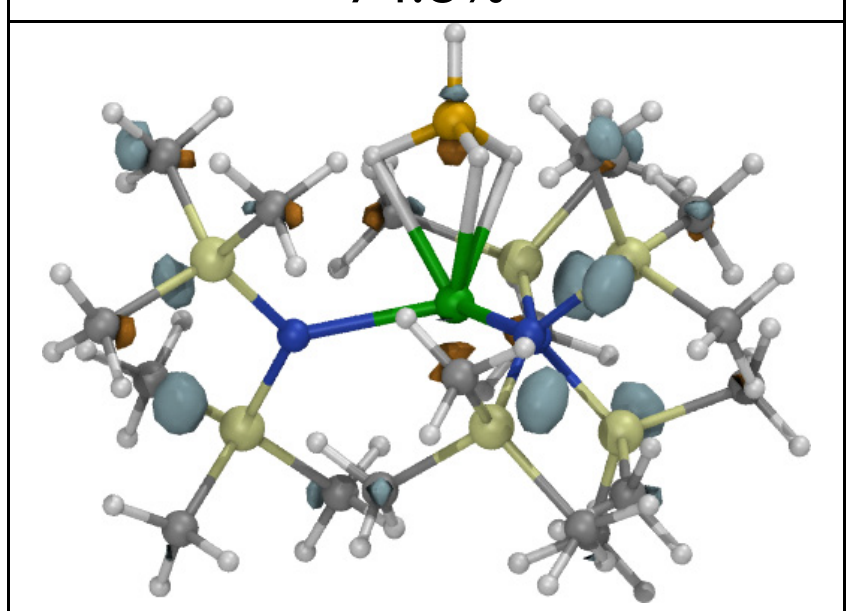<br>25.7%
84	193.770	0.000295	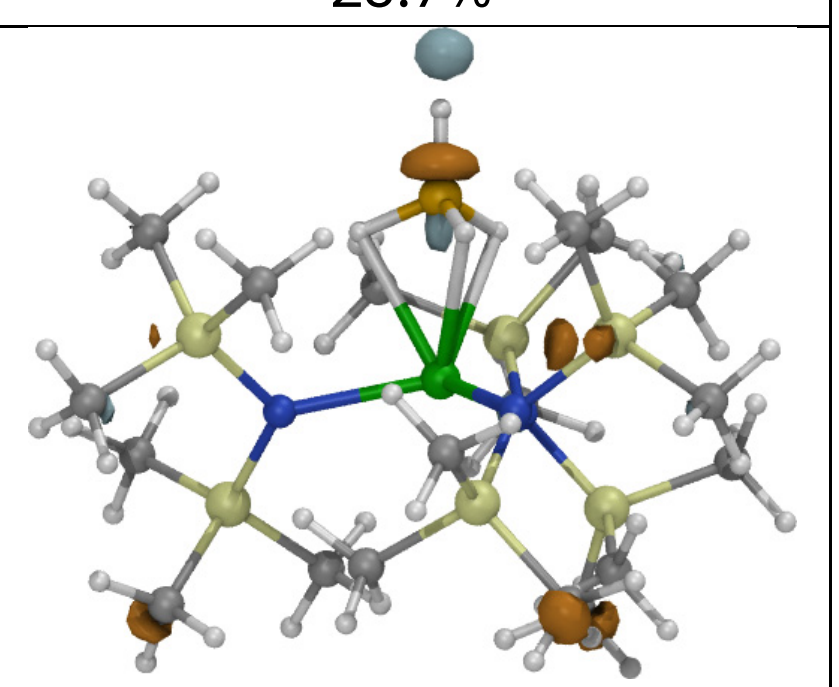<br>35.7%<br>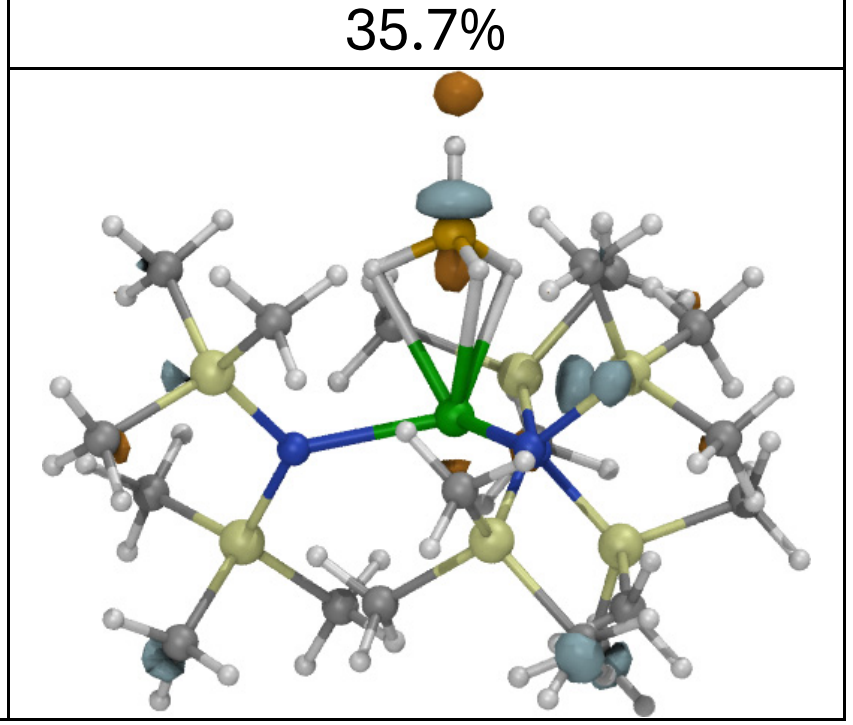

			64.3%
85	193.954	0.000078	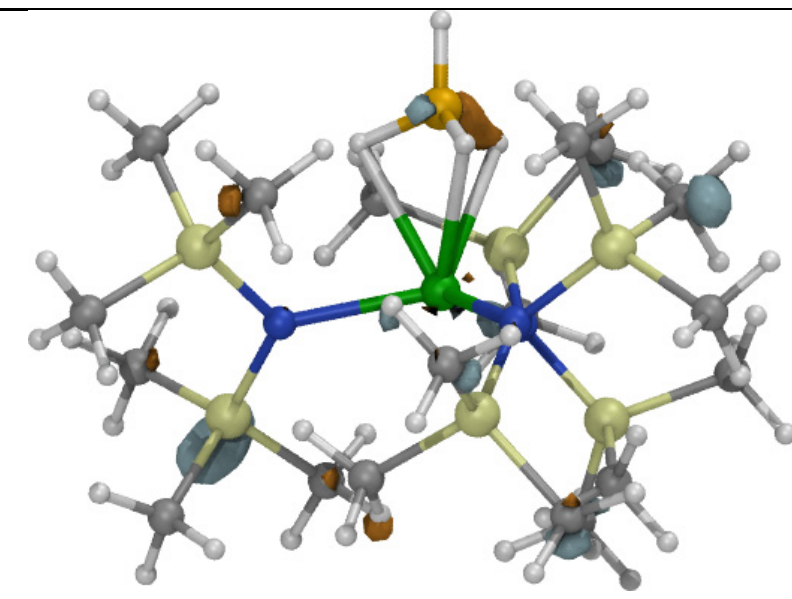<br>79.6%
			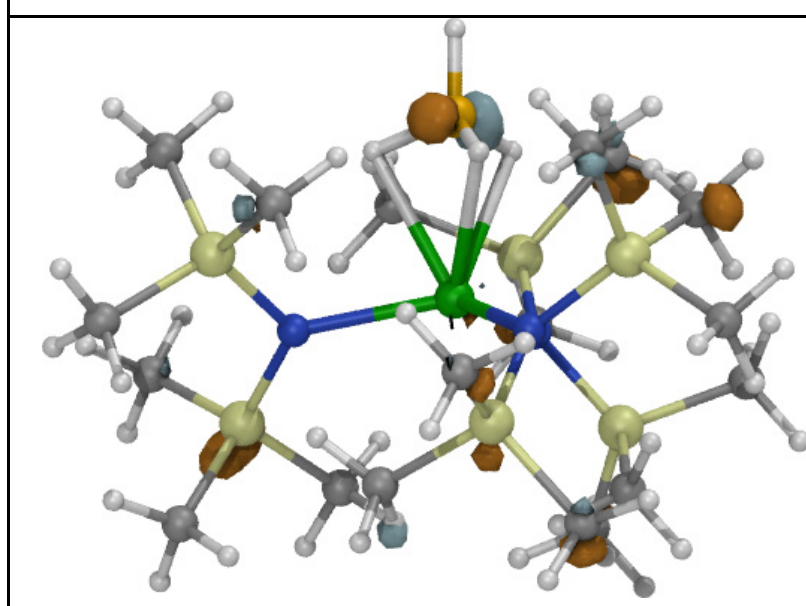<br>20.4%
86	194.002	0.002903	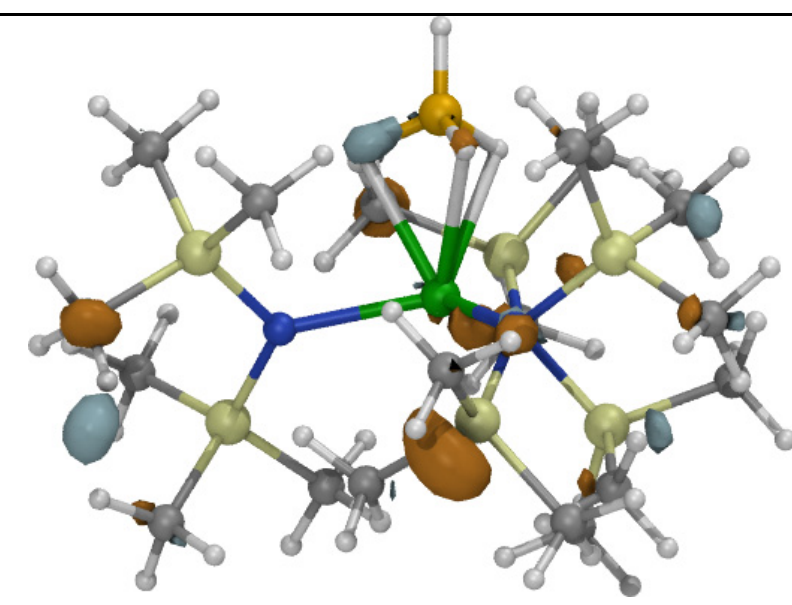<br>67.1%
			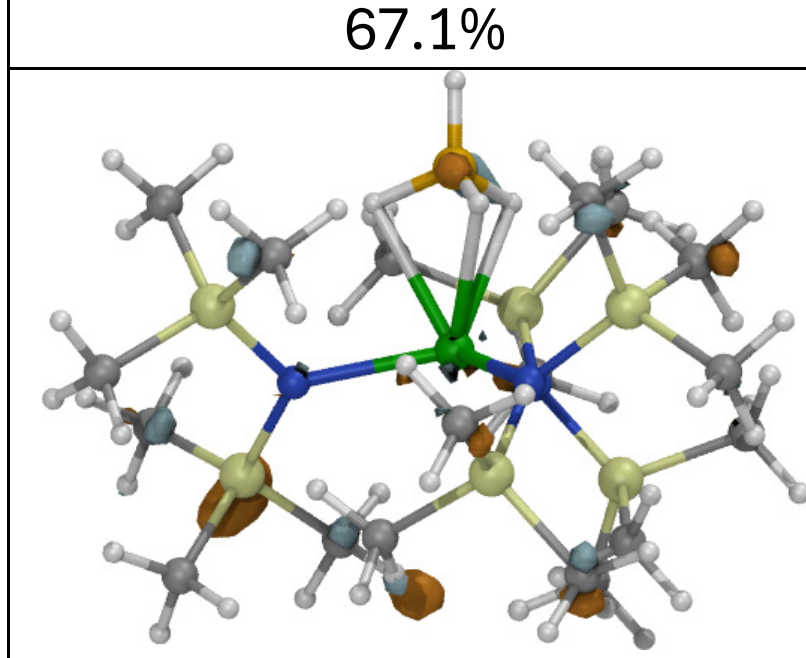<br>32.9%

87	194.110	0.000095	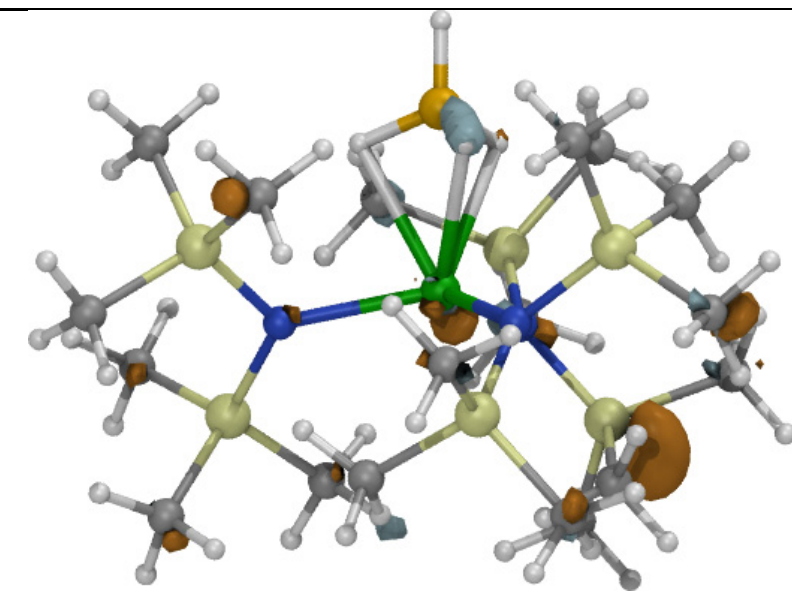 46.2%
			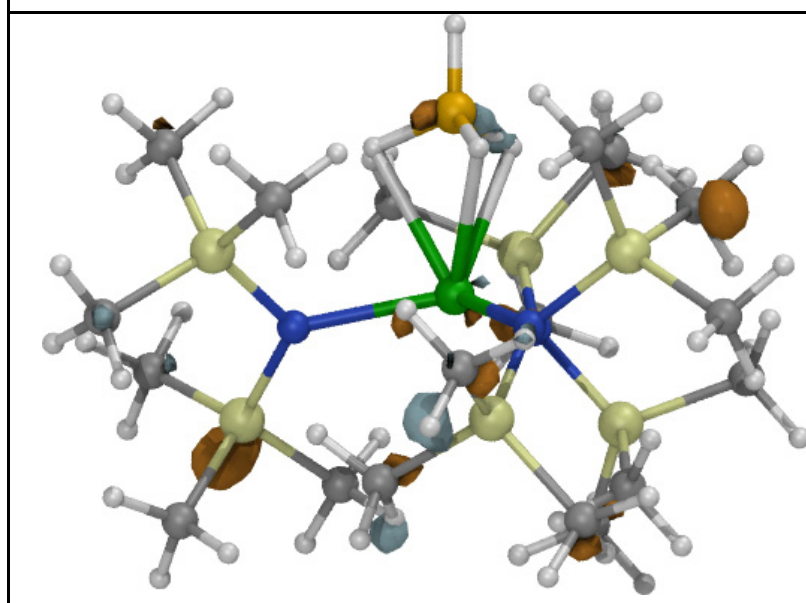 53.8%
88	194.134	0.000388	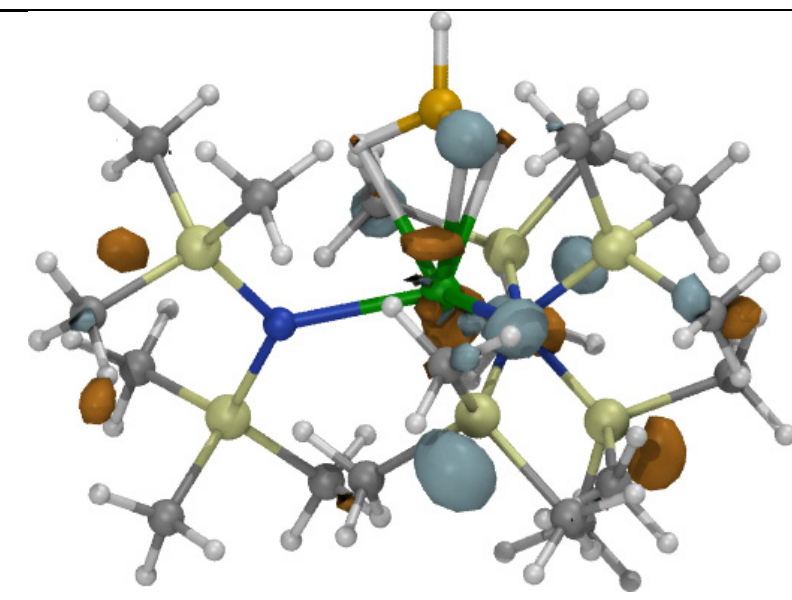 10.1%
			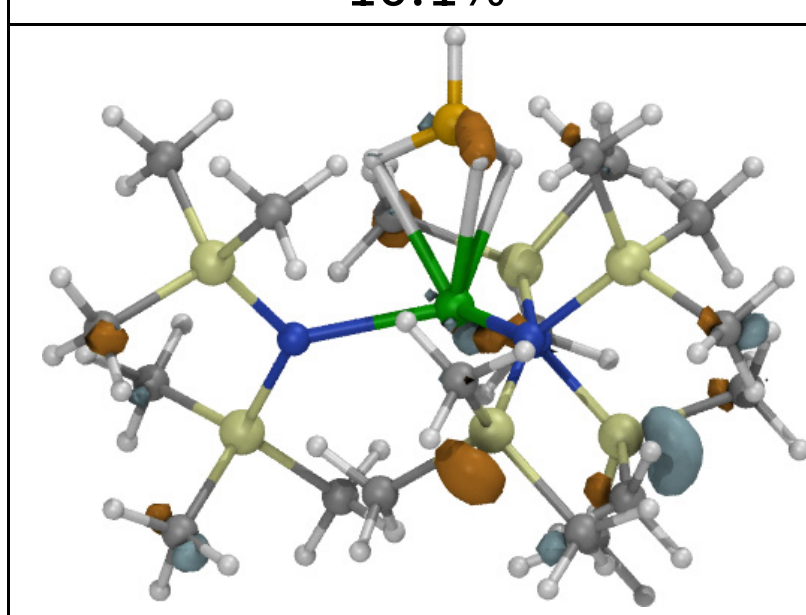 89.9%
89	194.137	0.000773	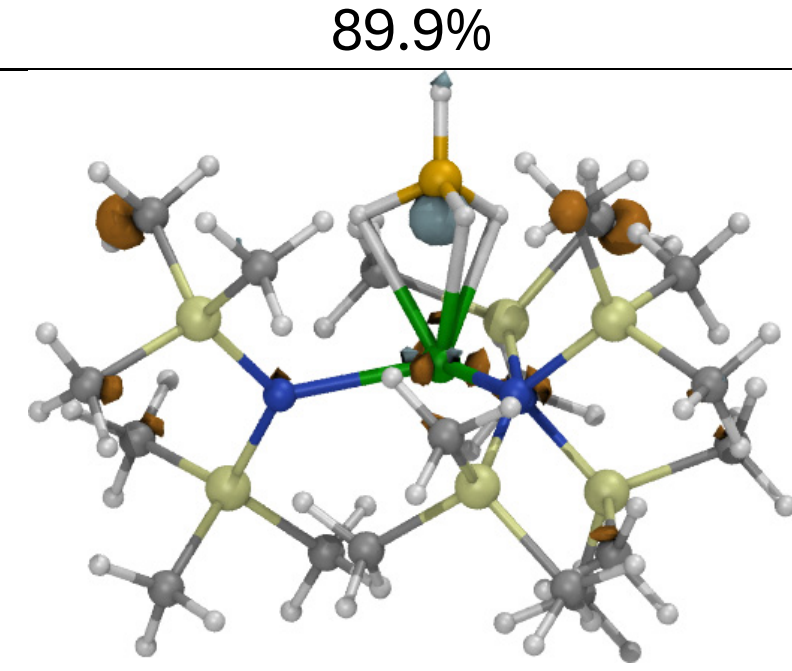 88.1%

			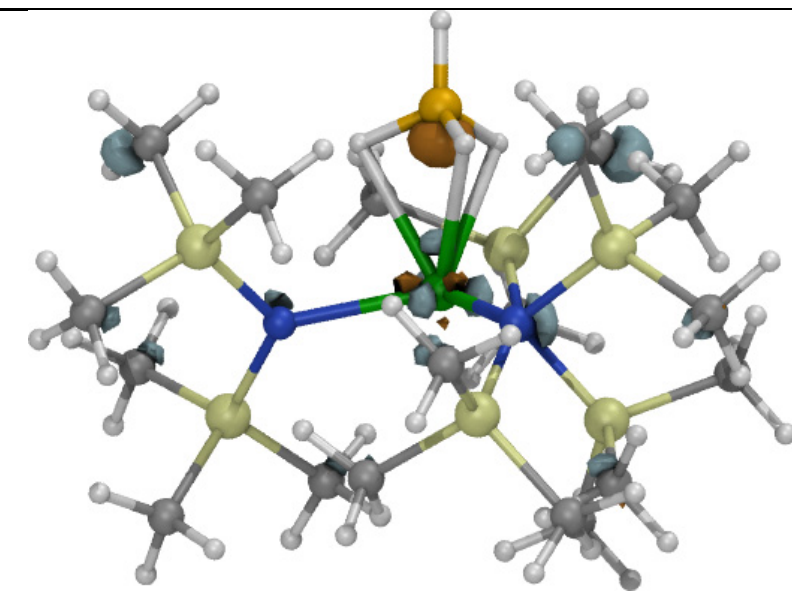 11.9%
90	194.167	0.000700	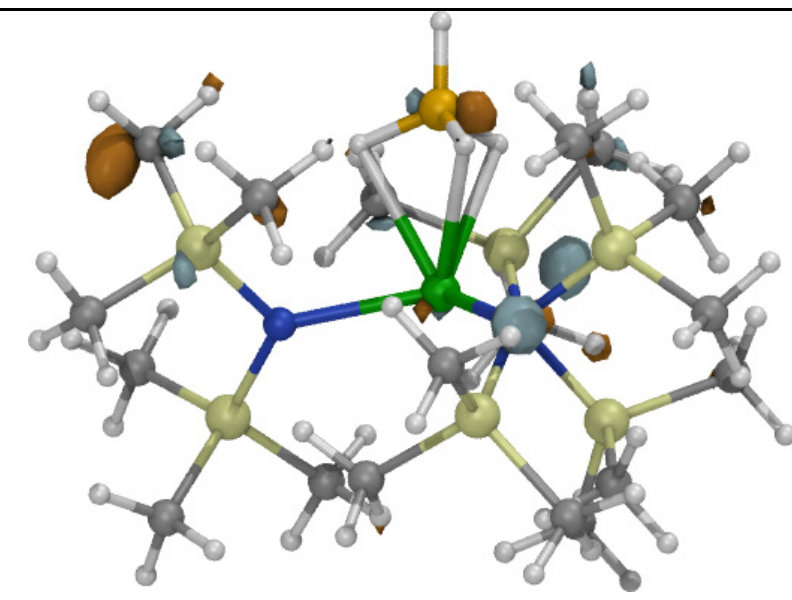 80.7%
			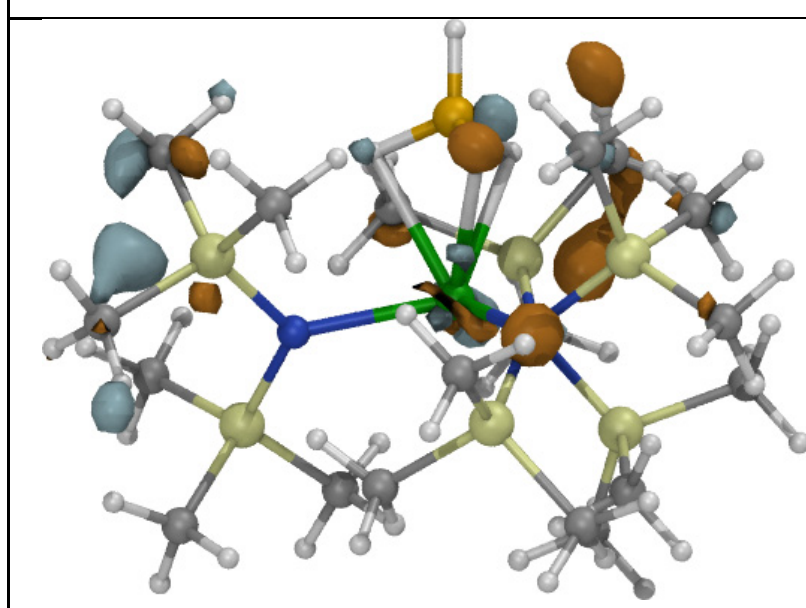 19.3%
91	194.191	0.000251	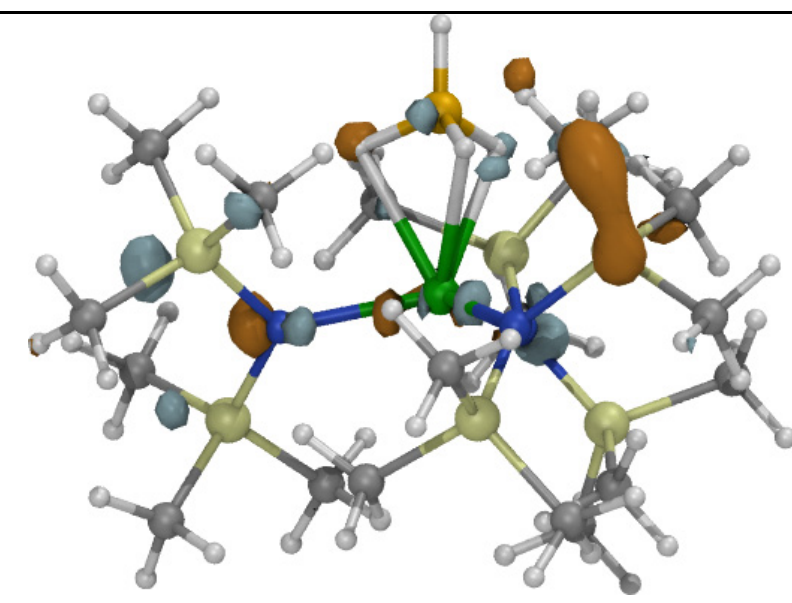 79.4%
			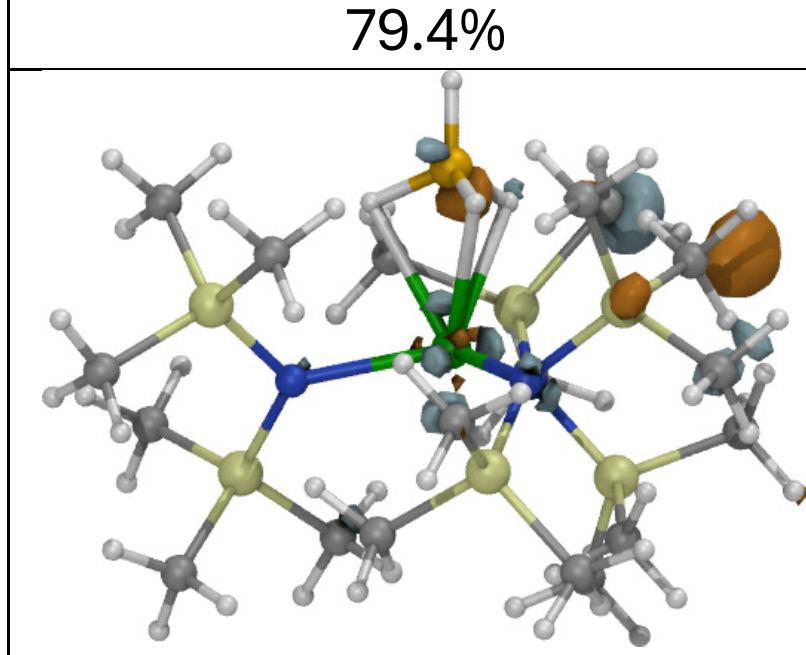 20.6%

92	194.224	0.000280	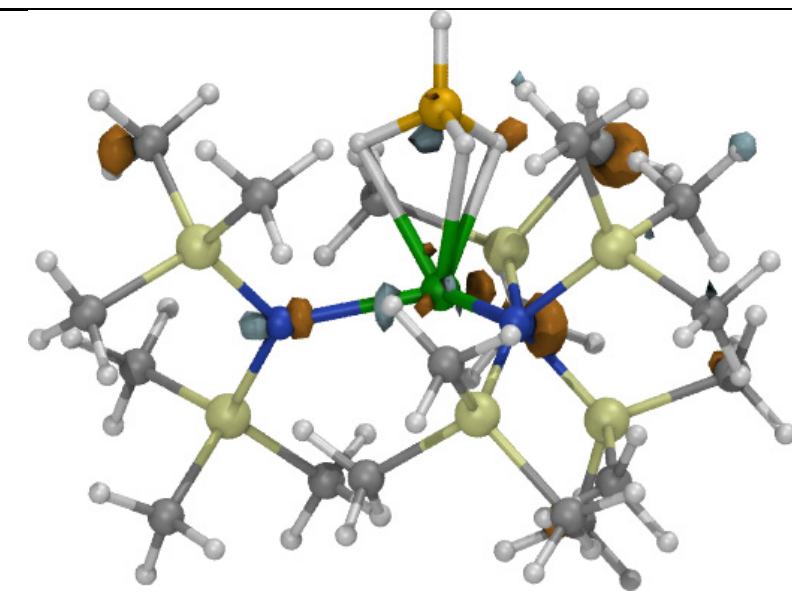 17.4%
			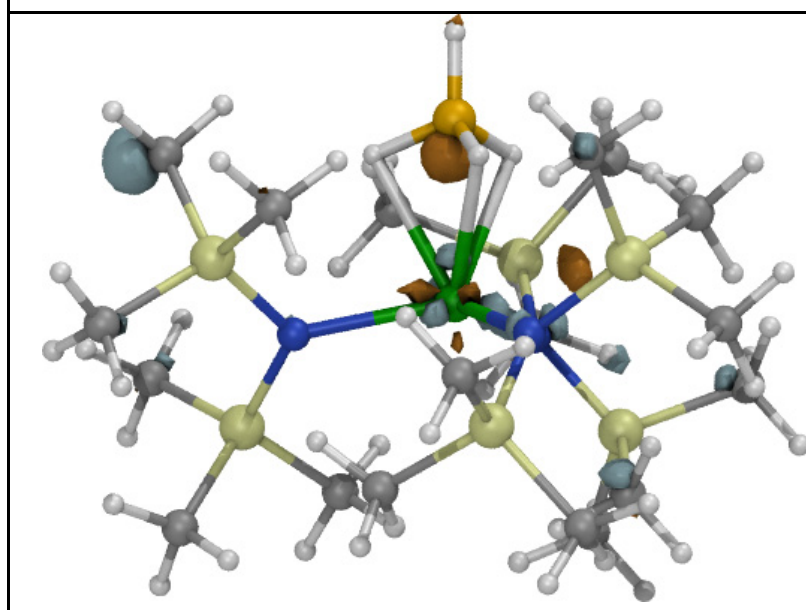 82.6%
93	194.236	0.000397	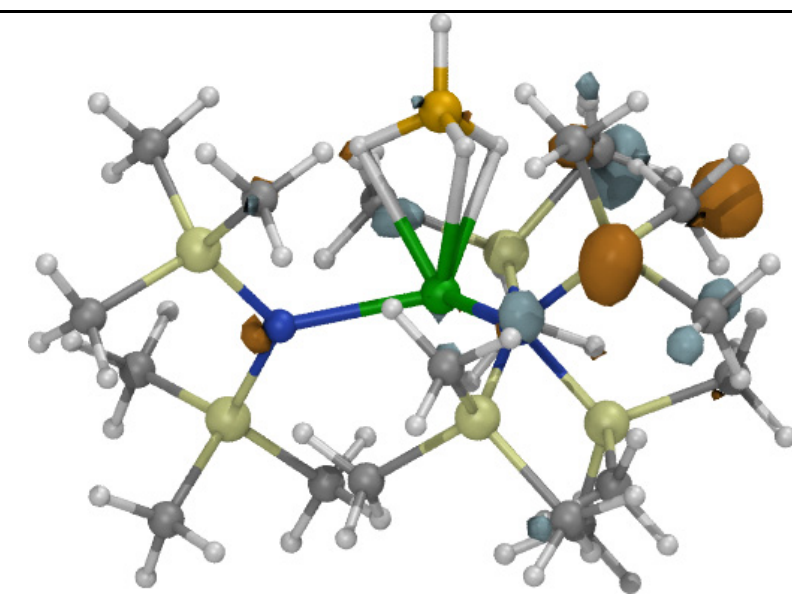 25.5%
			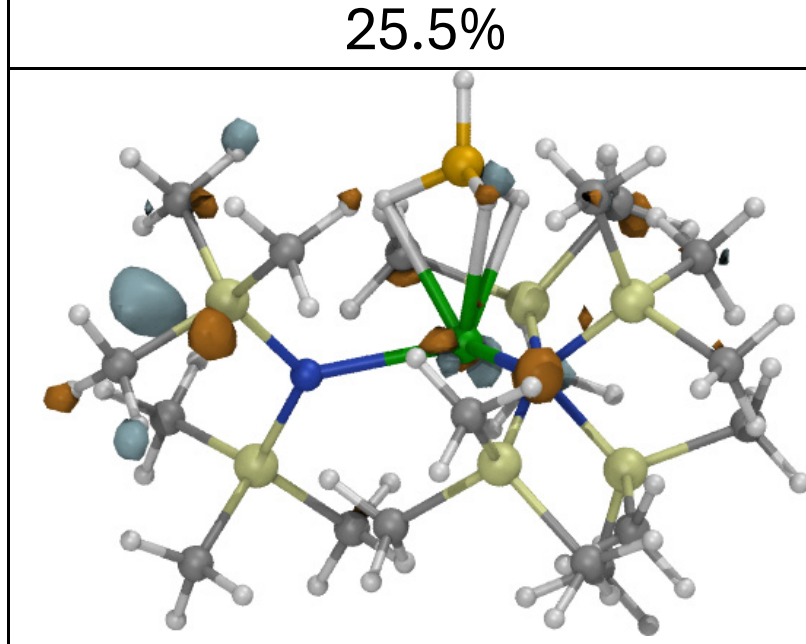 74.5%
94	194.238	0.001022	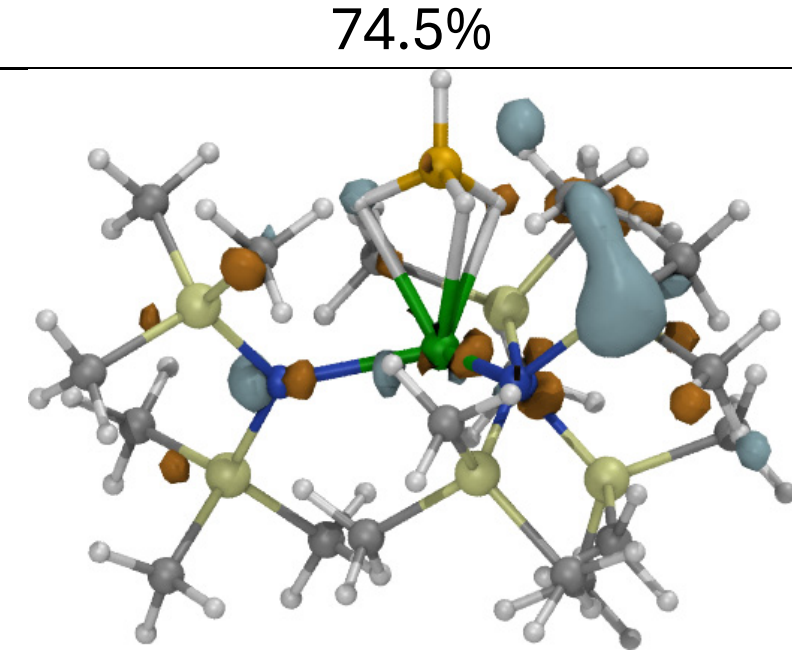 93.3%

95	194.251	0.001228	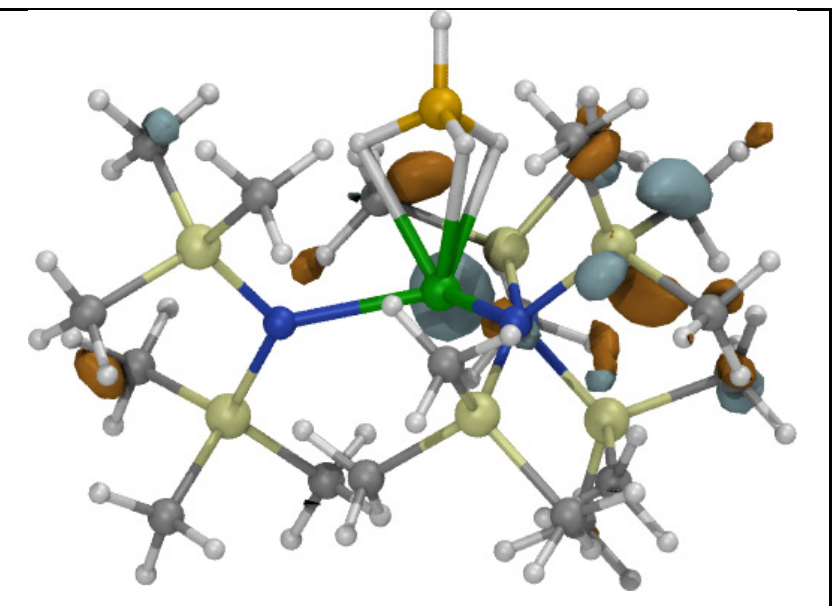<br>86.6%<br>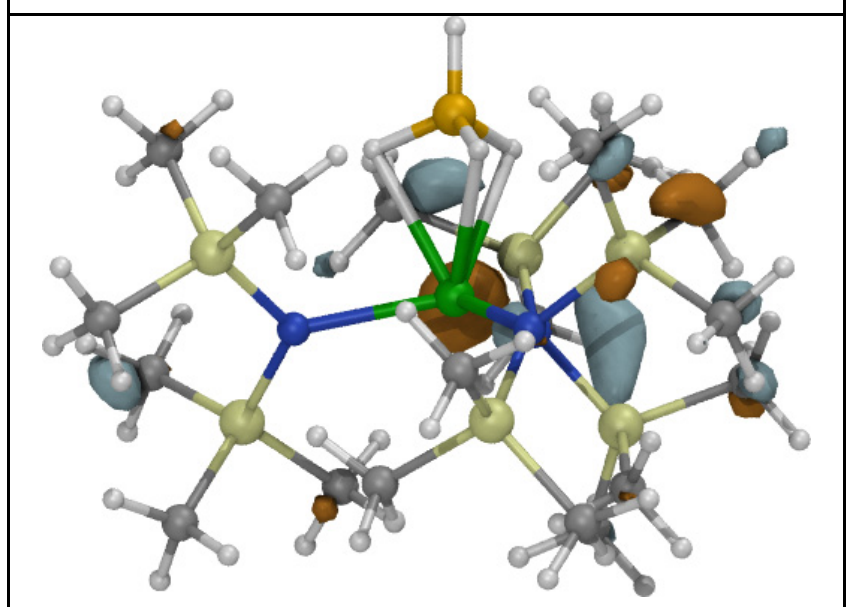<br>13.4%
96	194.270	0.000645	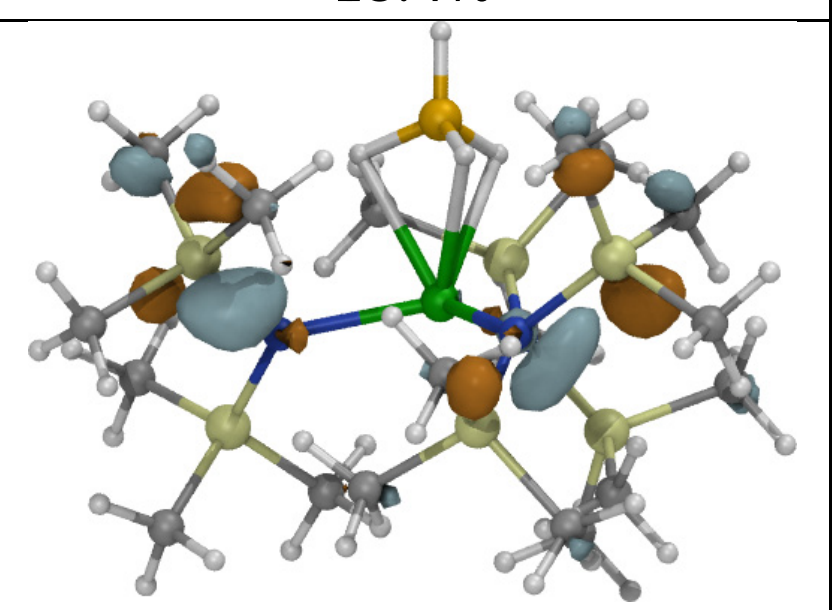<br>52.7%<br>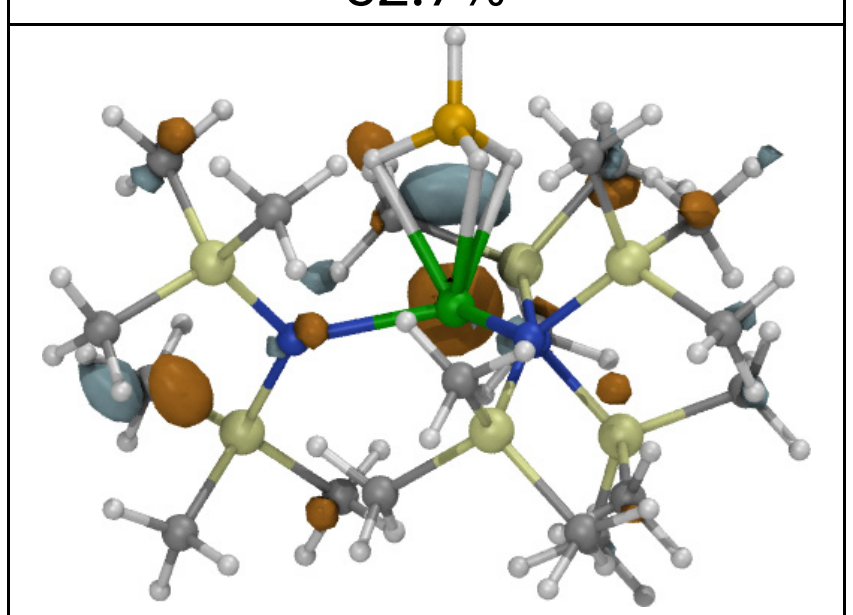<br>47.3%
97	194.315	0.000113	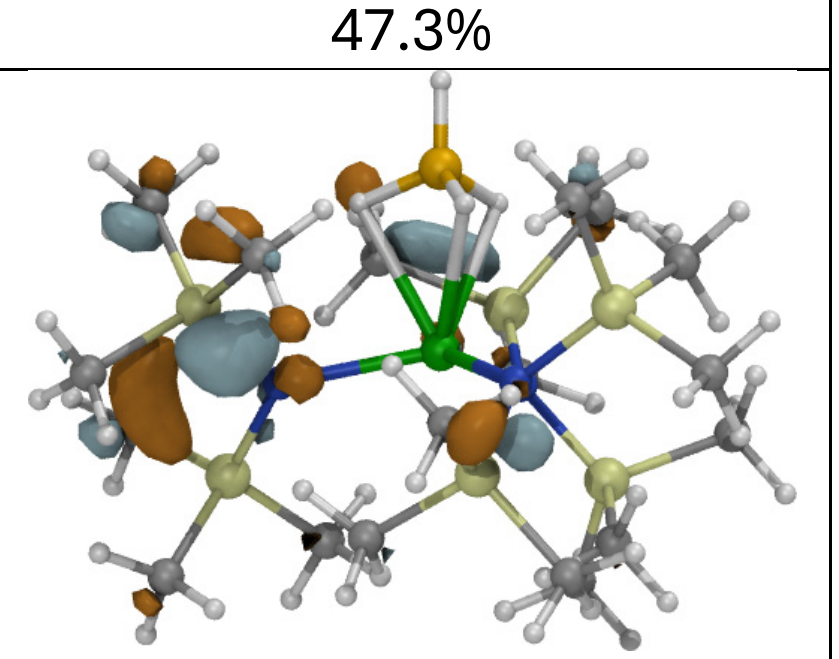<br>49.4%

<table>
<tr><td></td><td></td><td></td><td>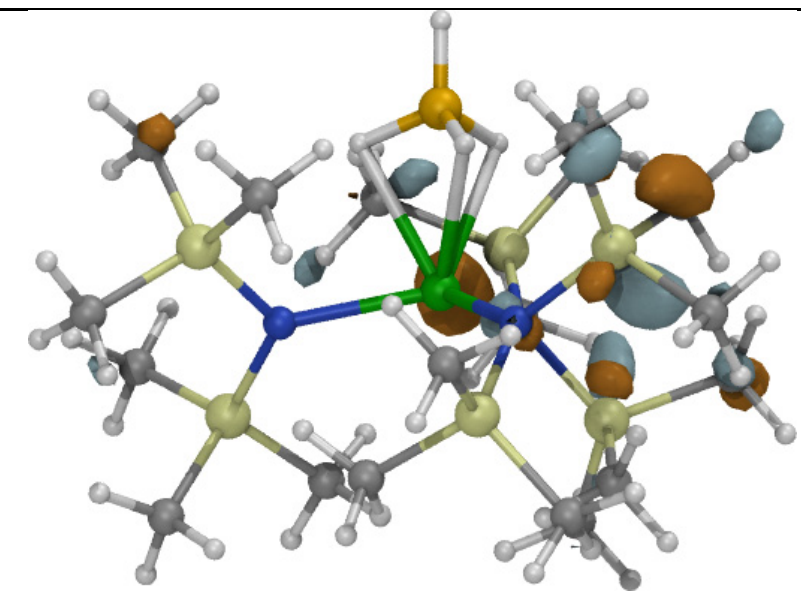
50.6%</td></tr>
<tr><td rowspan="2">98</td><td rowspan="2">194.327</td><td rowspan="2">0.000303</td><td>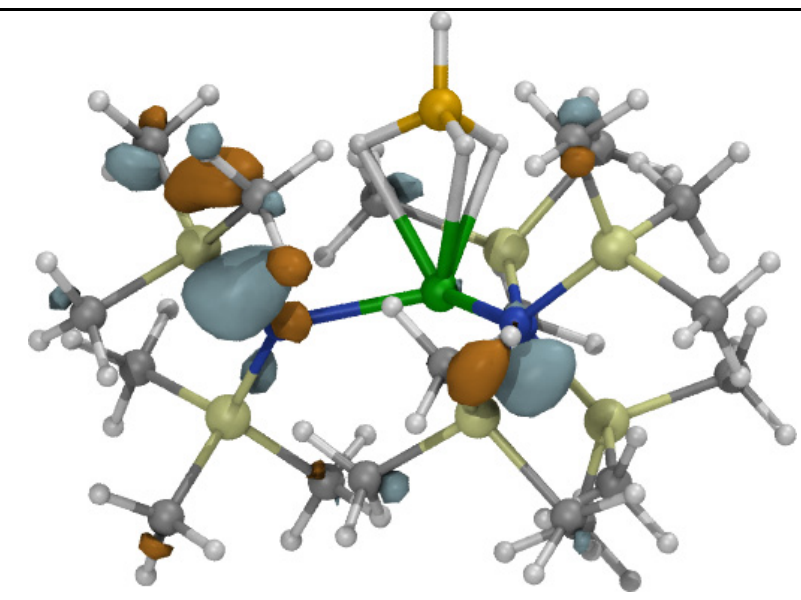
10.7%</td></tr>
<tr><td>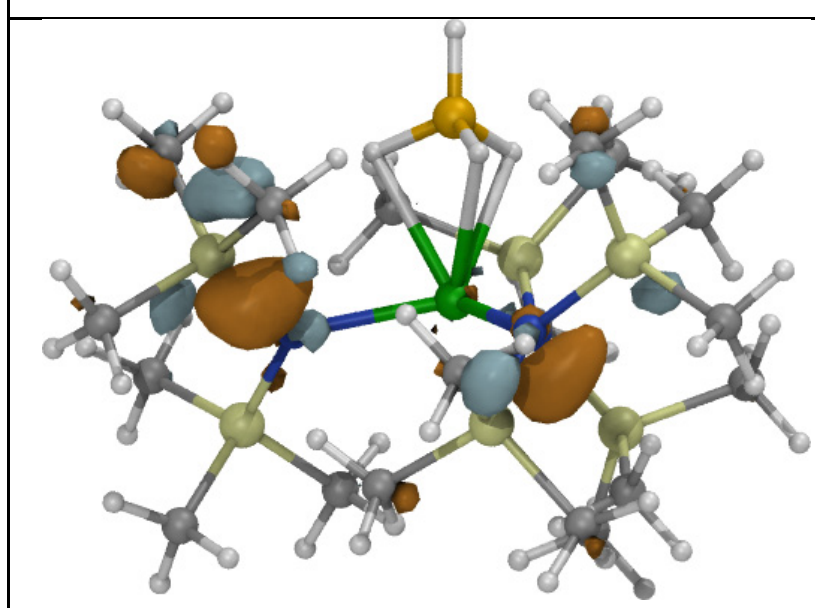
89.3%</td></tr>
<tr><td rowspan="2">99</td><td rowspan="2">194.328</td><td rowspan="2">0.000367</td><td>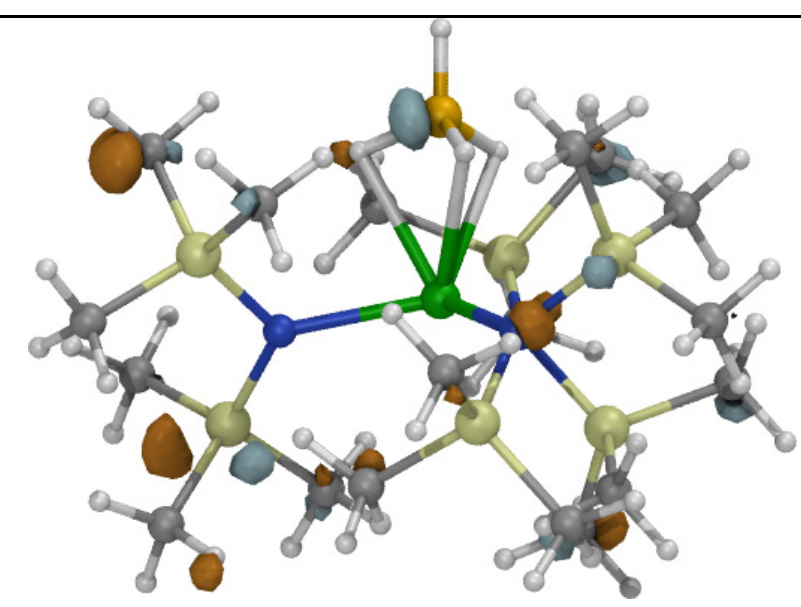
67.3%</td></tr>
<tr><td>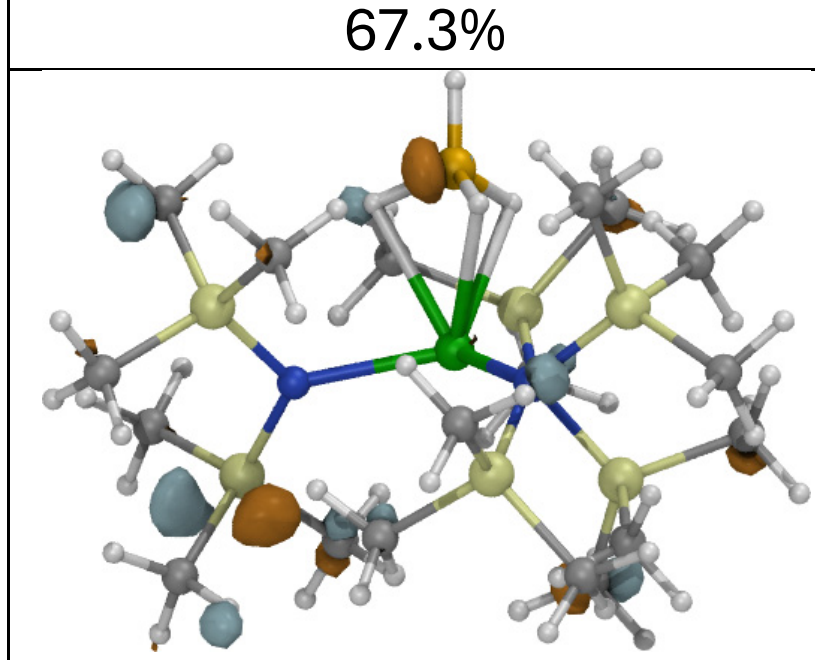
32.7%</td></tr>
</table>

Nuclear Magnetic Resonance

Table S16. Averaged calculated shielding (σ) and chemical shifts (δ) for TMS, boron trifluoride etherate (BTFE), and **1** (M = Th) using the PBE and PBE0 functionals with COSMO (THF).

Complexes	Atoms	Method	σ_{calc} (ppm)	δ_{calc} (ppm)	Δ_{SO} (ppm)	δ_{expt} (ppm)
**TMS**	H	PBE / SO-PBE	31.45 / 31.45	--	0.001	0
		PBE0 / SO-PBE0	31.51 / 31.52	--	0.002	
**BTFE**	B	PBE / SO-PBE	92.85 / 93.85	--	1.00	0
		PBE0 / SO-PBE0	99.36 / 100.38	--	1.02	
**1 (M = Th)**	H_{term}	PBE / SO-PBE	29.39 / 25.79	2.07 / 5.66	3.60	4.22
		PBE0 / SO-PBE0	29.59 / 27.83	1.92 / 5.68	3.76	
	H_2	PBE / SO-PBE	29.36 / 27.33	2.10 / 4.12	2.03	
		PBE0 / SO-PBE0	29.82 / 27.96	1.70 / 5.68	1.86	
	H_3	PBE / SO-PBE	29.36 / 27.33	2.09 / 4.12	2.03	
		PBE0 / SO-PBE0	29.82 / 27.96	1.70 / 5.68	1.86	
	H_4	PBE / SO-PBE	29.36 / 27.33	2.10 / 4.12	2.03	
		PBE0 / SO-PBE0	29.82 / 27.96	1.70 / 5.68	1.86	
	B	PBE / SO-PBE	115.83 / 109.05	-22.98 / -15.21	7.78	-7.99
		PBE0 / SO-PBE0	120.75 / 114.05	-21.39 / -13.68	7.72	

Table S17. Averaged calculated shielding (σ) and chemical shifts (δ) for TMS, boron trifluoride etherate (BTFE), **Hf-Ph**, and **Zr-Ph** using the PBE and PBE0 functionals with COSMO (benzene).

Complexes	Atoms	Method	σ_{calc} (ppm)	δ_{calc} (ppm)	Δ_{SO} (ppm)	δ_{expt} (ppm)
**TMS**	H	PBE / SO-PBE	31.23 / 31.23	--	0.001	0
		PBE0 / SO-PBE0	31.30 / 31.30	--	0.002	
**BTFE**	B	PBE / SO-PBE	92.62 / 93.62	--	1.00	0
		PBE0 / SO-PBE0	99.12 / 100.15	--	1.02	
**Zr-Ph**	H	PBE / SO-PBE	28.51 / 28.28	2.72 / 2.95	0.22	2.8
		PBE0 / SO-PBE0	28.83 / 28.57	2.47 / 2.73	0.26	
	B	PBE / SO-PBE	100.50 / 100.29	-7.88 / -6.66	1.22	-2.1
		PBE0 / SO-PBE0	106.06 / 105.83	-6.94 / -5.68	1.25	
**Hf-Ph**	H	PBE / SO-PBE	28.49 / 27.73	2.74 / 3.50	0.76	3.5
		PBE0 / SO-PBE0	28.82 / 27.99	2.48 / 3.31	0.83	
	B	PBE / SO-PBE	104.57 / 102.63	-12.34 / -9.40	2.94	-4.2
		PBE0 / SO-PBE0	110.12 / 108.12	-10.99 / -7.97	3.03	

Table S18. Decomposition of the components of the H1 (terminal H) shielding tensors at the PBE level by NLMO contributions.

NLMO	σ_{11}	σ_{22}	σ_{33}	σ_{iso}
ΣTh_{core}	-0.706	-0.706	0.829	-0.380
ΣB_{core}	-3.808	-3.809	0.447	-2.390
Th-N	0.222	0.440	-2.933	-0.608
B-H1	26.229	26.228	28.872	27.109
B-H2	2.128	0.959	1.150	1.413
B-H3	0.029	4.987	0.966	1.994
B-H4	4.157	0.370	1.018	1.849
Other	-0.129	-0.343	1.561	1.561
**Total**	**28.121**	**28.126**	**31.911**	**29.386**

Table S19. Decomposition of the components of the H2 shielding tensors at the PBE level by NLMO contributions.

NLMO	σ_{11}	σ_{22}	σ_{33}	σ_{iso}
ΣTh_{core}	7.190	-2.834	-3.002	-0.426
ΣB_{core}	0.899	0.759	0.926	0.861
ΣTh-N	-10.141	3.158	2.948	-1.178
B-H1	0.139	0.547	1.926	0.871
B-H2	26.019	27.461	26.264	26.581
B-H3	0.579	1.639	1.708	1.308
B-H4	0.946	1.505	1.174	1.209
Other	-1.798	-1.141	1.199	0.130
**Total**	**23.833**	**31.094**	**33.143**	**29.356**

Table S20. Decomposition of the components of the H3 shielding tensors at the PBE level by NLMO contributions.

NLMO	σ_{11}	σ_{22}	σ_{33}	σ_{iso}
ΣTh_{core}	7.193	-2.537	-2.931	-0.254
ΣB_{core}	0.903	0.751	0.925	0.860
ΣTh-N	-10.143	3.166	2.950	-1.217
B-H1	0.134	0.538	1.937	0.870
B-H2	0.879	1.157	1.089	1.042
B-H3	26.355	27.648	26.310	26.771
B-H4	0.309	1.799	1.743	1.284
Other	-1.793	-1.423	1.113	0.001
**Total**	**23.837**	**31.099**	**33.136**	**29.357**

Table S21. Decomposition of the components of the H4 shielding tensors at the PBE level by NLMO contributions.

NLMO	σ_{11}	σ_{22}	σ_{33}	σ_{iso}
Th_{core}	7.190	-4.471	-2.489	-0.265
B_{core}	0.901	0.755	0.925	0.860
ΣTh-N	-10.137	3.165	2.945	-1.175
B-H1	0.137	0.552	1.927	0.872
B-H2	0.710	0.868	1.521	1.033
B-H3	0.555	2.081	1.323	1.320
B-H4	26.279	27.625	26.304	26.736
Other	-1.804	0.529	0.673	-0.026
**Total**	**23.831**	**31.104**	**33.129**	**29.355**

Table S22. Decomposition of the components of the B shielding tensors at the PBE level by NLMO contributions.

NLMO	σ_{11}	σ_{22}	σ_{33}	σ_{iso}
Th_{core}	-2.402	-2.401	0.000	-1.263
B_{core}	165.043	165.043	165.066	165.051
ΣTh-N	1.821	1.793	-5.619	-1.044
B-H1	-32.344	-32.266	10.997	-17.871
B-H2	-11.610	-16.892	-9.681	-12.728
B-H3	-1.770	-6.699	-12.329	-6.933
B-H4	-11.790	-1.634	-11.638	-8.354
Other	-0.739	-0.691	-1.775	-1.030
**Total**	**106.209**	**106.253**	**135.021**	**115.828**

Table S23. NLMO Analysis in the Natural Atomic Orbital (NAO) Basis of the four NLMOs contributing to the total shielding tensors based on the PBE density computed in ADF.

NLMO	102	103	104	105
**% Th**	**0.479**	**7.096**	**7.104**	**7.101**
s	2.22	6.38	5.33	5.61
p	3.42	0.61	0.56	0.57
d	71.08	54.54	53.90	54.18
f	23.28	38.47	40.21	39.64
**% B**	**52.335**	**42.530**	**42.160**	**42.248**
s	25.92	26.02	22.04	23.03
p	74.03	73.87	77.85	76.86
d	0.05	0.10	0.11	0.11
f	0.01	0.00	0.00	0.00

% H	46.935	49.782	49.978	49.949
s	99.93	99.62	99.61	99.61
p	0.06	0.37	0.38	0.38
d	0.01	0.01	0.01	0.01

Table S24. NLMO Analysis in the Natural Atomic Orbital (NAO) Basis of the four NLMOs contributing to the total shielding tensors based on the PBE0 density computed in ADF.

NLMO	102	103	104	105
**% Th**	**0.445**	**6.680**	**6.683**	**6.687**
s	2.67	6.51	5.51	5.78
p	3.71	0.61	0.57	0.58
d	72.02	54.88	54.33	54.56
f	21.61	38.00	39.59	39.07
**% B**	**52.041**	**42.200**	**41.846**	**41.929**
s	26.15	25.92	22.21	23.21
p	73.78	73.96	77.65	76.65
d	0.06	0.12	0.13	0.13
f	0.01	0.00	0.00	0.00
**% H**	**47.300**	**50.594**	**50.790**	**50.747**
s	99.92	99.57	99.56	99.56
p	0.07	0.42	0.43	0.43
d	0.01	0.01	0.01	0.01

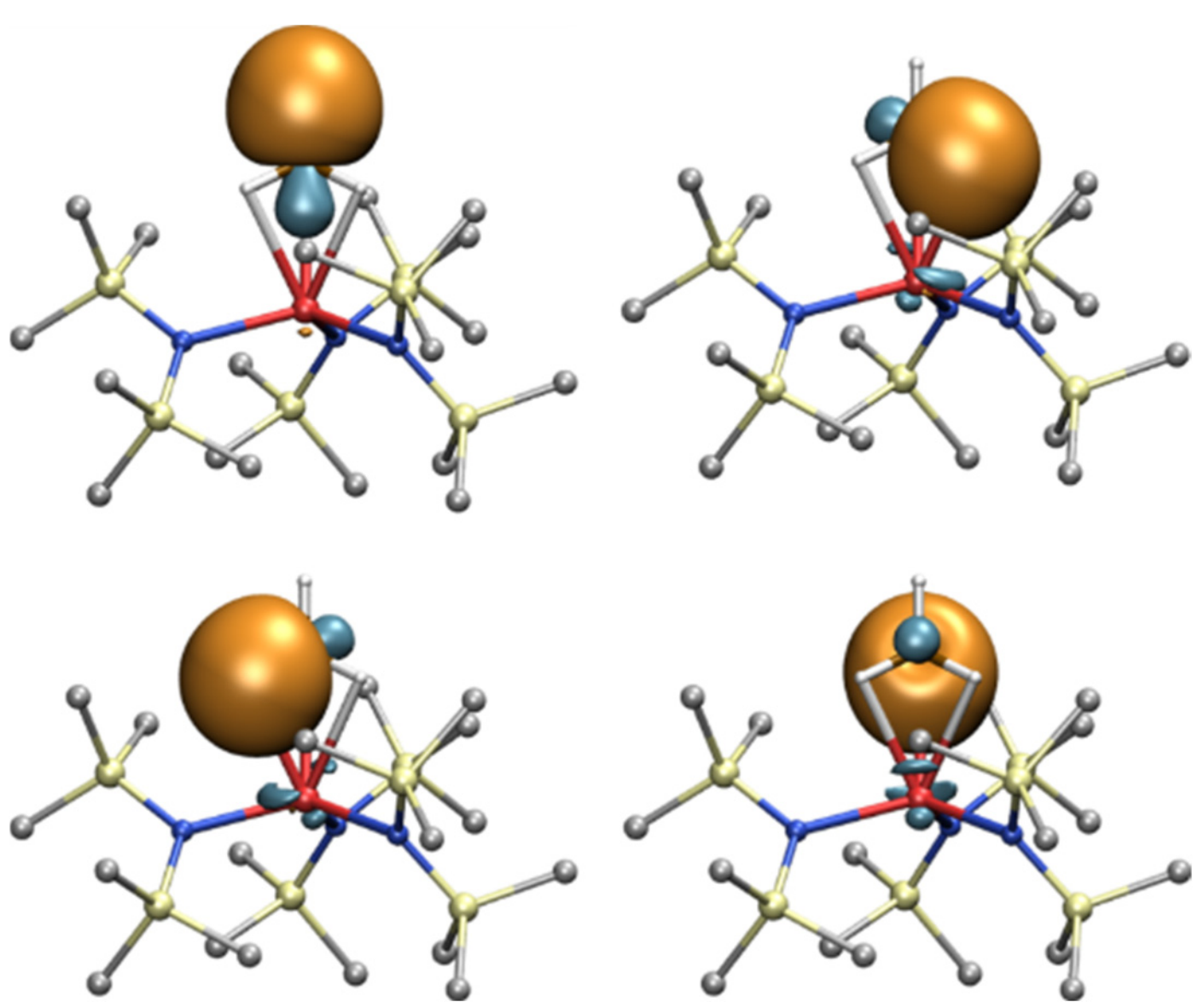

Figure S37. Natural Localized Molecular Orbitals (NLMOs) of **1** (M = Th) with PBE. An isosurface of 0.04 a.u. was employed. Hydrogens attached to carbons hidden for clarity.

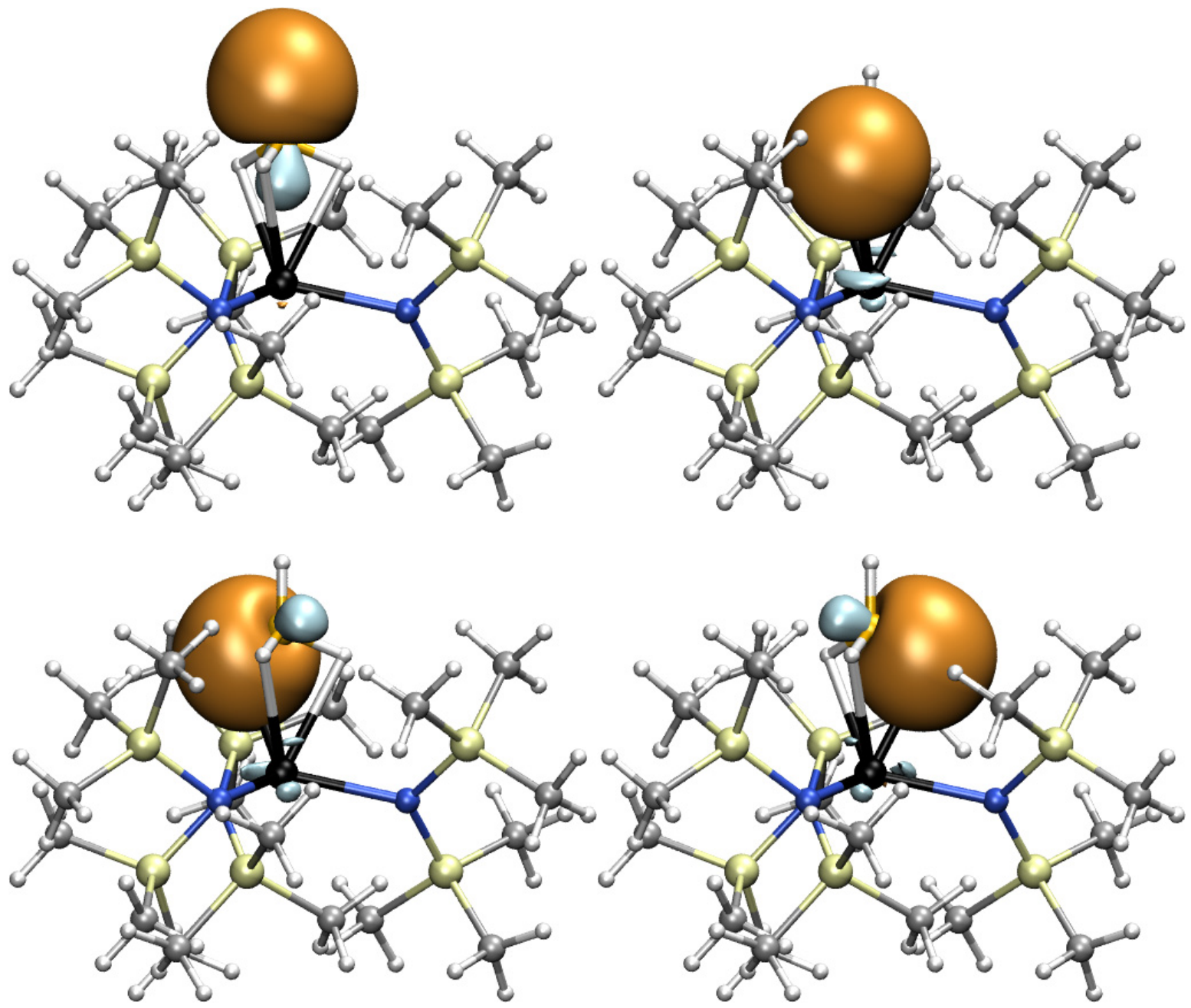

Figure S38. Natural Localized Molecular Orbitals (NLMOs) of **1** (M = Th) with PBE0. An isosurface of 0.04 a.u. was employed.

Table S25. Spin-spin coupling constants (J_{B-H}) and reduced spin-spin-coupling constants (K) for each B-H of **1** (M = Th).

	**H1**	**H2**	**H3**	**H4**	**Avg**
K_{B-H}	34.7	19.6	19.6	19.6	23.4
J_{B-H}	133.8	75.5	75.5	75.5	90.1

XYZ Coordinates

PBE optimized geometry of **1** (M = Th).

Th	-0.0014106	10.6135235	5.8503855
Si	-2.6597801	8.9680500	4.0765491
Si	-3.4891096	10.7707772	6.3748280
N	-2.2085306	10.0914611	5.3573690
C	-5.0967379	11.1448573	5.4470711
H	-5.6140263	10.2390899	5.1001514
H	-5.7766485	11.6608413	6.1438846
H	-4.9432960	11.8050877	4.5817459
C	-2.9303691	12.4428507	7.0692819
H	-2.8112462	13.1909988	6.2719040
H	-3.7209934	12.8100898	7.7431586
H	-2.0056291	12.4203917	7.6636182
C	-3.9542752	9.6294630	7.8064735
H	-3.1081700	9.4393307	8.4810655
H	-4.7615058	10.0848333	8.4016356
H	-4.3179759	8.6584980	7.4400969
C	-3.6027939	7.4650322	4.7301079
H	-4.5944146	7.7445336	5.1161311
H	-3.7611719	6.7360253	3.9198830
H	-3.0608803	6.9579259	5.5413595
C	-3.6968212	9.7255879	2.6859578
H	-3.2300847	10.6300873	2.2710562
H	-3.7751342	8.9878659	1.8710990
H	-4.7171777	9.9835800	2.9956319
C	-1.0963718	8.3443280	3.2084836
H	-0.4031668	7.7946980	3.8581375
H	-1.4104369	7.6616847	2.4026397
H	-0.5292401	9.1536995	2.7189337
Si	-0.0971015	13.7405850	4.0802878
Si	1.8843726	13.5509872	6.3734147
N	0.6523543	12.7850727	5.3575855
C	3.0144470	14.7515018	5.4426211
H	2.4912600	15.6540166	5.0961113
H	3.8044220	15.0800585	6.1369271
H	3.5052179	14.2856518	4.5763739

C	3.0501667	12.2287787	7.0685338
H	3.6363926	11.7482107	6.2716181
H	3.7656858	12.7299133	7.7401498
H	2.5677330	11.4415624	7.6652717
C	1.1331831	14.5264181	7.8061337
H	0.5443291	13.8898945	8.4808501
H	1.9334973	14.9944235	8.4008475
H	0.4764894	15.3294156	7.4412102
C	-0.9232097	15.3087016	4.7392303
H	-0.1829572	16.0258436	5.1243570
H	-1.4769387	15.8127795	3.9316764
H	-1.6309350	15.0925327	5.5524785
C	1.0737134	14.2614989	2.6871745
H	1.6206443	13.4053357	2.2677152
H	0.4715990	14.7014020	1.8758160
H	1.8096361	15.0141898	2.9961677
C	-1.4216812	12.7012229	3.2129423
H	-2.2433336	12.3744336	3.8630509
H	-1.8567352	13.3166523	2.4092145
H	-1.0054592	11.8063755	2.7205660
Si	2.7549392	9.1326833	4.0792701
Si	1.6067899	7.5147757	6.3776024
N	1.5552484	8.9632996	5.3595039
C	2.0883878	5.9352764	5.4510132
H	3.1319223	5.9405246	5.1056068
H	1.9811422	5.0886885	6.1480488
H	1.4411930	5.7373743	4.5849170
C	-0.1208654	7.1622930	7.0715054
H	-0.8301990	6.8966527	6.2740574
H	-0.0441384	6.2905053	7.7409887
H	-0.5611985	7.9723890	7.6703831
C	2.8257362	7.6831437	7.8109911
H	2.5651218	8.5102659	8.4855877
H	2.8349003	6.7560869	8.4056582
H	3.8488532	7.8550350	7.4463252
C	4.5275304	9.0667270	4.7344319
H	4.7787684	8.0684368	5.1227295
H	5.2388506	9.2908769	3.9241137

H	4.6968923	9.7907945	5.5443230
C	2.6196202	7.8545763	2.6895741
H	1.6037995	7.8066009	2.2724323
H	3.2993935	8.1551456	1.8759963
H	2.9055143	6.8419951	3.0002726
C	2.5142603	10.7970376	3.2076812
H	2.6411295	11.6737677	3.8555686
H	3.2645417	10.8649676	2.4036099
H	1.5310486	10.8814838	2.7150631
B	-0.0033914	10.6144273	8.4581751
H	-0.0043783	10.6149548	9.6602808
H	-1.0332959	10.1107131	7.9843850
H	0.0767026	11.7584797	7.9846721
H	0.9485492	9.9738882	7.9861463

PBE optimized geometry of **2** (M = U).

U	0.000000000000	0.000000000000	0.000000000000
Si	-0.892428115808	3.354459394783	0.567601494067
Si	0.981909195770	2.879676860186	-1.780297502758
C	-2.433292164905	2.514280340050	1.275811401045
H	-3.153543171001	2.266356941391	0.482173718642
H	-2.928806654954	3.227171507429	1.954212322010
H	-2.235849775956	1.604455866372	1.858901657820
C	0.169382306238	4.015704908950	1.983432949455
H	0.491013973499	3.222893101186	2.672317943034
H	-0.407548371411	4.751886628300	2.565158917717
H	1.069625341652	4.521517871239	1.605571643140
C	-1.551461369500	4.871646413461	-0.355782297464
H	-0.756484369101	5.537578012971	-0.720086970534
H	-2.165300122512	5.453660626014	0.350313435058
H	-2.191865386380	4.602949778319	-1.207976129100
N	-0.004943278664	2.220854278243	-0.471778625770
C	0.020114700353	3.739598544690	-3.164524377962
H	-0.769531812293	3.099683513692	-3.581566219060
H	0.726549600290	3.970578172445	-3.978252035315
H	-0.439773911352	4.684596709322	-2.849893249584
C	1.904884016151	1.469495857270	-2.643100470955
H	2.588994655595	0.909488058508	-1.992728550174

H	2.505369856428	1.912285613881	-3.454012129809
H	1.225742145290	0.745748974913	-3.123121987399
C	2.270176428586	4.109483258031	-1.144671028031
H	1.802173041785	5.025225311185	-0.754431126507
H	2.939545829079	4.408032049693	-1.966732487051
H	2.888170985288	3.682393624562	-0.341955657410
Si	-2.438836469402	-2.436668271234	0.605974941485
Si	-3.037169789818	-0.587921986437	-1.724388227654
C	-0.940177471045	-3.353299374770	1.308416059268
H	-0.328387706638	-3.799955439496	0.511272041772
H	-1.315964754348	-4.180987543802	1.931536495329
H	-0.286436592820	-2.741644691475	1.943422181505
C	-3.536083090244	-1.857222078715	2.030659369634
H	-3.015484052970	-1.166669944783	2.707708772162
H	-3.857766135859	-2.728530691492	2.622802314099
H	-4.440695417419	-1.352936261513	1.661984859726
C	-3.422890227590	-3.773690949219	-0.307881077757
H	-4.401136965424	-3.427100303801	-0.669195820492
H	-3.610740973390	-4.591554908225	0.406242406827
H	-2.872317819223	-4.198150117377	-1.159423866094
N	-1.925862602929	-1.091761320998	-0.442564516979
C	-3.292816128546	-1.855637074646	-3.106490684965
H	-2.341974220907	-2.175600164416	-3.554480782958
H	-3.889596695866	-1.376364533888	-3.899341847147
H	-3.834001386823	-2.753222205362	-2.783688481154
C	-2.355681704574	0.936632485670	-2.612065465469
H	-2.241571405253	1.818093404942	-1.969309584456
H	-3.063099769649	1.194081606266	-3.416913833542
H	-1.386105890165	0.746230631050	-3.099850235949
C	-4.744320152664	-0.138618455571	-1.045699807722
H	-5.275179659377	-1.016793371729	-0.649915901520
H	-5.364639830144	0.283330165254	-1.852028550077
H	-4.683453076944	0.606685863564	-0.239690154350
Si	3.306200887776	-0.946248480538	0.602975293106
Si	1.985212312652	-2.414448294883	-1.702211150986
C	3.355342447620	0.841271671264	1.227425238599
H	3.530965626129	1.545791427617	0.401141226749
H	4.207262952804	0.935191030522	1.920124604769

H	2.464284966143	1.170546745879	1.779511527167
C	3.349777623041	-2.135786475829	2.069808780204
H	2.501052908775	-1.996916964422	2.752634992588
H	4.274629755503	-1.976192484757	2.646456561287
H	3.345016075964	-3.182173249078	1.731883755676
C	4.956520765974	-1.167761249950	-0.301498636674
H	5.135227164547	-2.197845558830	-0.640257715065
H	5.760436437565	-0.914499016677	0.408279473817
H	5.056751885905	-0.498315485847	-1.167531828145
N	1.883060919946	-1.189102062708	-0.431551550118
C	3.198648652307	-2.006738895185	-3.097084693636
H	2.986580384181	-1.030029368566	-3.554192366275
H	3.085534492891	-2.771948202627	-3.881998666783
H	4.248627415184	-2.011821634801	-2.780109213612
C	0.317742834900	-2.596851654125	-2.575371980474
H	-0.500981976197	-2.918232812936	-1.919947501200
H	0.435661153564	-3.355353422817	-3.366068949710
H	0.002185853878	-1.668580748695	-3.077950068689
C	2.463416474919	-4.109481616459	-1.012381951068
H	3.488545160996	-4.117086656622	-0.613932297214
H	2.417746675440	-4.863718522897	-1.813685112800
H	1.789464565157	-4.431727185206	-0.205856824049
B	-0.000000000131	0.000000000013	2.511728528164
H	0.006943844177	-0.000474638049	3.713846961151
H	-1.096301933947	-0.322952923889	2.026429145371
H	0.832222861202	-0.778641079086	2.019688289104
H	0.262832517623	1.114303657683	2.025368070392

Supporting Information References

(1) Turner, H. W.; Andersen, R. A.; Zalkin, A.; Templeton, D. H. Chloro-, methyl-, and (tetrahydroborato)tris ((hexamethyldisilyl)amido)thorium (IV) and uranium (IV). Crystal structure of (tetrahydroborato)tris ((hexamethyldisilyl)amido)thorium (IV). *Inorganic Chemistry* **1979**, *18*, 1221-1224.

(2) Smiles, D. E.; Wu, G.; Kaltsoyannis, N.; Hayton, T. W. Thorium–ligand multiple bonds via reductive deprotection of a trityl group. *Chemical Science* **2015**, *6*, 3891-3899.

(3) Cantat, T.; Scott, B. L.; Kiplinger, J. L. Convenient access to the anhydrous thorium tetrachloride complexes $ThCl_4$ $(DME)_2$, $ThCl_4$(1,4-dioxane)$_2$ and $ThCl_4(THF)_{3.5}$ using commercially available and inexpensive starting materials. *Chemical Communications* **2010**, *46*, 919-921.

(4) Kiplinger, J. L.; Morris, D. E.; Scott, B. L.; Burns, C. J. Convenient synthesis, structure, and reactivity of $(C_5Me_5)U(CH_2C_6H_5)_3$: a simple strategy for the preparation of monopentamethylcyclopentadienyl uranium (IV) complexes. *Organometallics* **2002**, *21*, 5978-5982.

(5) Schlesinger, H. I.; Brown, H. C.; Hyde, E. K. The Preparation of Other Borohydrides by Metathetical Reactions Utilizing the Alkali Metal Borohydrides[1]. *Journal of the American Chemical Society* **1953**, *75*, 209-213.

(6) *SMART Apex II*; Bruker AXS Inc.: Madison, WI, 2005.

(7) *SAINT Software User's Guide*; Bruker AXS Inc.: Madison, WI, 2005.

(8) *SADABS*; Univeristy of Gottingen: Germany, 2005.

(9) *SHELXTL PC*; Bruker AXS Inc.: Madison, WI, 2005.

(10) Python Software Foundation. Python, Version 3.12.3; Python Software Foundation: Wilmington, DE, 2024. https://www.python.org/.

(11) Hunter, J. D. Matplotlib: A 2D Graphics Environment. *Computing in Science and Engineering* **2007**, *9*, 90-95.

(12) Harris, C. R.; Millman, K. J.; van der Walt, S. J.; Gommers, R.; Virtanen, P.; Cournapeau, D.; Wieser, E.; Taylor, J.; Berg, S.; Smith, N. J.; Kern, R.; Picus, M.; Hoyer, S.; van Kerkwijk, M. H.; Brett, M.; Haldane, A.; del Río, J. F.; Wiebe, M.; Peterson, P.; Gérard-Marchant, P.; Sheppard, K.; Reddy, T.; Weckesser, W.; Abbasi, H.; Gohlke, C.; Oliphant, T. E. Array programming with NumPy. *Nature* **2020**, *585*, 357-362.

(13) Ousterhout, J. K. Tcl and the Tk Toolkit; Addison-Wesley: Reading, MA, 1994.

(14) The Pandas Development Team. Pandas-dev/pandas: Pandas. v2.3.3, Zenodo 2025. DOI: 10.5281/zenodo.17229934

(15) Ravel, B.; Newville, M. ATHENA, ARTEMIS, HEPHAESTUS: data analysis for x-ray absorption spectroscopy using IFEFFIT. *Journal of Synchrotron Radiation* **2005**, *12*, 537-541.

(16) IGOR Pro (Version 10.01); WaveMetrics, Inc: Lake Oswego, OR, USA, 2025.

(17) Cao, X.; Dolg, M.; Stoll, H. Valence basis sets for relativistic energy-consistent small-core actinide pseudopotentials. *The Journal of Chemical Physics* **2003**, *118*, 487-496.

(18) Weigend, F.; Ahlrichs, R. Balanced basis sets of split valence, triple zeta valence and quadruple zeta valence quality for H to Rn: Design and assessment of accuracy. *Physical Chemistry Chemical Physics* **2005**, *7*, 3297-3305.

(19) Treutler, O.; Ahlrichs, R. Efficient molecular numerical integration schemes. *The Journal of Chemical Physics* **1995**, *102*, 346-354.

(20) Weigend, F.; Häser, M.; Patzelt, H.; Ahlrichs, R. RI-MP2: optimized auxiliary basis sets and demonstration of efficiency. *Chemical Physics Letters* **1998**, *294*, 143-152.

(21) Neese, F. The ORCA program system. *WIREs Computational Molecular Science* **2012**, *2*, 73-78.

(22) Neese, F. Software Update: The ORCA Program System—Version 6.0. *WIREs Computational Molecular Science* **2025**, *15*, e70019.

(23) Pantazis, D. A.; Chen, X.-Y.; Landis, C. R.; Neese, F. All-Electron Scalar Relativistic Basis Sets for Third-Row Transition Metal Atoms. *Journal of Theory and Computation* **2008**, *4*, 908-919.

(24) Rolfes, J. D.; Neese, F.; Pantazis, D. A. All-electron scalar relativistic basis sets for the elements Rb–Xe. *Journal of Computational Chemistry* **2020**, *41*, 1842-1849.

(25) Weigend, F.; Ahlrichs, R. Balanced basis sets of split valence, triple zeta valence and quadruple zeta valence quality for H to Rn: Design and assessment of accuracy. *Physical Chemistry Chemical Physics* **2005**, *7*, 3297-3305.

(26) Izsák, R.; Hansen, A.; Neese, F. The resolution of identity and chain of spheres approximations for the LPNO-CCSD singles Fock term. *Molecular Physics* **2012**, *110*, 2413-2417.

(27) Izsák, R.; Neese, F. An overlap fitted chain of spheres exchange method. *The Journal of Chemical Physics* **2011**, *135*, 144105.

(28) Izsák, R.; Neese, F.; Klopper, W. Robust fitting techniques in the chain of spheres approximation to the Fock exchange: The role of the complementary space. *The Journal of Chemical Physics* **2013**, *139*, 094111.

(29) Annegarn, M.; Kahk, J. M.; Lischner, J. Combining Time-Dependent Density Functional Theory and the ΔSCF Approach for Accurate Core-Electron Spectra. *Journal of Chemical Theory and Computation* **2022**, *18*, 7620-7629.

(30) Stephens, P. J.; Devlin, F. J.; Chabalowski, C. F.; Frisch, M. J. Ab Initio Calculation of Vibrational Absorption and Circular Dichroism Spectra Using Density Functional Force Fields. *The Journal of Physical Chemistry* **1994**, *98*, 11623-11627.

(31) Tao, J.; Perdew, J. P.; Staroverov, V. N.; Scuseria, G. E. Climbing the Density Functional Ladder: Nonempirical Meta--Generalized Gradient Approximation Designed for Molecules and Solids. *Physical Review Letters* **2003**, *91*, 146401.

(32) Staroverov, V. N.; Scuseria, G. E.; Tao, J.; Perdew, J. P. Comparative assessment of a new nonempirical density functional: Molecules and hydrogen-bonded complexes. *The Journal of Chemical Physics* **2003**, *119*, 12129-12137.

(33) Caldeweyher, E.; Bannwarth, C.; Grimme, S. Extension of the D3 dispersion coefficient model. *The Journal of Chemical Physics* **2017**, *147*, 034112.

(34) Caldeweyher, E.; Ehlert, S.; Hansen, A.; Neugebauer, H.; Spicher, S.; Bannwarth, C.; Grimme, S. A generally applicable atomic-charge dependent London dispersion correction. *The Journal of Chemical Physics* **2019**, *150*, 154122.

(35) E.J. Baerends, T. Z., A.J. Atkins, J. Autschbach, O. Baseggio, D. Bashford, A. Bérces, F.M. Bickelhaupt, C. Bo, P.M. Boerrigter, C. Cappelli, L. Cavallo, C. Daul, D.P. Chong, D.V. Chulhai, L. Deng, R.M. Dickson, J.M. Dieterich, F. Egidi, D.E. Ellis, M. van Faassen, L. Fan, T.H. Fischer, A. Förster, C. Fonseca Guerra, M. Franchini, A. Ghysels, A. Giammona, S.J.A. van Gisbergen, A. Goez, A.W. Götz, J.A. Groeneveld, O.V. Gritsenko, M. Grüning, S. Gusarov, F.E. Harris, P. van den Hoek, Z. Hu, C.R. Jacob, H. Jacobsen, L. Jensen, L. Joubert, J.W. Kaminski, G. van Kessel, C. König, F. Kootstra, A. Kovalenko, M.V. Krykunov, P. Lafiosca, E. van Lenthe, D.A. McCormack, M. Medves, A. Michalak, M. Mitoraj, S.M. Morton, J. Neugebauer, V.P. Nicu, L. Noodleman, V.P. Osinga, S. Patchkovskii, M. Pavanello, C.A. Peeples, P.H.T. Philipsen, D. Post, C.C. Pye, H. Ramanantoanina, P. Ramos, W. Ravenek, M. Reimann, J.I. Rodríguez, P. Ros, R. Rüger, P.R.T. Schipper, D. Schlüns, H. van Schoot, G. Schreckenbach, J.S. Seldenthuis, M. Seth, J.G. Snijders, M. Solà, M. Stener, M. Swart, D. Swerhone, V. Tognetti, G. te Velde, P. Vernooijs, L. Versluis, L. Visscher, O. Visser, F. Wang, T.A. Wesolowski, E.M. van Wezenbeek, G. Wiesenekker, S.K. Wolff, T.K. Woo, A.L. Yakovlev. ADF 2024. SCM, Theorhetical Chemistry: Vrije University, Amsterdam, The Netherlands, 2024.

(36) Baerends, E. J.; Aguirre, N. F.; Austin, N. D.; Autschbach, J.; Bickelhaupt, F. M.; Bulo, R.; Cappelli, C.; van Duin, A. C. T.; Egidi, F.; Fonseca Guerra, C.; Förster, A.; Franchini, M.; Goumans, T. P. M.; Heine, T.; Hellström, M.; Jacob, C. R.; Jensen, L.; Krykunov, M.; van Lenthe, E.; Michalak, A.; Mitoraj, M. M.; Neugebauer, J.; Nicu, V. P.; Philipsen, P.; Ramanantoanina, H.; Rüger, R.; Schreckenbach, G.; Stener, M.; Swart, M.; Thijssen, J. M.; Trnka, T.; Visscher, L.; Yakovlev, A.; van Gisbergen, S. The Amsterdam Modeling Suite. *The Journal of Chemical Physics* **2025**, *162*, 162501.

(37) Lenthe, E. v.; Baerends, E. J.; Snijders, J. G. Relativistic regular two‐component Hamiltonians. *The Journal of Chemical Physics* **1993**, *99*, 4597-4610.

(38) van Lenthe, E.; Baerends, E. J.; Snijders, J. G. Relativistic total energy using regular approximations. *The Journal of Chemical Physics* **1994**, *101*, 9783-9792.

(39) Wolff, S. K.; Ziegler, T.; van Lenthe, E.; Baerends, E. J. Density functional calculations of nuclear magnetic shieldings using the zeroth-order regular approximation (ZORA) for relativistic effects: ZORA nuclear magnetic resonance. *The Journal of Chemical Physics* **1999**, *110*, 7689-7698.

(40) Schreckenbach, G.; Ziegler, T. Calculation of NMR Shielding Tensors Using Gauge-Including Atomic Orbitals and Modern Density Functional Theory. *The Journal of Physical Chemistry* **1995**, *99*, 606-611.

(41) Autschbach, J.; Zheng, S. Analyzing Pt chemical shifts calculated from relativistic density functional theory using localized orbitals: The role of Pt 5d lone pairs. *Magnetic Resonance in Chemistry* **2008**, *46*, S45-S55.

(42) Autschbach, J. Analyzing NMR shielding tensors calculated with two-component relativistic methods using spin-free localized molecular orbitals. *The Journal of Chemical Physics* **2008**, *128*, 164112.

(43) Krykunov, M.; Ziegler, T.; van Lenthe, E. Implementation of a Hybrid DFT Method for Calculating NMR Shieldings Using Slater-Type Orbitals with Spin−Orbital Coupling Included. Applications to ^{187}Os, ^{195}Pt, and ^{13}C in Heavy-Metal Complexes. *The Journal of Physical Chemistry A* **2009**, *113*, 11495-11500.

(44) Autschbach, J.; Zurek, E. Relativistic Density-Functional Computations of the Chemical Shift of ^{129}Xe in Xe@C_{60}. *The Journal of Physical Chemistry A* **2003**, *107*, 4967-4972.

(45) Novotný, J.; Vícha, J.; Bora, P. L.; Repisky, M.; Straka, M.; Komorovsky, S.; Marek, R. Linking the Character of the Metal–Ligand Bond to the Ligand NMR Shielding in Transition-Metal Complexes: NMR Contributions from Spin–Orbit Coupling. *Journal of Chemical Theory and Computation* **2017**, *13*, 3586-3601.

(46) Vícha, J.; Komorovsky, S.; Repisky, M.; Marek, R.; Straka, M. Relativistic Spin–Orbit Heavy Atom on the Light Atom NMR Chemical Shifts: General Trends Across the Periodic Table Explained. *Journal of Chemical Theory and Computation* **2018**, *14*, 3025-3039.

(47) Perdew, J. P.; Burke, K.; Ernzerhof, M. Generalized Gradient Approximation Made Simple. *Physical Review Letters* **1996**, *77*, 3865-3868.

(48) Becke, A. D. Density-functional exchange-energy approximation with correct asymptotic behavior. *Physical Review A* **1988**, *38*, 3098-3100.

(49) Perdew, J. P.; Yue, W. Accurate and simple density functional for the electronic exchange energy: Generalized gradient approximation. *Physical Review B* **1986**, *33*, 8800-8802.

(50) Perdew, J. P.; Chevary, J. A.; Vosko, S. H.; Jackson, K. A.; Pederson, M. R.; Singh, D. J.; Fiolhais, C. Atoms, molecules, solids, and surfaces: Applications of the generalized gradient approximation for exchange and correlation. *Physical Review B* **1992**, *46*, 6671-6687.

(51) Ernzerhof, M.; Scuseria, G. E. Assessment of the Perdew–Burke–Ernzerhof exchange-correlation functional. *The Journal of Chemical Physics* **1999**, *110*, 5029-5036.

(52) Seth, M.; Ziegler, T. Range-Separated Exchange Functionals with Slater-Type Functions. *Journal of Chemical Theory and Computation* **2012**, *8*, 901-907.

(53) Van Lenthe, E.; Baerends, E. J. Optimized Slater-type basis sets for the elements 1–118. *Journal of Computational Chemistry* **2003**, *24*, 1142-1156.

(54) Pye, C. C.; Ziegler, T. An implementation of the conductor-like screening model of solvation within the Amsterdam density functional package. *Theoretical Chemistry Accounts* **1999**, *101*, 396-408.

(55) Glendening, E. D.; Landis, C. R.; Weinhold, F. Natural bond orbital methods. *WIREs Computational Molecular Science* **2012**, *2*, 1-42.

(56) Glendening, E. D.; Landis, C. R.; Weinhold, F. NBO 6.0: Natural bond orbital analysis program. *Journal of Computational Chemistry* **2013**, *34*, 1429-1437.

(57) Autschbach, J.; Ziegler, T. Nuclear spin–spin coupling constants from regular approximate relativistic density functional calculations. II. Spin–orbit coupling effects and anisotropies. *The Journal of Chemical Physics* **2000**, *113*, 9410-9418.

(58) Bryce, D. L.; Autschbach, J. Relativistic hybrid density functional calculations of indirect nuclear spin–spin coupling tensors — Comparison with experiment for diatomic alkali metal halides. *Canadian Journal of Chemistry* **2009**, *87*, 927-941.

(59) Autschbach, J. Two-component relativistic hybrid density functional computations of nuclear spin-spin coupling tensors using Slater-type basis sets and density-fitting techniques. *The Journal of Chemical Physics* **2008**, *129*, 164112.

(60) Woo, T. K.; Patchkovskii, S.; Ziegler, T. Atomic scale modeling of polymerization catalysts. *Computing in Science & Engineering* **2000**, *2*, 28-37.

(61) Rodríguez, J. I.; Bader, R. F. W.; Ayers, P. W.; Michel, C.; Götz, A. W.; Bo, C. A high performance grid-based algorithm for computing QTAIM properties. *Chemical Physics Letters* **2009**, *472*, 149-152.

(62) Rodríguez, J. I. An efficient method for computing the QTAIM topology of a scalar field: The electron density case. *Journal of Computational Chemistry* **2013**, *34*, 681-686.

(63) Ziegler, T.; Rauk, A. A theoretical study of the ethylene-metal bond in complexes between copper(1+), silver(1+), gold(1+), platinum(0) or platinum(2+) and ethylene, based on the Hartree-Fock-Slater transition-state method. *Inorganic Chemistry* **1979**, *18*, 1558-1565.

(64) Ziegler, T.; Rauk, A. Carbon monoxide, carbon monosulfide, molecular nitrogen, phosphorus trifluoride, and methyl isocyanide as σ donors and π acceptors. A theoretical study by the Hartree-Fock-Slater transition-state method. *Inorganic Chemistry* **1979**, *18*, 1755-1759.

(65) Mitoraj, M. P.; Michalak, A.; Ziegler, T. A Combined Charge and Energy Decomposition Scheme for Bond Analysis. *Journal of Chemical Theory and Computation* **2009**, *5*, 962-975.
